# Hetero-Diels–Alder Reaction between Singlet Oxygen and Anthracene Drives Integrative Cage Self-Sorting

**DOI:** 10.1021/jacs.3c04228

**Published:** 2023-08-23

**Authors:** Yuchong Yang, Tanya K. Ronson, Dingyu Hou, Jieyu Zheng, Ilma Jahović, Kai Hong Luo, Jonathan R. Nitschke

**Affiliations:** † Yusuf Hamied Department of Chemistry, University of Cambridge, Cambridge CB2 1EW, United Kingdom; ‡ Department of Mechanical Engineering, University College London, London WC1E 7JE, United Kingdom

## Abstract

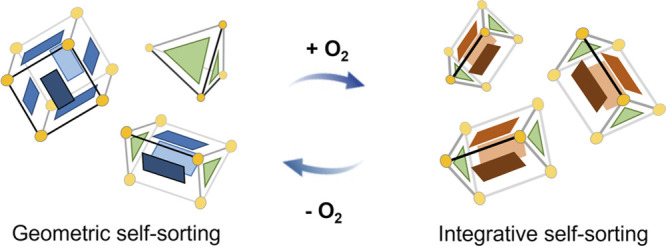

A Zn^II^_8_L_6_ pseudocube containing anthracene-centered ligands, a Zn^II^_4_L′_4_ tetrahedron with a similar side length as the cube, and a trigonal prism Zn^II^_6_L_3_L′_2_ were formed in equilibrium from a common set of subcomponents. Hetero-Diels–Alder reaction with photogenerated singlet oxygen transformed the anthracene-containing “L” ligands into endoperoxide “L^O^” ones and ultimately drove the integrative self-sorting to form the trigonal prismatic cage Zn^II^_6_L^O^_3_L′_2_ exclusively. This Zn^II^_6_L^O^_3_L′_2_ structure lost dioxygen in a retro-Diels–Alder reaction after heating, which resulted in reversion to the initial Zn^II^_8_L_6_ + Zn^II^_4_L′_4_ ⇌ 2 × Zn^II^_6_L_3_L′_2_ equilibrating system. Whereas the Zn^II^_8_L_6_ pseudocube had a cavity too small for guest encapsulation, the Zn^II^_6_L_3_L′_2_ and Zn^II^_6_L^O^_3_L′_2_ trigonal prisms possessed peanut-shaped internal cavities with two isolated compartments divided by bulky anthracene panels. Guest binding was also observed to drive the equilibrating system toward exclusive formation of the Zn^II^_6_L_3_L′_2_ structure, even in the absence of reaction with singlet oxygen.

Self-sorting processes in chemical systems^1^ enable multiple structures to form from a common pool of subunits, thereby potentially exercising their functions in parallel within the same solution. Understanding these processes can shed light on the complex self-assembly pathways in natural systems,^1a^ as well as enable the design of chemical systems that serve useful purposes.^2^ Artificial self-sorting systems have been developed where subunits are bound together by hydrogen bonds,^3^ metal–ligand coordination,^4^ aromatic stacking interactions,^5^ and electrostatic attraction.^6^

Coordination cages can be produced in self-sorting systems where selectivity is driven by thermodynamic and geometric parameters.^7^ These cages can undergo structural changes in response to different stimuli,^8^ such as post-assembly modification.^9^

As shown in Figure 1, Zn^II^_8_L_6_ pseudocubic cage **1** and Zn^II^_4_L′_4_ tetrahedral cage **2** self-assembled from trigonal subcomponent **A** and anthracene-centered tetragonal subcomponent **B**, respectively. As a consequence of the matching side lengths of **A** and **B**, mixing of solutions of **1** and **2** led to the emergence of a third cage, trigonal prismatic **3**,^7a^ in equilibrium with the other two.

**Figure 1:**
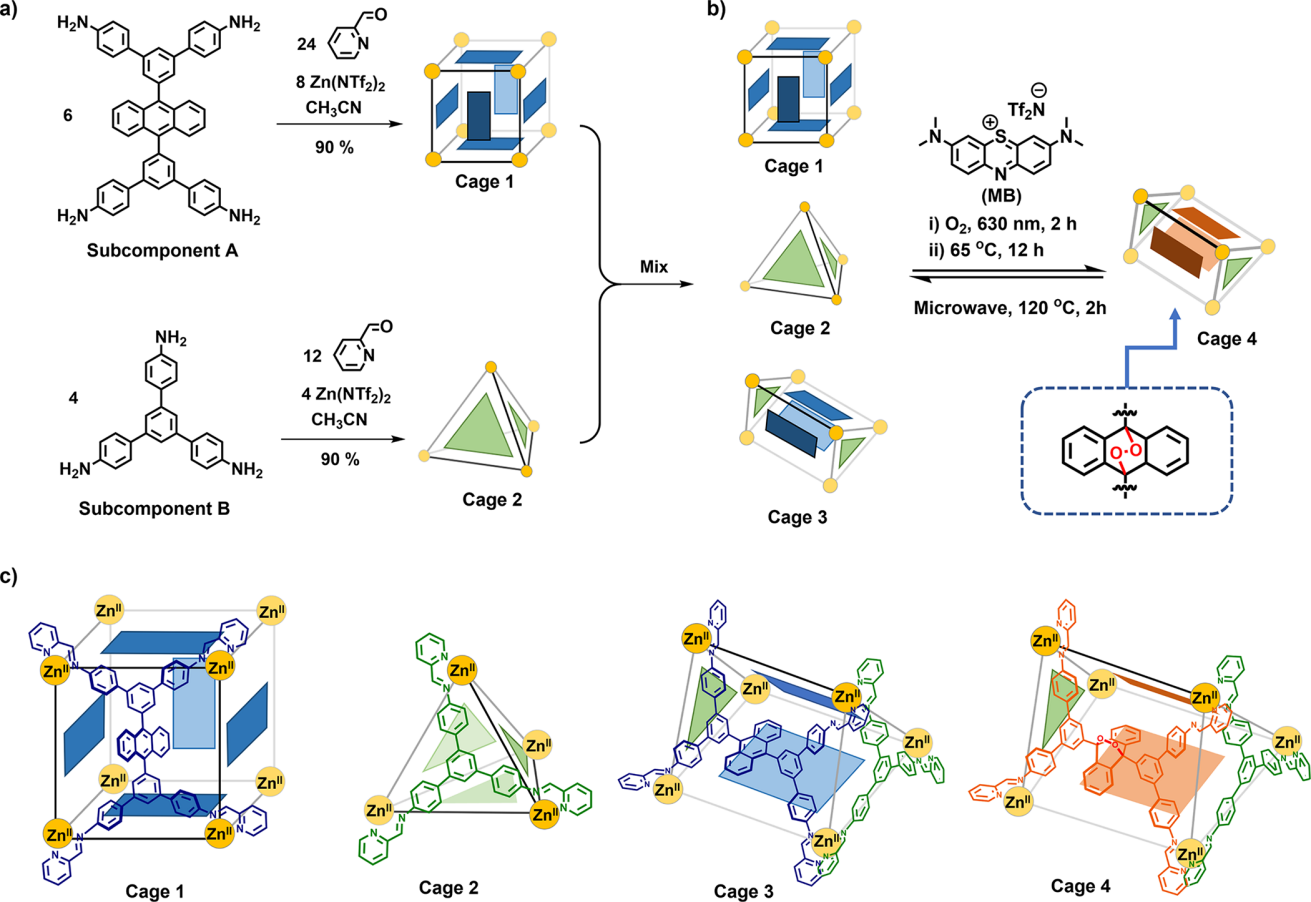
(a) Self-assembly and structural transformation of pseudocubic cage **1** and tetrahedral cage **2** from tetramine **A** and triamine **B**, respectively. (b) Construction of trigonal prismatic cage **3** and its transformation into cage **4** via hetero-Diels–Alder reaction with photogenerated ^1^O_2_. (c) Schematic illustrating how the ligands panel the faces of cages **1**–**4**.

Singlet oxygen (^1^O_2_) reacted with the anthracene-containing **B** residues within both **1** and **3** to generate the hetero-Diels–Alder endoperoxide product.^10^ This post-assembly modification^9^ impacted the relative stabilities of the members of the system by favoring the oxidized trigonal prismatic cage **4** and, thus, tilting the system toward integrative self-sorting.^1a,1b,6^ This modified trigonal prism **4** was observed to thermally revert to the precursor system following retro-Diels–Alder extrusion of O_2_ thermally, thus allowing for reversible switching between the mixed and integratively self-sorted states of the system.

The bulky anthracene panels of trigonal prismatic coordination cages **3** and **4** separated the internal cavity into two isolated compartments. Neutral guest molecules were encapsulated in the trigonal prisms **3** and **4**, but not in pseudocube **1** or only weakly in tetrahedron **2**.^11a^ Reaction with ^1^O_2_ thus set in motion a cascade of events that resulted in guest binding as the system of cages reconfigured.

In the absence of ^1^O_2_, the strong binding of adamantane within **3** also reconfigured the **1** + **2** ⇌ 2 × **3** equilibrium. This binding stabilized **3**, favoring its formation. Adamantane binding thus served as an alternative signal, which triggered the system to integratively self-sort.

Cages **1** and **2** were synthesized individually via subcomponent self-assembly, as shown in Figure 1a, where dynamic coordinative (N→Zn) and covalent (C=N) bonds formed during the same overall process. Subcomponent **A** was synthesized from commercially available anthracene-9,10-diboronic acid bis(pinacol) ester (Supporting Information, Section 2.1). The reaction of **A** (6 equiv) with zinc(II) bis(trifluoromethanesulfonyl)imide [Zn(NTf_2_)_2_, 8 equiv] and 2-formylpyridine (24 equiv) in acetonitrile at 70 °C produced Zn^II^_8_L_6_ cubic cage **1**. Electrospray ionization mass spectrometry (ESI-MS) confirmed the Zn^II^_8_L_6_ composition (Figure S11 and S12), in line with diffusion-ordered spectroscopy (DOSY) NMR measurements (Figure S10), which provided a hydrodynamic radius of 20.9 Å. The reaction of commercially available subcomponent **B** (4 equiv) with 2-formylpyridine (12 equiv) and Zn(NTf_2_)_2_ (4 equiv) provided cage **2**, following published procedures.^11^

Single crystals of cage **1** suitable for analysis by X-ray diffraction were obtained by the slow diffusion of diethyl ether into an acetonitrile solution. The solid-state structure (Figure 2a) revealed six anthracene ligands bridging eight octahedral Zn^II^ centers in an *S*_6_-symmetric framework, with four metal centers adopting a Λ configuration, and the other four adopting a Δ configuration.^12^ Within cage **1**, the metal–metal distances between adjacent vertices range from 14.8 to 16.4 Å. The bulky anthracene panels protrude into the cage cavity and leave only a small cavity volume of 9.0 Å^3^, as calculated using MoloVol^13^ (Figure S116).

**Figure 2:**
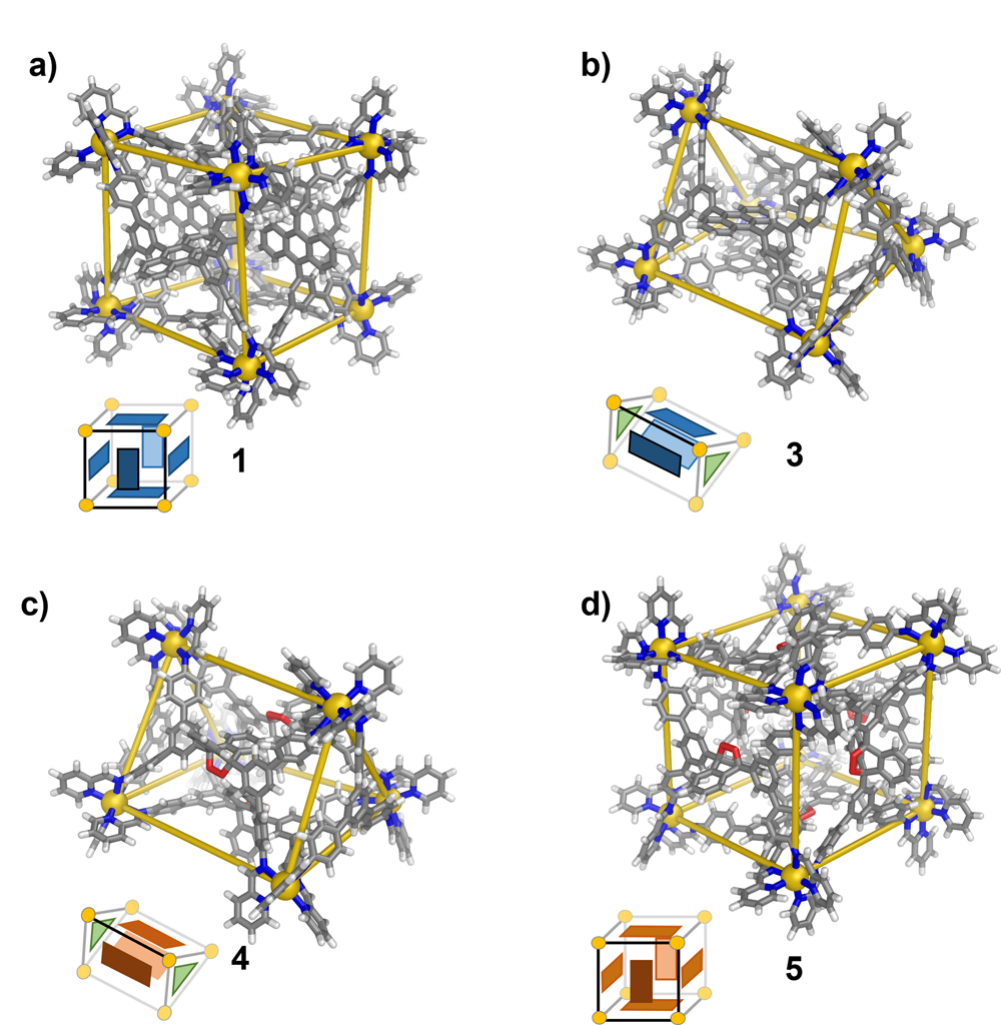
(a) X-ray structure of cage **1** (Zn, yellow; N, blue; O, red; C, gray; H, white). DFT-minimized structures of (b) trigonal prismatic cages **3** and (c) **4**. (d) DFT-minimized structure of pseudocubic **5**.

Cages **1** and **2** were mixed in acetonitrile and heated at 65 °C for 24 h. The formation of trigonal prismatic cage **3** was observed through integrative self-sorting^1b^ in equilibrium with the narcissistic products **1** and **2** (Figure 1). The presence of all three products was confirmed by ^1^H NMR, DOSY spectra (Figures S14, S15), and ESI-MS (Figures S16, S17). Two-dimensional NMR techniques provided structural information consistent with a *D*_3_-symmetric trigonal prismatic framework for cage **3** (Figures S54–S57). DFT calculations were undertaken using the Gaussian 16 program^14^ to obtain an energy-minimized structure for cage **3** shown in Figure 2c. This structure gave conformations of the three bulky anthracene panels that projected inward, which separated the internal cavity into two isolated compartments.

We then investigated the [4 + 2] hetero-Diels–Alder reaction between anthracene and ^1^O_2_ involving both anthracene-based cubic cage **1** and the self-sorted system containing cages **1**, **2**, and **3**. Pioneering work employing this reaction in supramolecular structures was conducted by Smith and co-workers.^15^ The groups of Stang,^16^ Shionoya,^17^ and Bibal^18^ have explored structural transformations of host organic molecular capsules and 2D metallo-macrocycles with changes of their binding affinities via this [4 + 2] hetero-Diels–Alder reaction. Building upon this work, cage **1** was mixed with the previously reported^17^ photosensitizer methylene blue (MB, 0.05 equiv) in acetonitrile. This solution was irradiated (λ_max_ = 630 nm) for 2 h at room temperature under air (Figure 1). After irradiation, the anthracene moieties of the cages were found to have reacted to form endoperoxides, thereby generating the oxidized cubic cage **5**, as shown in Figure 3b. ESI-MS and ^1^H NMR analyses confirmed a complete **1** → **5** transformation (Figures S28, S36, S37). Comparison of the ^1^H NMR spectra of cages **1** and **5** revealed the same number of signals but with different chemical shift values (Figures S3, S28), which implied that the *S*_6_ symmetry of the framework was maintained. The structure of cage **5** was further confirmed by 2D NMR spectroscopy (Figures S31–S35), and it was also minimized by DFT calculation (Figure 2b, see the Supporting Information, Section 7).

**Figure 3:**
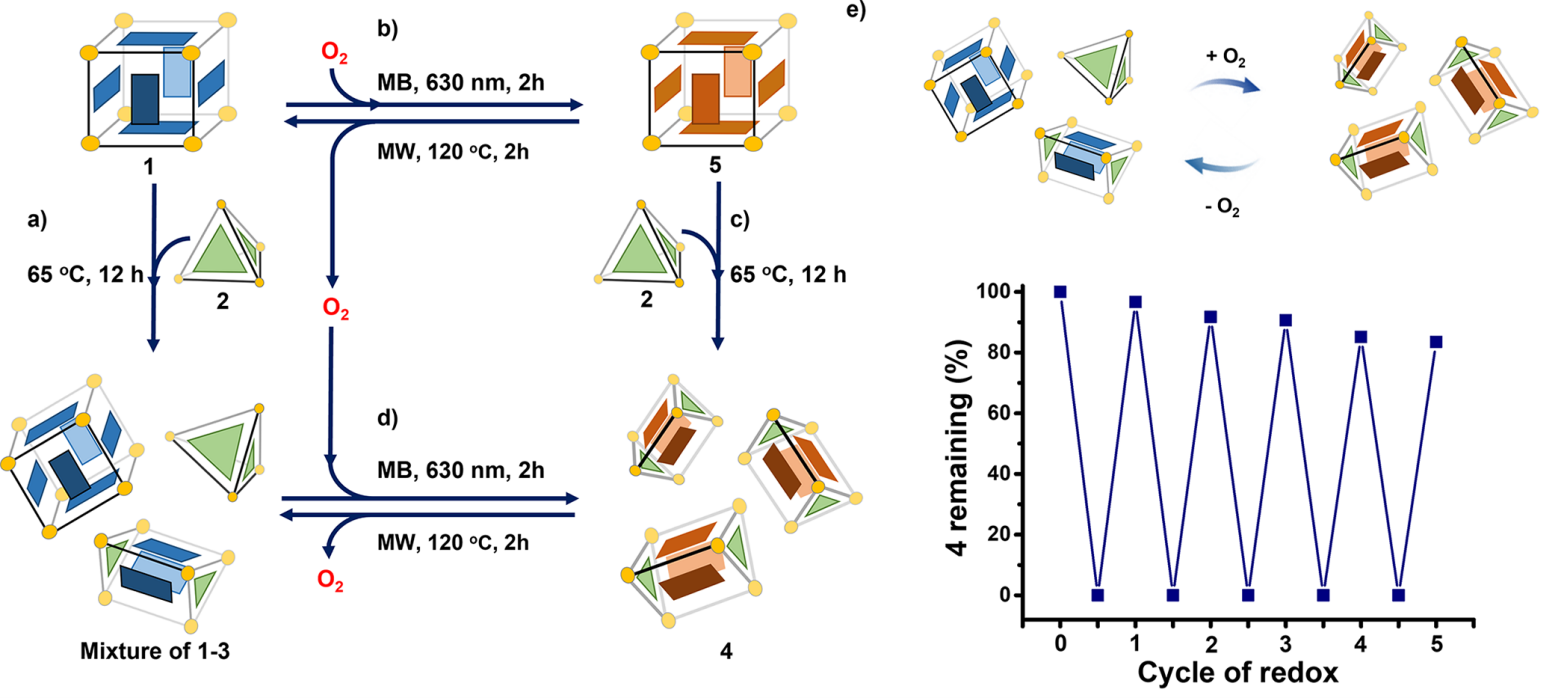
Interacting system of cages before and after reaction of **1** with ^1^O_2_. (a) The reaction between **1** and **2** led to an equilibrium mixture containing **1**, **2**, and **3**; (b) **1** reacted reversibly with in situ generated ^1^O_2_ to form **5**, (c) which in turn reacted with **2** to form only **4**; (d) treatment of the equilibrium mixture of **1**–**3** with ^1^O_2_ gave only **4**; both processes (b) and (d) were reversible following thermal retro-cycloaddition. (e) The reversibility of process (d) is charted over five cycles.

Next, we applied the same oxidation conditions to a mixture of cages **1**–**3**. All anthracene panels in this self-sorting system also underwent complete transformation into endoperoxides after irradiation. The hetero-Diels–Alder reaction triggered integrative self-sorting, thereby resulting in the exclusive formation of oxidized trigonal prismatic cage **4**, the formulation of which was confirmed by ^1^H NMR and ESI-MS (Figures S18, S26, S27). DFT geometry minimization provided a structure for oxidized trigonal prismatic cage **4** (Figure 2c) that was again consistent with 2D NMR spectra (Figures S21–25), which reflected the *C*_3_ point symmetry.

We also attempted to construct oxidized cages **4** and **5** by first treating subcomponent **A** with ^1^O_2_. However, the oxidized subcomponent **A** not only took a much longer time (10 h) under irradiation for oxidation to approach completion but also underwent thermal decomposition during cage assembly (Figures S38, S39). The endoperoxide moiety thus appears to be more stable when incorporated into a coordination cage.

As the cycloaddition reaction between ^1^O_2_ and anthracene is thermally reversible,^16,18^ we studied the recovery of parent cage **1** from oxidized **5** (Figure S40). This transformation occurred after heating **5** in acetonitrile above its atmospheric boiling point (caution!) at 120 °C under microwave irradiation for 2 h. Trigonal prismatic cage **4** also underwent deoxygenative retro-cycloaddition to transform back into the initial mixture of **1**–**3** following microwave heating. After five cycles of photooxygenation/cycloreversion, NMR integration indicated that 84% of the oxidized trigonal prism was formed relative to the amount initially present (Figures 3e, S42). We infer that the high temperature (120 °C) reached during the microwave reaction may result in partial product decomposition.

Oxidized trigonal prismatic cage **4** also assembled directly from a mixture of tetrahedral cage **2** and oxidized cubic cage **5** (Figures 3c, S41). We infer that the addition of ^1^O_2_ stabilized oxidized cage **4** relative to **3**, which rendered the **2** + **5** → 2 × **4** transformation more thermodynamically favorable than the corresponding **1** + **2** → 2 × **3** process.

Variable temperature ^1^H NMR measurements were used to construct a van’t Hoff plot from which thermodynamic parameters were obtained^19^ for the **1** + **2** ⇌ 2 × **3** equilibrium (Figure S43). Conversion into **3** was an endothermic and entropically favored process, with Δ*H* = 48.35 ± 1.61 kJ mol^–1^ and Δ*S* = 150.6 ± 4.7 J K^–1^ mol^–1^ (Figure S44).

By contrast, the **2** + **5** → 2 × **4** transformation (Figure 3d) was found not to be reversible between 25 and 65 °C, which was consistent with the high thermodynamic stability of **4** relative to **2** and **5** (Figure S45). The relative energetic favorability of structure **4** was also supported by DFT calculations (Figures S113, S114). DFT energy-minimized structures also suggested that the bent anthracene endoperoxide moieties reduced hindrance inside the cage to further stabilize cage **4** (Figure S112).

The host–guest properties of trigonal prismatic cages **3** and **4** were then investigated (Figure 4). Notably, empty cage **4** was obtained directly through post-assembly modification with ^1^O_2_, whereas empty cage **3** could not be purified because of its equilibration with cages **1** and **2**. In cages **3** and **4**, the bulky anthracene units were designed to separate the internal cavity into two isolated compartments, which resembles a “peanut” structure.^20^ The internal cavity volumes were calculated by MoloVol^13^ to be 309 and 279 Å^3^ for **3** and 259 and 226 Å^3^ for **4**, respectively (Figures 4a, S115). Cages **3** and **4** both bound a series of alkanes, including adamantane (**G1**) and norbornane (**G2**) in slow exchange on the NMR time scale (Figures S48, S58, S75, S81). Encapsulation was further confirmed by DOSY NMR (Figures S51, S59, S76, S82), where the encapsulated guest and host were observed to diffuse at the same rate. Intriguingly, some terpenoid natural products, such as (1*S*)-(−)-camphor (**G3**), verbenone (**G4**), and (−)-beta pinene (**G5**), which are similar in size to norbornane, were also observed to encapsulate within both **3** and **4** (Figures 4b, S63, S67, S70, S87, S93, S99) and also in slow exchange on the NMR time scale. These host–guest interactions were additionally confirmed by NOESY NMR, ESI-MS analysis, and isothermal titration calorimetry (ITC) (Figures S52–S104).

**Figure 4:**
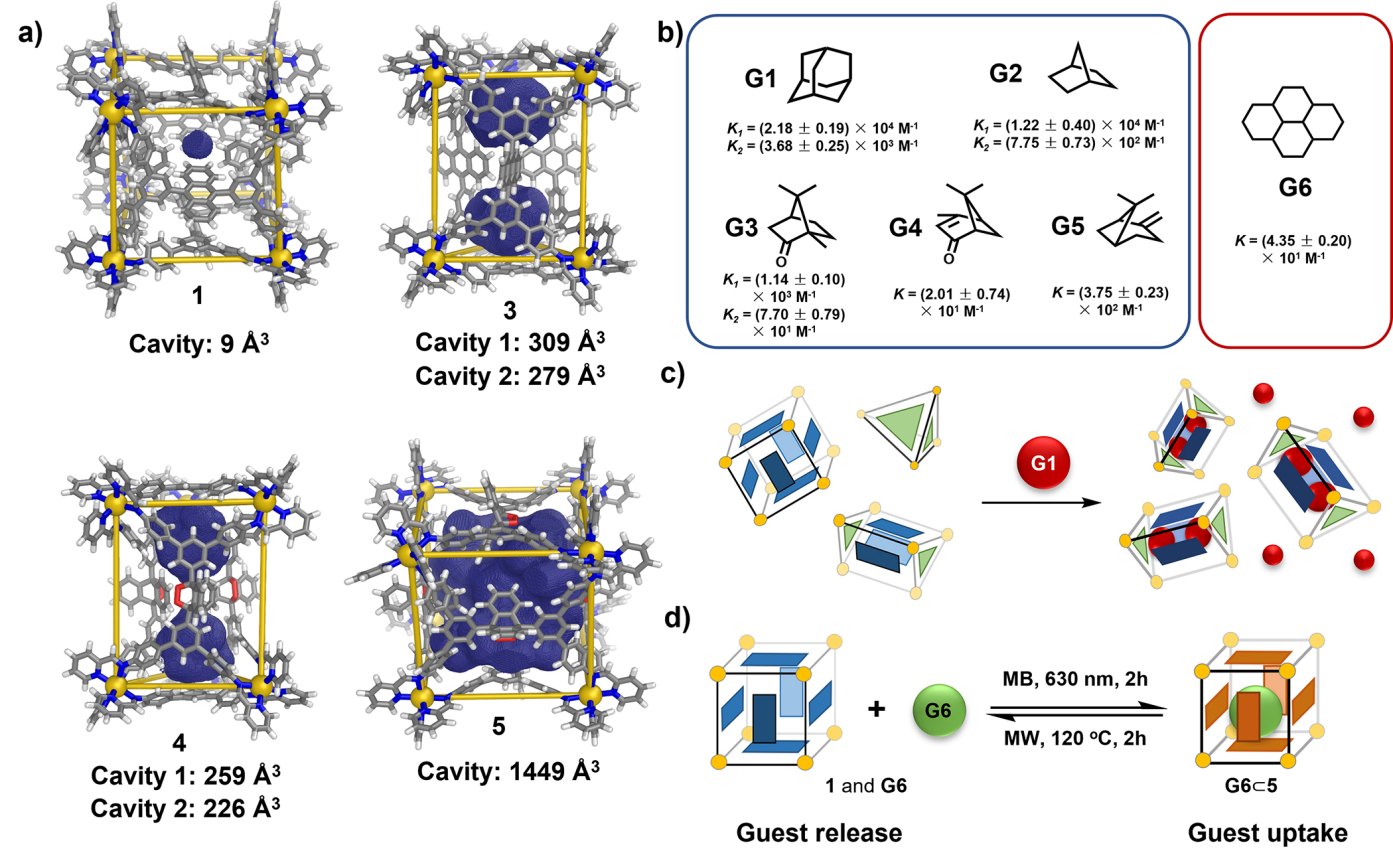
(a) Cavity volumes of **1**, **3**, **4**, and **5**, outlined in deep blue mesh. (b) Guest molecules **G1**–**G5** were encapsulated by both **3** and **4** (binding constants of guests **G1**–**G5** with **4** are given below), whereas **G6** was encapsulated only by **5** (binding constants of **G6**⊂**5** are given below). (c) **G1** induced conversion from **1** and **2** to form exclusively **G1**⊂**3**. (d) **G6** was taken up and released as a result of the reversible ^1^O_2_-mediated transformation of **1** to **5**.

Host–guest binding between **G1**–**G5** with cages **3** and **4** is relatively weak. The binding constants between guests **G1**–**G5** and **4** were determined through ITC. The titrations of guests **G1–G3** with **4** were fitted using a two-sequential-binding sites model in keeping with the two internal cavities of **4**. The binding constants were calculated to be *K*_*1*_ = (2.18 ± 0.19) × 10^4^ M^–1^ and *K*_*2*_ = (3.68 ± 0.25) × 10^3^ M^–1^ (2·**G1**⊂**4**); *K*_*1*_ = (1.22 ± 0.40) × 10^4^ M^–1^ and *K*_*2*_ = (7.75 ± 0.73) × 10^2^ M^–1^ (2·**G2**⊂**4**); and *K*_*1*_ = (1.14 ± 0.10) × 10^3^ M^–1^ and *K*_*2*_ = (7.70 ± 0.79) × 10^1^ M^–1^ (2·**G3**⊂**4**). Because of the weaker binding between guests **G4** and **G5** with **4**, only a single binding event could be observed in ITC. The binding constants of **G4**⊂**4** and **G5**⊂**4** were calculated to be (2.01 ± 0.74) × 10^1^ M^–1^ and (3.75 ± 0.23) × 10^2^ M^–1^, respectively. These single-guest bindings of **G4**⊂**4** and **G5**⊂**4** were also supported by ESI-MS analysis, as shown in Figures S95 and S101.

Synthetic receptors have been shown to adjust their binding sites to better bind guests.^8b^ Thus, we also studied guest-encapsulation-induced structural transformation in the equilibrium mixture of cages **1**, **2**, and **3**. The addition of **G1** to this mixture prompted re-equilibration, which resulted in the formation of only cage **3** containing **G1** (Figure 4c). ^1^H NMR integrations indicated the formation of the host–guest complex 2·**G1**⊂**3**, which was also confirmed by ESI-MS, with no signals observed corresponding to cages **1** and **2**. The host–guest complex 2·**G1**⊂**3** was characterized by 2D NMR spectroscopy (Figures S55–S57).

Entropy changes associated with guest encapsulation may help drive the reconfiguration of the system. The freeing of solvent from the cavity of a cage provides an entropic driving force for guest binding. The entropy change associated with guest binding within **3** could, thus, result in the stabilization of heteroleptic **3** as opposed to homoleptic **2** and **1**, which bind guest **G1** at best weakly (for **2**).^11a^

The host–guest properties of pseudo-cubic cages **1** and **5** were also investigated. In **1**, the bulky anthracene moieties were oriented toward the center of the structure, thereby effectively occupying the internal cavity volume, which was calculated to be only 9 Å^3^ using MoloVol^13^ (Figure 4a), precluding guest encapsulation. However, cycloaddition between ^1^O_2_ and the anthracene panels in cage **5** brought about a substantial expansion of the internal cavity volume to 1449 Å^3^. Consequently, the guest hexadecahydropyrene (**G6**) was complexed by **5** with a low binding constant of 43.5 ± 2.01 M^–1^, whereas no binding of this guest by **1** was observed (Figures S105, S107–S110). The reversible hetero-Diels–Alder reaction could, thus, be used to regulate **G6** uptake and release, as shown in Figures 4d and S111.

The use of the reversible cycloaddition of ^1^O_2_ to anthracene to reconfigure a self-sorting system may, thus, open new possibilities for signal transduction within systems of cages involving guest uptake and release. The incorporation of enantiopure anthracene ligands may also enable the dynamic control of the chirotopic internal cavities of these coordination cages for potential applications of enantioselective guest recognition and separation.^21^

## Supporting Information Available

Experimental procedures, NMR characterizations, mass spectrometry data, volume calculations, DFT calculations, and X-ray crystallographic data (PDF)
