# Supplementary material for: Hetero-Diels–Alder Reaction between Singlet Oxygen and Anthracene Drives Integrative Cage Self-Sorting
Source: J Am Chem Soc. 2023 Aug 23;145(35):19164–70. doi: 10.1021/jacs.3c04228 (PMC10485901; doi:10.1021/jacs.3c04228)
Supplement: Supplementary file 1 — ja3c04228_si_001.pdf [file ja3c04228_si_001.pdf]

Supporting Information for

Hetero-Diels-Alder Reaction between Singlet Oxygen and Anthracene Drives Integrative Cage Self-Sorting

Yuchong Yang,† Tanya K. Ronson,† Dingyu Hou,‡ Jieyu Zheng,† Ilma Jahović,† Kai Hong Luo,‡ Jonathan R. Nitschke†,*

† Yusuf Hamied Department of Chemistry, University of Cambridge, Cambridge CB2 1EW, United Kingdom.

‡ Department of Mechanical Engineering, University College London, London, WC1E 7JE, United Kingdom.

* To whom correspondence should be addressed: jrn34@cam.ac.uk

1. General Information 3

2. Synthesis and characterization of subcomponents 4

2.1 Synthesis of subcomponent A..........4

3. Characterization of coordination cages 6

3.2 Construction of tetrahedron cage 2..........13

3.3 Formation of a mixture of cages 1, 2 and 3..........15

3.4 Characterization of oxidized trigonal prism cage 4 18

3.5 Characterization of oxidized cubic cage 5..........24

3.6 Oxidation of Subcomponent A before cage assembly 30

4 Structural transfer among Cage 1-5 32

4.1 Structural transformations in Figure 3b 32

4.2 Structural transformations in Figure 3c 32

4.3 Structural transformations in Figure 3d and Figure 3e..........33

5 Variable temperature ^{1}H NMR for energy calculation 34

5.1 General procedures 34

5.2 Variable temperature ^{1}H NMR of cage 1-3 mixture..........34

5.3 Variable temperature ^{1}H NMR of cage 4..........36

6 Host-guest studies 37

6.1 General procedures 37

6.2 Host-guest interactions of cage mixture with G1-G6....................................37

6.2.1 Host-guest interactions of cage mixture with adamantane (G1) 39

6.2.2 Host-guest interactions of cage mixture with norbornane (G2).........................47

6.2.3 Host-guest interactions of cage mixture with (1*S*)-(-)-camphor (G3) 51

6.2.4 Host-guest interactions of cage mixture with verbenone (G4) 55

6.2.5 Host-guest interactions of cage mixture with (-)-beta pinene (G5)....................58

6.3 Host-guest interactions of cage 4 with G1-G6 61

6.3.1 Host-guest interactions of cage 4 with adamantane (G1)................................62

6.3.2 Host-guest interactions of cage 4 with norbornane (G2)..................................67

6.3.3 Host-guest interactions of cage 4 with (1*S*)-(-)-camphor (G3) 72

6.3.4 Host-guest interactions of cage 4 with verbenone (G4)...................................77

6.3.5 Host-guest interactions of cage 4 with (-)-beta pinene (G5) 82

6.4 Host-guest interactions of cage 5..87

6.4.1 Host-guest interactions of cage 5 with hexadecahydropyrene (G6) 87

6.4.2 Cage 1 mixed with G6..88

6.4.3 DOSY of G6⊂cage 5 and free G6..89

6.4.4 ESI-MS of G6⊂cage 5 89

6.4.5 ITC titration of guest G6 to cage 5 91

6.4.6 Uptake and release of G6 92

7 DFT calculations 93

7.1 General procedure 93

7.2 Structural optimization of cage 1-5...93

7.3 Calculated energy of cage 1-5 143

7.4 Energy calculations of structural transformation between cages 143

7.5 Internal cavity volume of cage 1, 3 and 4...145

8 X-ray crystallography 147

9 References 149

1. General Information

All experiments were carried out under inert atmosphere of purified nitrogen or using standard Schlenk techniques. Reactions were stirred using Teflon-coated magnetic stir bars. Elevated temperatures were maintained using thermostat-controlled silicone oil baths. Organic solutions were concentrated using a rotary evaporator with a diaphragm vacuum pump. Analytical TLC was performed on Merck silica gel 60 F_{254} plates. The TLC plates were visualized by either ultraviolet light. Purification of products was accomplished by flash column chromatography on silica gel 60 (Merck, particle diameter 40-63 μm). Unless otherwise specified, all reagents were purchased from commercial sources and used as received.

NMR spectra were recorded at room temperature, unless stated otherwise, on either a Bruker 400 MHz Avance III HD Smart Probe or Bruker 500 MHz Avance III HD Smart Probe (^{1}H, ^{13}C and 2D experiments). Data for ^{1}H NMR spectra were reported as follows: chemical shift (ppm), peak shape (s = singlet, d = doublet, t = triplet, m = multiplet), coupling constant (Hz), and integration. Data for ^{13}C NMR were reported in terms of chemical shift (ppm). DOSY experiments were performed on a Bruker 400 MHz Avance III HD Smart Probe.

A microwave reactor from Discover SP-D 80-CEM Corporation was used for the stereochemical induction experiments. Centrifugation of samples was carried out using a Grant-Bio LMC-3000 low speed benchtop centrifuge. Low-resolution electrospray ionization mass spectra (ESI-MS) were obtained on Waters XevoTQD (cone voltage 5-20eV; desolvation temperature 307K; ionization temperature 325K) or Micromass Quattro LC mass spectrometer (cone voltage 5-20eV; desolvation temperature 313K; ionization temperature 313K), both infused from a Harvard Syringe Pump at a rate of 10 μL per minute. High-resolution ESI mass spectra were obtained with Waters Synapt G2-Si instrument.

Isothermal Titration Calorimetry (ITC). Titration experiments were carried out in dry MeCN at 298 K on a MicroCal iTC200. To set up an experimental run, a 200 μL solution of the cage was transferred to the sample cell and 40 μL of the solution of a guest molecule in dry MeCN was placed in the syringe. Exact concentrations used are specified underneath each thermogram. The first injection of 0.5 μL was followed by another 19 injections of 2 μL each, with around 300 s spacing between the injections. The solution was stirred at 750 rpm. Heats of dilution were determined in identical experiments, but without cage in the cell and were subtracted from each data set. We titrated a solution of each cage with MeCN and observed negligible heats of dilution evolved. The data were processed using the Origin software package with the ITC plugin.

2. Synthesis and characterization of subcomponents

2.1 Synthesis of subcomponent A

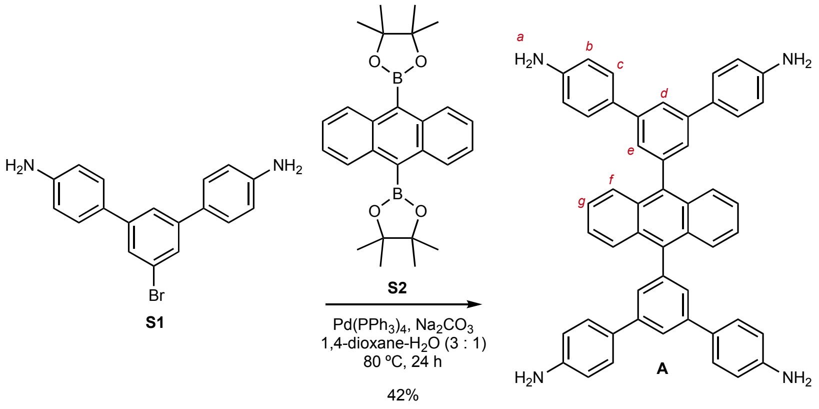

Scheme 1 Synthesis of subcomponent **A**

S1 was synthesized according to reported procedures.[1]

Subcomponent **A**: **S1** (376 mg, 1.11 mmol, 4.7 eq.), Na_2CO_3 (54 mg, 0.51 mmol, 2.2 eq.) and $Pd(PPh_3)_4$ (28 mg, 24 µmol, 10 mol%) were added to a solution of **S2** (100 mg, 0.232 mmol, 1.0 eq.) in degassed 1,4-dioxane/water (16 mL, v/v = 3:1). The reaction mixture was stirred at 80 °C for 24 hours. After cooling to room temperature, the solvent was removed *in vacuo*, and the crude product was dissolved in dichloromethane (50 mL) and water (50 mL). The organic phase was washed with brine (50 mL), dried over Na_2SO_4, filtered, and concentrated *in vacuo*. The crude product was purified by flash column chromatography (SiO_2, EtOAc / *n*-hexane = 50→75%) to afford (5',5''''-(anthracene-9,10-diyl)bis(([1,1':3',1''-terphenyl]-4,4''-diamine)) (**A**) as a pale yellow solid (67.85 mg, 97.6 µmol, 42% yield).

^{1}H NMR (500 MHz, d_6-DMSO, 298 K) δ (ppm) = 7.91 (s, 2H, $\boldsymbol{H}_d$), 7.80-7.78 (d, J = 8.5 Hz, 4H, $\boldsymbol{H}_f$), 7.54 (d, J = 8.5 Hz, 8H, $\boldsymbol{H}_c$), 7.46-7.44 (m, 8H, $\boldsymbol{H}_g$, $\boldsymbol{H}_e$), 6.66 (d, J = 8.5 Hz, 8H, $\boldsymbol{H}_b$), 5.26 (s, 8H, $\boldsymbol{H}_a$).

^{13}C NMR (126 MHz, d_6-DMSO, 298 K) δ (ppm) = 149.0, 141.8, 139.4, 137.3, 129.7, 127.9, 127.5, 127.0, 125.9, 125.7, 122.0, 114.6.

ESI-HRMS Found m/z = 694.3076 $[M]^+$, $C_{50}H_{38}N_4$ requires m/z =694.3096.

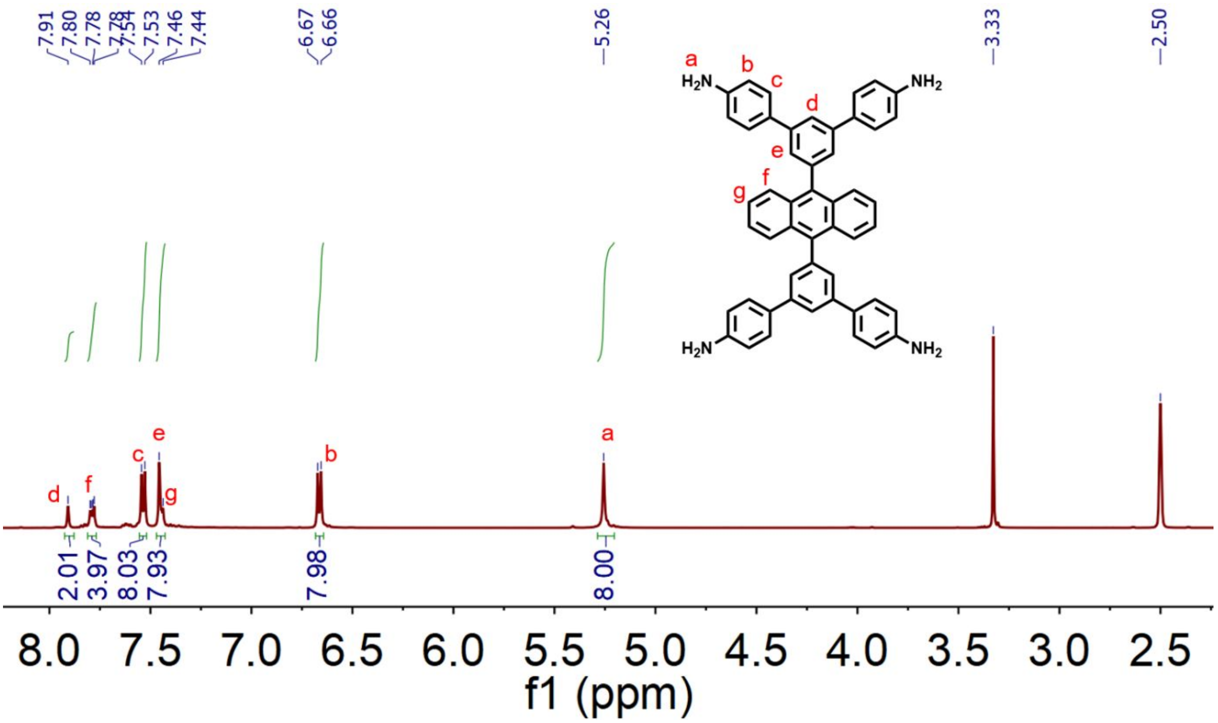

Figure S1. ^{1}H NMR spectrum of **A** (500 MHz, d_6-DMSO, 298 K).

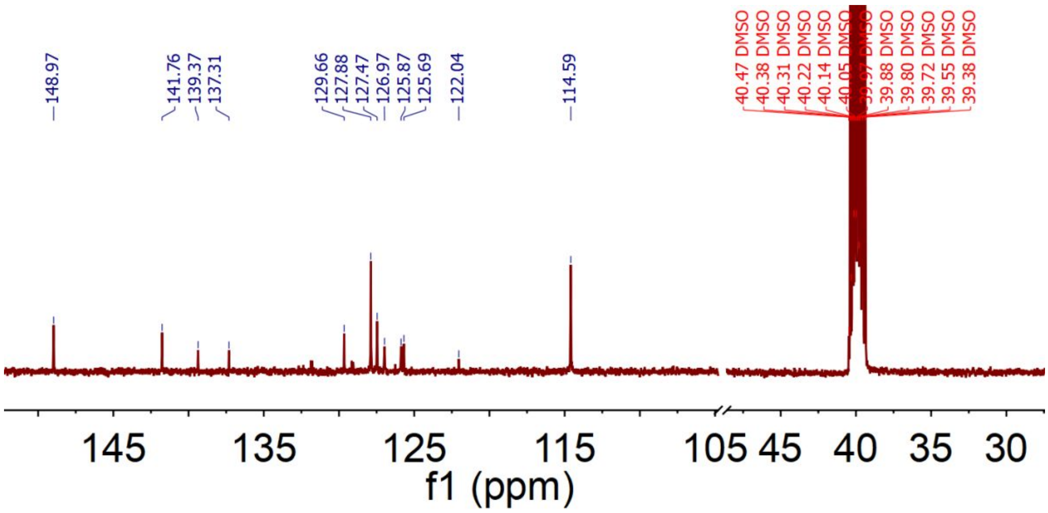

Figure S2. ^{13}C NMR spectrum of **A** (101 MHz, d_6-DMSO, 298 K).

3. Characterization of coordination cages

3.1 Characterization of cubic coordination cage 1

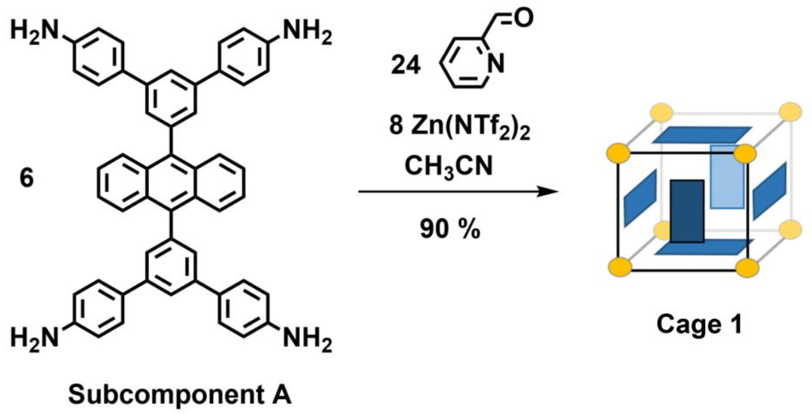

Scheme S2: Subcomponent self-assembly of cage **1**.

A (1.0 mg, 1.4 μmol, 1.0 equiv) was added to CD_3CN (0.6 mL) together with $Zn(NTf_2)_2$ (0.63 mg, 1.9 μmol, 1.4 equiv) and 2-formylpyridine (0.33 mg, 5.8 μmol, 4.1 equiv). The reaction mixture was stirred at 70 °C for 12 h or microwave 120 °C for 2 h. After cooling to room temperature, the solvent was evaporated and diethyl ether (14 ml) was then added. The residue was resuspended and then centrifuged and the diethyl ether decanted. This procedure was repeated three times with fresh diethyl ether. The residue was then dried *in vacuo* to afford the desired product (cage **1**) as a light yellow solid (2.5 mg, 90% yield).

Characterization of cage **1:**

^{1}H NMR (500 MHz, CD_3CN, 298 K) δ (ppm) = 8.87 (s, 6H), 8.76 (s, 6H), 8.55-8.52 (m, 18H), 8.44-8.23 (m, 18H), 8.19 (d, *J* = 5.0 Hz, 6H), 8.06 (d, *J* = 5.0 Hz, 6H), 7.99 (ddd, *J* = 8.0, 5.0, 1.2 Hz, 6H), 7.93 (t, *J* = 1.7 Hz, 6H), 7.85-7.68 (m, 48H), 7.66 (d, *J* = 5.0 Hz, 6H), 7.62-7.33 (m, 90H), 7.23 (t, *J* = 1.6 Hz, 36H), 7.19 (d, *J* = 8.5 Hz, 12H), 6.95 (d, *J* = 8.5 Hz, 12H), 6.68 (d, *J* = 8.5 Hz, 12H), 5.82 (d, *J* = 8.5 Hz, 12H).

^{13}C NMR (126 MHz, CD_3CN, 298 K) δ (ppm) = 146.36, 146.29, 146.28, 146.16, 146.10, 146.09, 145.88, 142.81, 142.46, 142.33, 141.99, 141.76, 141.34, 141.26, 140.84, 140.50, 140.49, 140.23, 139.20, 136.63, 136.58, 131.29, 131.06, 130.90, 130.68, 130.31, 130.21, 129.73, 129.62, 129.48, 129.42, 128.90, 128.82, 128.73, 128.17, 127.56, 127.36, 126.86, 126.73, 126.57, 126.43, 125.85, 125.85, 125.78, 125.32, 124.89, 124.72, 123.67, 123.14, 123.10, 122.47, 121.12.

ESI-MS: *m/z* = 662.5 [**1**$(NTf_2^-)_4]^{12+}$, 748.2 [**1**$(NTf_2^-)_5]^{11+}$, 850.9 [**1**$(NTf_2^-)_6]^{10+}$, 976.8 [**1**$(NTf_2^-)_7]^{9+}$, 1134.0 [**1**$(NTf_2^-)_8]^{8+}$, 1335.9 [**1**$(NTf_2^-)_9]^{7+}$,1605.4 [**1**$(NTf_2^-)_{10}]^{6+}$, 1982.2 [**1**$(NTf_2^-)_{11}]^{5+}$. Calculated results: m/z = 662.6 [**1**$(NTf_2^-)_4]^{12+}$, 748.3 [**1**$(NTf_2^-)_5]^{11+}$, 851.2 [**1**$(NTf_2^-)_6]^{10+}$, 976.9 [**1**$(NTf_2^-)_7]^{9+}$, 1134.0 [**1**$(NTf_2^-)_8]^{8+}$, 1336.0 [**1**$(NTf_2^-)_9]^{7+}$,1605.4 [**1**$(NTf_2^-)_{10}]^{6+}$, 1982.5 [**1**$(NTf_2^-)_{11}]^{5+}$.

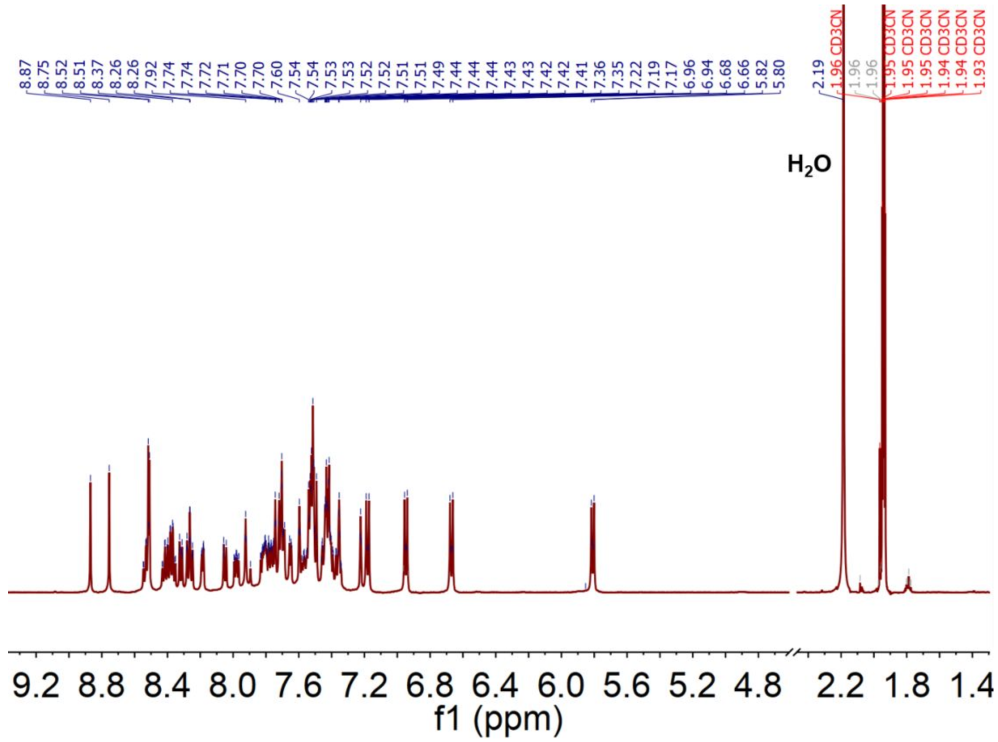

Figure S3. ^{1}H NMR spectrum of cage **1** (500 MHz, CD_3CN, 298 K).

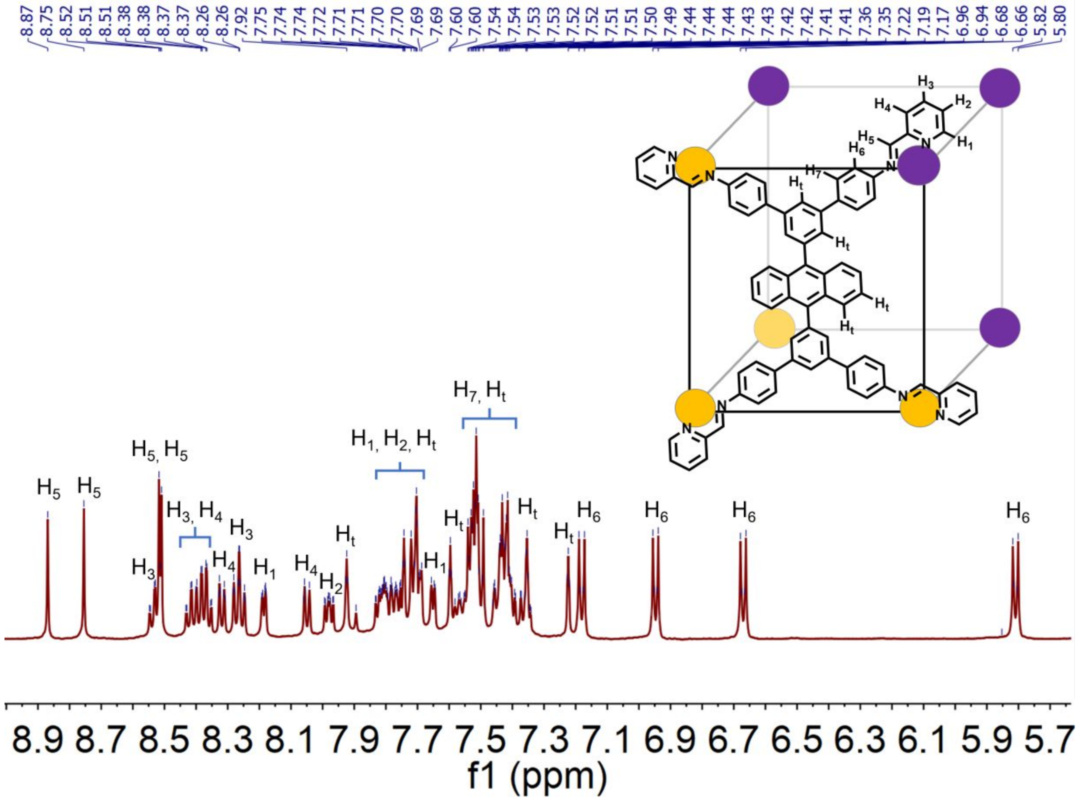

Figure S4. Partial ^{1}H NMR spectrum of cage **1** (500 MHz, CD_3CN, 298 K). The observed four imine peaks indicated the S_6-symmetric cubic configurations with four stereogenic metal centers adopting a *Λ* configuration, the other four metal centers adopting a *Δ* configuration.

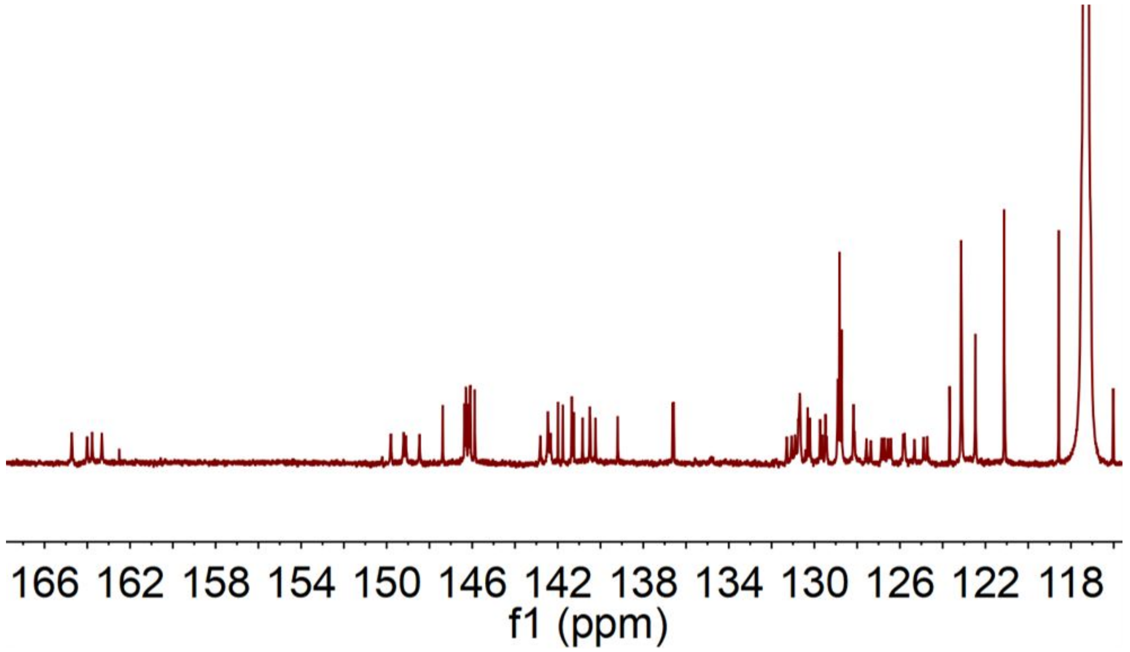

Figure S5. Enlarged region of the ^{13}C NMR spectrum of cage **1** (126 MHz, CD_3CN, 298 K).

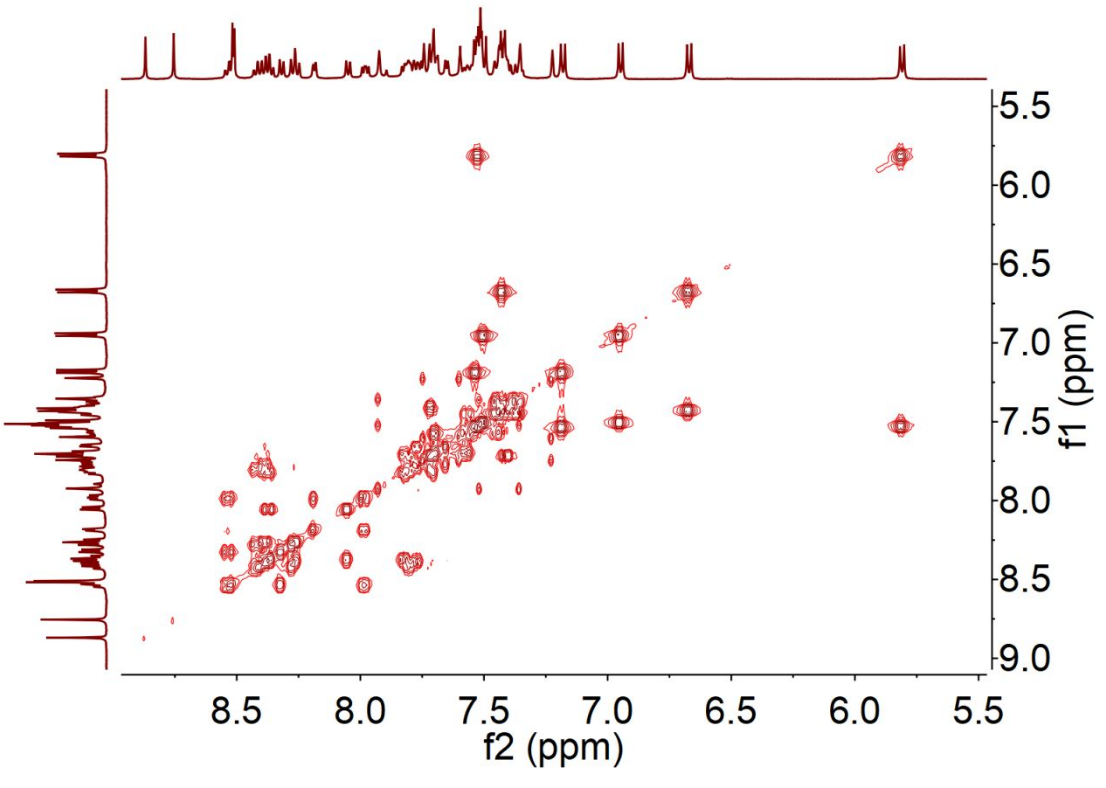

Figure S6. Partial ^{1}H-^{1}H COSY NMR spectrum of **1** (500 MHz, CD_3CN, 298 K).

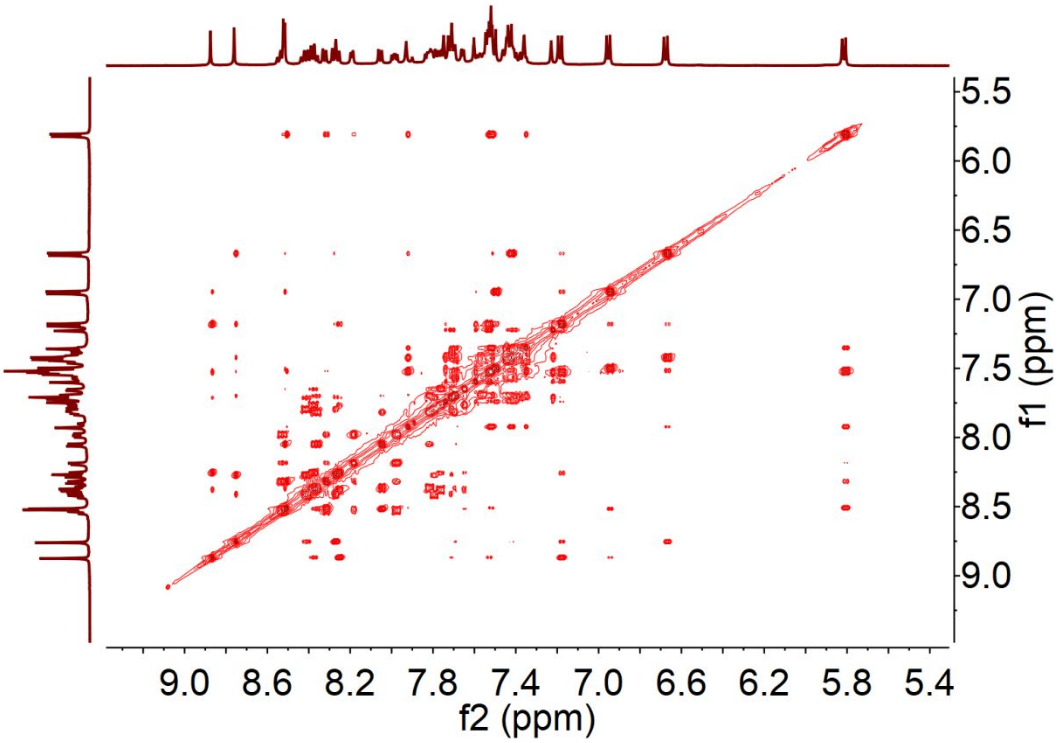

Figure S7. Aromatic region of the ^1H-^1H NOESY NMR spectrum of cage **1** (500 MHz, CD_3CN, 298 K).

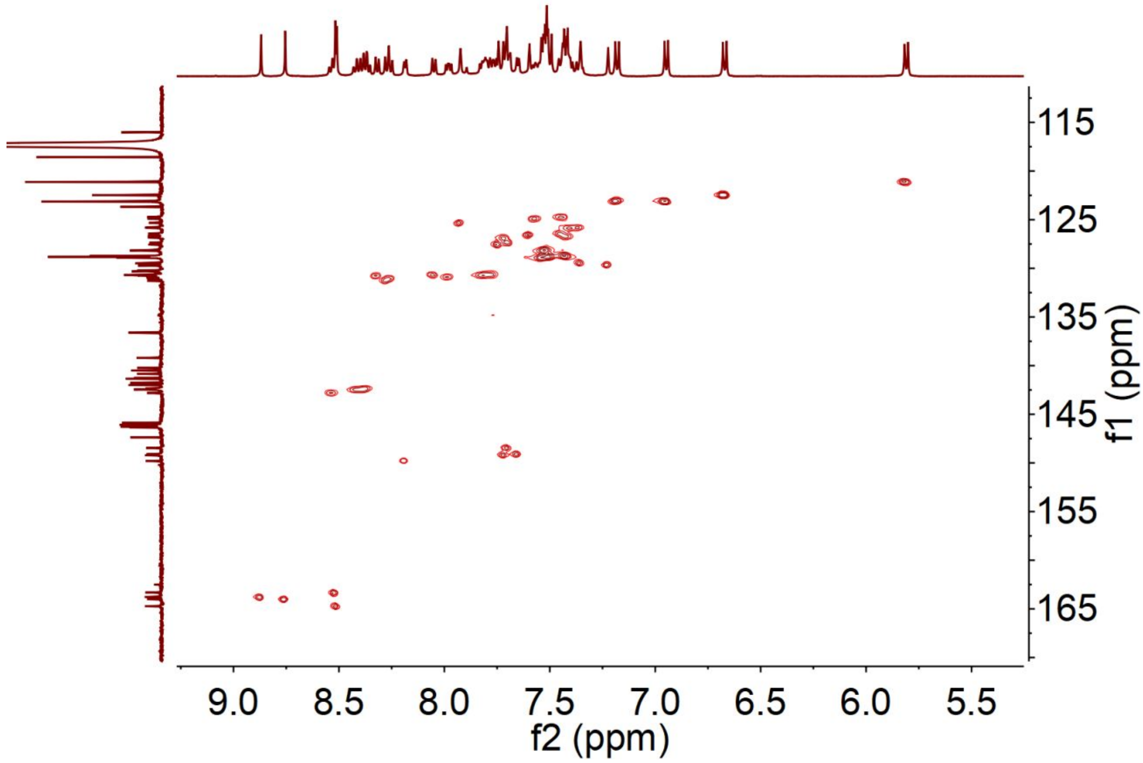

Figure S8. Aromatic region of the ^1H-^{13}C HSQC NMR spectrum of cage **1** (500 MHz, CD_3CN, 298 K).

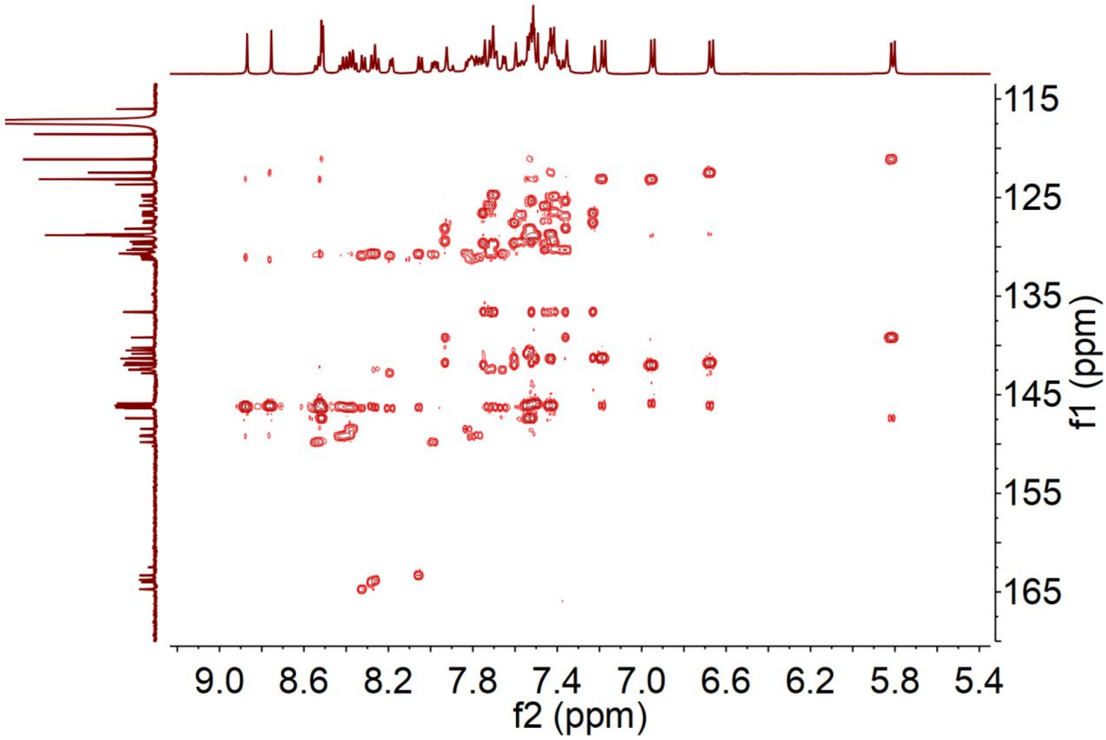

Figure S9. Aromatic region of the ^{1}H-^{13}C HMBC NMR spectrum of cage **1** (500 MHz, CD_3CN, 298 K).

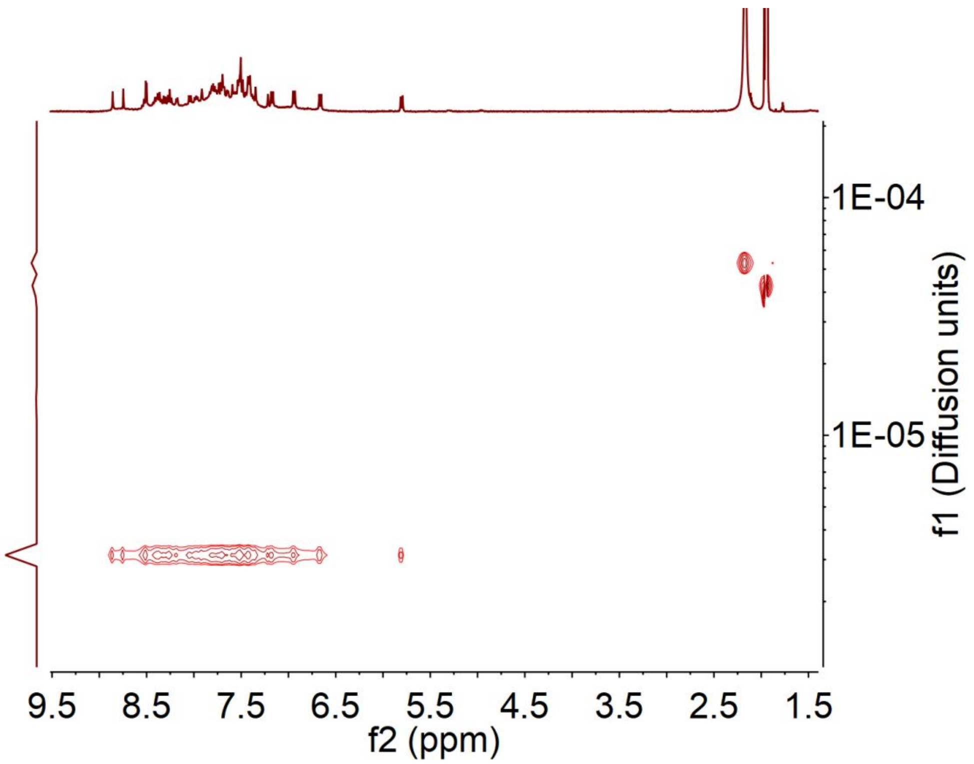

Figure S10. ^{1}H DOSY NMR spectrum of cage **1** (400 MHz, CD_3CN, 298 K). The diffusion coefficient for **1** in CD_3CN was measured to be 3.13×10^{-6} cm^2s^{-1}, corresponding to a hydrodynamic radius of 20.9 Å.

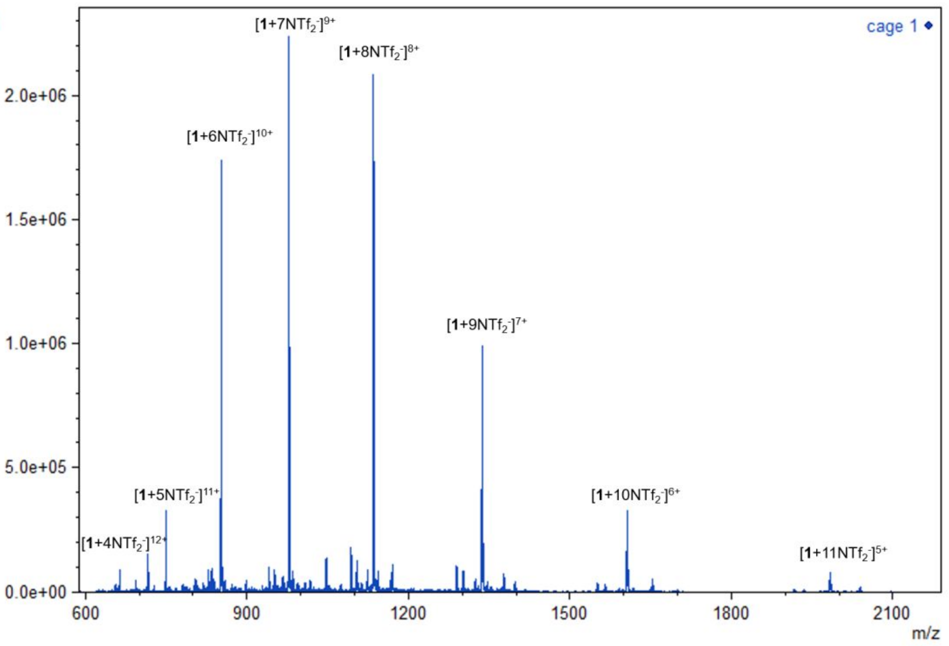

Figure S11: Low-resolution ESI-mass spectrum of **1**$(NTf_2^-)_{16}$. ESI-MS: Experimental results: *m*/*z* = 662.5 [**1**$(NTf_2^-)_4]^{12+}$, 748.2 [**1**$(NTf_2^-)_5]^{11+}$, 850.9 [**1**$(NTf_2^-)_6]^{10+}$, 976.8 [**1**$(NTf_2^-)_7]^{9+}$, 1134.0 [**1**$(NTf_2^-)_8]^{8+}$, 1335.9 [**1**$(NTf_2^-)_9]^{7+}$,1605.4 [**1**$(NTf_2^-)_{10}]^{6+}$, 1982.2 [**1**$(NTf_2^-)_{11}]^{5+}$.

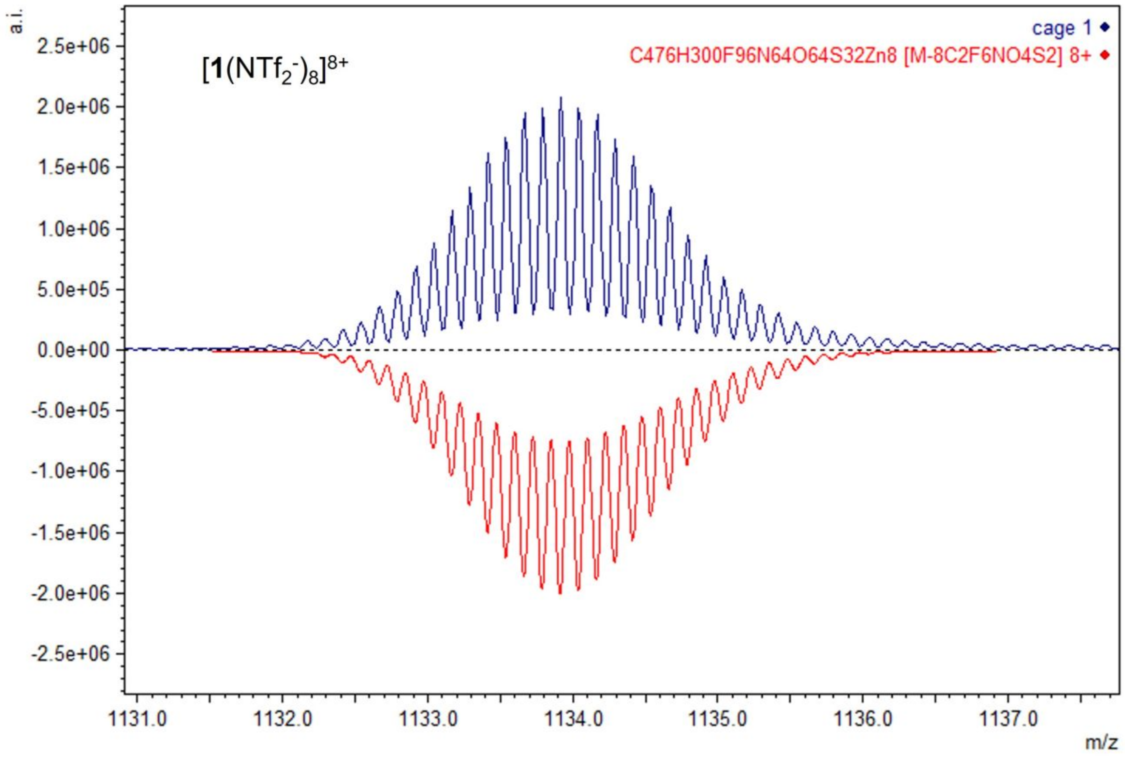

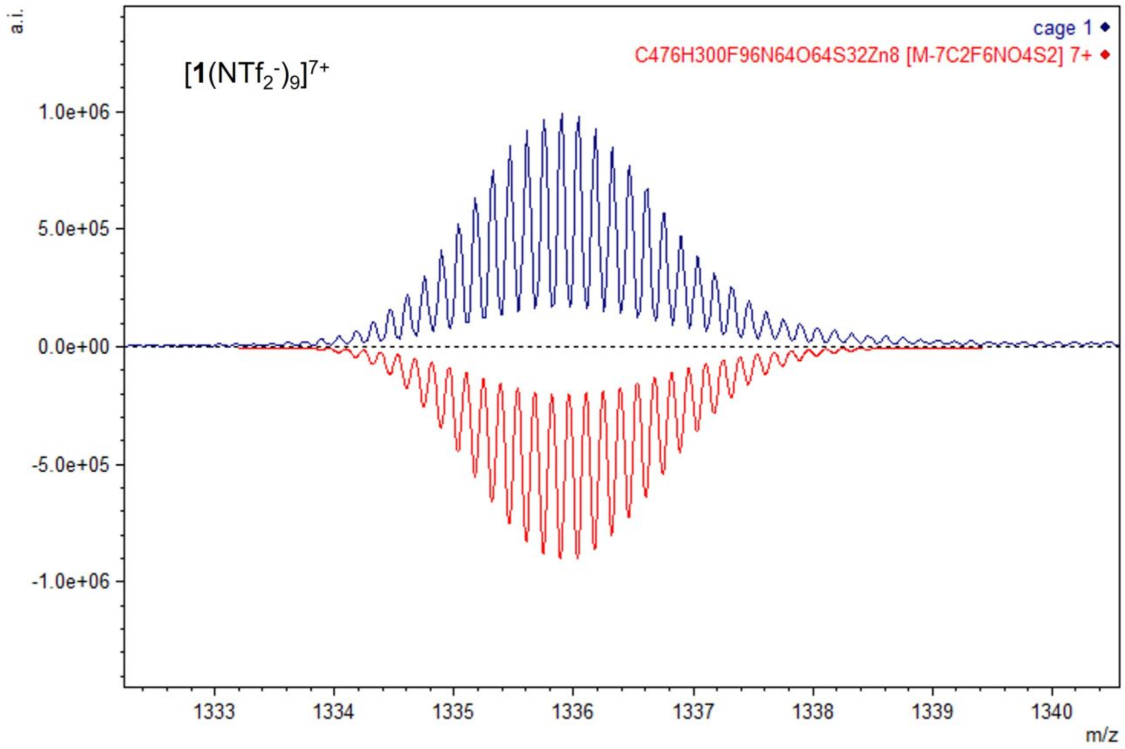

Figure S12: High-resolution ESI-mass spectrometry analysis of **1**·$(NTf_2^-)_{16}$ showing the observed (blue) and theoretical (red) isotope patterns for the +8 and +7 peaks.

3.2 Construction of tetrahedron cage 2

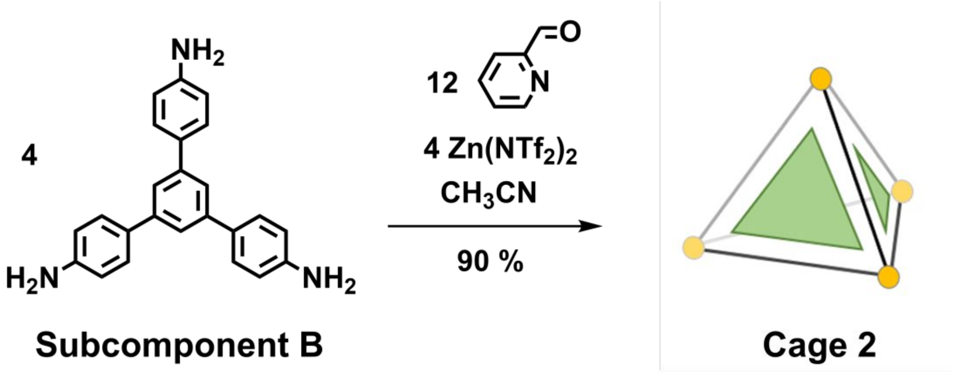

Scheme S3: Subcomponent self-assembly of cage **2**.

The preparation of cage **2** followed previously reported procedures.[2,3]

Subcomponent **B** (1.0 mg, 2.8 μmol, 1.0 equiv) was added to CD_3CN (0.6 mL) together with $Zn(NTf_2)_2$ (2.1 mg, 3.6 μmol, 1.3 equiv) and 2-formylpyridine (0.90 mg, 8.4 μmol, 3.0 equiv). The reaction mixture was stirred at 70 °C for 12 h. After cooling to room temperature, the solvent was evaporated and Et_2O was then added. The residue was resuspended and then centrifuged and the diethyl ether decanted. This was repeated three times with fresh diethyl ether. The residue was then dried *in vacuo* to afford the desired product (Cage **2**) as a yellow solid (3.3 mg, 90% yield).

^{1}H NMR (400 MHz, CD_3CN, 298 K) δ (ppm) = 8.75 (s, 12H, $\boldsymbol{H}_a$), 8.52 - 8.48 (m, 12H, $\boldsymbol{H}_b$), 8.32 (d, 12H, J = 6.9 Hz, $\boldsymbol{H}_c$), 7.94 - 7.91 (m, 24H, $\boldsymbol{H}_d$, $\boldsymbol{H}_e$), 7.53 (s, 12H, $\boldsymbol{H}_f$), 7.31 (d, 24H, J = 8.5 Hz, $\boldsymbol{H}_g$), 6.47 (d, 24H, J = 8.4 Hz, $\boldsymbol{H}_h$).

ESI-MS: *m*/*z*: 430.75 $[\mathbf{2}(NTf_2)]^{7+}$, 549.35 $[\mathbf{2}(NTf_2)_2]^{6+}$, 715.17 $[\mathbf{2}(NTf_2)_3]^{5+}$, 964.17 $[\mathbf{2}(NTf_2)_4]^{4+}$, 1378.3 $[\mathbf{2}(NTf_2)_5]^{3+}$.

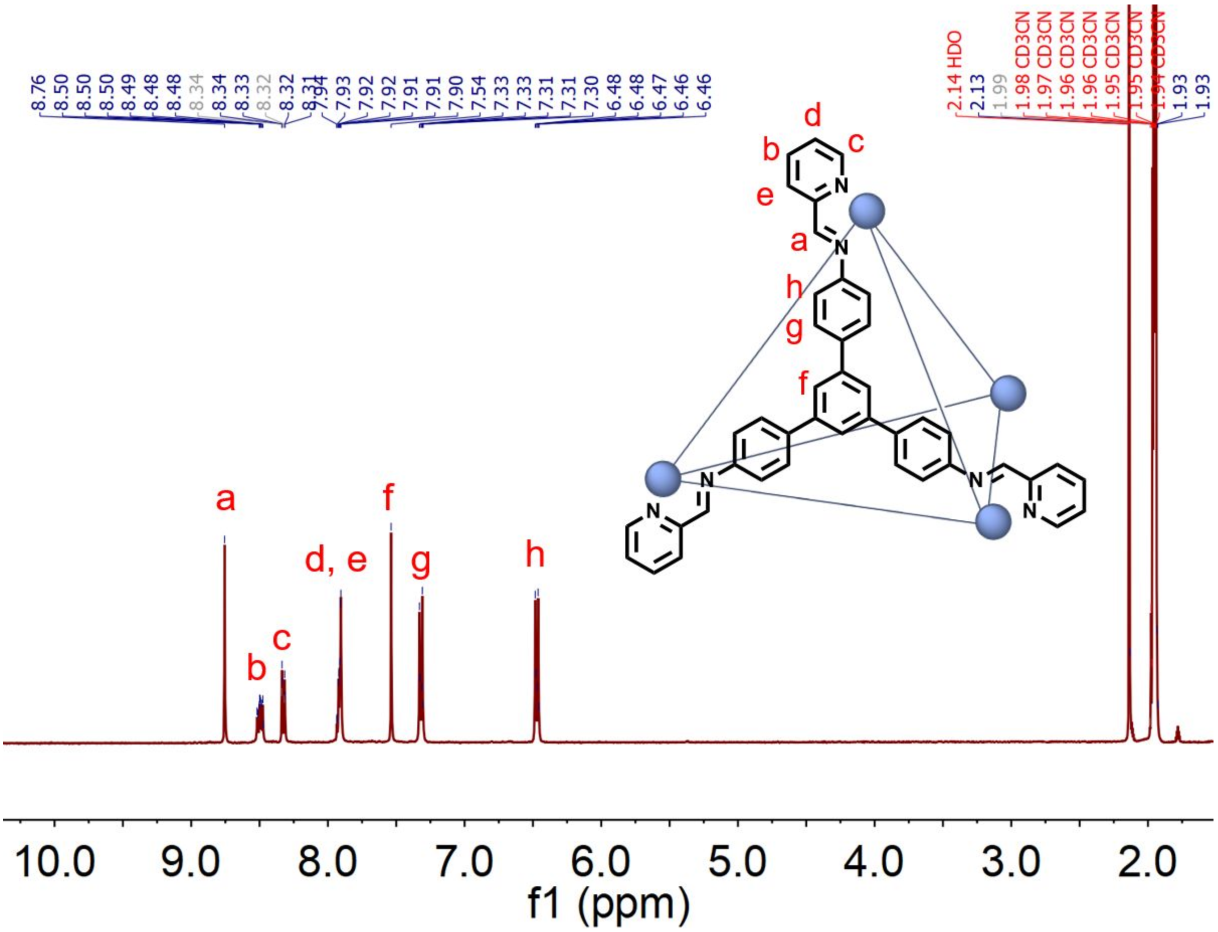

Figure S13. ^{1}H NMR spectrum of cage **2** (400 MHz, CD_3CN, 298 K).

3.3 Formation of a mixture of cages 1, 2 and 3

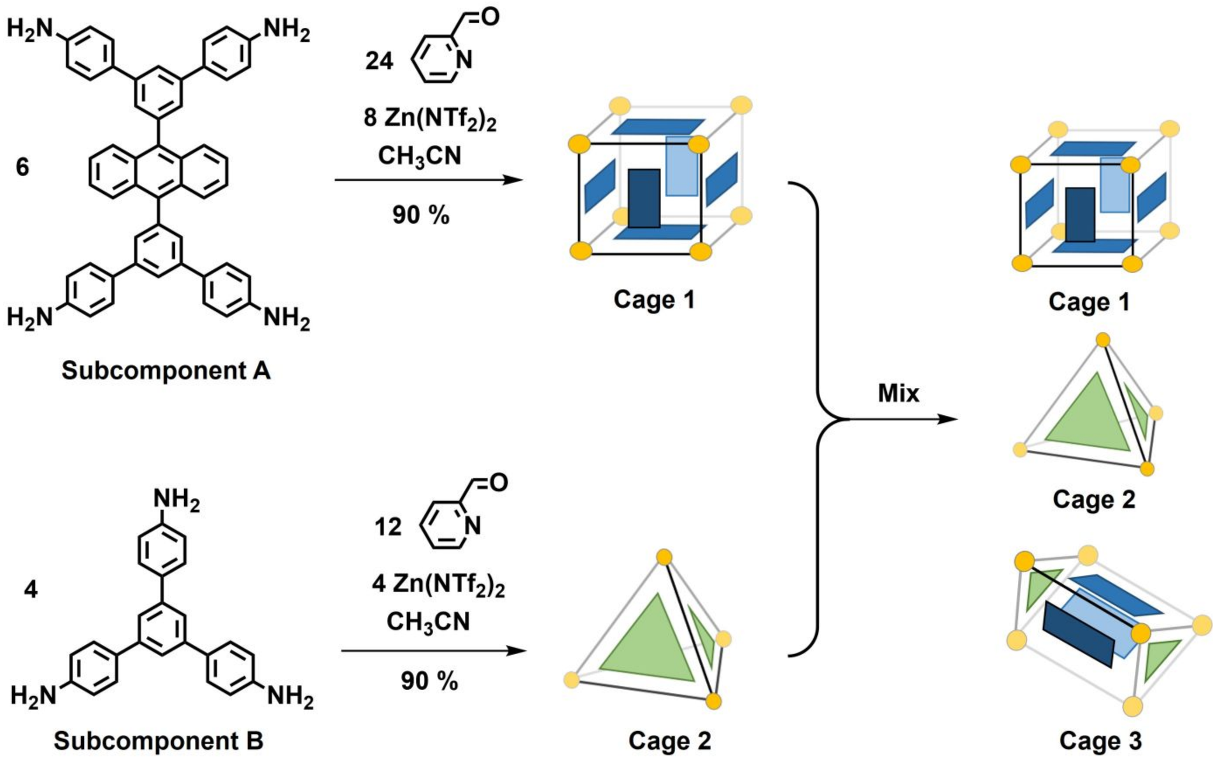

Scheme S4. Cage **1** (2.55 mg, 1 equiv) and cage **2** (1.12 mg, 1 equiv) were mixed in CD_3CN (0.45 mL) in a react tube. The reaction mixture was stirred at 65 °C for 24 h until equilibration. After cooling to room temperature, a mixture of cages **1**, **2** and **3** was formed.

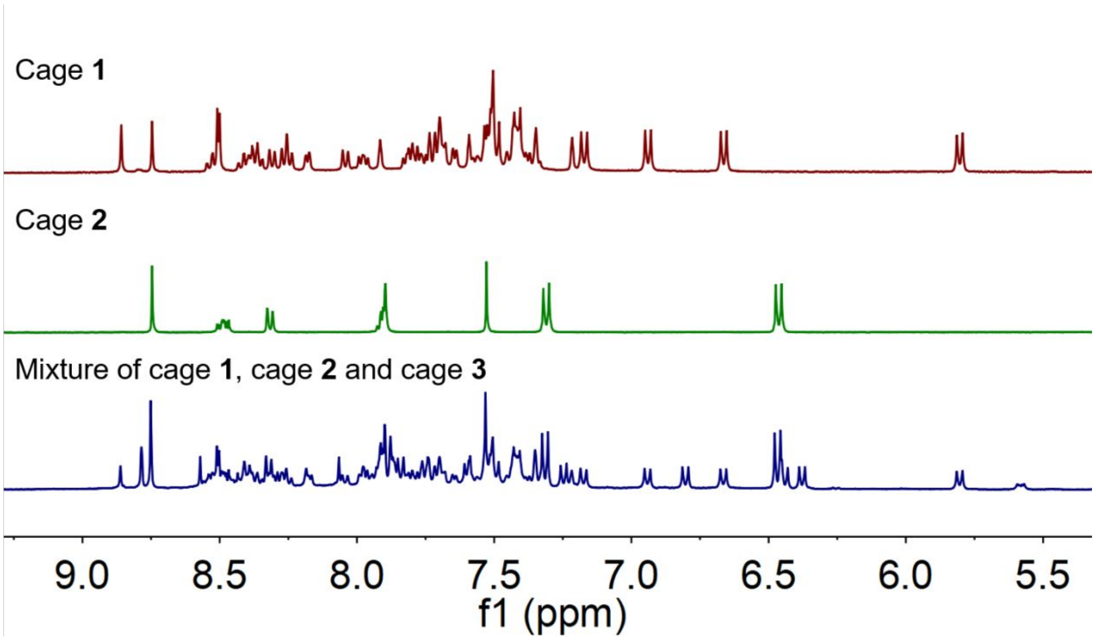

Figure S14. Partial ^1H NMR spectrum of **a)** cage **1**; **b)** Cage **2**; **c)** Cage mixture of cage **1**, cage **2** and cage **3** (400 MHz, CD_3CN, 298 K).

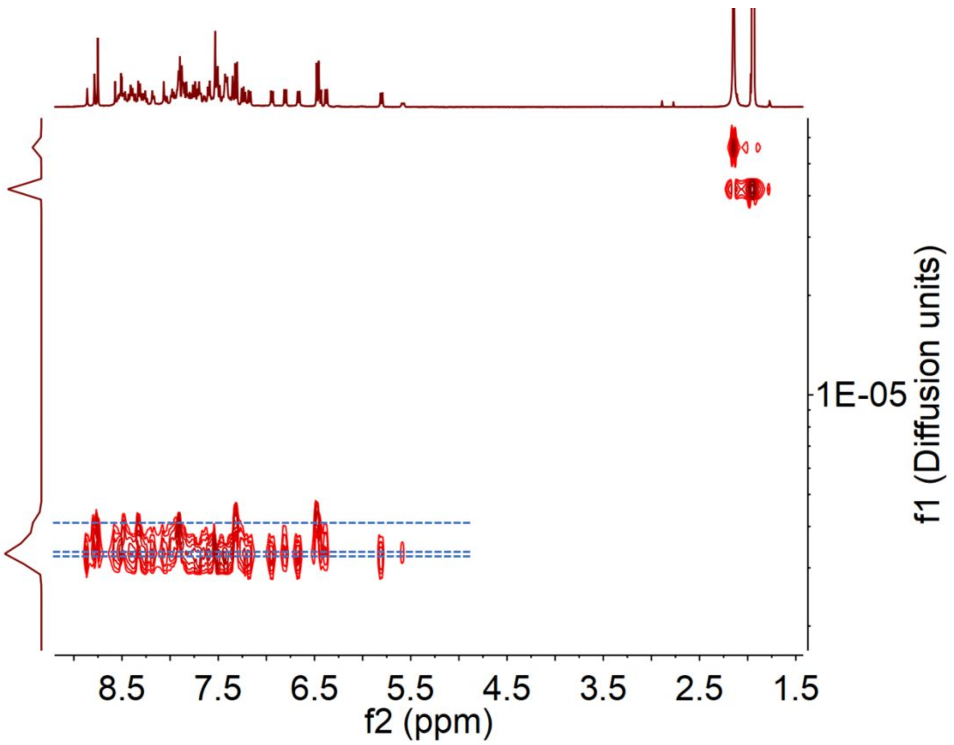

Figure S15. ^{1}H DOSY NMR spectrum of a mixture of cages **1**, **2** and **3**. The three cage species were observed in the DOSY spectrum (400 MHz, CD_3CN, 298 K).

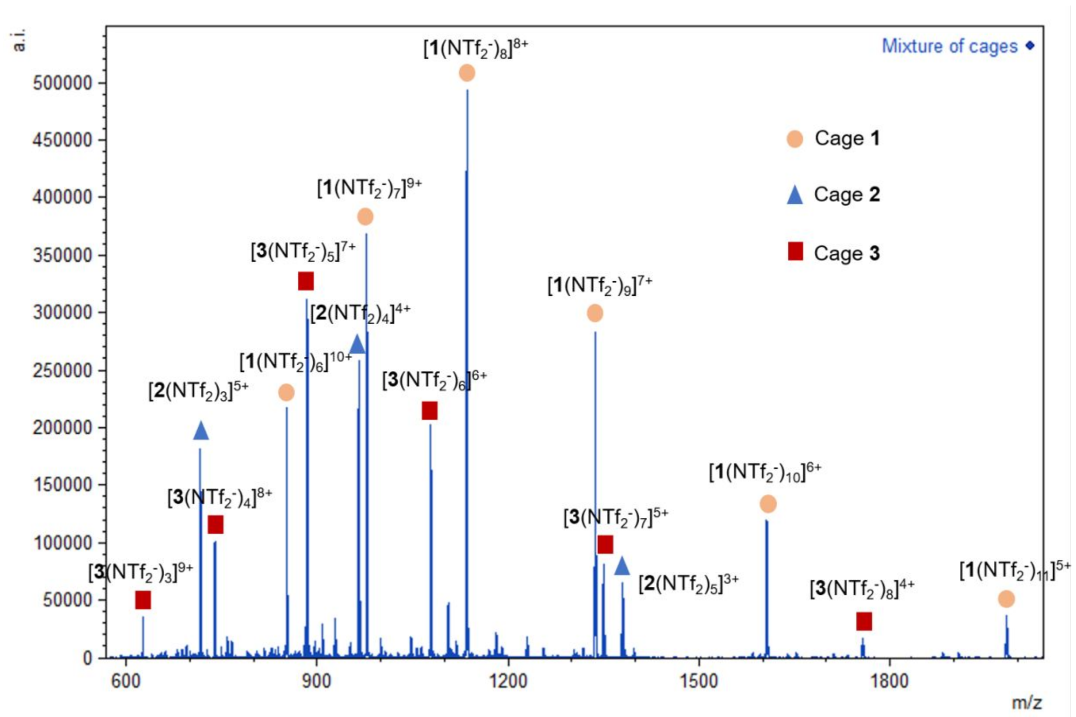

Figure S16. Low resolution ESI-MS spectrum of a mixture of cages **1-3**. Experimental **ESI-MS** of cage **3**: *m/z*: 624.9 [**3**$(NTf_2)_3]^{9+}$, 738.1 [**3**$(NTf_2)_4]^{8+}$, 883.3 [**3**$(NTf_2)_5]^{7+}$, 1077.1 [**3**$(NTf_2)_6]^{6+}$, 1348.8 [**3**$(NTf_2)_7]^{5+}$, 1756.2 [**3**$(NTf_2)_8]^{4+}$. Calculated: *m/z*: 625.5 [**3**$(NTf_2)_3]^{9+}$, 738.8 [**3**$(NTf_2)_4]^{8+}$, 884.3 [**3**$(NTf_2)_5]^{7+}$, 1078.4 [**3**$(NTf_2)_6]^{6+}$, 1350.1 [**3**$(NTf_2)_7]^{5+}$, 1757.7 [**3**$(NTf_2)_8]^{4+}$.

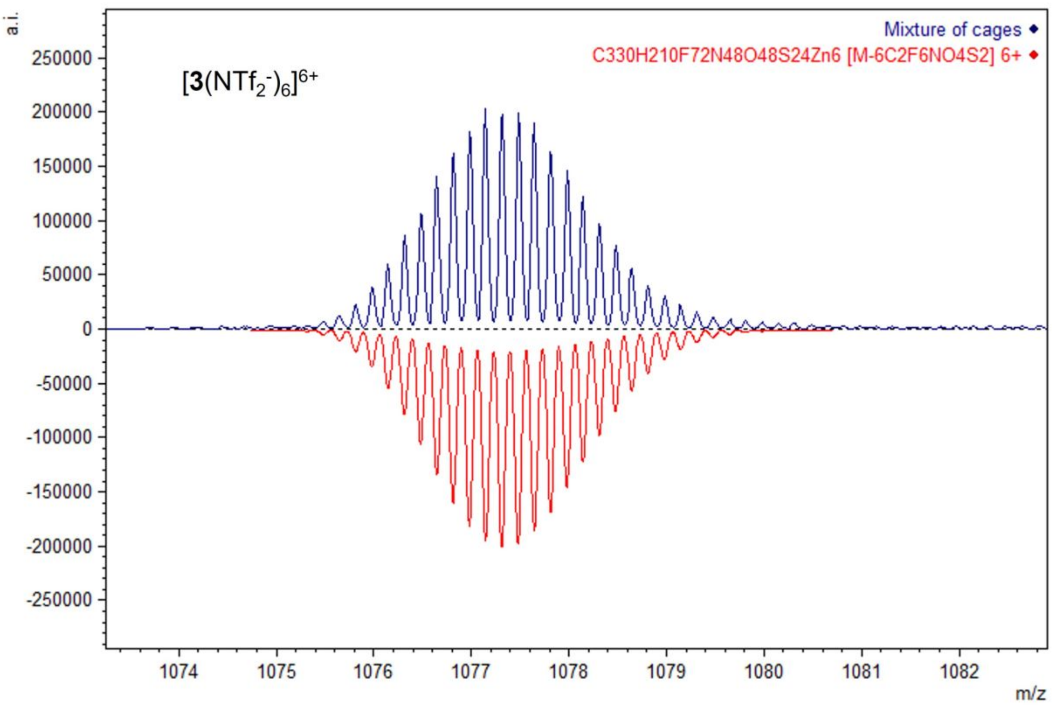

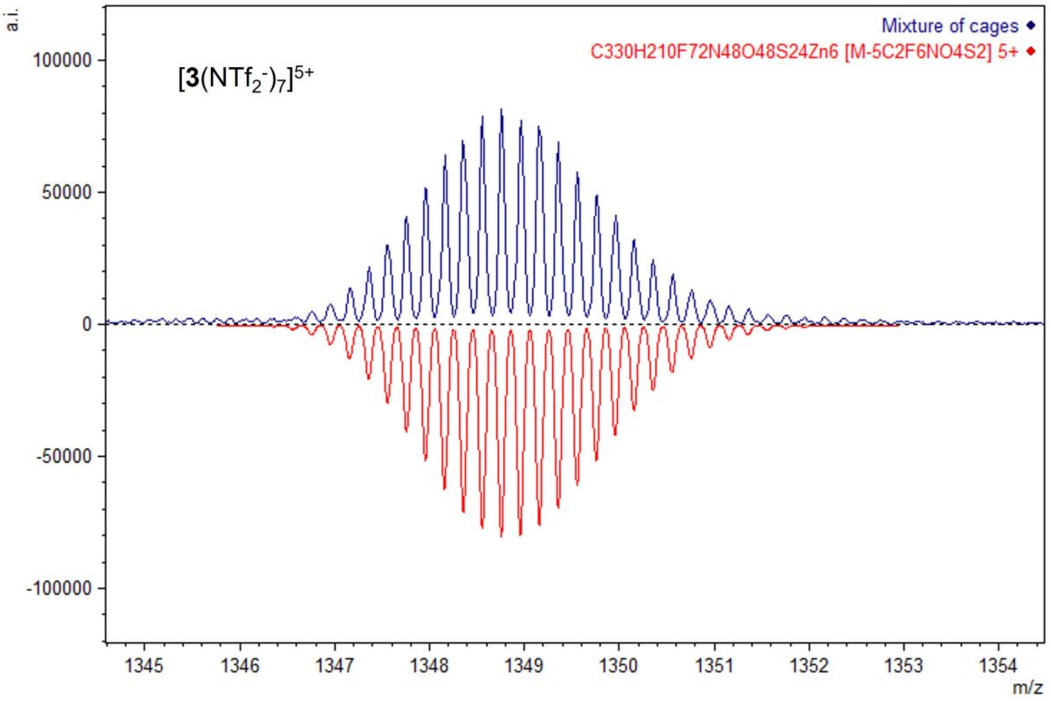

Figure S17. High-resolution ESI-mass spectrometry analysis of cage **3** (**3**$(NTf_2^-)_{12}$) in the cage mixture showing the observed (blue) and theoretical (red) isotope patterns for the +6 and +5 peaks.

3.4 Characterization of oxidized trigonal prism cage 4

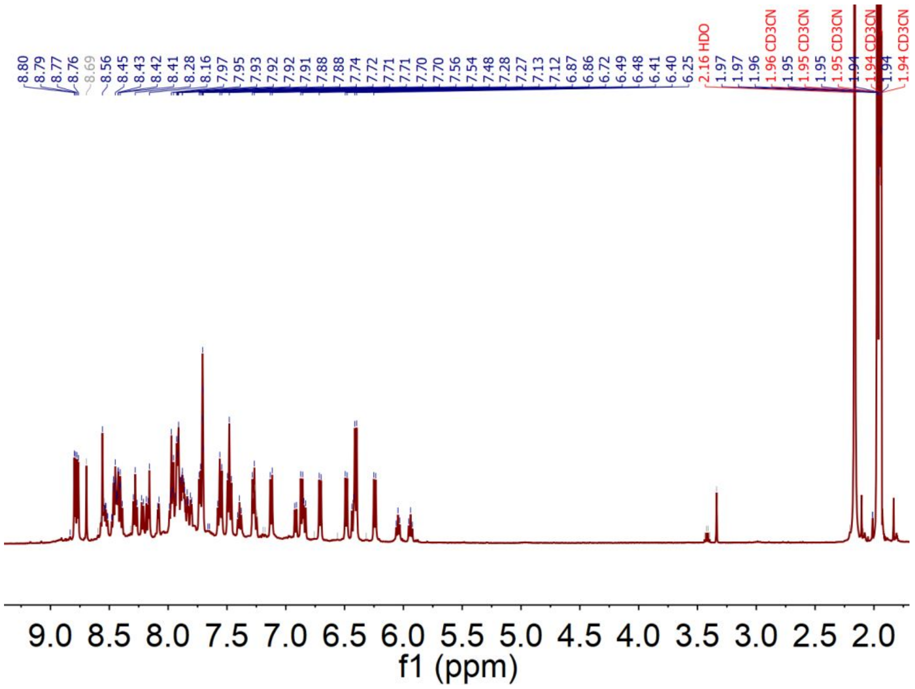

Figure S18. ^{1}H NMR spectrum of cage **4** (500 MHz, CD_3CN, 298 K).

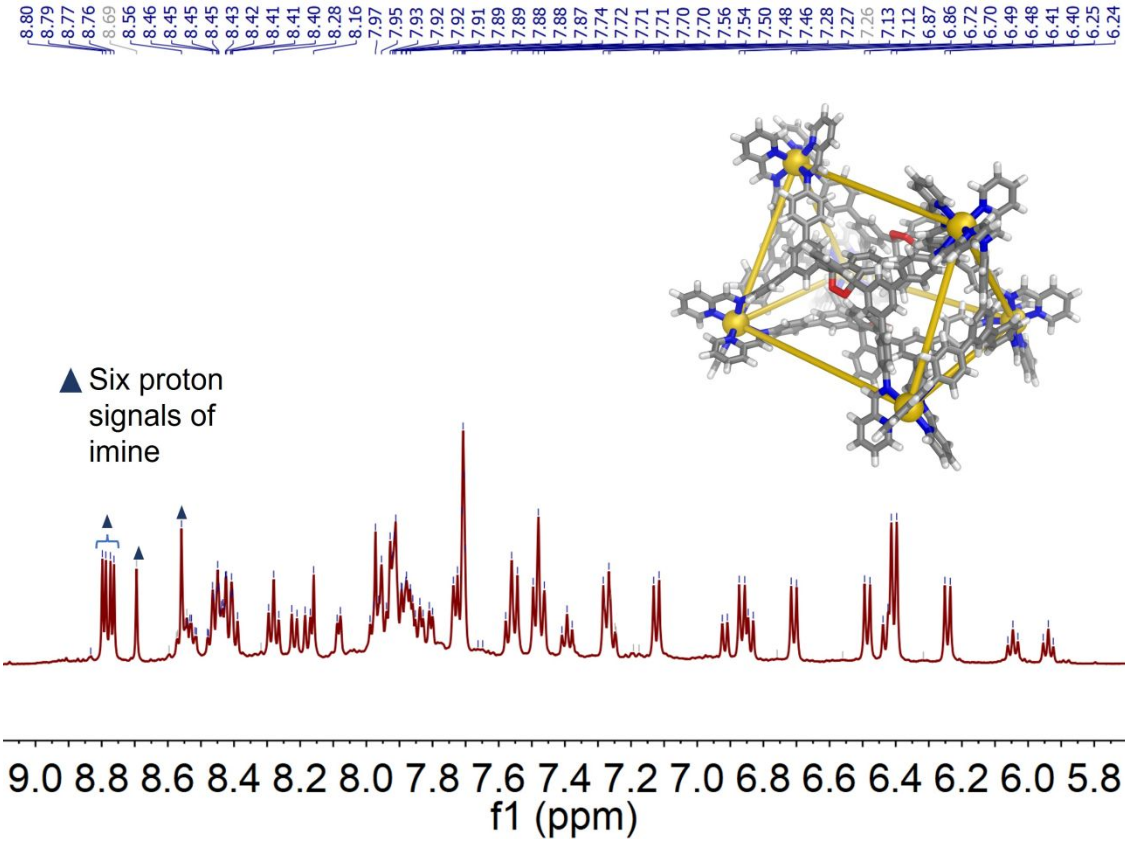

Figure S19. Aromatic region of ^{1}H NMR spectrum of cage **4** (500 MHz, CD_3CN, 298 K). Six imine peaks indicated a C_3-symmetric structure.

^{1}**H NMR** (500 MHz, CD_3CN, 298 K) δ (ppm) = 8.80 (s, 3H), 8.79 (s, 3H), 8.77 (s, 3H), 8.76 (s, 3H), 8.69 (s, 3H), 8.60 – 8.51 (m, 9H), 8.50 – 8.38 (m, 18H), 8.28 (t, *J* = 7.7 Hz, 6H), 8.22 (d, *J* = 7.8 Hz, 3H), 8.20 – 8.15 (m, 6H), 8.08 (d, *J* = 5.0 Hz, 3H), 8.00 – 7.78 (m, 33H), 7.75 – 7.69 (m, 12H), 7.56 (t, *J* = 8.8 Hz, 9H), 7.48 (t, *J* = 8.4 Hz, 12H), 7.39 (t, *J* = 7.7 Hz, 3H), 7.26 (td, *J* = 8.4, 2.1 Hz, 9H), 7.12 (d, *J* = 8.2 Hz, 6H), 6.92 (d, *J* = 7.7 Hz, 3H), 6.85 (dd, *J* = 13.2, 8.0 Hz, 9H), 6.71 (d, *J* = 8.2 Hz, 6H), 6.49 (d, *J* = 8.1 Hz, 6H), 6.42 (dd, *J* = 12.8, 7.9 Hz, 12H), 6.24 (d, *J* = 8.2 Hz, 6H), 6.05 (t, *J* = 7.6 Hz, 3H), 5.94 (t, *J* = 7.6 Hz, 3H).

^{13}**C NMR** (126 MHz, CD_3CN, 298 K) δ (ppm) = 165.75 , 165.27 , 164.67 , 164.38 , 164.25 , 163.62 , 149.59 , 147.97 , 147.57 , 147.13 , 146.91 , 146.55 , 146.31 , 146.23 , 146.11 , 145.89 , 143.46 , 143.00 – 142.38 (m), 141.86 , 141.71 , 141.50 , 141.35 , 141.19 , 141.00 , 140.64 , 140.27 , 140.16 , 139.42 , 139.04 , 138.85 , 137.93, 137.91, 134.09 , 134.00 , 131.34 , 131.19 – 130.62 (m), 129.07 , 128.89 , 128.37 (d, *J* = 14.1 Hz), 128.21 , 128.10 , 127.82 , 127.37 , 126.83 , 126.39 , 125.59 , 125.37 , 124.05 , 123.69 , 123.30 , 123.13 , 123.02 , 122.68 , 122.52 , 122.08 , 121.99 , 121.63 , 121.14 , 84.42 , 84.34 .

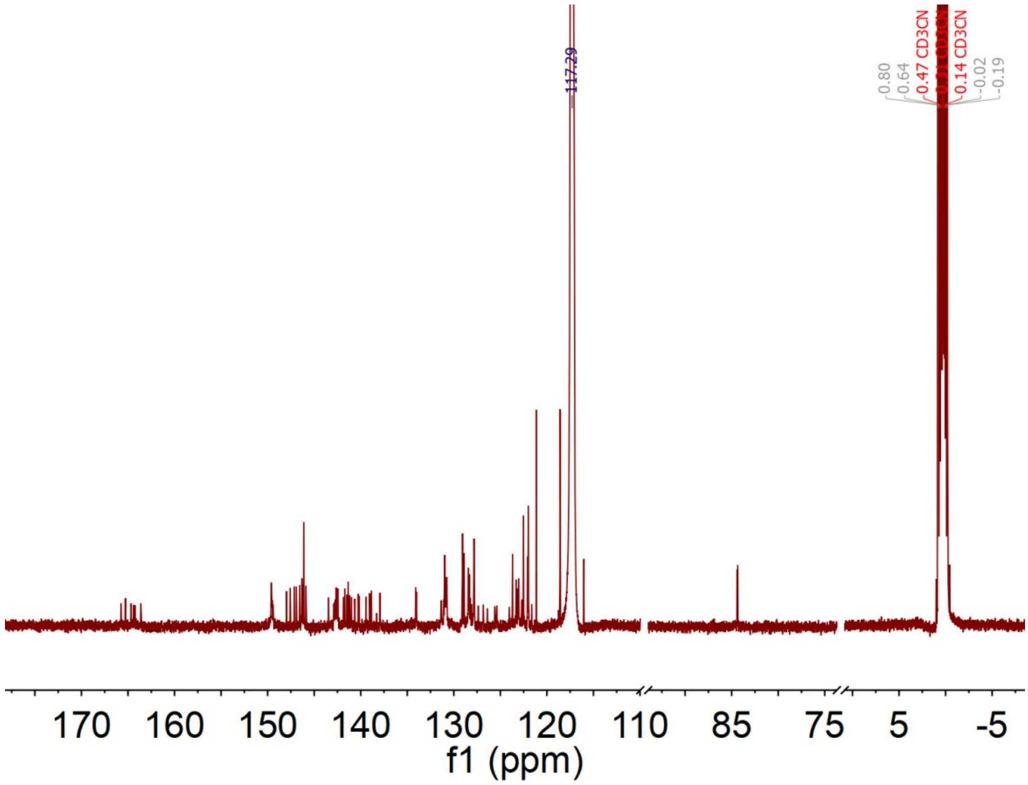

Figure S20. ^{13}C NMR spectrum (126 MHz, 298 K, CD3CN) of **4**.

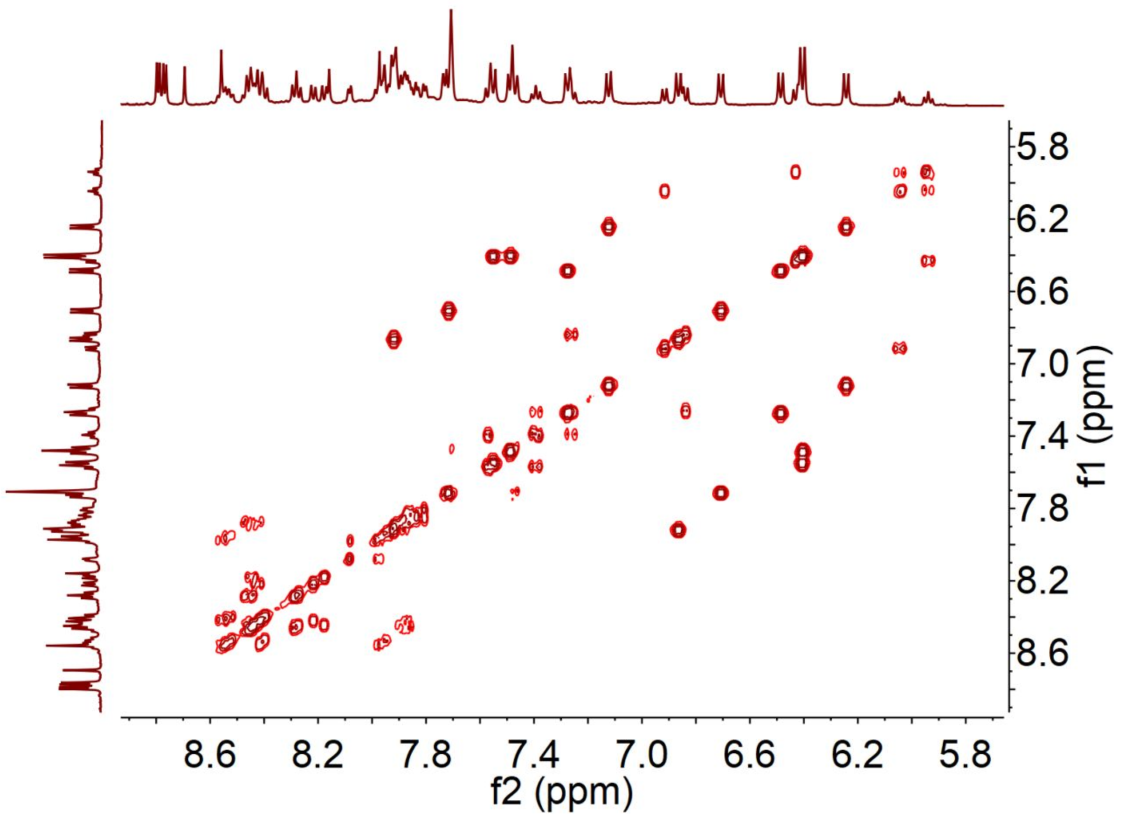

Figure S21. Aromatic region of the ^{1}H-^{1}H COSY NMR spectrum of cage **4** (500 MHz, CD_3CN, 298 K)

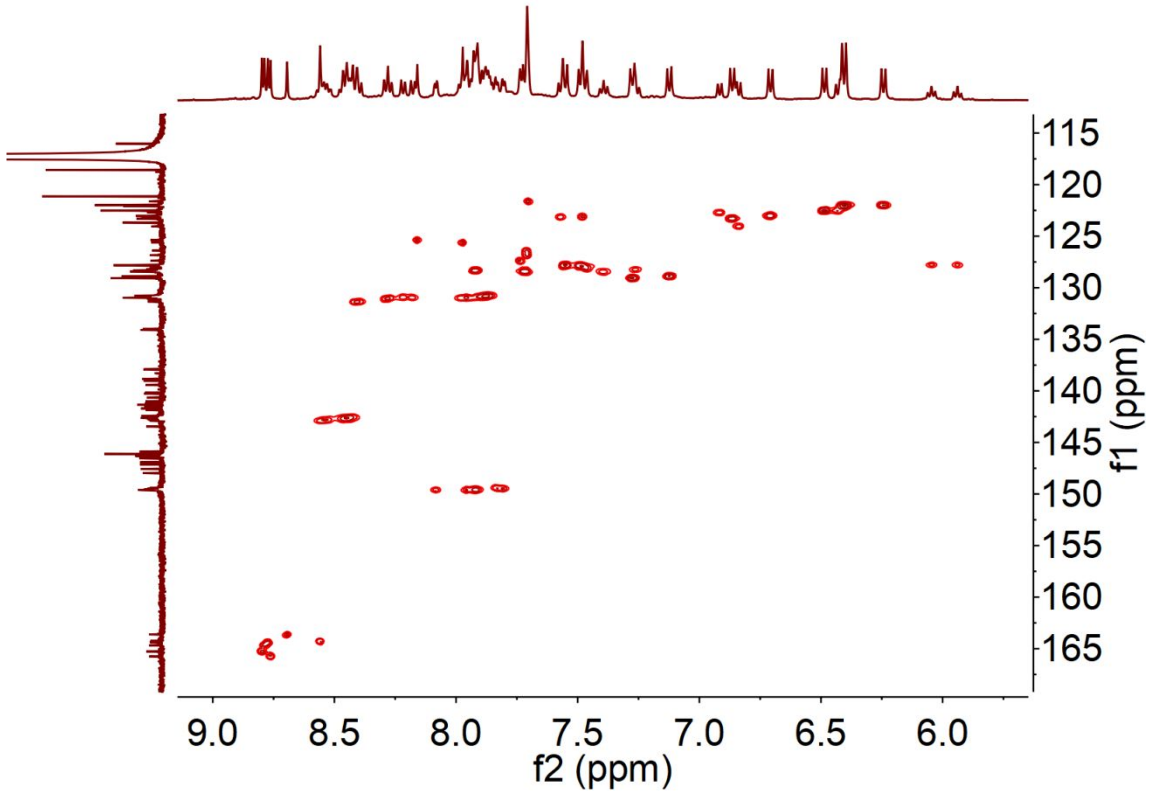

Figure S22. Aromatic region of the ^{1}H-^{13}C HSQC NMR spectrum of **4** (500 MHz, CD_3CN, 298 K).

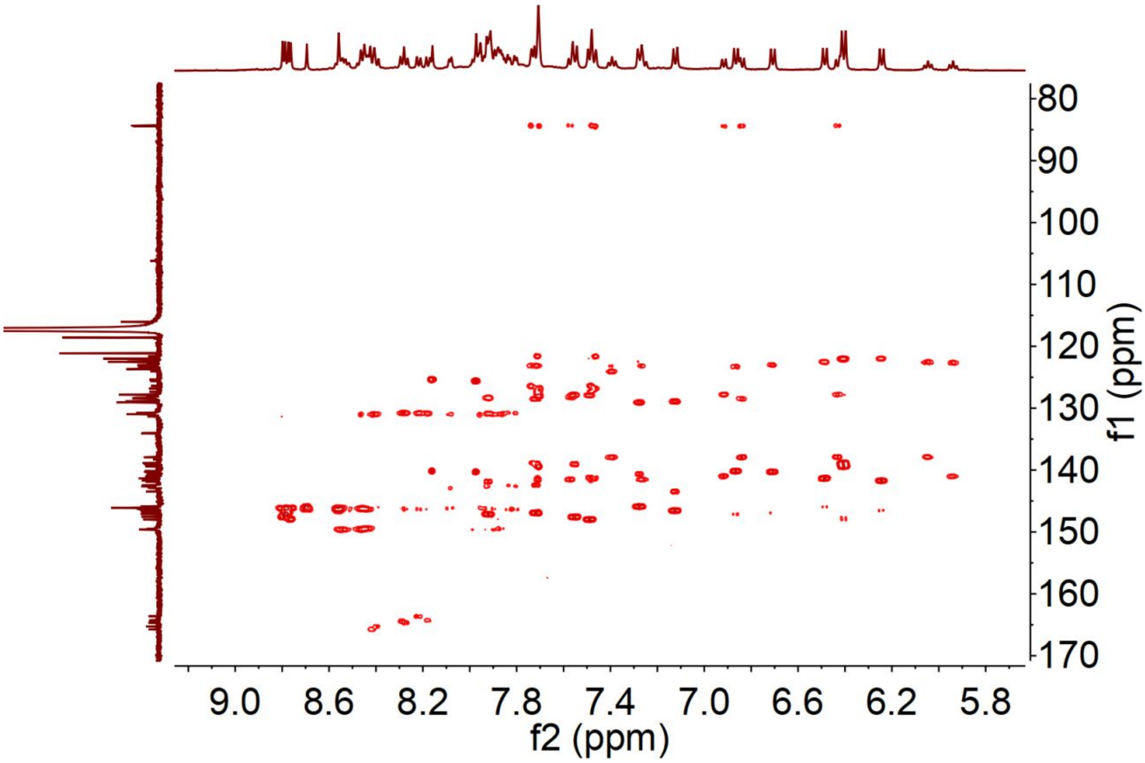

Figure S23. Aromatic region of the ^{1}H-^{13}C HMBC NMR spectrum of **4** (500 MHz, CD_3CN, 298 K).

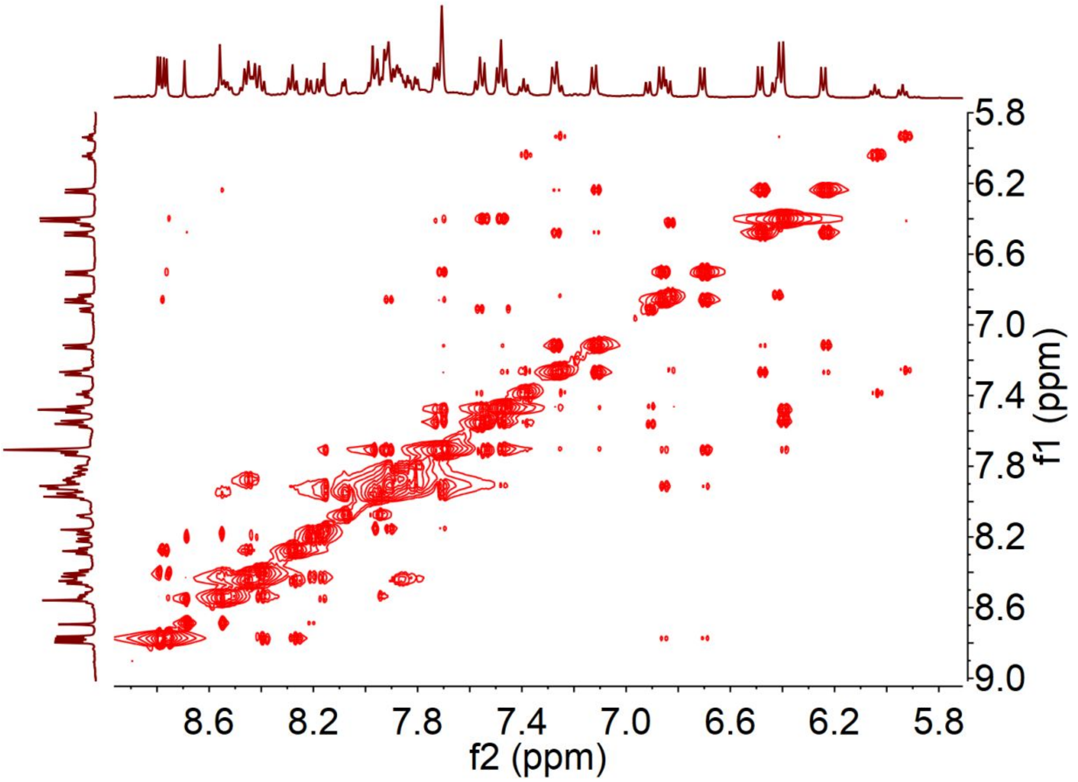

Figure S24. Aromatic region of the ^{1}H-^{1}H NOESY NMR spectrum of **4** (500 MHz, CD_3CN, 298 K).

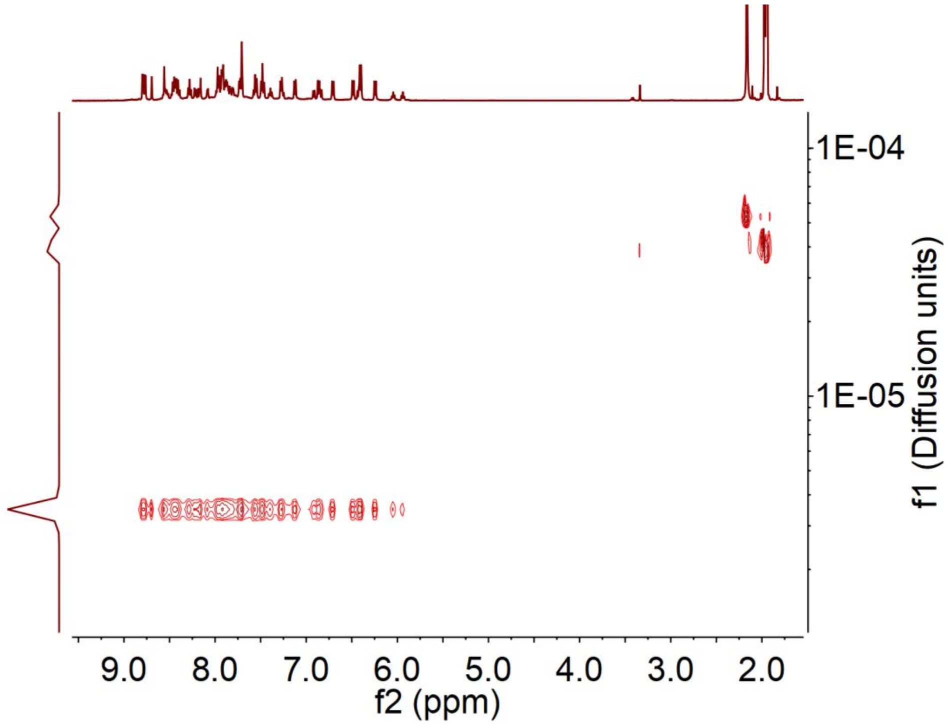

Figure S25. ^{1}H DOSY NMR spectrum of **4** (400 MHz, CD_3CN, 298 K). The diffusion coefficient for **1** in CD_3CN was measured to be 3.48×10^{-6} cm^2s^{-1}, corresponding to a hydrodynamic radius of 18.8 Å.

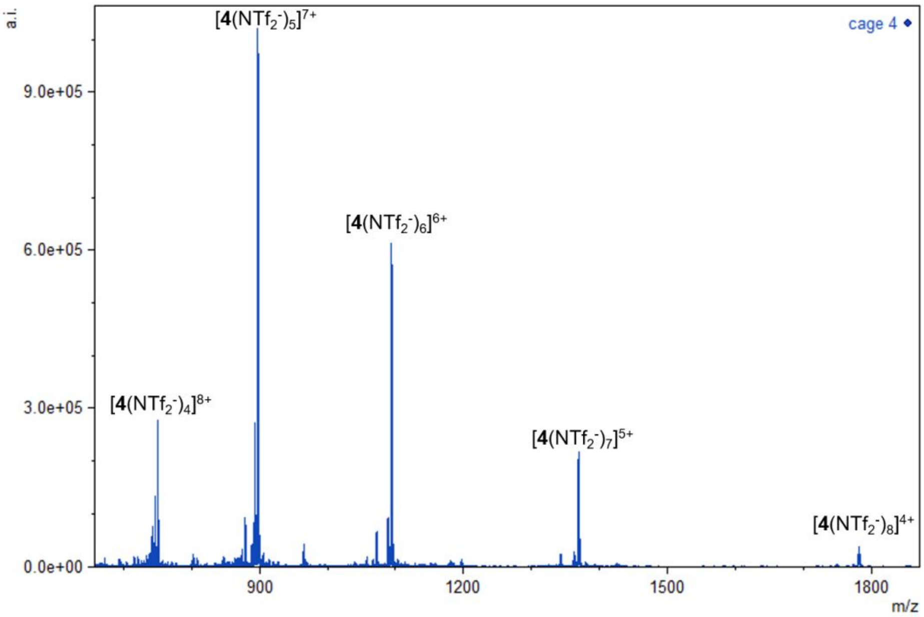

Figure S26: Low-resolution ESI-mass spectrum of $\mathbf{4}(NTf_2^-)_{12}$. ESI-MS: Experimental results: m/z = 749.9 $[\mathbf{4}(NTf_2^-)_4]^{8+}$, 897.1 $[\mathbf{4}(NTf_2^-)_5]^{7+}$, 1093.3 $[\mathbf{4}(NTf_2^-)_6]^{6+}$, 1368.1 $[\mathbf{4}(NTf_2^-)_7]^{5+}$, 1779.9 $[\mathbf{4}(NTf_2^-)_8]^{4+}$. Calculated results: m/z = 749.6 $[\mathbf{4}(NTf_2^-)_4]^{8+}$, 896.8 $[\mathbf{4}(NTf_2^-)_5]^{7+}$, 1092.9 $[\mathbf{4}(NTf_2^-)_6]^{6+}$, 1367.5 $[\mathbf{4}(NTf_2^-)_7]^{5+}$, 1779.4 $[\mathbf{4}(NTf_2^-)_8]^{4+}$.

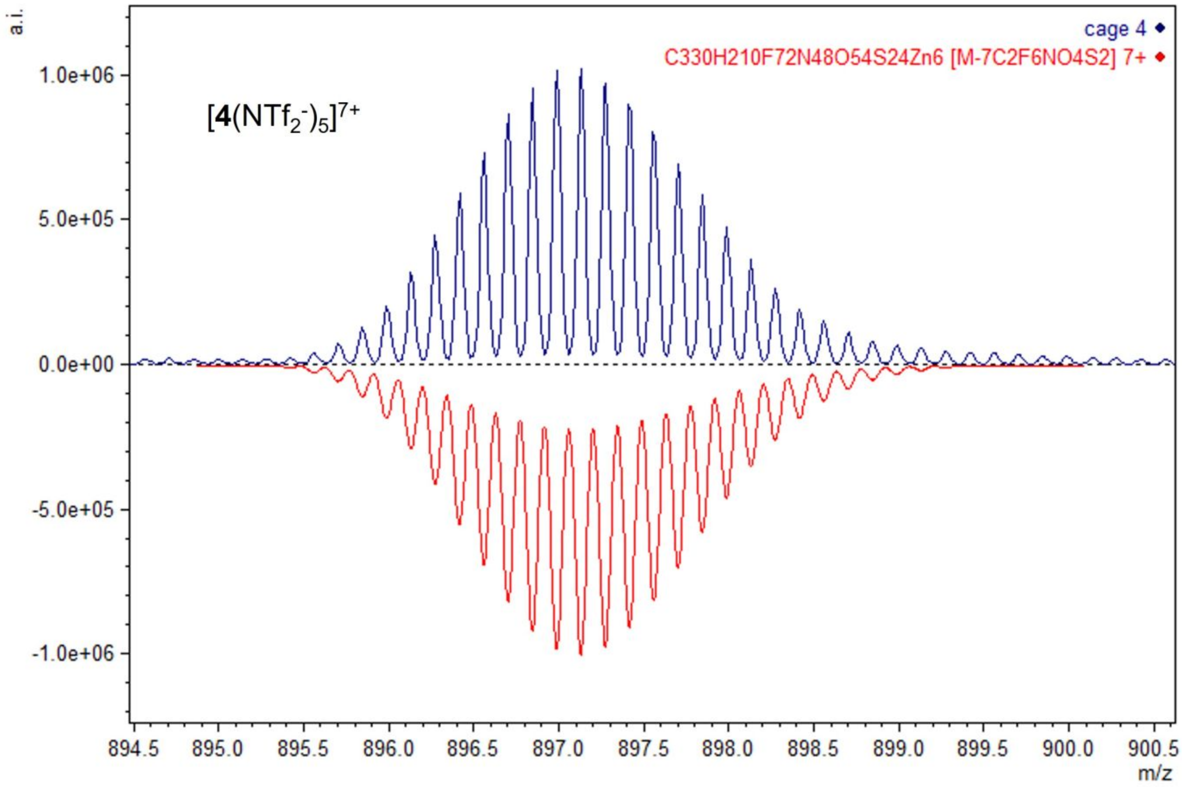

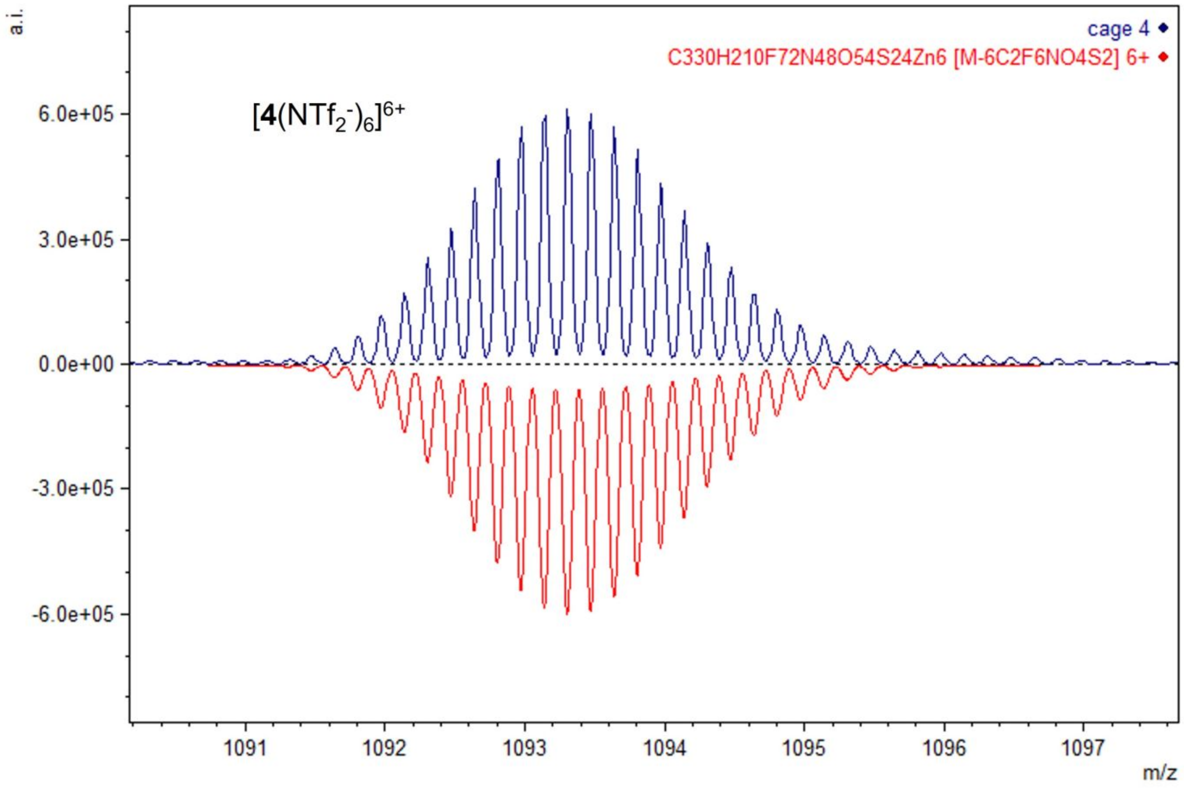

Figure S27: High-resolution ESI-mass spectrometry analysis of **4**$(NTf_2)_{12}$ showing the observed (blue) and theoretical (red) isotope patterns for the +7 and +6 peaks.

3.5 Characterization of oxidized cubic cage 5

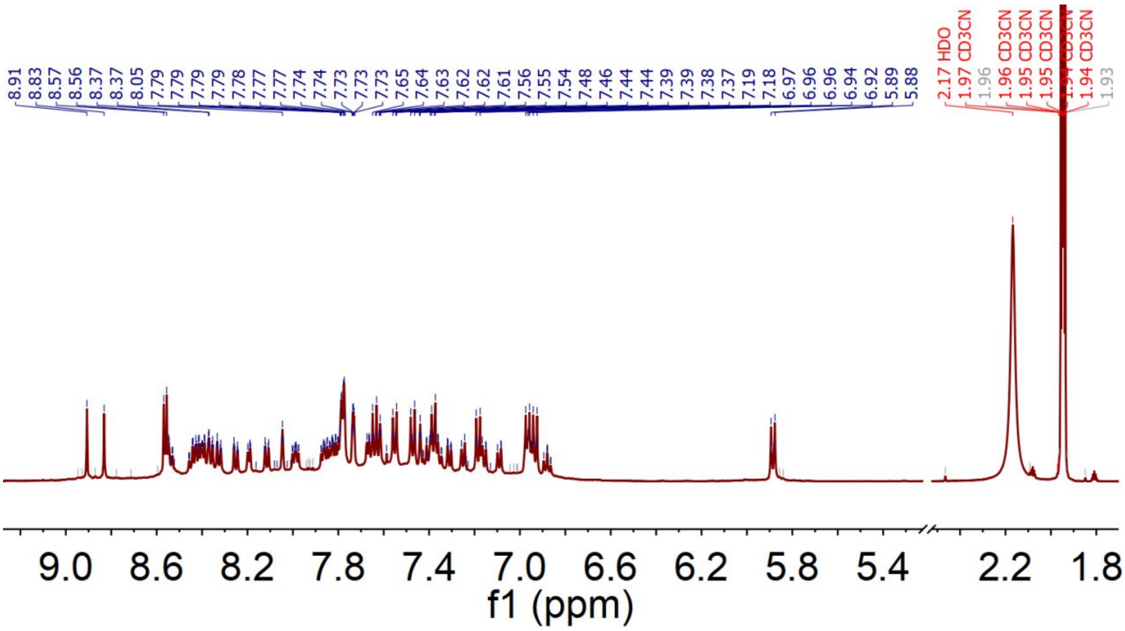

Figure S28. ^{1}H NMR spectrum of cage **5** (400 MHz, CD_3CN, 298 K).

^{1}H NMR (500 MHz, CD_3CN, 298 K) δ (ppm) = 8.91 (s, 6H), 8.83 (s, 6H), 8.59-8.51 (m, 30H), 8.47-8.31 (m, 36H), 8.25 (d, *J* = 7.4 Hz, 6H), 8.20 (d, *J* = 7.4 Hz, 6H), 8.11 (d, *J* = 7.4 Hz, 6H), 8.05 (t, *J* = 1.6 Hz, 6H), 7.99 (ddd, *J* = 8.0, 5.1, 1.2 Hz, 6H), 7.90-7.76 (m, 42H), 7.73 (dt, *J* = 3.1, 1.6 Hz, 12H), 7.69-7.60 (m, 24H), 7.56 (d, *J* = 7.4 Hz, 6H), 7.49-7.46 (d, *J* = 7.4 Hz, 6H), 7.40 (d, *J* = 7.4 Hz, 6H), 7.31 (dd, *J* = 7.7, 1.3 Hz, 6H), 7.25 (d, *J* = 7.4 Hz, 6H), 7.21-7.14 (m, 18H), 7.10 (dd, *J* = 7.7, 1.3 Hz, 6H), 7.00-6.91 (m, 36H), 6.88 (t, *J* = 1.6 Hz, 6H), 5.89 (d, *J* = 8.5 Hz, 12H).

^{13}C NMR (126 MHz, CD_3CN, 298 K) δ (ppm) = 164.62 , 164.01 , 163.83 , 163.51 , 149.83 , 149.37 , 149.06 , 148.70 , 147.31 , 146.38 , 146.27 (d, *J* = 6.6 Hz), 146.07 , 142.83 , 142.51 (d, *J* = 14.9 Hz), 141.84 , 141.72 , 141.61 , 141.41 , 140.80 , 140.43 , 140.05 , 139.83 , 139.66 , 139.52 , 134.32 , 133.87 , 131.19 , 130.85 (d, *J* = 20.2 Hz), 129.14 , 128.98 , 128.75 , 128.33 (d, *J* = 7.5 Hz), 127.90 , 127.70 , 127.12 , 126.76 , 126.10 , 123.68 , 123.34 (d, *J* = 18.6 Hz), 122.74 , 122.58 , 121.13 , 121.06 , 83.83 , 83.77 .

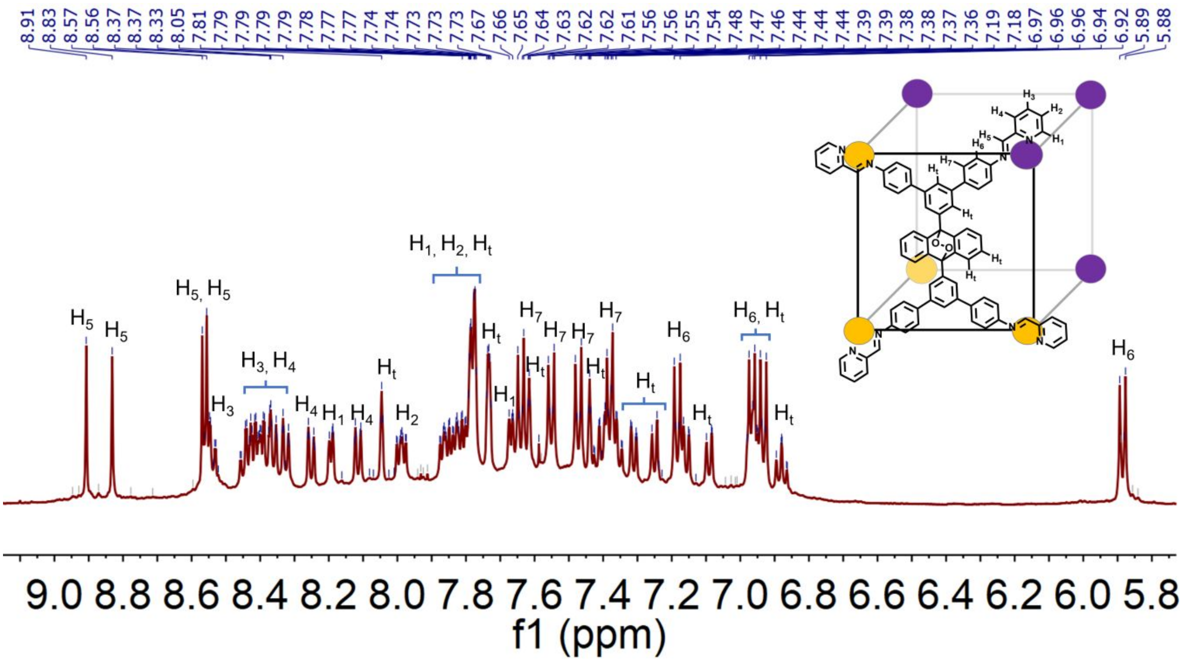

Figure S29. Aromatic region of ^{1}H NMR spectrum of cage **5** (400 MHz, CD_3CN, 298 K).

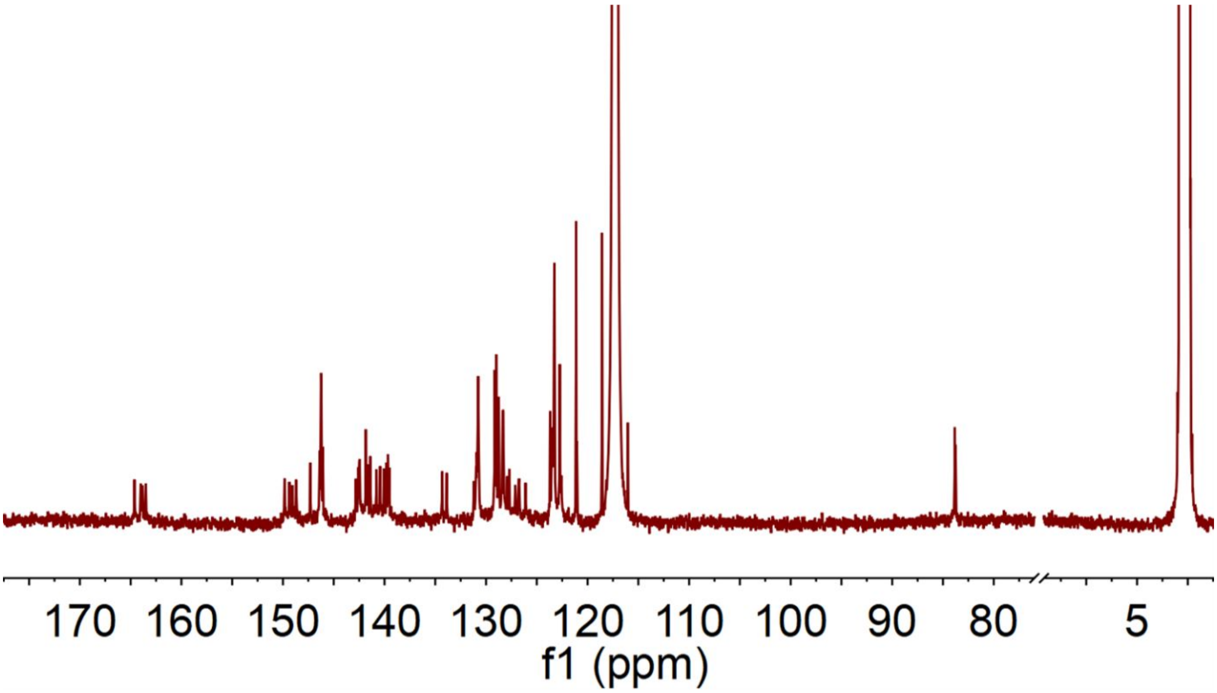

Figure S30. ^{13}C NMR spectrum of **5** (126 MHz, CD_3CN, 298 K).

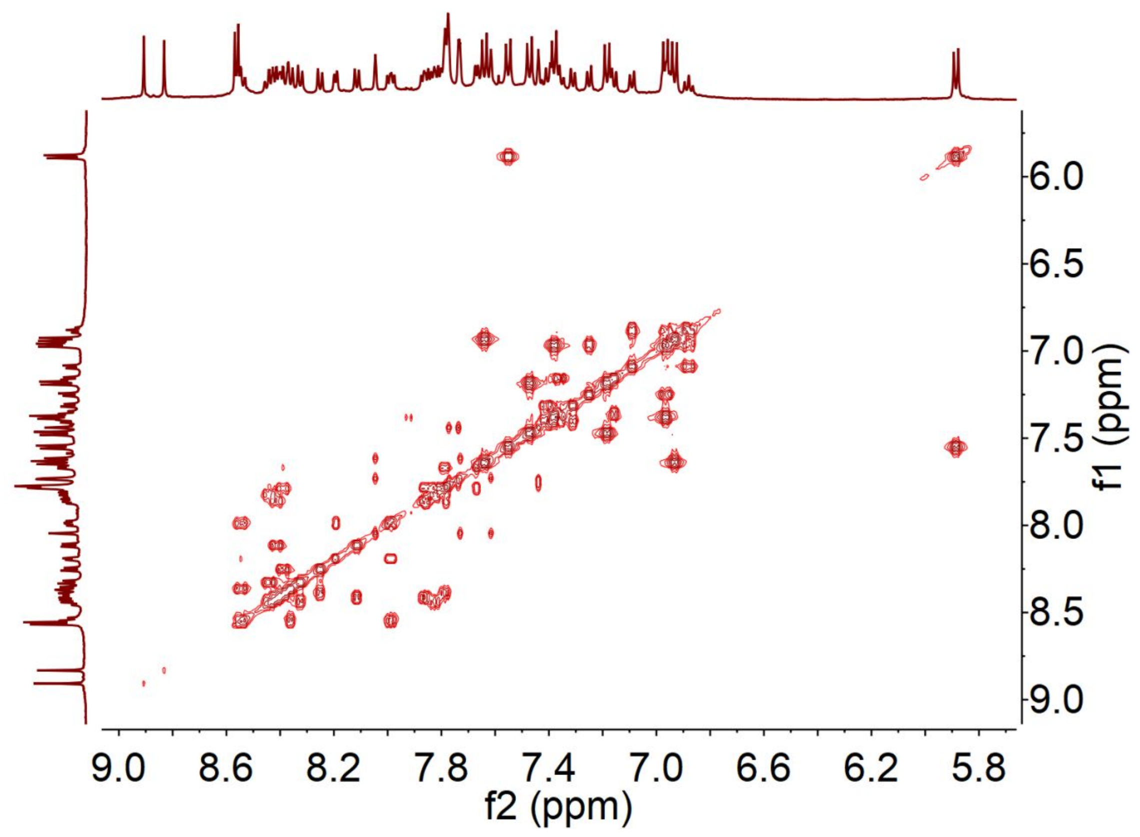

Figure S31. Aromatic region of the ^{1}H-^{1}H COSY NMR spectrum of **5** (500 MHz, CD_3CN, 298 K)

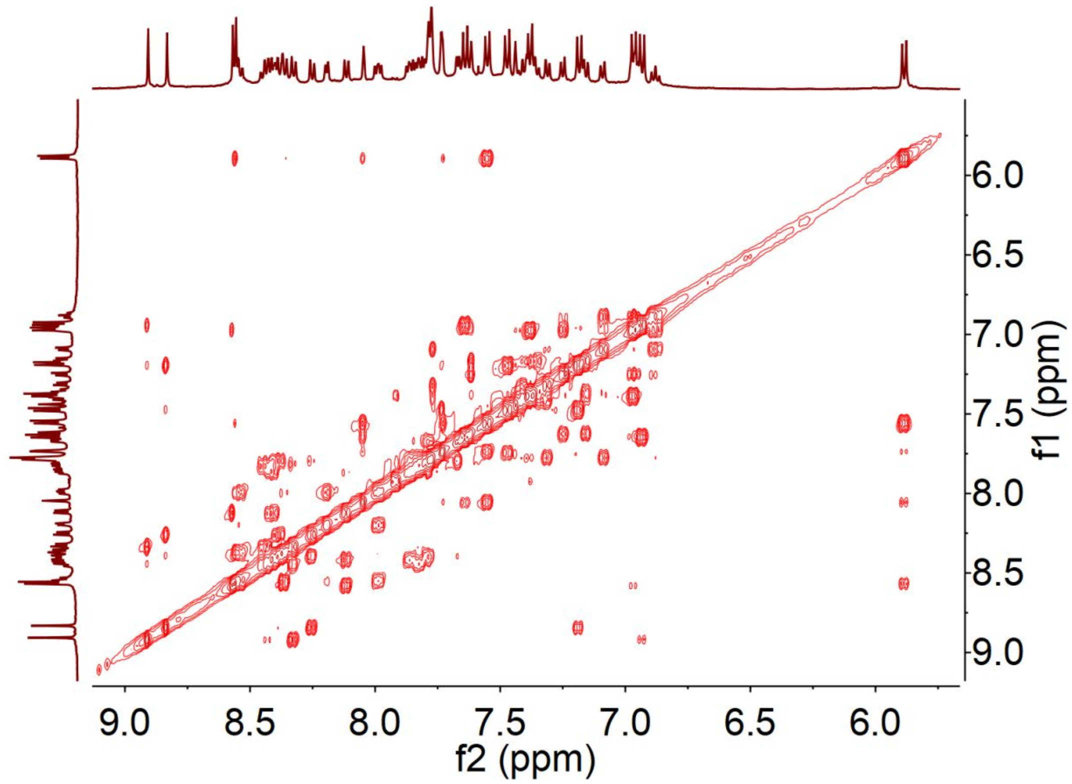

Figure S32. Aromatic region of the ^{1}H-^{1}H NOESY NMR spectrum of **5** (500 MHz, CD_3CN, 298 K).

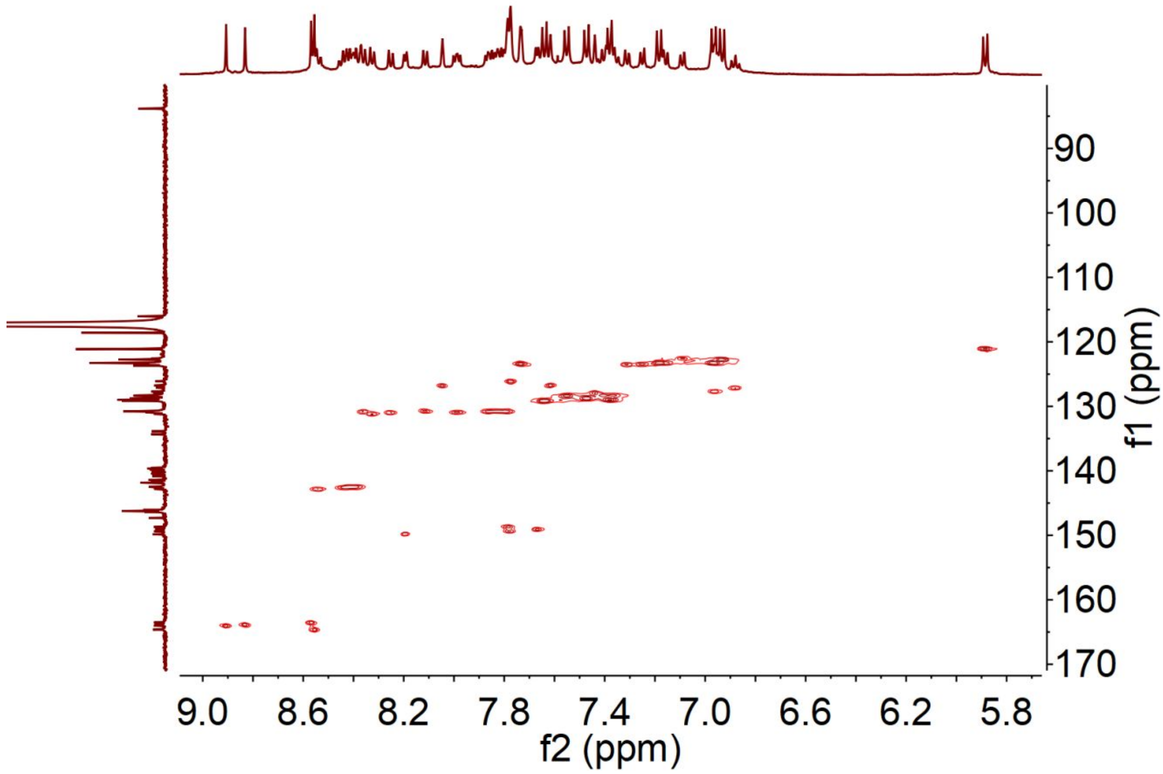

Figure S33. Aromatic region of the ^{1}H-^{13}C HSQC NMR spectrum of **5** (500 MHz, CD_3CN, 298 K).

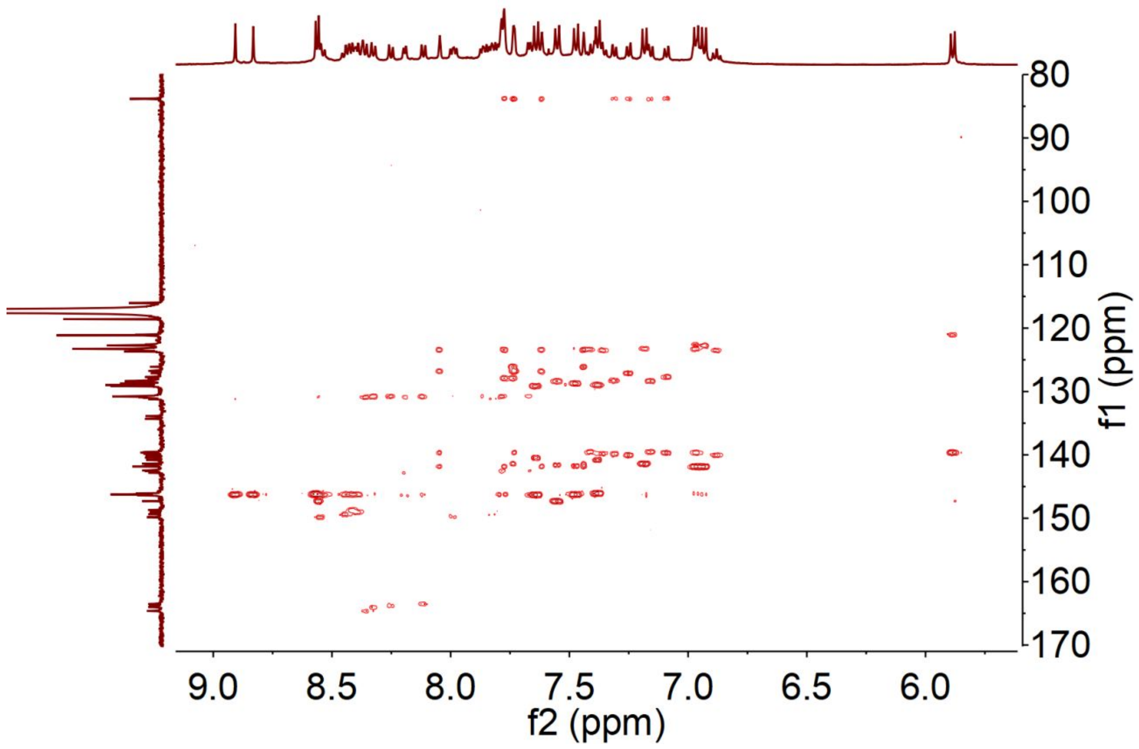

Figure S34. Aromatic region of the ^{1}H-^{13}C HMBC NMR spectrum of **5** (500 MHz, CD_3CN, 298 K).

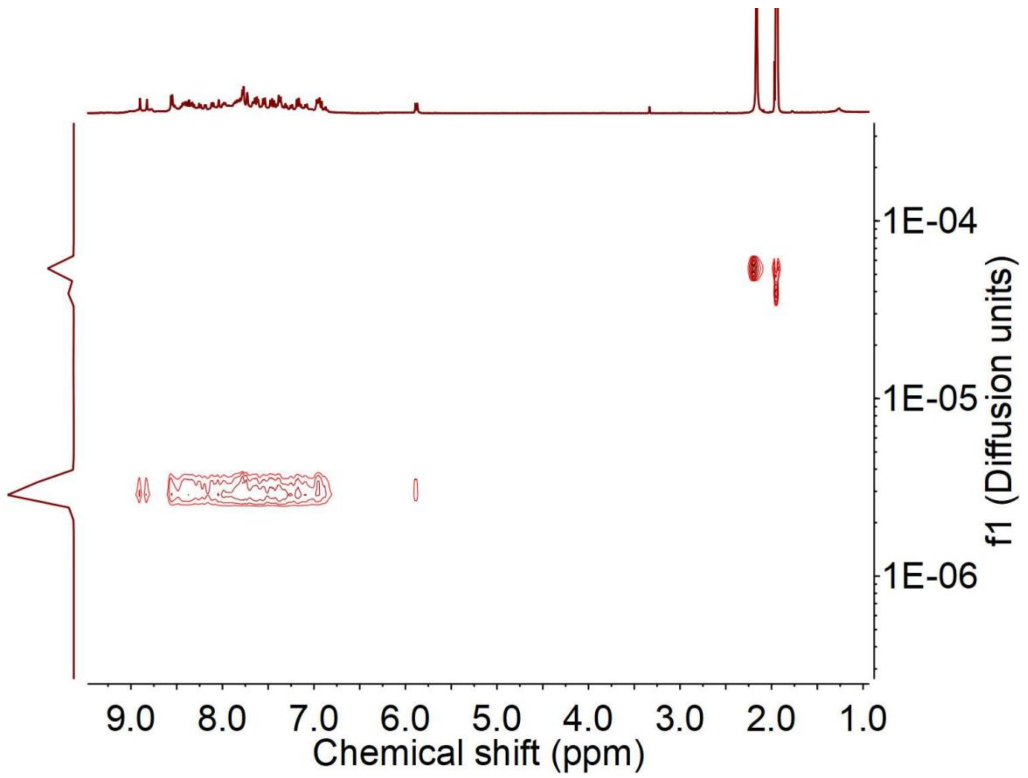

Figure S35. ^{1}H DOSY NMR spectrum of **5** (400 MHz, CD_3CN, 298 K). The diffusion coefficient for **1** in CD_3CN was measured to be 2.89 × 10^{-6} cm^2 s^{-1}, corresponding to a hydrodynamic radius of 22.6 Å.

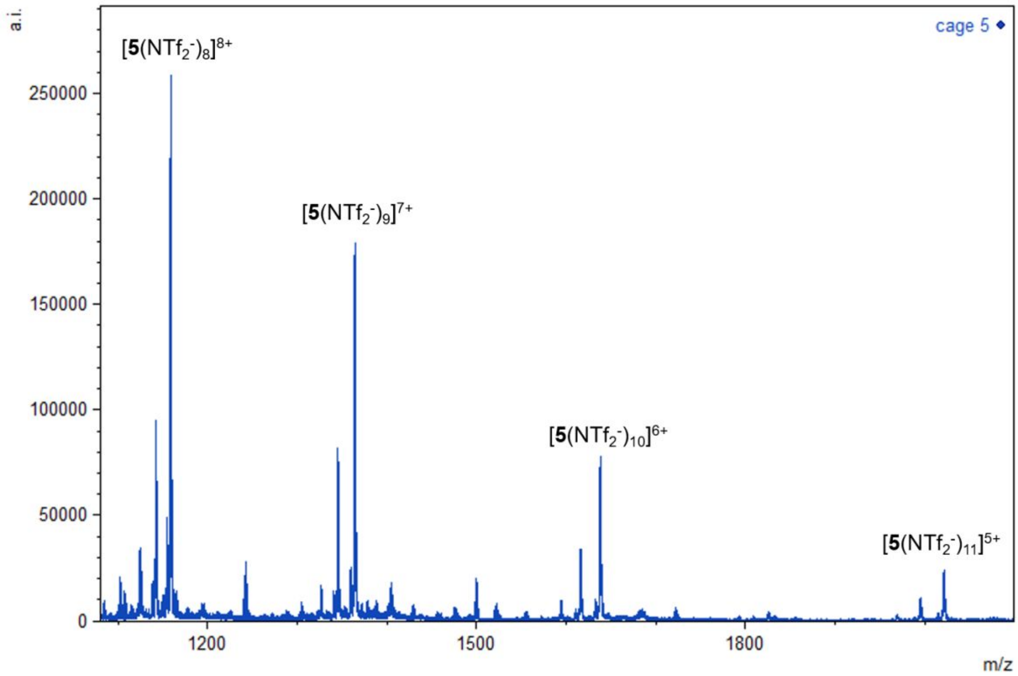

Figure S36: Low-resolution ESI-mass spectrum of **5**$(NTf_2^-)_{16}$. ESI-MS: Experimental results: *m/z* = 1157.9 [**5**$(NTf_2^-)_8$]$^{8+}$, 1363.3 [**5**$(NTf_2^-)_9$]$^{7+}$,1637.2 [**5**$(NTf_2^-)_{10}$]$^{6+}$, 2020.8 [**5**$(NTf_2^-)_{11}$]$^{5+}$. Calculated results: m/z = 1157.2 [**5**$(NTf_2^-)_8$]$^{8+}$, 1362.6 [**5**$(NTf_2^-)_9$]$^{7+}$,1636.4 [**5**$(NTf_2^-)_{10}$]$^{6+}$, 2019.7 [**5**$(NTf_2^-)_{11}$]$^{5+}$.

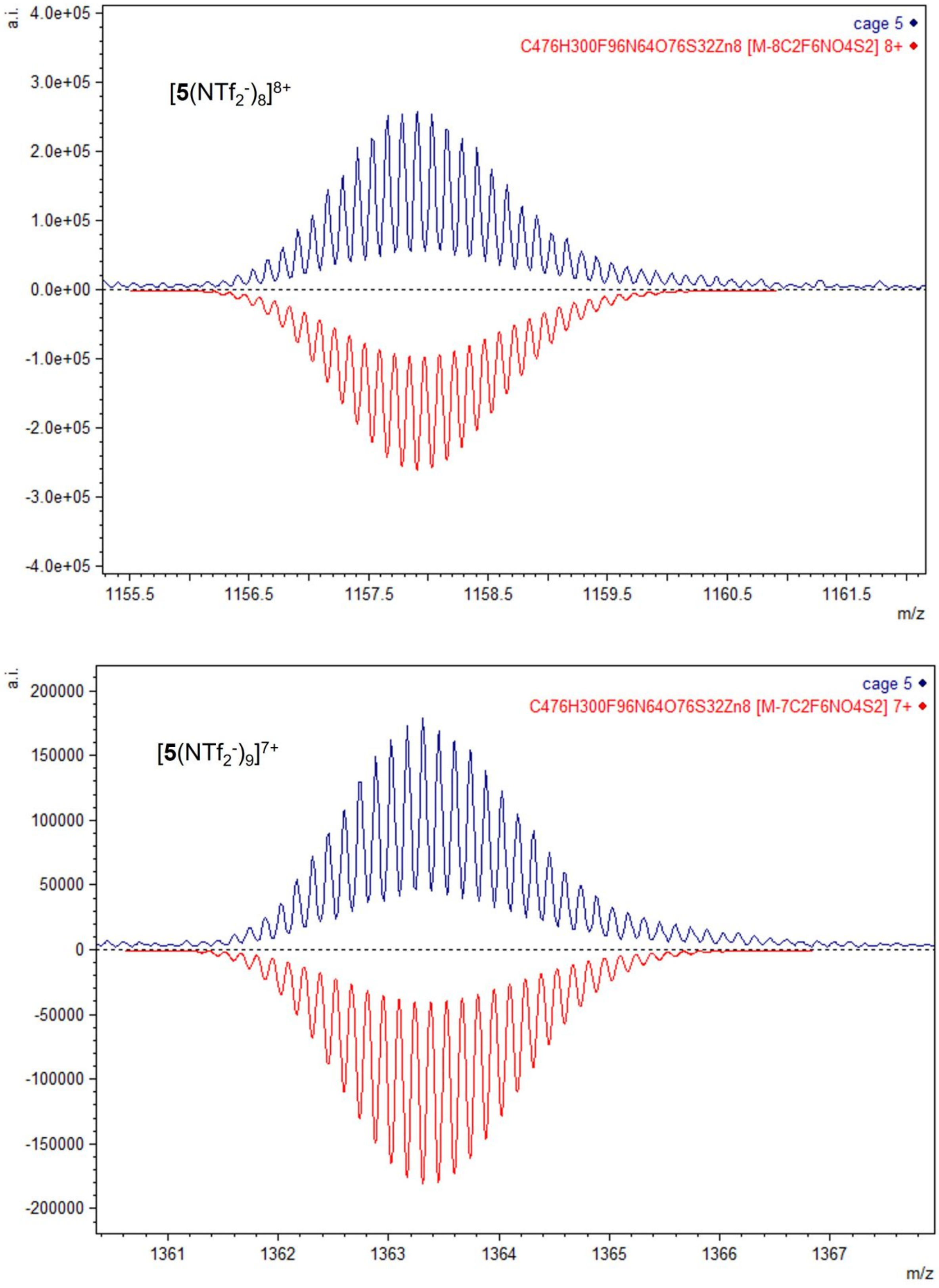

Figure S37: High-resolution ESI-mass spectrometry analysis of **5**$(NTf_2)_{16}$ showing the observed (blue) and theoretical (red) isotope patterns for the +8 and +7 peaks.

3.6 Oxidation of Subcomponent A before cage assembly

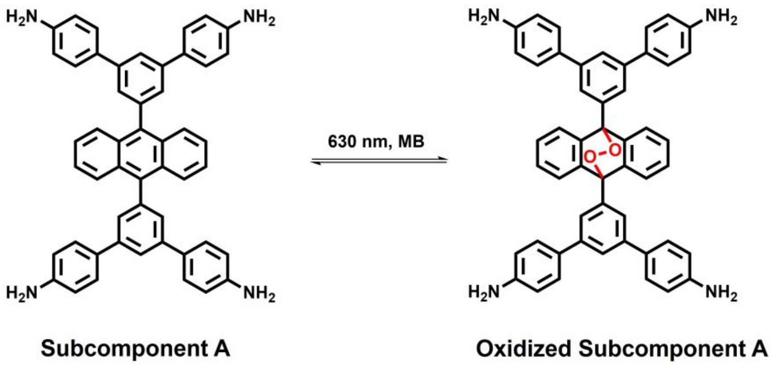

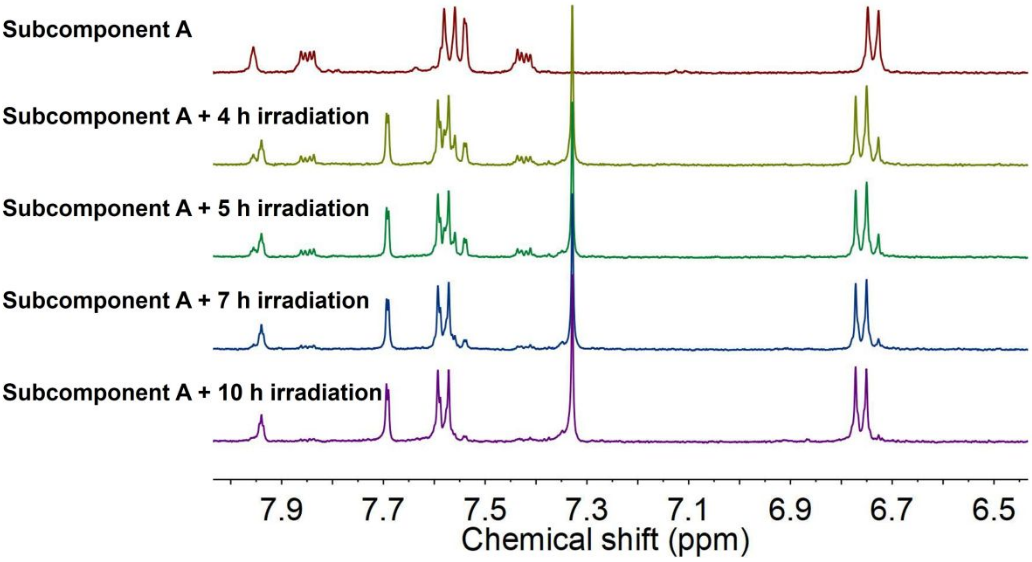

Figure S38: Partial ^1H NMR (400 MHz, CD_3CN, 298 K) spectrum of 1 mg Subcomponent **A** + methylene blue (MB, 0.05 equiv) was irradiated with red light (λ_{max} = 630 nm) for 10 h at room temperature. After 10 h irradiation, Subcomponent **A** was transferred to Oxidized Subcomponent **A**.

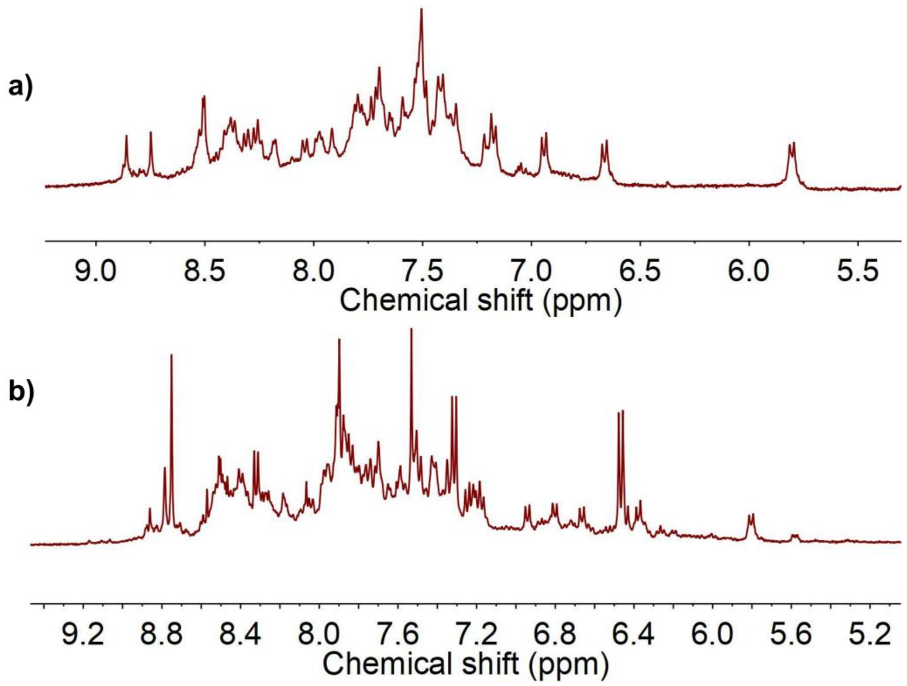

Figure S39: Partial ^{1}H NMR (400 MHz, CD_3CN, 298 K) spectra of **a)** Oxidized subcomponent **A** (1.05 mg, 1.0 equiv) + $Zn(NTf_2)_2$ (0.63 mg, 1.4 equiv) + 2-formylpyridine (0.33 mg, 4.1 equiv), after the reaction mixture was stirred at 65 °C for 12 h and washed by Et_2O. In the ^{1}H NMR, only signals from cage **1** and some broad peaks were observed, suggesting the decomposition of the oxidized subcomponent **A** during cage assembly; **b)** Oxidized subcomponent **A** (1.05 mg, 1.0 equiv), Subcomponent **B** (0.34 mg, 0.67 equiv), $Zn(NTf_2)_2$ (1.08 mg, 2.4 equiv) and 2-formylpyridine (0.58 mg, 7.2 equiv) were mixed and heated to 65 °C for 12 h and then washed with Et_2O. Only signals belonging to cage mixture **1**-**3** and some unknown broad peaks were observed, suggesting the decomposition of the oxidized subcomponent **A** during cage assembly.

4 Structural transfer among Cage 1-5

4.1 Structural transformations in Figure 3b

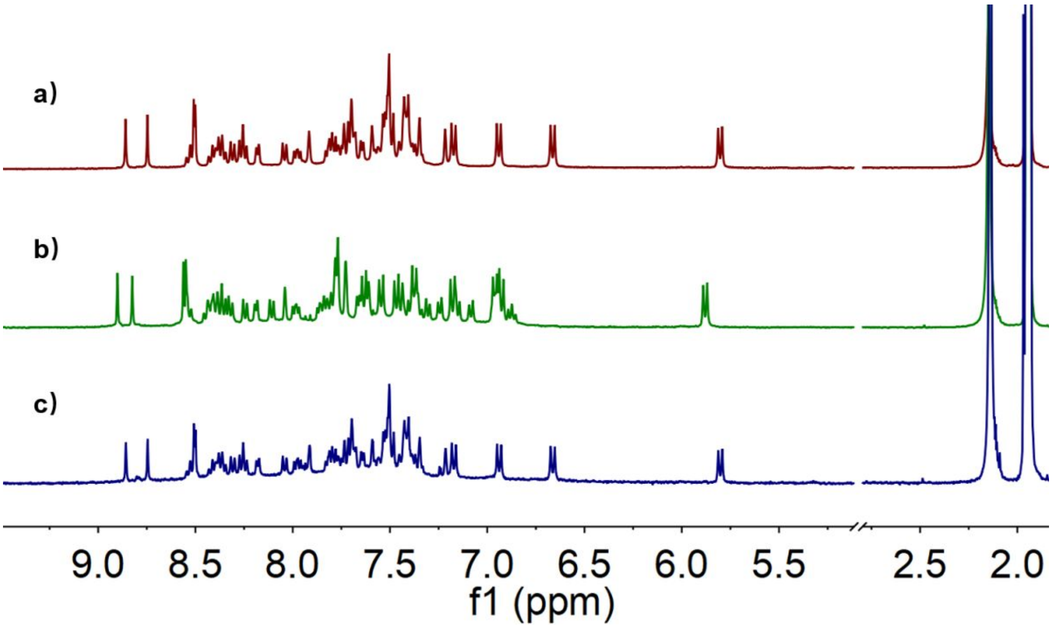

Figure S40: ^{1}H NMR (400 MHz, CD_3CN, 298 K) spectrum of **a)** 1 mM cage **1**; **b)** 1 mM cage **1** + methylene blue (MB, 0.05 equiv) was irradiated with red light (max = 630 nm) for 2h at room temperature; **c)** Sample **b)** after microwave heating at 120 °C for 2h.

4.2 Structural transformations in Figure 3c

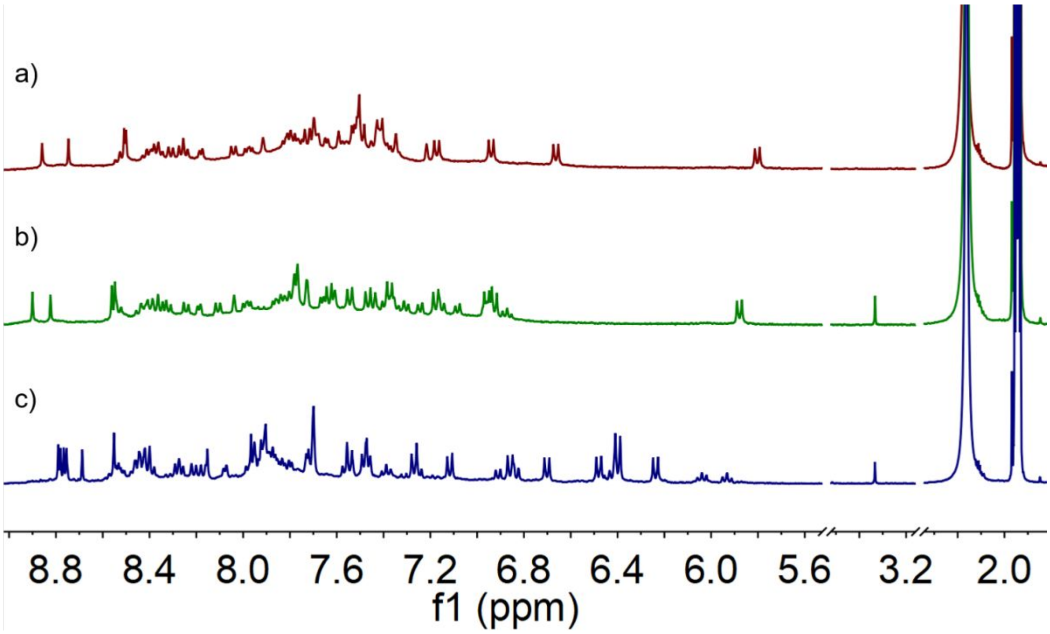

Figure S41: ^{1}H NMR (400 MHz, CD3CN, 298 K) spectrum of **a)** 0.5 mM cage **1**; **b)** 0.5 mM cage **1** + methylene blue (MB, 0.2 equiv) was irradiated with red light (max = 630 nm) for 30 min at room temperature, obtaining 0.5 mM cage **5**; **c)** 0.5 mM cage **5** + 0.5 mM cage **2** heating at 65 °C for overnight, obtaining 1 mM cage **4**.

4.3 Structural transformations in Figure 3d and Figure 3e

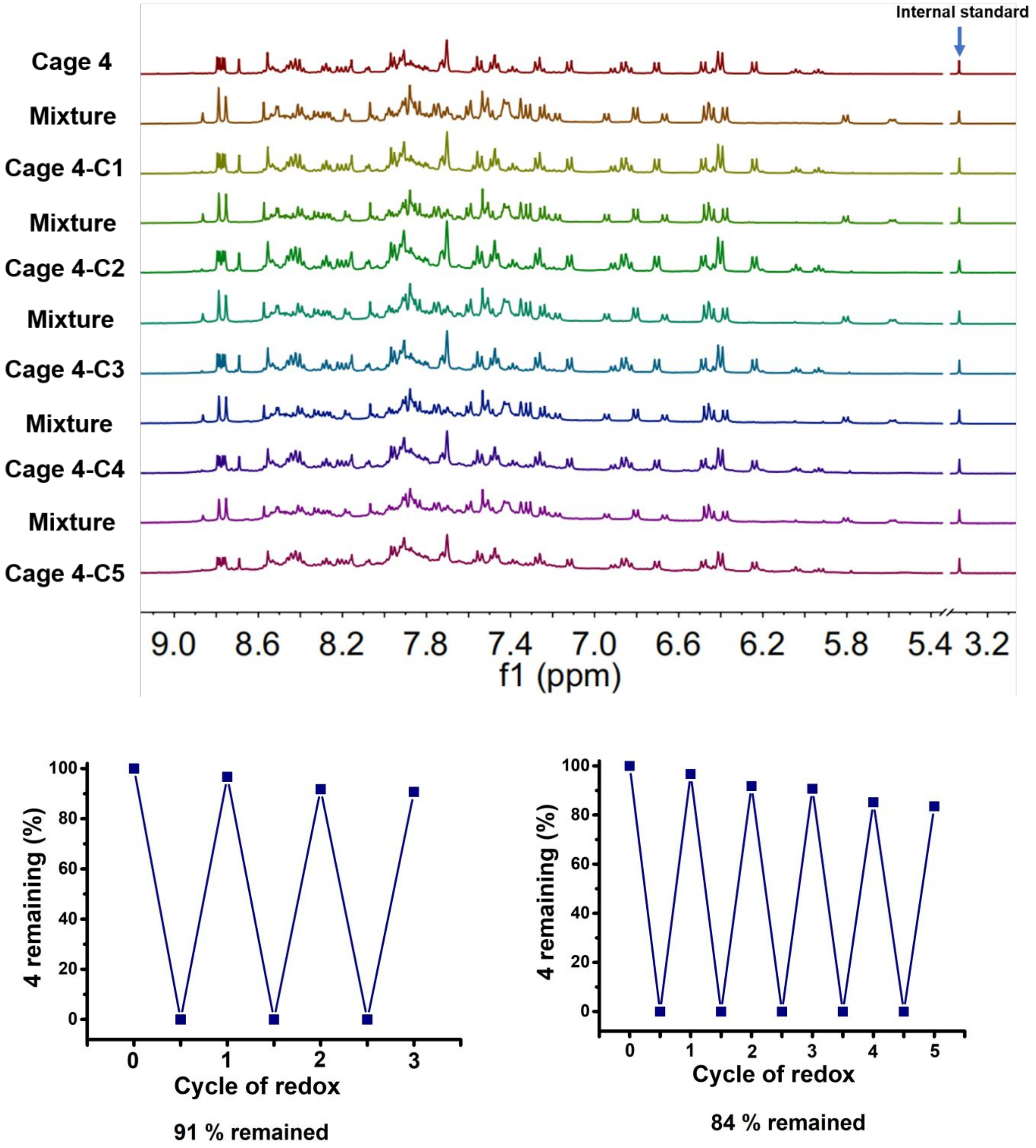

Figure S42: Partial ^{1}H NMR (400 MHz, CD3CN, 298 K) spectrum of 5 redox cycle (C1 to C5) between cage **4** (1 mM) and mixture of cage **1**, cage **2** and cage **3**. Procedure of photo-oxidation: Methylene blue (MB, 0.05 equiv) was irradiated with red light (max = 630 nm) for 2h at room temperature; Reverse procedure: Microwave 120 °C for 2h; MB was used as the internal standard for cycle rate calculation (91% remained after 3 cycles; 84% remained after 5 cycles) .

5 Variable temperature ^{1}H NMR for energy calculation

5.1 General procedures

In variable temperature ^{1}H NMR experiments of cage mixture (cage **1**, cage **2** and cage **3**) Cage **1** (2.55 mg, 1 equiv) and cage **2** (1.12 mg, 1 equiv) were mixed together in 0.45 mL CD_3CN. Samples heated at 120 °C, 90 °C and 75 °C were obtained via microwave reactor heating until equilibrium. For samples heated at 65 °C, 60 °C, 55 °C, 50 °C were obtained via 24 hours heating until the system reached equilibrium.

5.2 Variable temperature ^{1}H NMR of cage 1-3 mixture

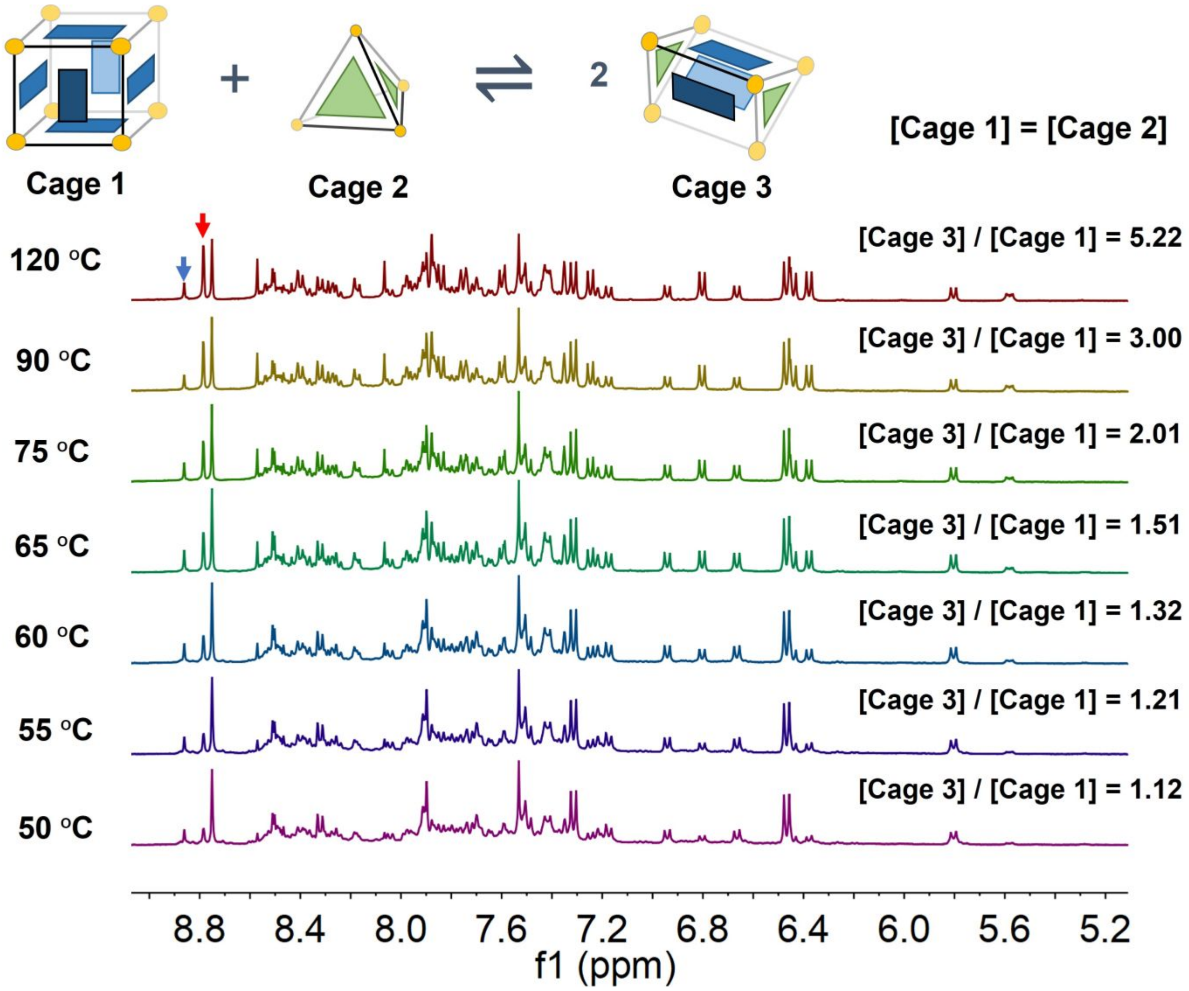

Figure S43: Partial variable temperature ^{1}H NMR (400 MHz, CD3CN, 298 K) spectra of an equilibrating mixture of cages **1**, **2** and **3** at temperatures of 120 °C, 90 °C, 75 °C, 65 °C, 60 °C, 55 °C and 50 °C. The ratio of [cage **3**] / [cage **1**] was determined by comparing the ^{1}H NMR intensities of the imine peaks labeled with a red arrow (cage **3**) and with those marked by a blue arrow (cage **1**).

a)

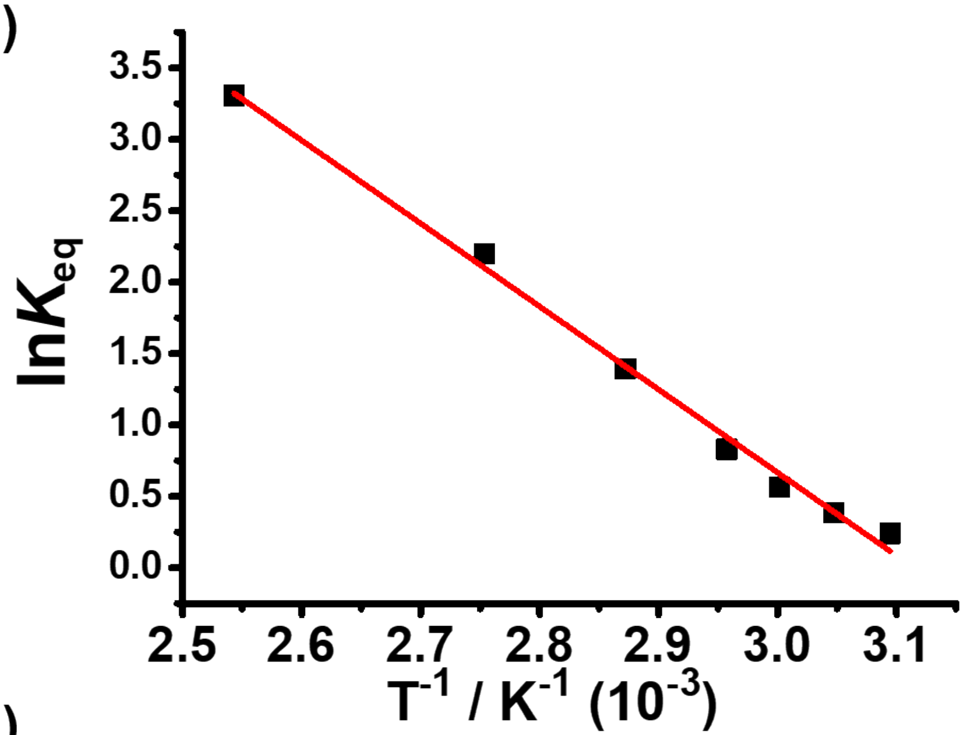

b)

$$\ln K_{eq} = \frac{-\Delta H}{RT} + \frac{\Delta S}{R}$$

Equation	y = a + b*x		
Residual Sum of Squares	0.0418		
Pearson's r	-0.99723		
Adj. R-Square	0.99336		
		Value	Standard Error
S/R	Intercept	18.11284	0.56263
-H/R	Slope	-5.81536	0.19393

$$\Delta S = R(18.11284 \pm 0.56263) = 150.59 \pm 4.68\, J/K{\cdot}mol$$

$$\Delta H = R(5.81536 \pm 0.19393) = 48.35 \pm 1.61\ kJ/mol$$

Figure S44: Entropy as a driving force for generating heteroleptic cage **3,** van't Hoff analysis of the equilibrium of reaction in Figure S43.

5.3 Variable temperature ^{1}H NMR of cage 4

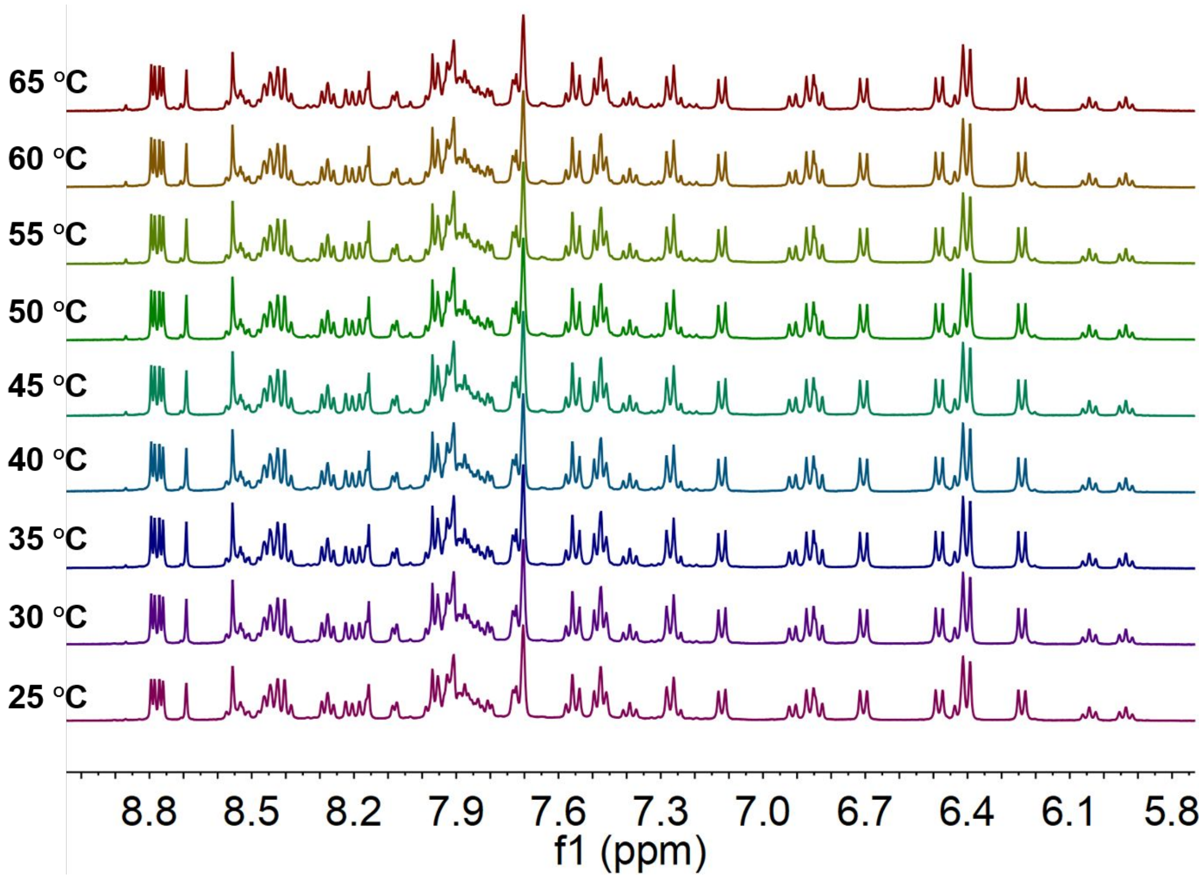

Figure S45: Partial variable temperature ^{1}H NMR (400 MHz, CD3CN, 298 K) spectrum of cage **4** (1 mM) at temperature of 65 °C, 60 °C, 55 °C, 50 °C, 45 °C, 40 °C, 35 °C, 30 °C and 25 °C.

In variable temperature ^{1}H NMR spectrum, the transition of process (iii) was found not be reversible between 25°C and 65 °C, indicating excellent thermal stability of cage **4** in process (iii).

6 Host-guest studies

6.1 General procedures

Host-guest complexes were prepared on an NMR scale and characterized by ^{1}H NMR spectroscopy. A solution of 1 mM cage sample in CD_3CN was transferred to an NMR tube and 20 mM guest molecules were added. The NMR tube was shaken and heated for 12 h at 50 °C after guest addition, and then the ^{1}H NMR spectrum, DOSY NMR spectrum, 2D ^{1}H-^{1}H NOESY NMR spectrum and ITC titrations were measured to certify the guest encapsulation.

6.2 Host-guest interactions of cage mixture with G1-G6

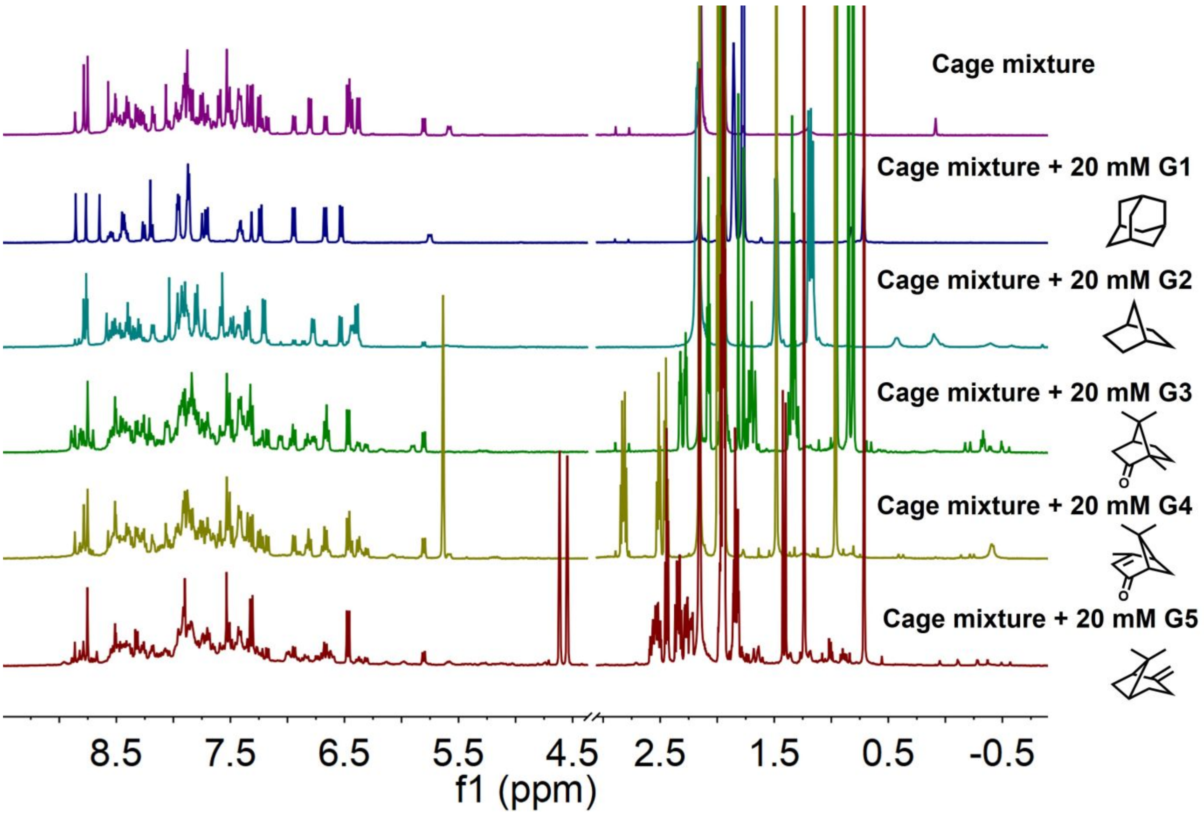

Figure S46: ^{1}H NMR (400 MHz, CD3CN, 298 K) spectrum of cage **1**-**3** mixture (cage mixture: 0.5 mM cage **1** + 0.5 mM cage **2** heating at 65 °C for 6 h) with 20 mM different guest molecules (**G1** to **G5**).

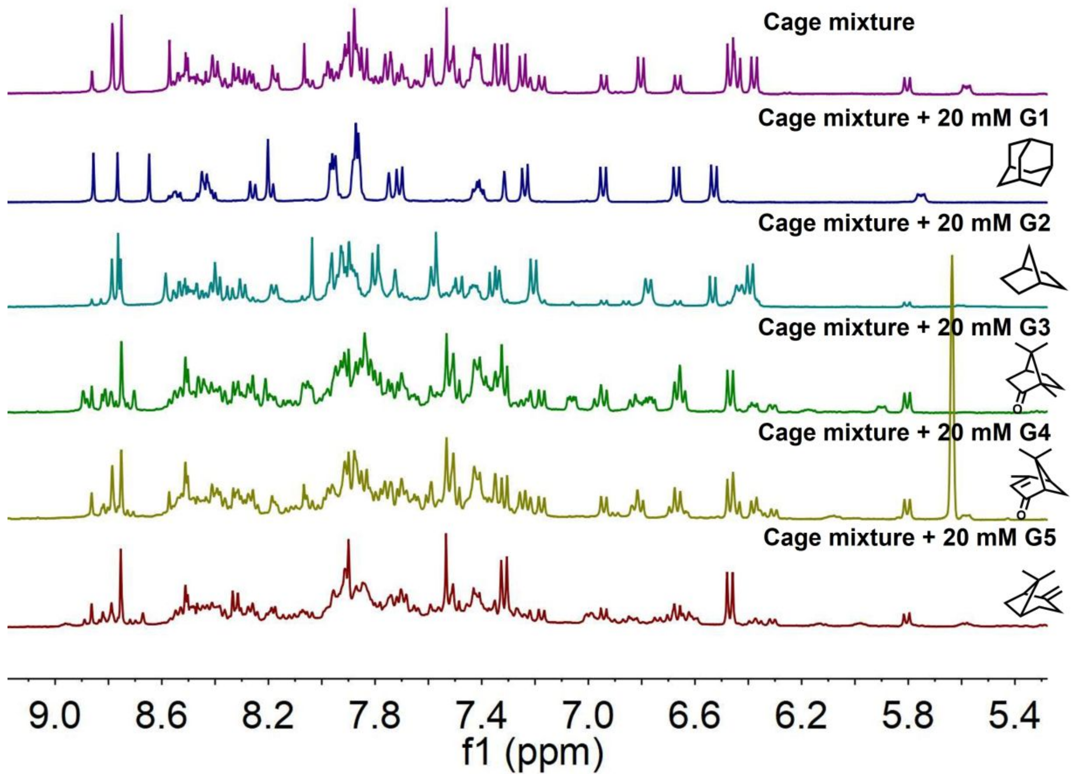

Figure S47: Aromatic region ^{1}H NMR (400 MHz, CD3CN, 298 K) spectrum of cage **1**-**3** mixture (cage mixture: 0.5 mM cage **1** + 0.5 mM cage **2** heating at 65 °C for 6 h) with 20 mM guest molecules (**G1** to **G5**).

6.2.1 Host-guest interactions of cage mixture with adamantane (G1)

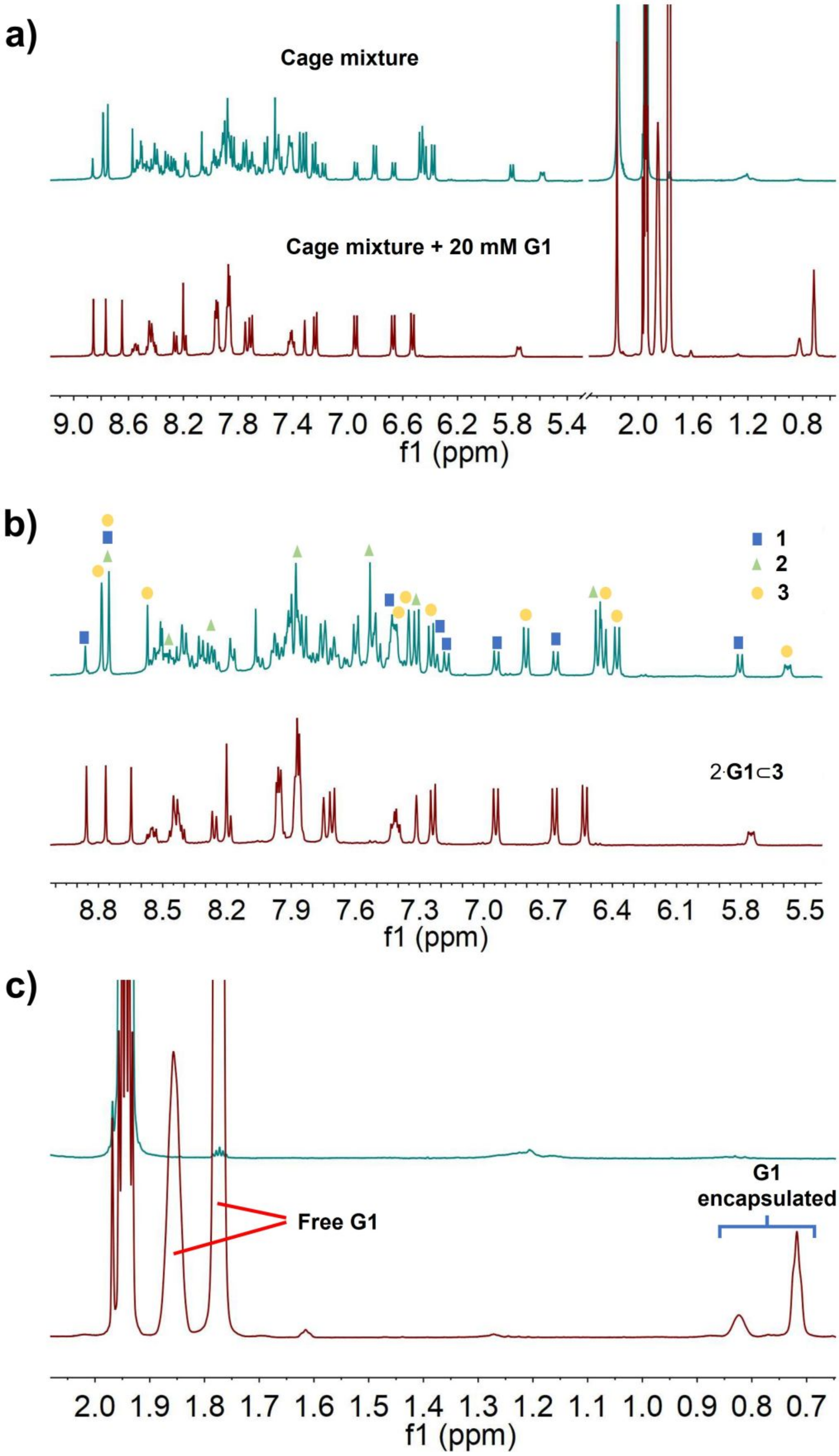

Figure S48: **a)** ^{1}H NMR (400 MHz, CD_3CN, 298 K) spectra of the mixture of cages **1**-**3** (cage mixture: 0.5 mM cage **1** + 0.5 mM cage **2**, heating at 65 °C for 6 h) and the mixture of cages **1**-**3** with 20 mM **G1**. **G1** induced conversion from cages **1**-**3** to form exclusively 2·**G1**⊂**3**; **b)** The aromatic region of the ^{1}H NMR spectrum shown in **a)**; **c)** Partial ^{1}H NMR region of **a)**. The coexistence of both free **G1** and encapsulated **G1** signals indicated the occurrence of slow-exchange host-guest binding on the NMR timescale.

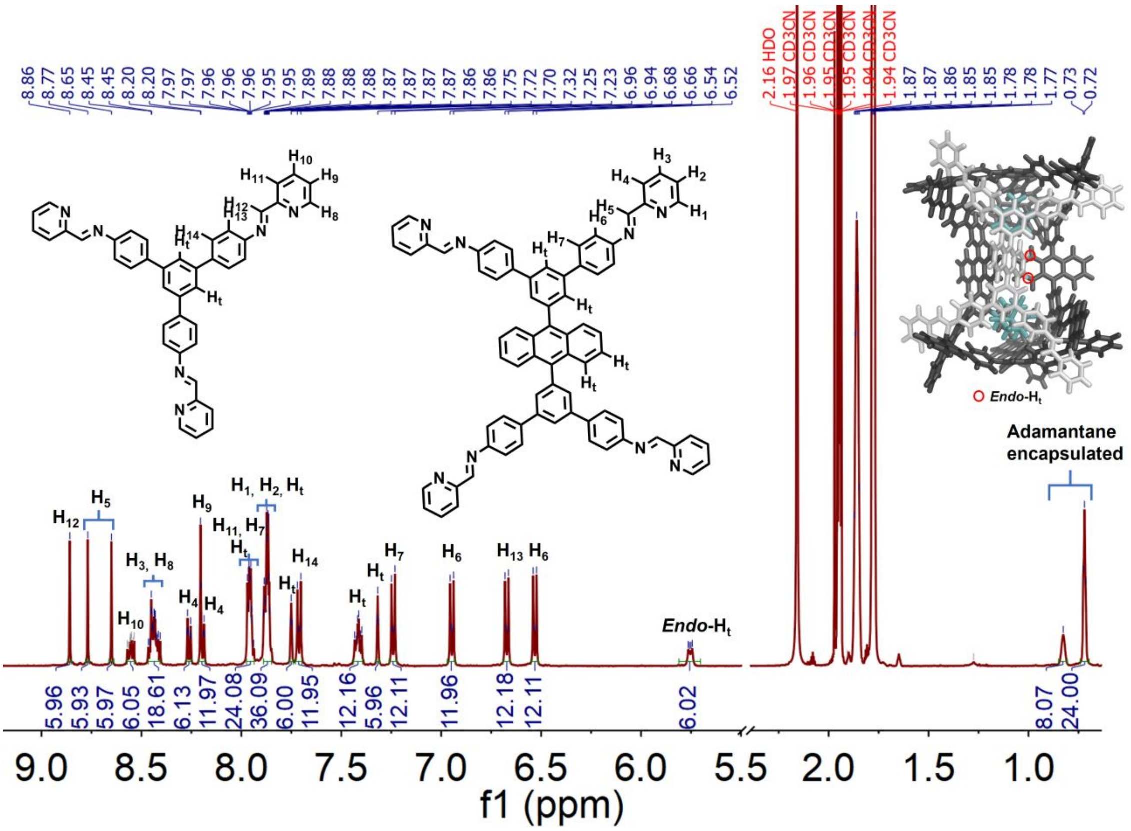

Figure S49: ^{1}H NMR (500 MHz, CD_3CN, 298 K) spectrum of 2·**G1**⊂**3** (1 mM 2·**G1**⊂**3**, 18 mM free **G1**, 20 equiv **G1** in total). **G1** induced the conversion from a cage **1**-**3** mixture to form exclusively 2·**G1**⊂**3**.

^{1}H NMR (500 MHz, CD_3CN, 298 K) δ (ppm) = 8.86 (s, 6H, H_{12}), 8.77 (s, 6H, H_5), 8.65 (s, 6H, H_5), 8.55 (ddd, J = 7.6, 6.7, 2.7 Hz, 6H, H_{10}), 8.48-8.39 (m, 18H, H_3, H_8), 8.26 (dt, J = 7.9, 1.1 Hz, 6H, H_4), 8.22-8.17 (m, 12H, H_9, H_4), 7.96 (ddt, J = 8.1, 5.1, 1.9 Hz, 24H, H_{11}, H_7, H_t), 7.87 (ddq, J = 7.3, 4.1, 1.4 Hz, 36H, H_1, H_2, H_t), 7.75 (t, J = 1.6 Hz, 6H, H_t), 7.71 (d, J = 8.6 Hz, 12H, H_{14}), 7.45-7.38 (m, 12H, H_t), 7.32 (s, 6H, H_t), 7.24 (d, J = 8.6 Hz, 12H, H_7), 6.97-6.93 (d, J = 8.6 Hz, 12H, H_6), 6.68 (d, J = 8.6 Hz, 12H, H_{13}), 6.53 (d, J = 7.0 Hz, 12H, H_6), 5.75 (dd, J = 8.6 Hz, 6H, *Endo*-H_t) 0.83 (s, 12H, **G1** encapsulated), 0.73 (s, 24H, **G1** encapsulated).

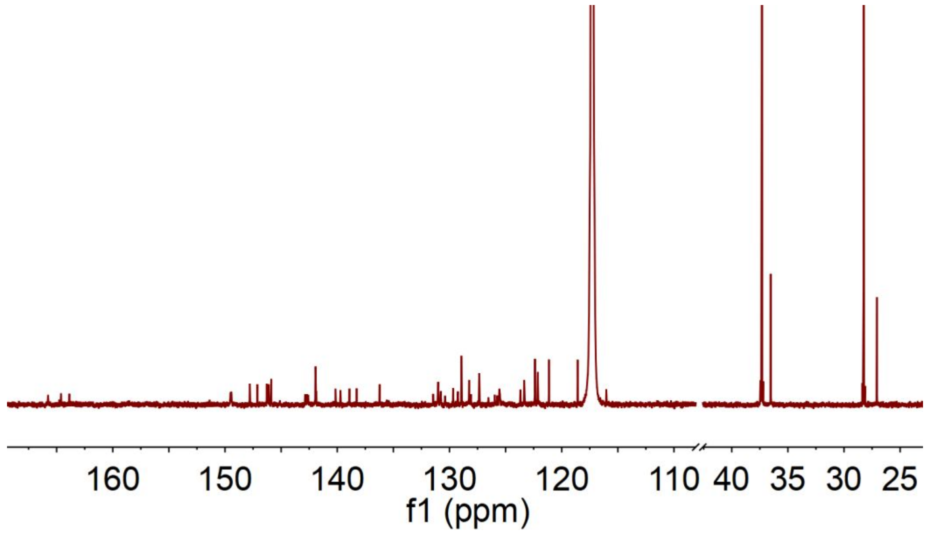

Figure S50: ^{13}C NMR spectrum of 2·**G1⊂3** (126 MHz, CD_3CN, 298 K).

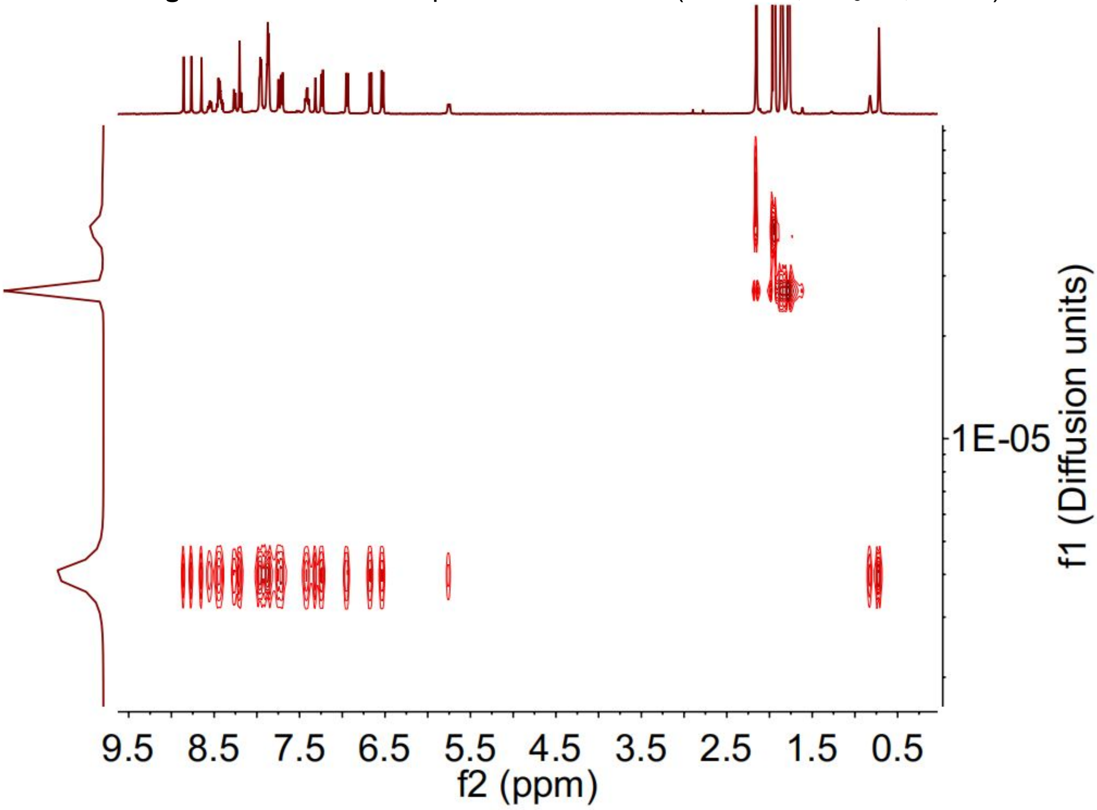

Figure S51: ^{1}H DOSY NMR spectrum of 2·**G1⊂3** (400 MHz, CD_3CN, 298 K). The diffusion coefficient for 2·**G1⊂3** in CD_3CN was measured to be 3.90×10^{-6} cm^2 s^{-1},corresponding to a hydrodynamic radius of 16.8 Å.

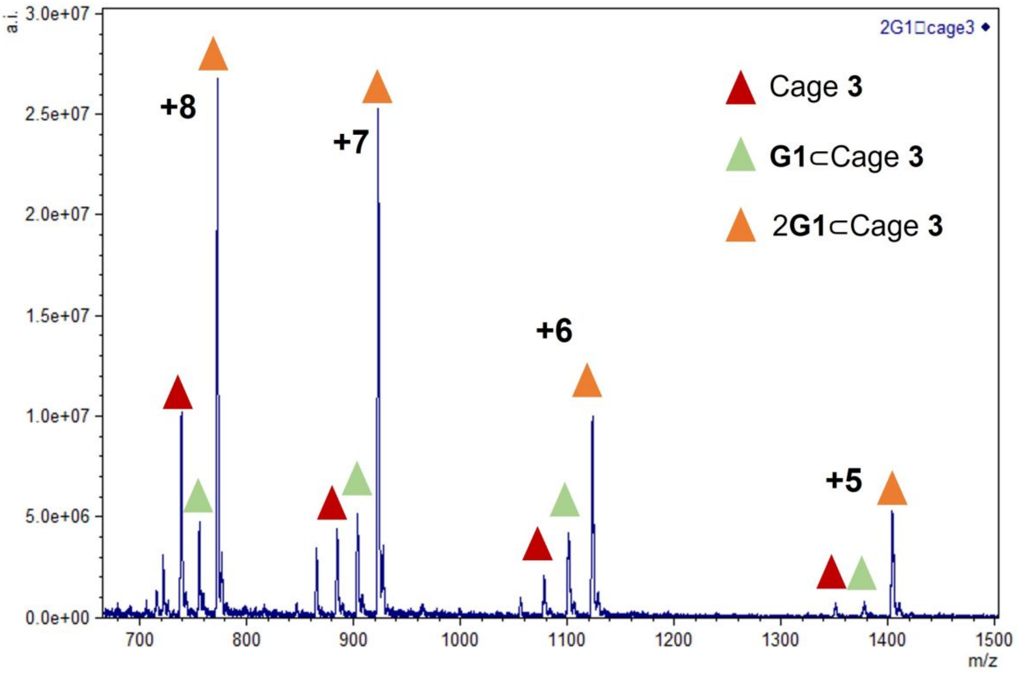

Figure S52: Low-resolution ESI-MS spectrum of 2·**G1**⊂cage **3** in MeCN

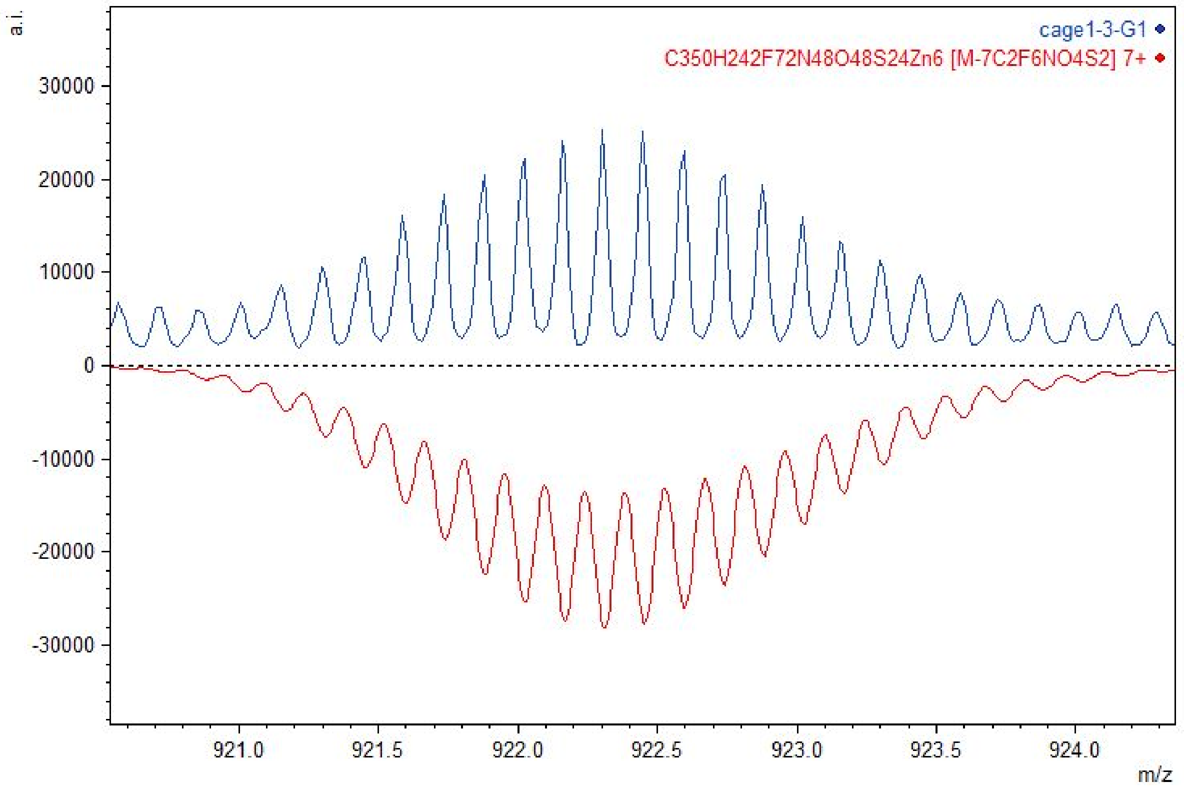

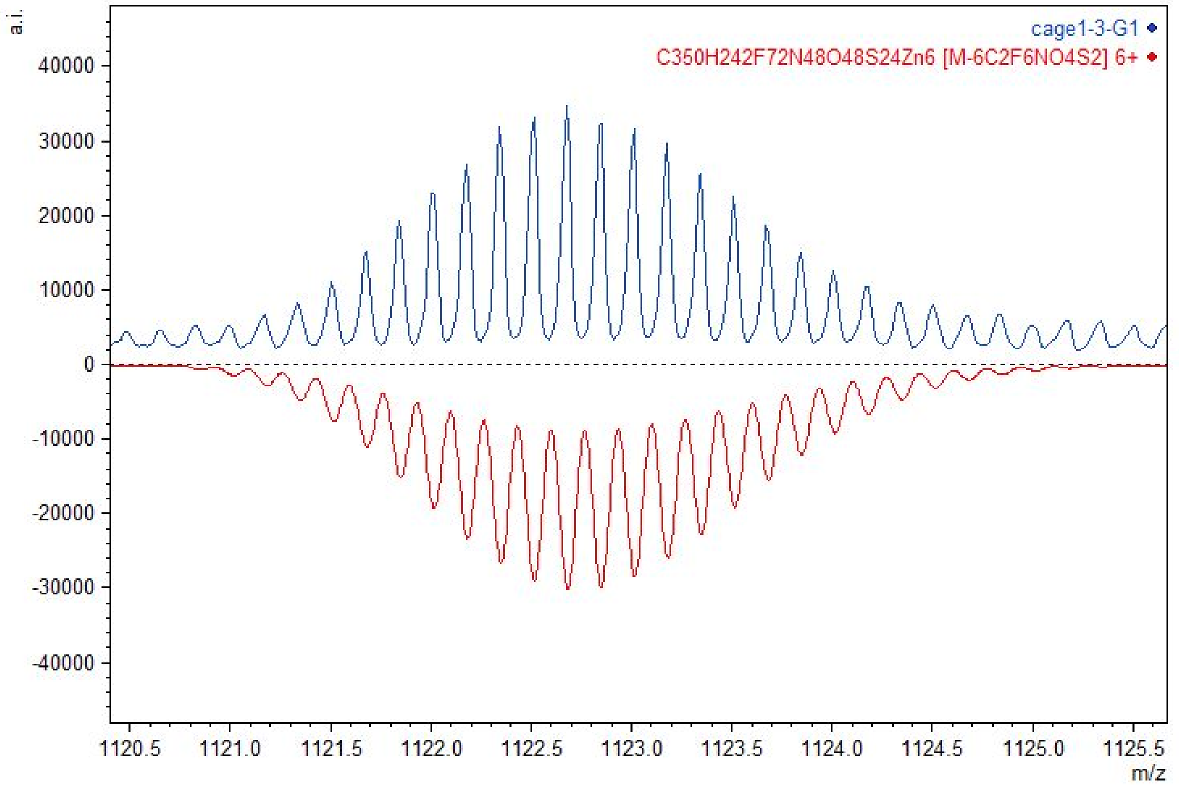

Figure S53: High-resolution ESI-mass spectrometric analysis of host-guest complex 2·**G1**⊂cage **3** showing the observed (blue) and theoretical (red) isotope patterns for the +7 and +6 peaks.

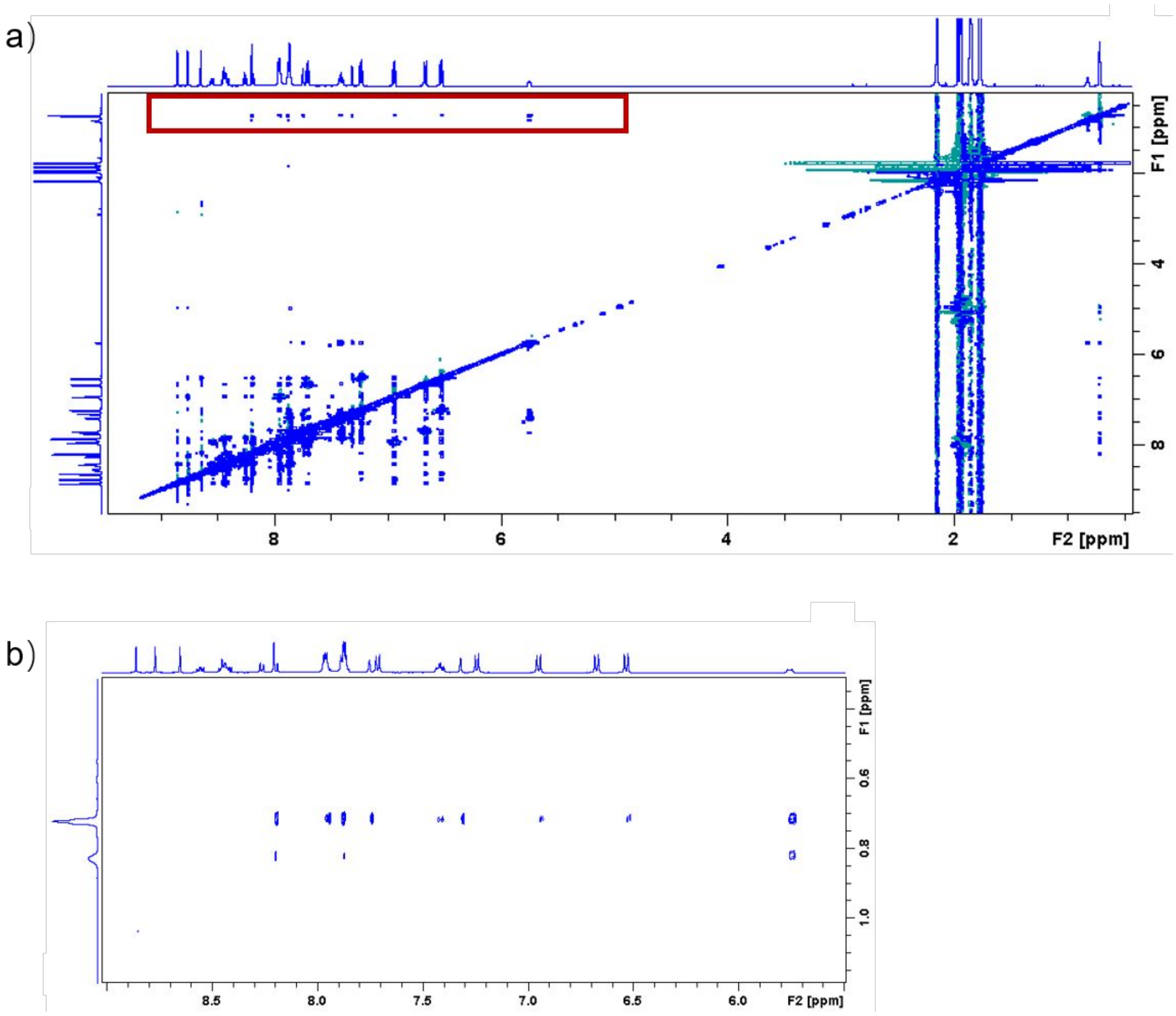

Figure S54: **a)** ^{1}H-^{1}H NOESY NMR spectrum of 2·**G1**⊂**3** (400 MHz, CD_3CN, 298 K); **b)** Partial ^{1}H-^{1}H NOESY NMR spectrum of 2·**G1**⊂**3.**

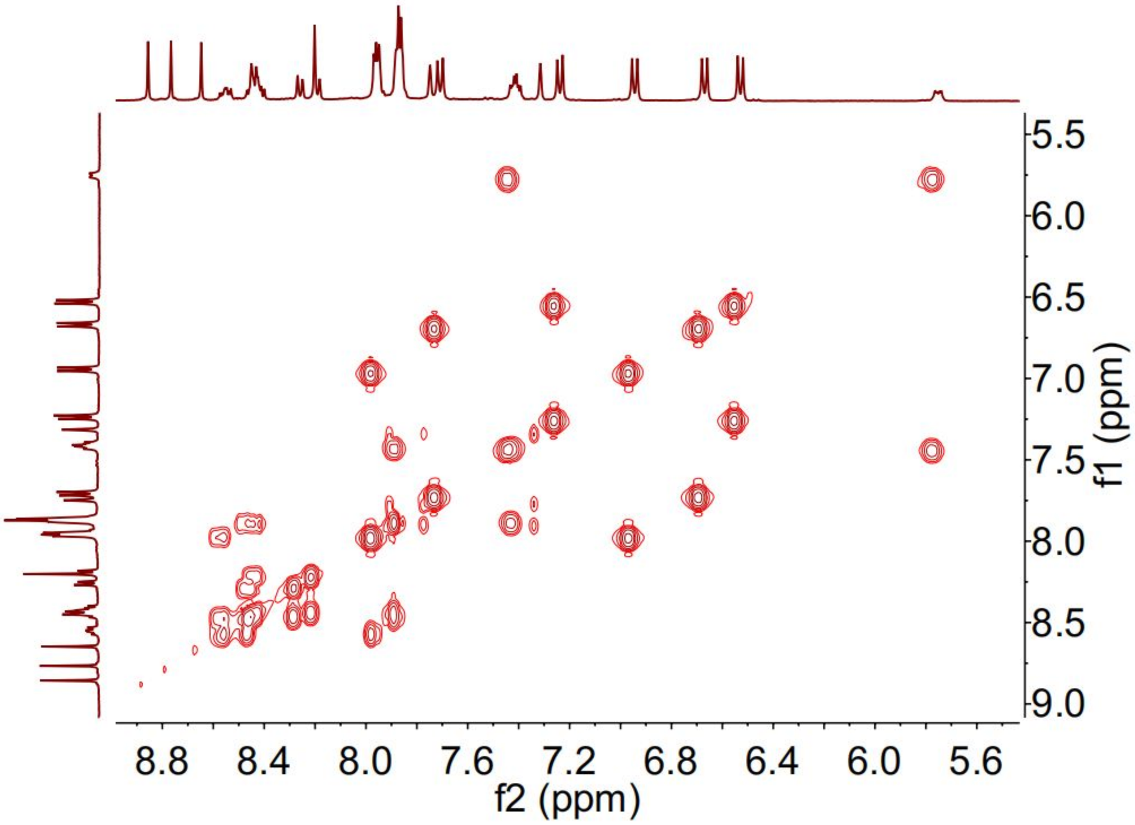

Figure S55: Aromatic region of the ^{1}H-^{1}H COSY NMR spectrum of 2·**G1**⊂**3** (400 MHz, CD_3CN, 298 K)

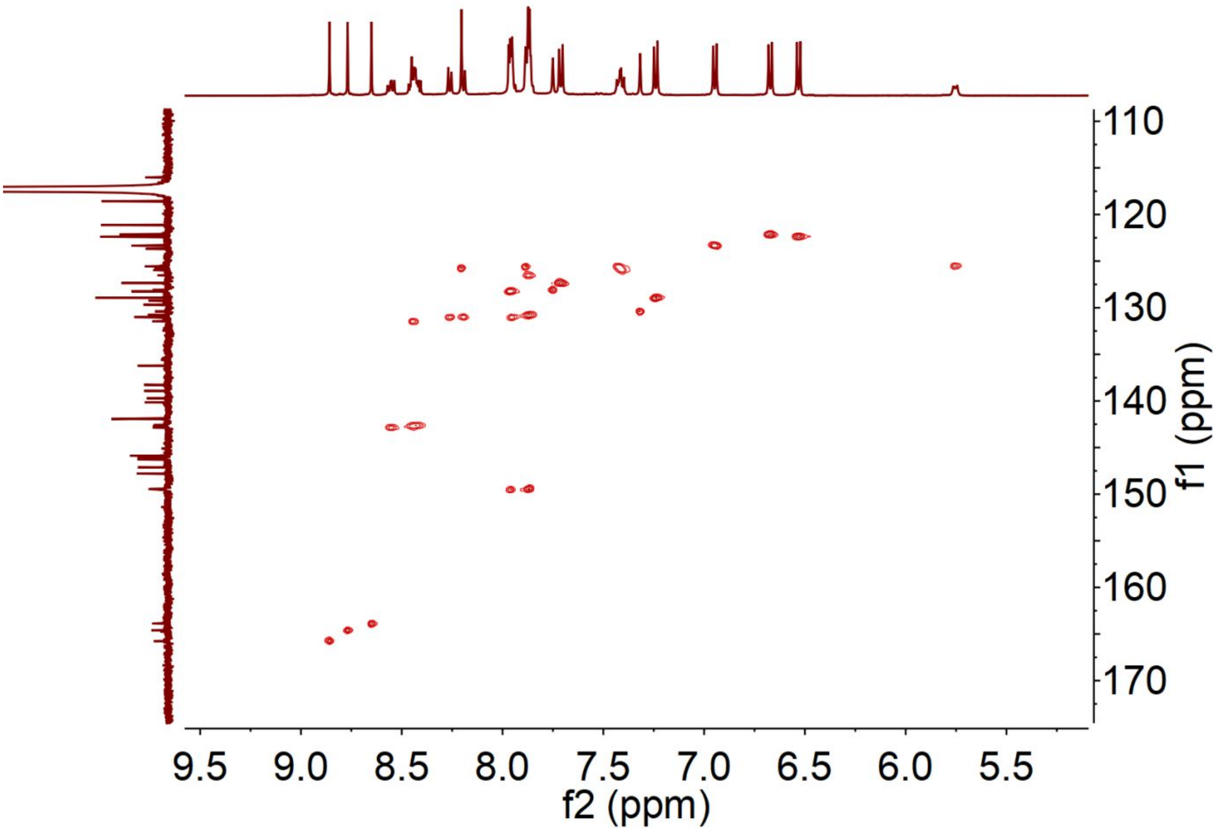

Figure S56: Aromatic region of the ^{1}H-^{13}C HSQC NMR spectrum of 2·**G1**⊂**3** (400 MHz, CD_3CN, 298 K).

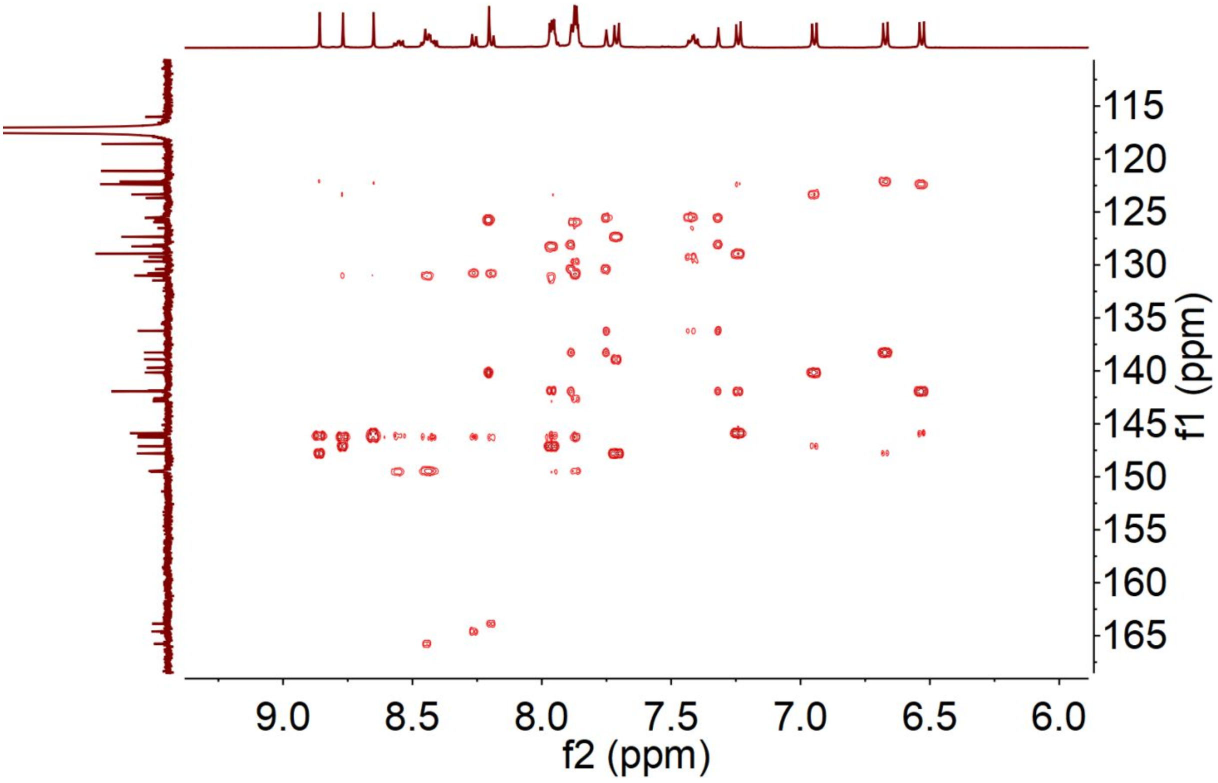

Figure S57: Aromatic region of the ^{1}H-^{13}C HMBC NMR spectrum of 2·**G1**⊂**3** (400 MHz, CD_3CN, 298 K).

6.2.2 Host-guest interactions of cage mixture with norbornane (G2)

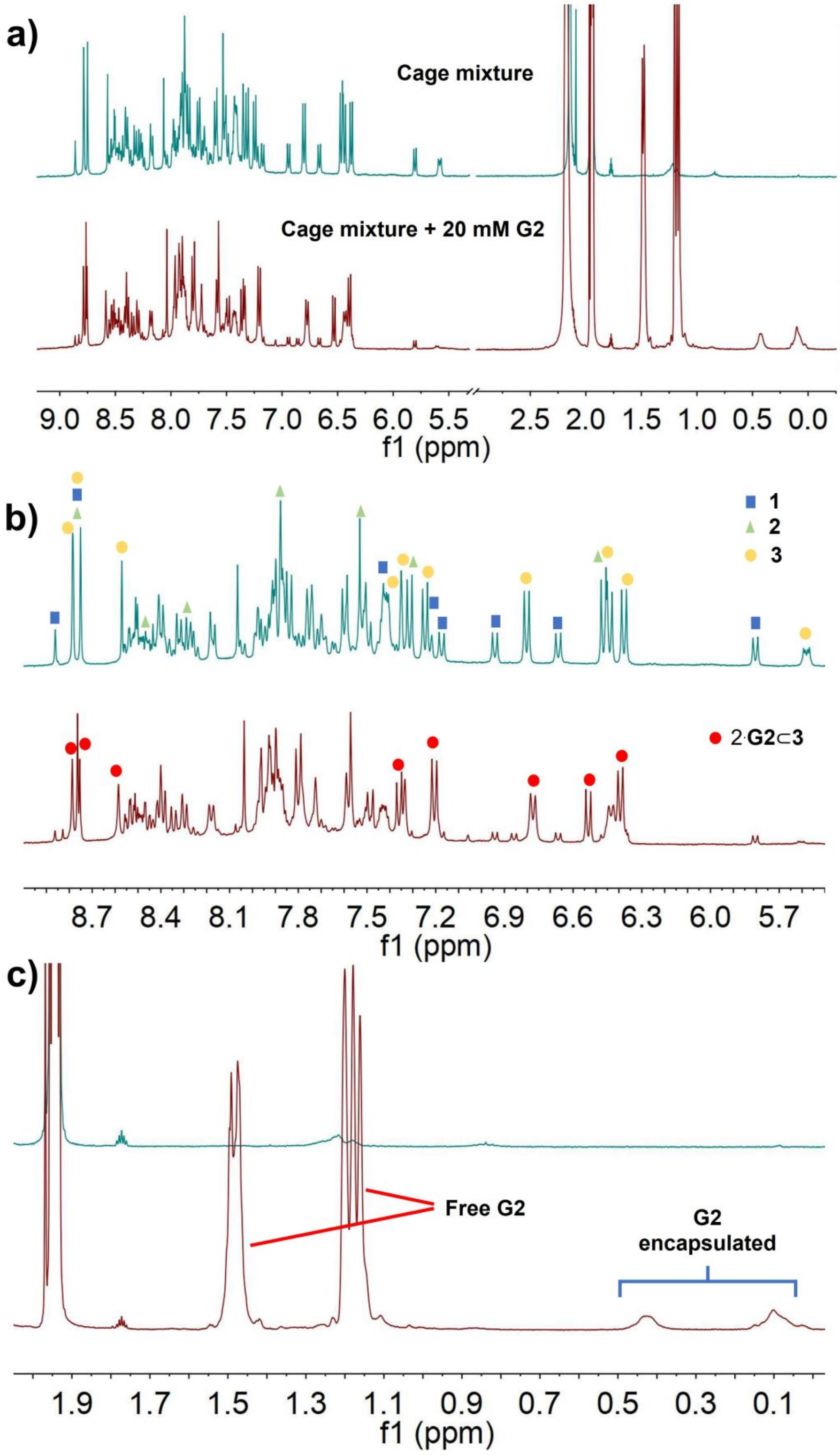

Figure S58: **a)** ^{1}H NMR (400 MHz, CD_3CN, 298 K) spectrum of the mixture of cages **1**-**3** and this mixture containing 20 mM **G2**. **G2** induced partial conversion from the mixture of cages **1**-**3** to form 2·**G2⊂3** and **G2⊂3**; **b)** The aromatic ^{1}H NMR region of **a)**, the small peaks are attributed to **G2⊂3**; **c)** Expansion of the ^{1}H NMR spectrum of **a)**. The coexistence of both free **G2** and encapsulated **G2** signals indicated slow-exchange host-guest binding on the NMR timescale.

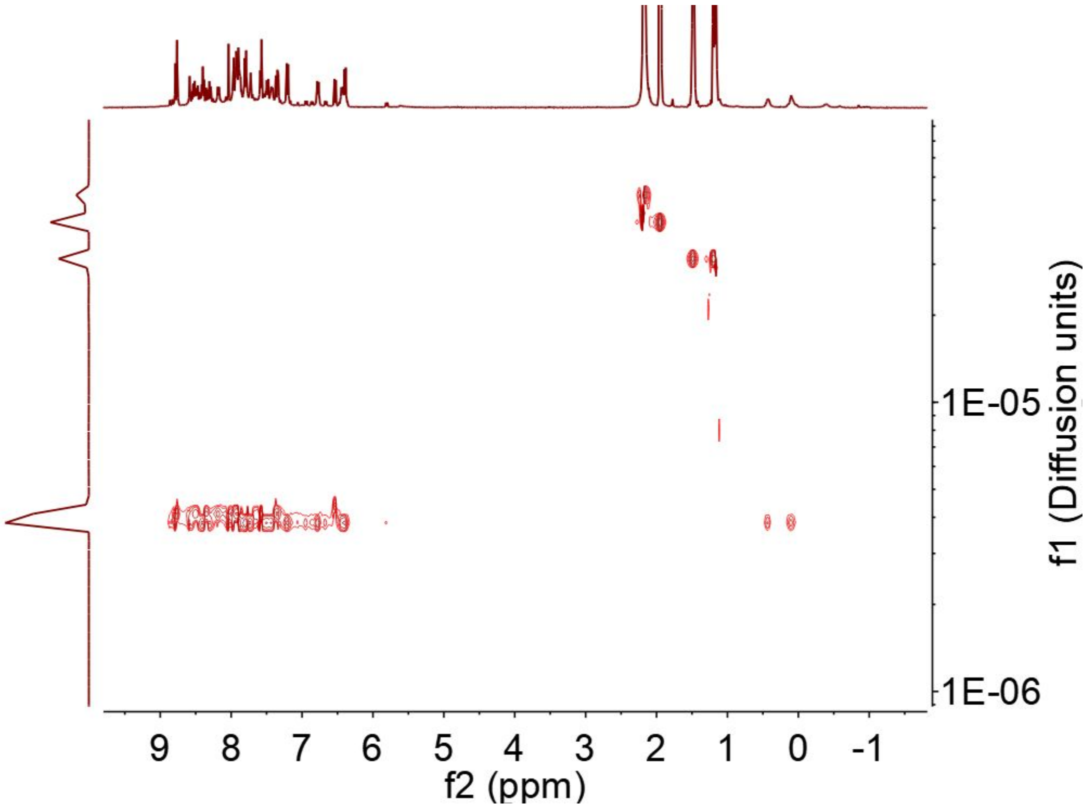

Figure S59: ^{1}H DOSY NMR spectrum of 2·**G2**⊂**3** (400 MHz, CD_3CN, 298 K). The proton signals belong to encapsulated guest could be observed.

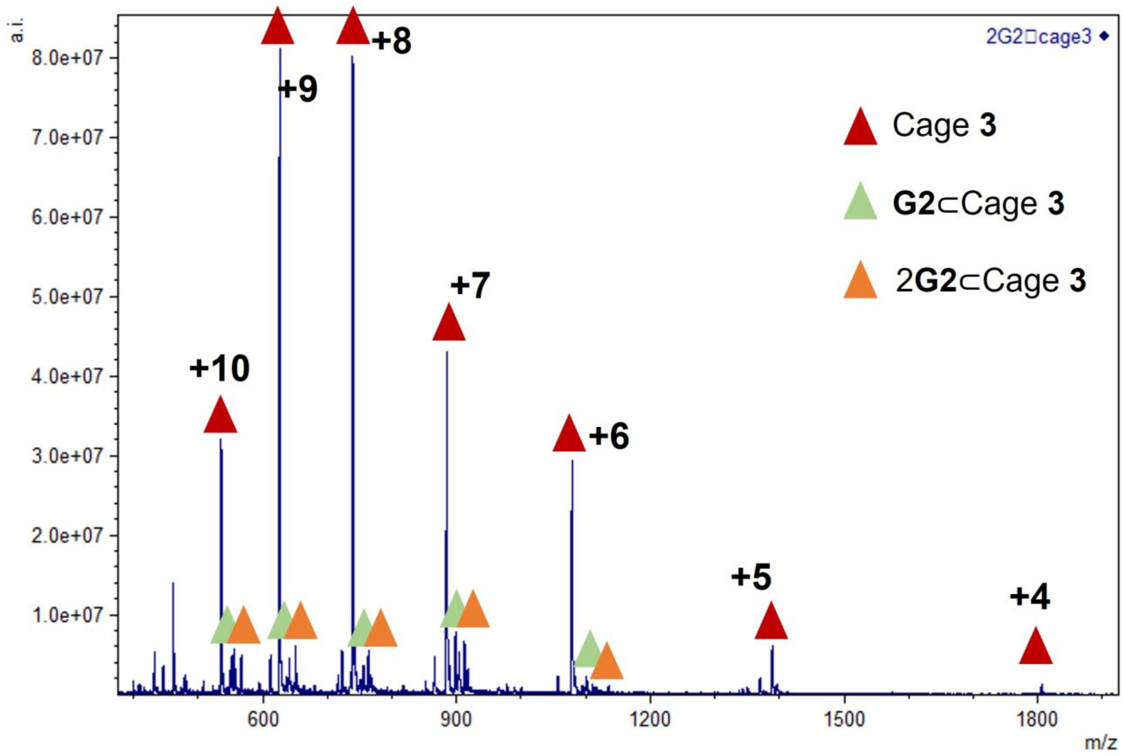

Figure S60: Low-resolution ESI-MS spectrum of cage mixture **1-3** with **G2** in MeCN

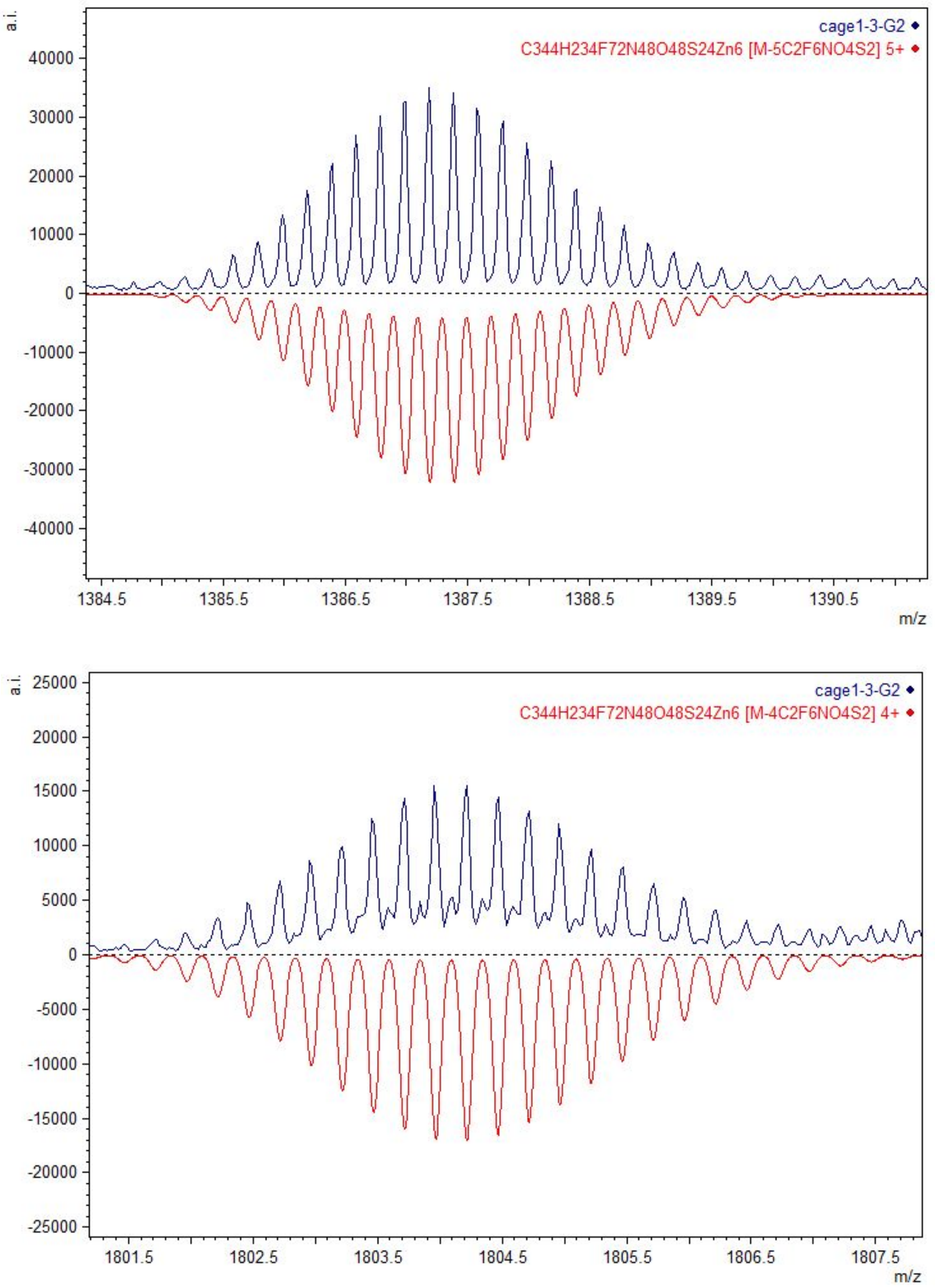

Figure S61: High-resolution ESI-mass spectrometric analysis of host-guest complex 2·**G2**⊂cage **3** showing the observed (blue) and theoretical (red) isotope patterns for the +5 and +4 peaks

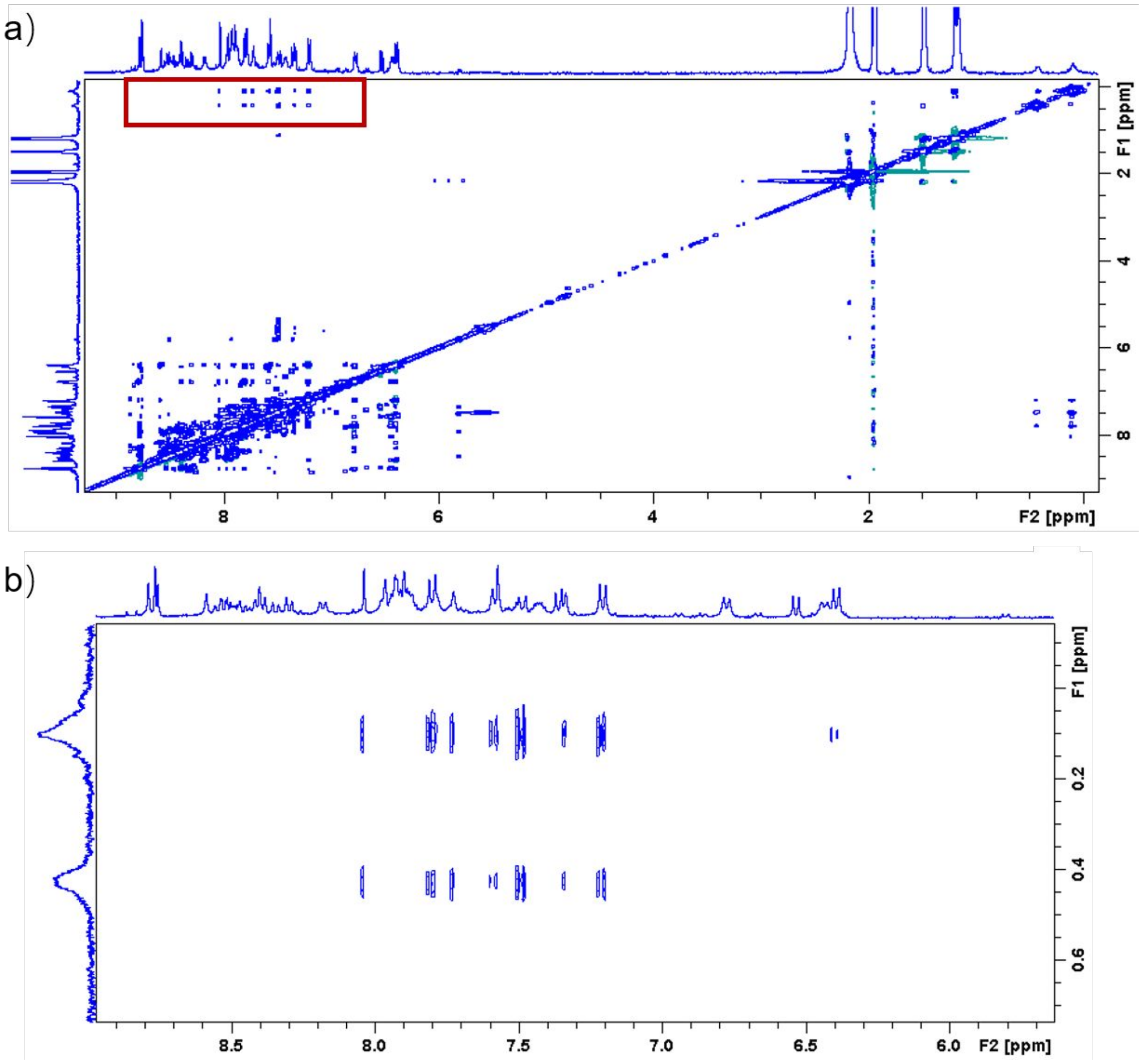

Figure S62: **a)** ^{1}H-^{1}H NOESY NMR spectrum of 2·**G2**⊂**3** (400 MHz, CD_3CN, 298 K); **b)** Partial $^{1}H\{^{1}H\}$ NOESY NMR spectrum of 2·**G2**⊂**3.**

6.2.3 Host-guest interactions of cage mixture with (1*S*)-(-)-camphor (G3)

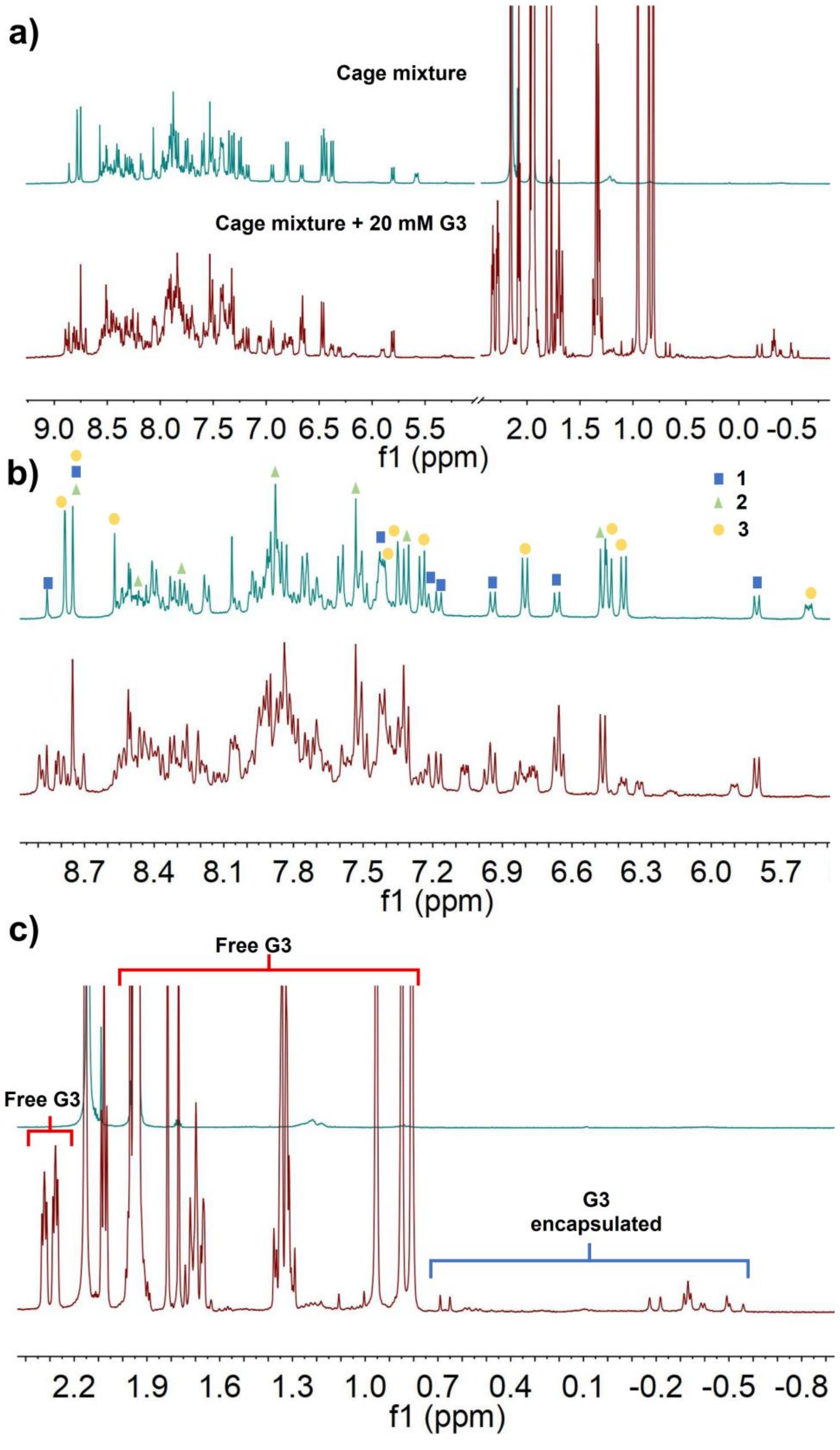

Figure S63: **a)** ^{1}H NMR (400 MHz, CD_3CN, 298 K) spectra of the mixture of cages **1**-**3**, and the same mixture containing 20 mM **G3**. **G3** induced partial conversion from the mixture of cages **1**-**3** to form 2·**G3**⊂**3** and **G3**⊂**3**; **b)** In the aromatic ^{1}H NMR region of **a)**, the disappearance of signals corresponding to empty cage **3** was observed, indicating host-guest binding between **G3** and **3**. The formation of desymmetrized **G3**⊂**3** gave a complex spectrum with multiple low-intensity peaks; **c)** Zoom in of the ^{1}H NMR spectrum shown in **a)**. The observation of signals corresponding to both free **G3** and encapsulated **G3** indicated slow-exchange host-guest binding on the NMR timescale.

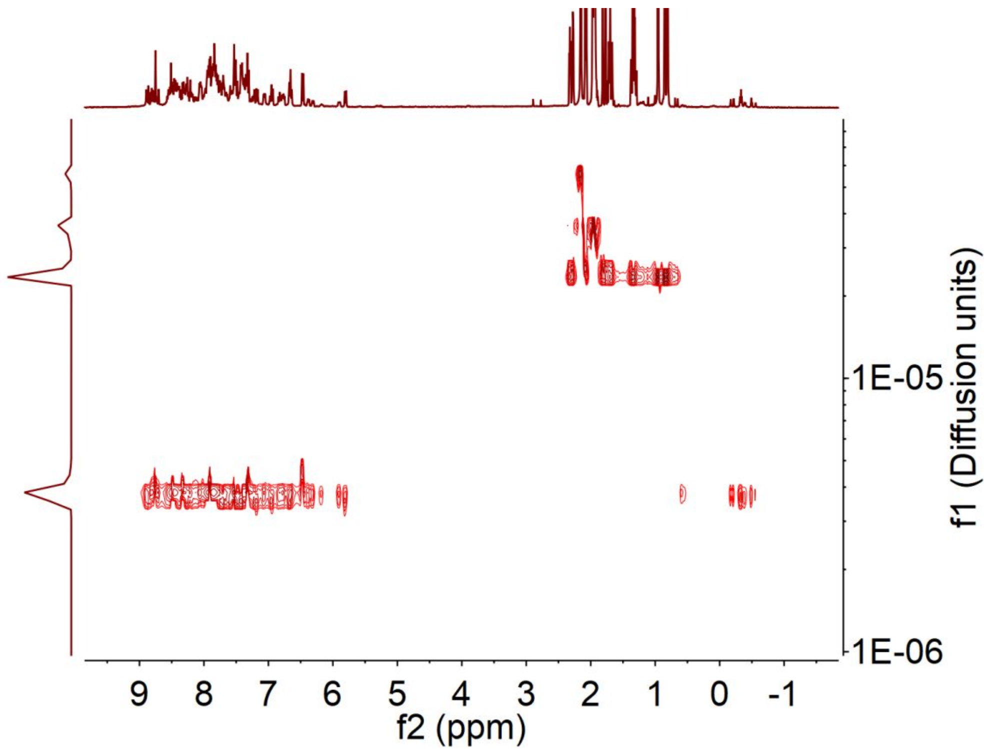

Figure S64: ^{1}H DOSY NMR spectrum of 2·**G3**⊂**3** (400 MHz, CD_3CN, 298 K). The proton signals belong to encapsulated guest could be observed.

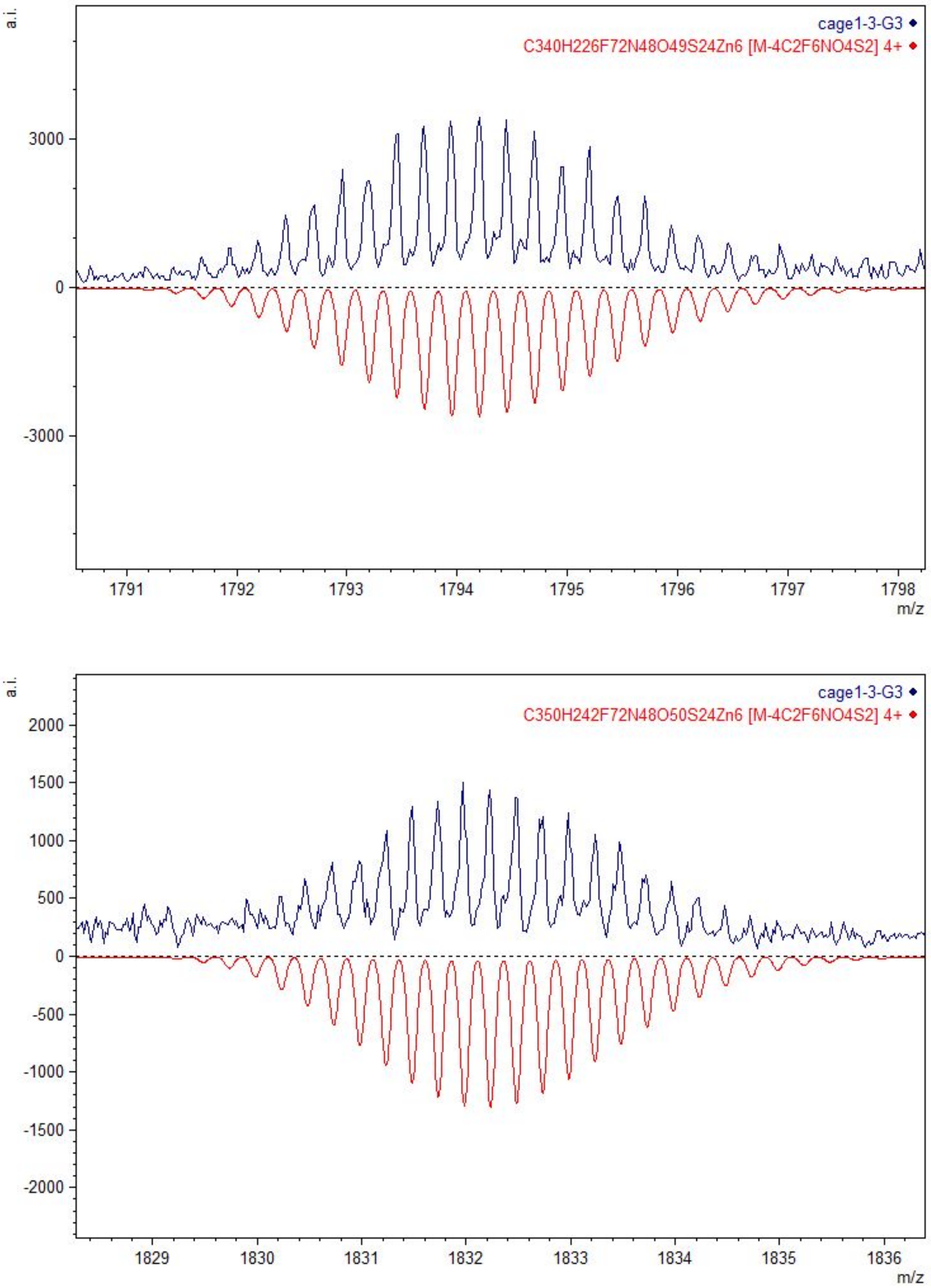

Figure S65: High resolution ESI-MS spectrum of of host-guest complex **G3**⊂cage **3** (top) and 2·**G2**⊂cage **3** (bottom) showing the observed (blue) and theoretical (red) isotope patterns for the +4 peaks.

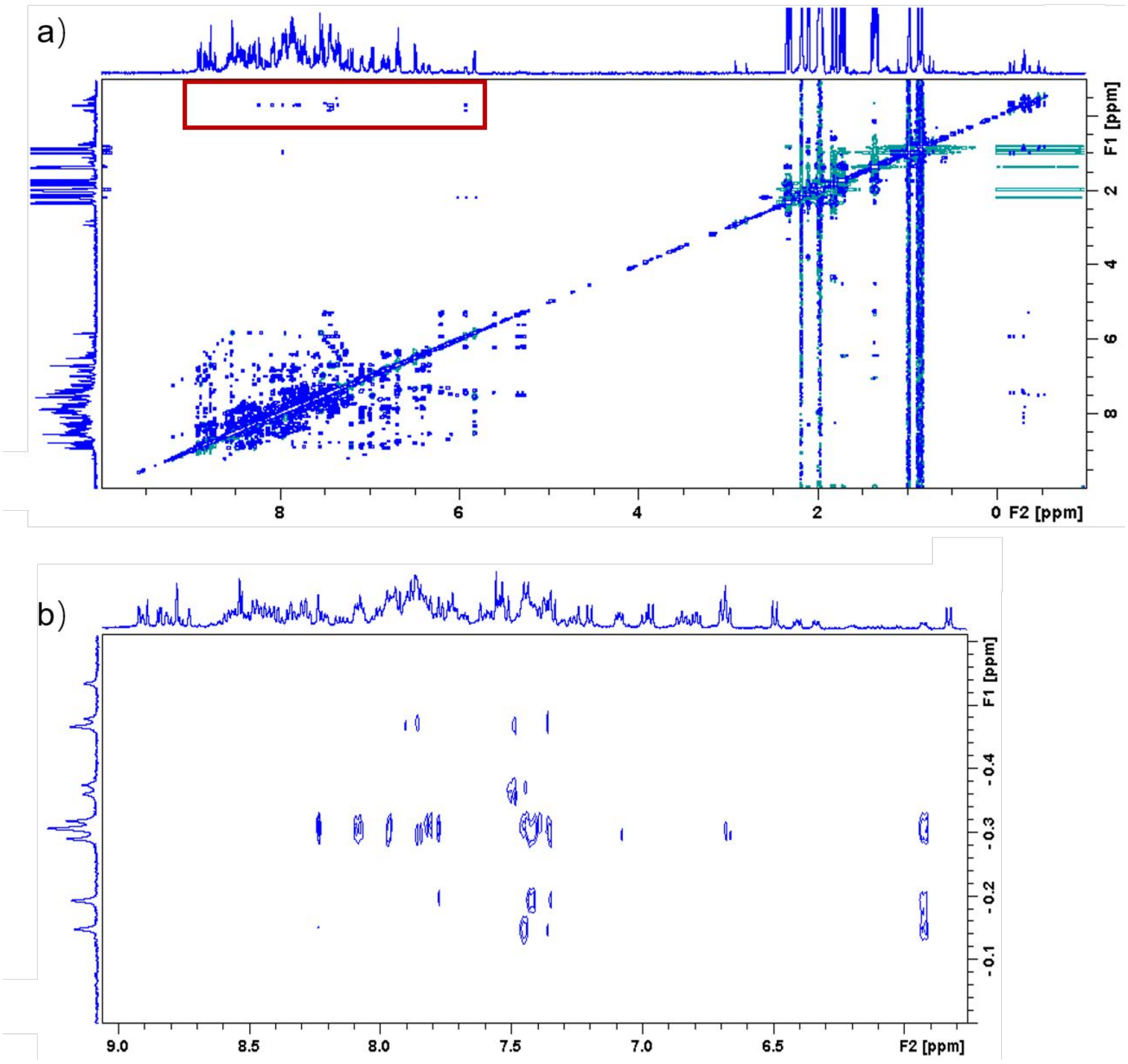

Figure S66: **a)** ^{1}H-^{1}H NOESY NMR spectrum of 2·**G3**⊂**3** (400 MHz, CD_3CN, 298 K); **b)** Partial ^{1}H-^{1}H NOESY NMR spectrum of 2·**G3**⊂**3.**

6.2.4 Host-guest interactions of cage mixture with verbenone (G4)

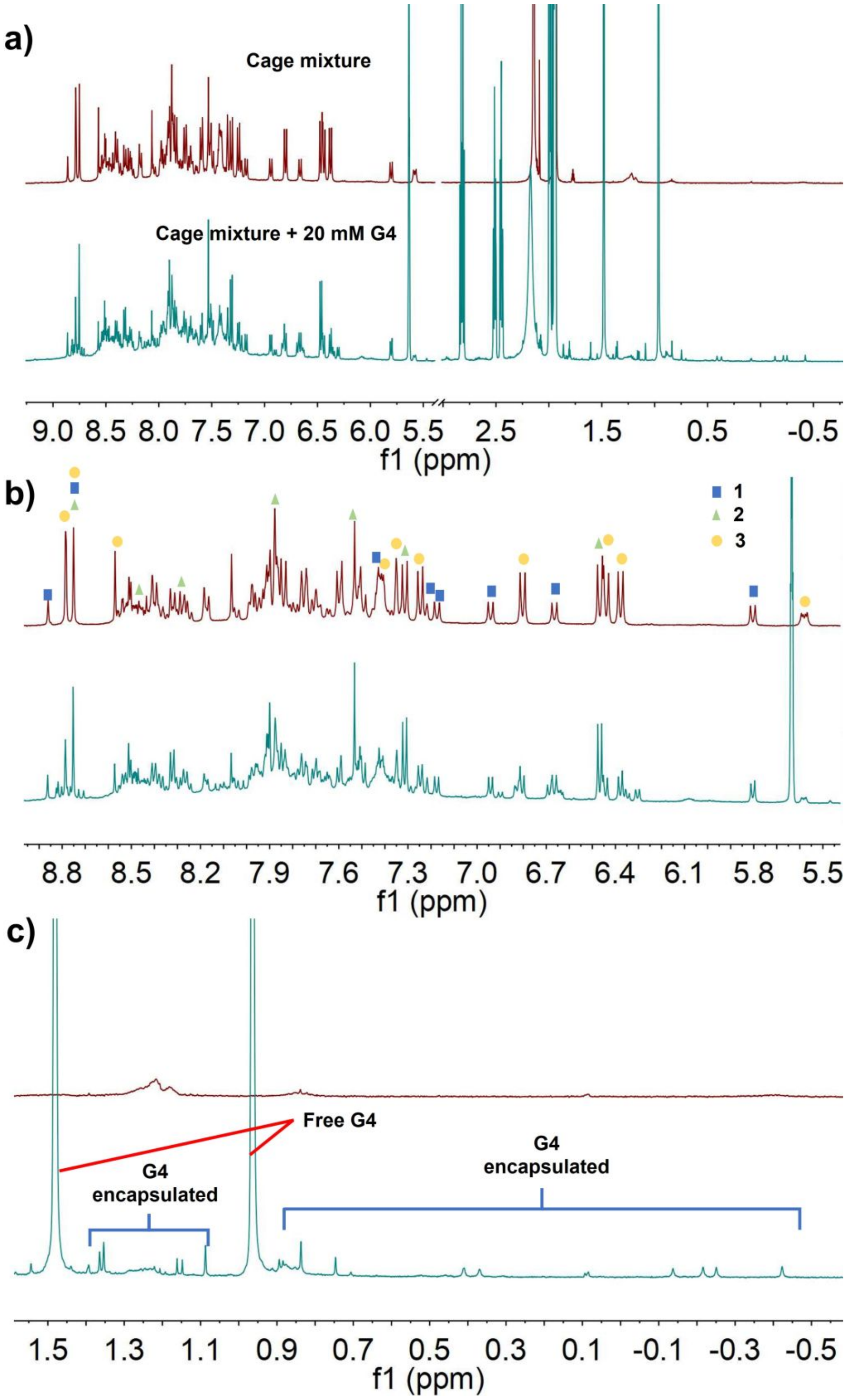

Figure S67: **a)** ^{1}H NMR (400 MHz, CD_3CN, 298 K) spectra of the mixture of cages **1**-**3** and the mixture of cages **1**-**3** with 20 mM **G4**. **G4** was encapsulated within **3** to form **G4**⊂**3**; **b)** The aromatic region of the ^{1}H NMR spectrum shown in **a)**, showing a decrease in the intensity of empty cage **3** signals, indicating host-guest binding between **G4** and **3**. The formation of the desymmetrized **G4**⊂**3** complex led to a complex spectrum with multiple low-intensity peaks **c)** Zoom in of the ^{1}H NMR spectrum shown in **a)**. The observation of signals corresponding to both free **G4** and encapsulated **G4** indicated slow-exchange host-guest binding on the NMR timescale.

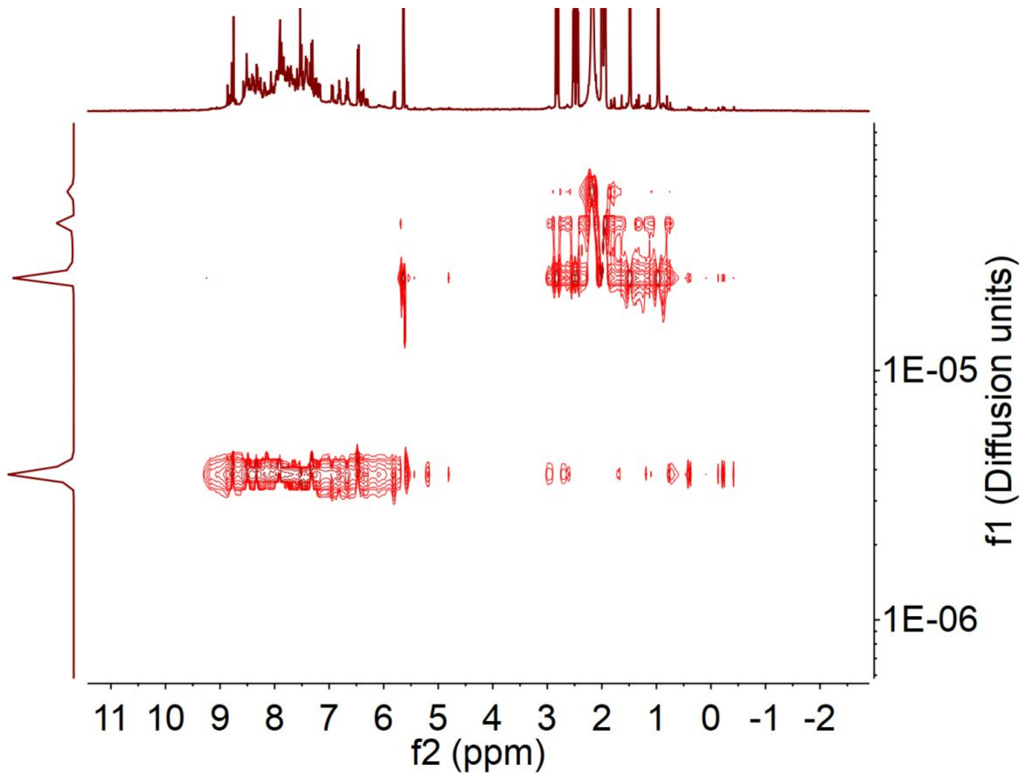

Figure S68: ^{1}H DOSY NMR spectrum of 2·**G4**⊂**3** (400 MHz, CD_3CN, 298 K). The proton signals belong to encapsulated guest could be observed.

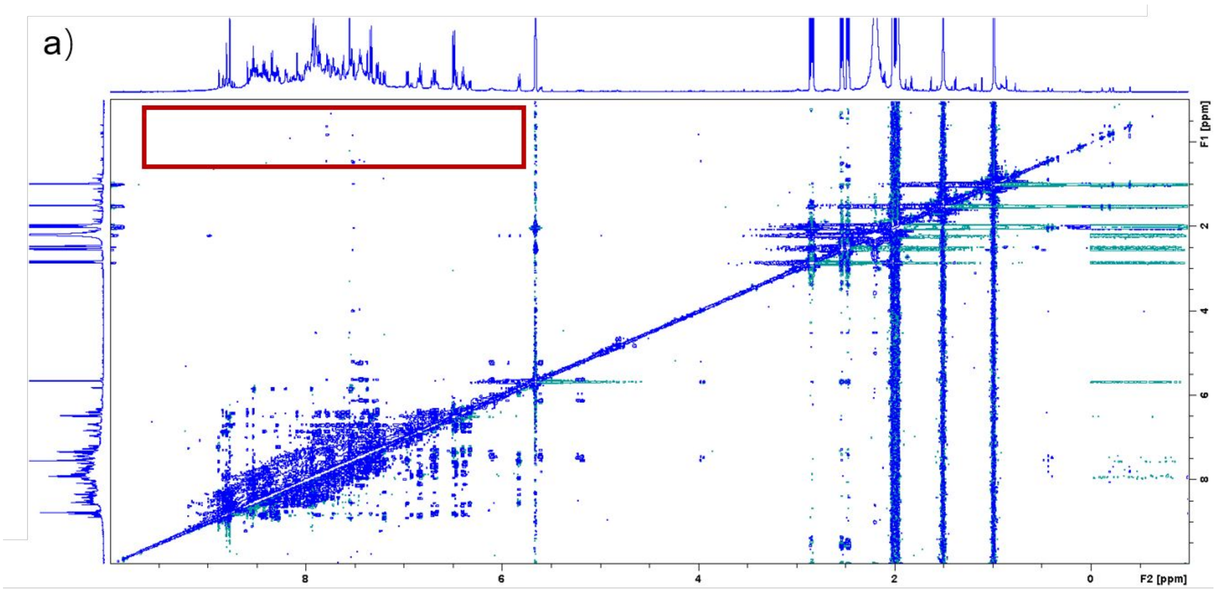

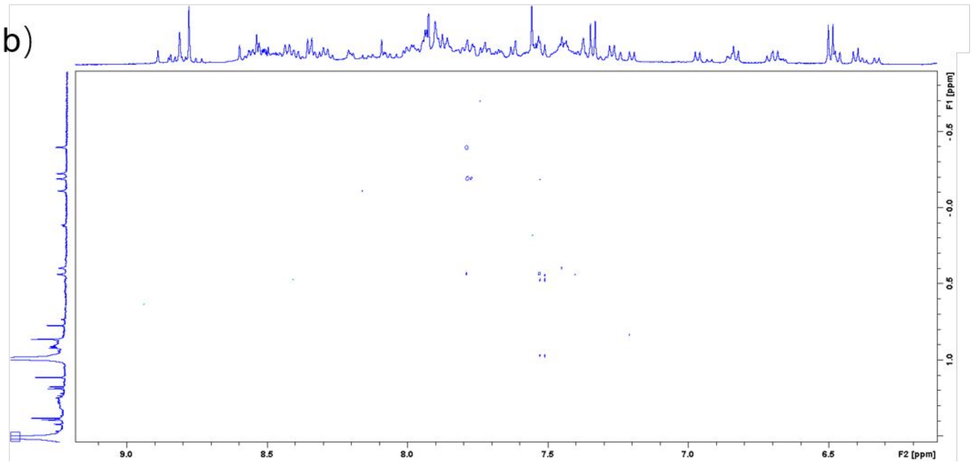

Figure S69: **a)** ^1H-^1H NOESY NMR spectrum of **G4⊂3** (400 MHz, CD_3CN, 298 K); **b)** Partial ^1H-^1H NOESY NMR spectrum of **G4⊂3.**

6.2.5 Host-guest interactions of cage mixture with (-)-beta pinene (G5)

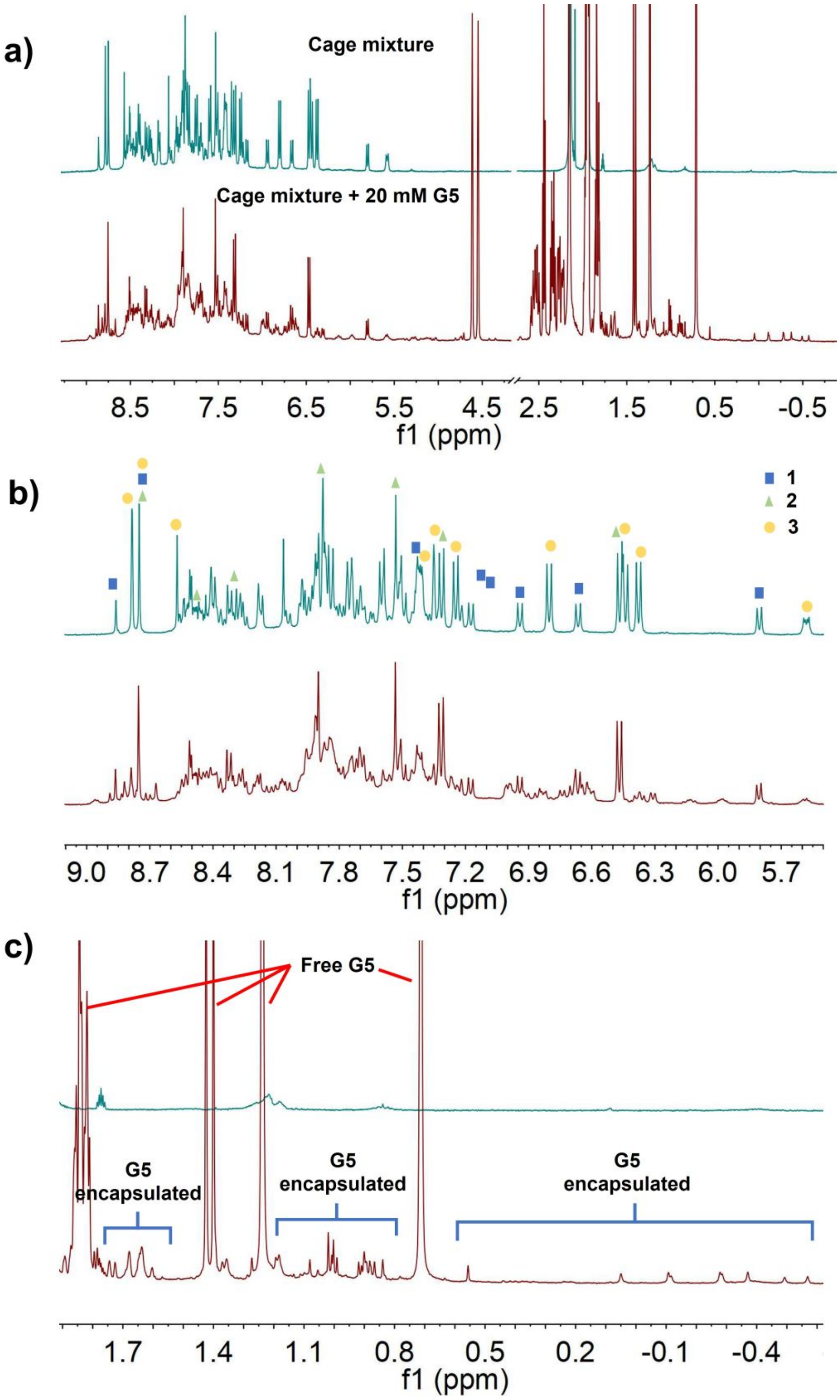

Figure S70: **a)** ^{1}H NMR (400 MHz, CD_3CN, 298 K) spectrum of the mixture of cages **1**-**3**, and the same mixture containing 20 mM **G5**. **G5** was encapsulated within **3** to form **G4⊂3**; **b)** In the aromatic ^{1}H NMR region of **a)**, a decrease in the intensity of empty cage **3** signals was observed, indicating host-guest binding between **G5** and **3**. The formation of desymmetrized **G5⊂3** gave a complex spectrum with multiple low-intensity peaks; **c)** Zoom in of the ^{1}H NMR spectrum shown in **a)**. The observation of signals corresponding to both free **G5** and encapsulated **G5** indicated slow-exchange host-guest binding on the NMR timescale.

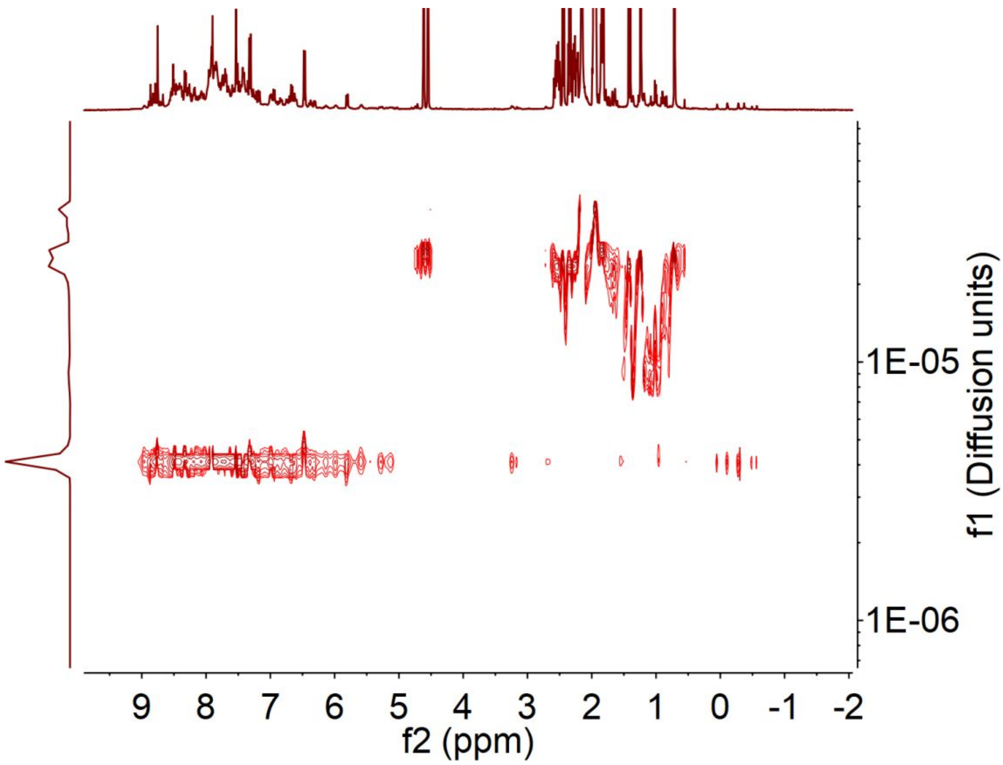

Figure S71: ^{1}H DOSY NMR spectrum of **G5⊂3** (400 MHz, CD_3CN, 298 K). The proton signals belong to encapsulated guest could be observed.

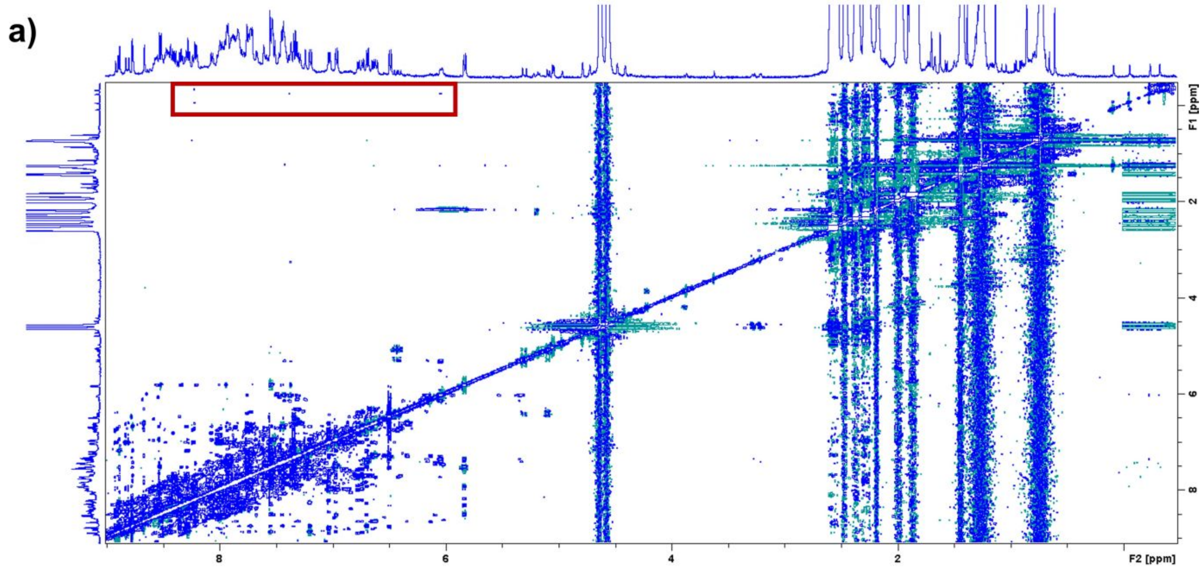

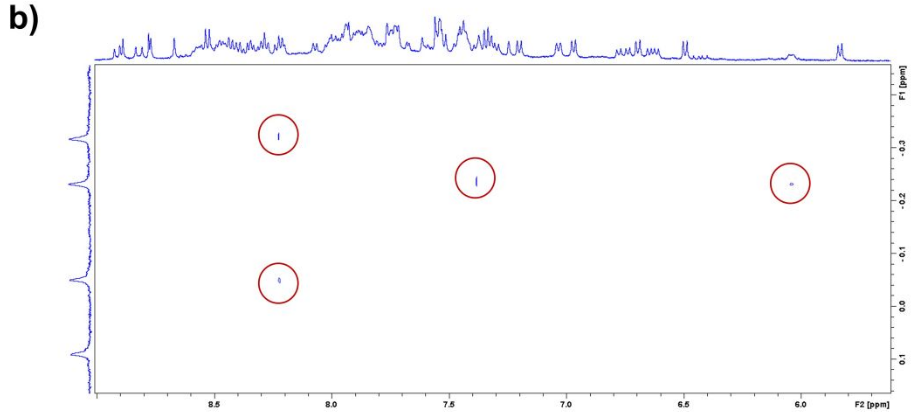

Figure S72: **a)** ^{1}H-^{1}H NOESY NMR spectrum of **G5⊂3** (400 MHz, CD_3CN, 298 K); **b)** Partial ^{1}H-^{1}H NOESY NMR spectrum of **G5⊂3.**

6.3 Host-guest interactions of cage 4 with G1-G6

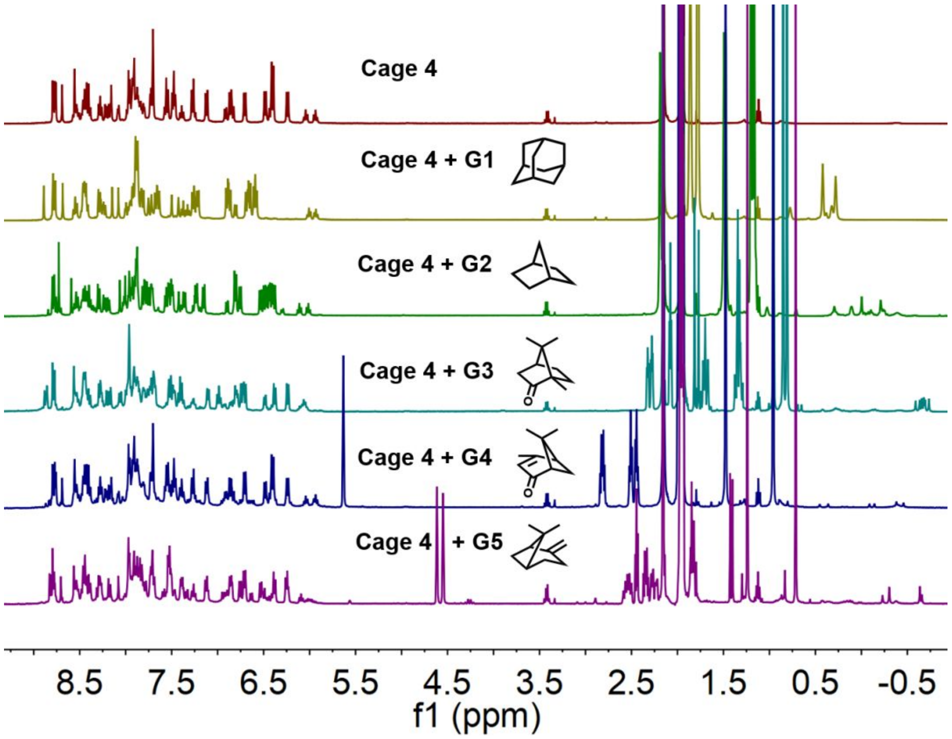

Figure S73: ^{1}H NMR (400 MHz, CD_3CN, 298 K) spectrum of cage **4** with guest molecules (**G1** to **G5**).

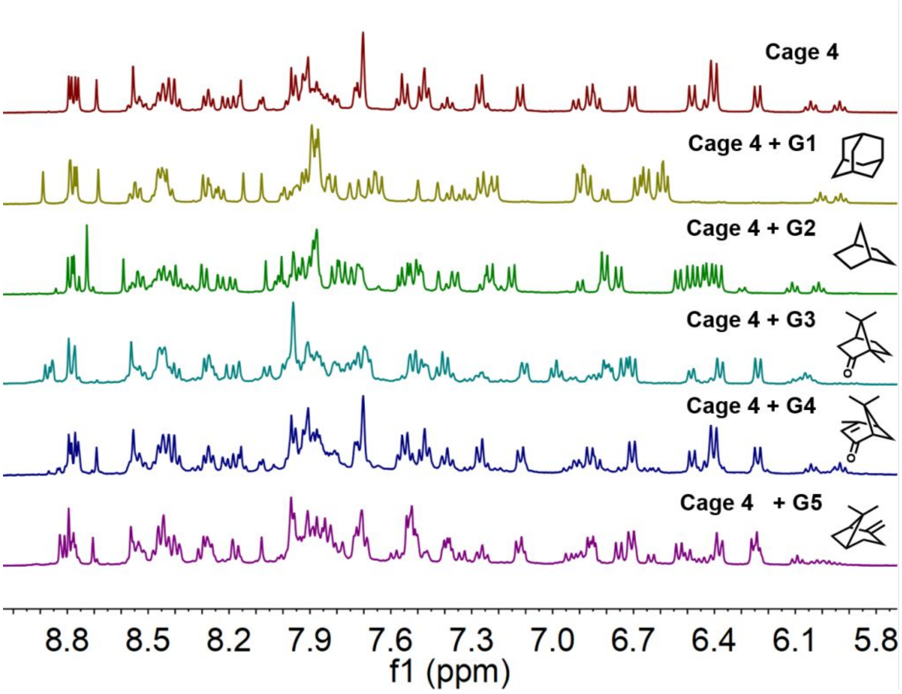

Figure S74: Aromatic region of ^{1}H NMR (400 MHz, CD_3CN, 298 K) spectrum of cage **4** with 20 equiv guest molecules (**G1** to **G5**).

6.3.1 Host-guest interactions of cage 4 with adamantane (G1)

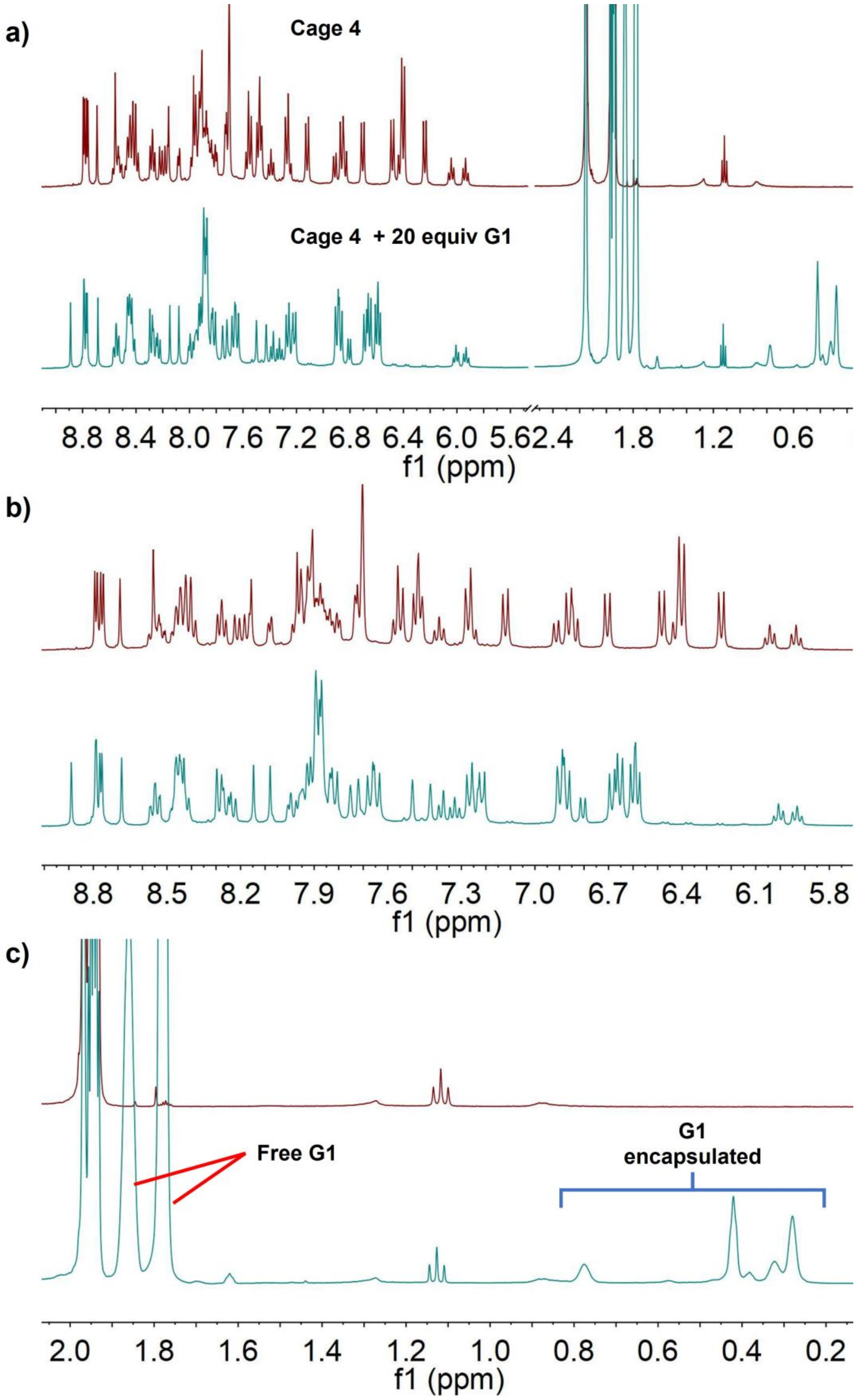

Figure S75: **a)** ^{1}H NMR (400 MHz, CD_3CN, 298 K) spectrum of cage **4** (1 mM) and cage **4** with 20 equiv **G1**. **G1** was encapsulated within cage **4** to form 2·**G1**⊂**4**; **b)** The aromatic region of the ^{1}H NMR spectrum shown in **a)**; **c)** Zoom in of the ^{1}H NMR spectrum shown in **a)**. The observation of signals corresponding to both free **G1** and encapsulated **G1** indicated slow-exchange host-guest binding on the NMR timescale.

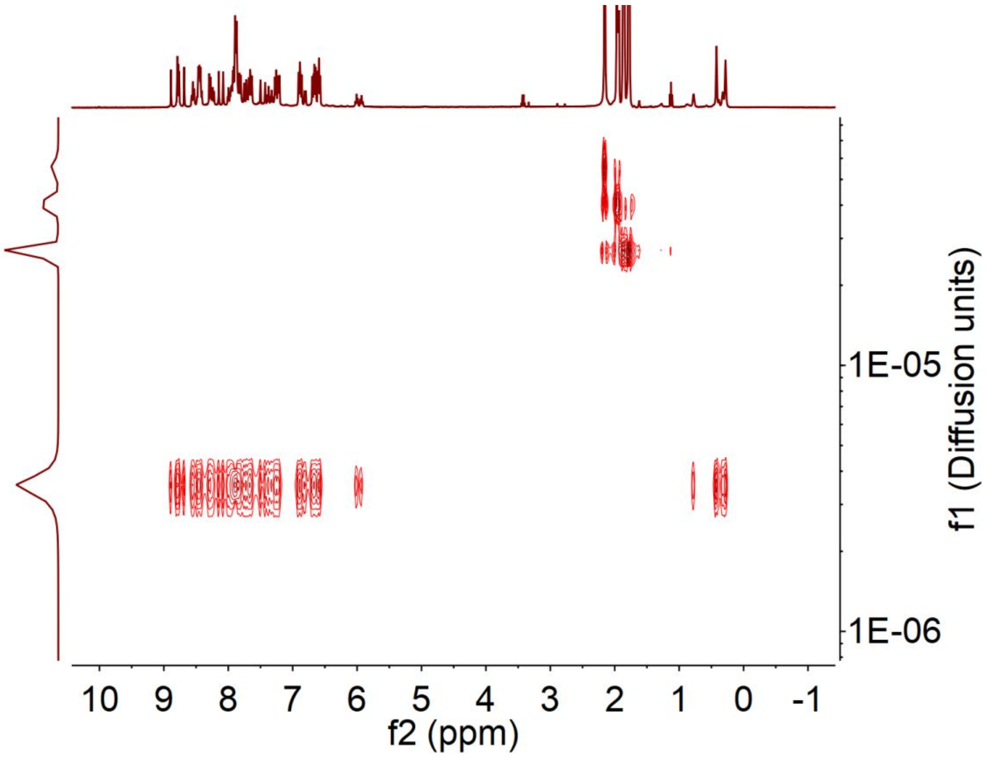

Figure S76: ^{1}H DOSY NMR spectrum of 2·**G1⊂4** (400 MHz, CD_3CN, 298 K). The proton signals belong to encapsulated guest could be observed.

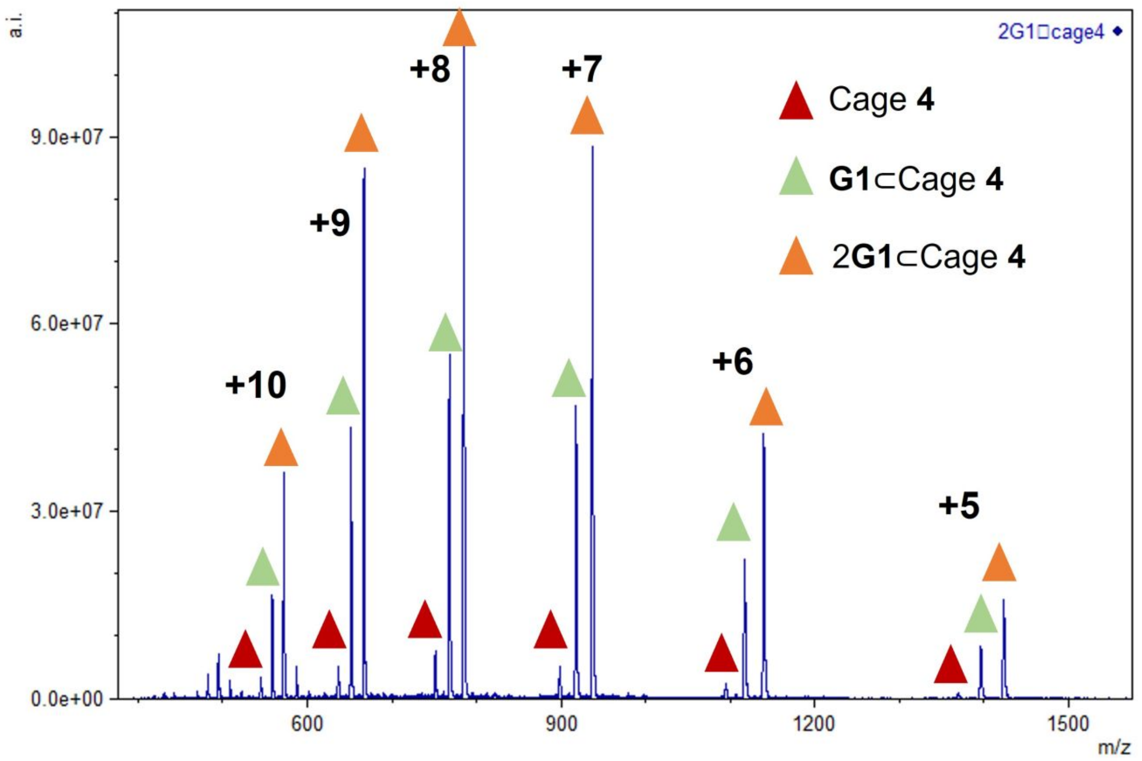

Figure S77: Low-resolution ESI-MS spectrum of 2·**G1⊂4** in MeCN. It was observed that the guest **G1** could undergo partial release from the complex, resulting in the detection of signals corresponding to both **G1⊂4** and empty **4**.

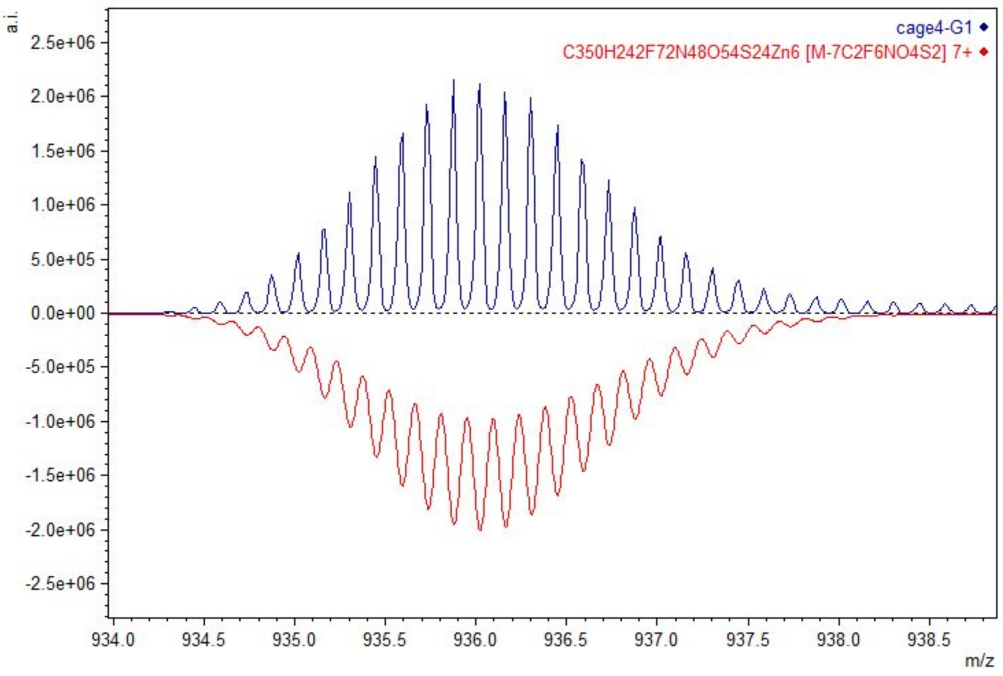

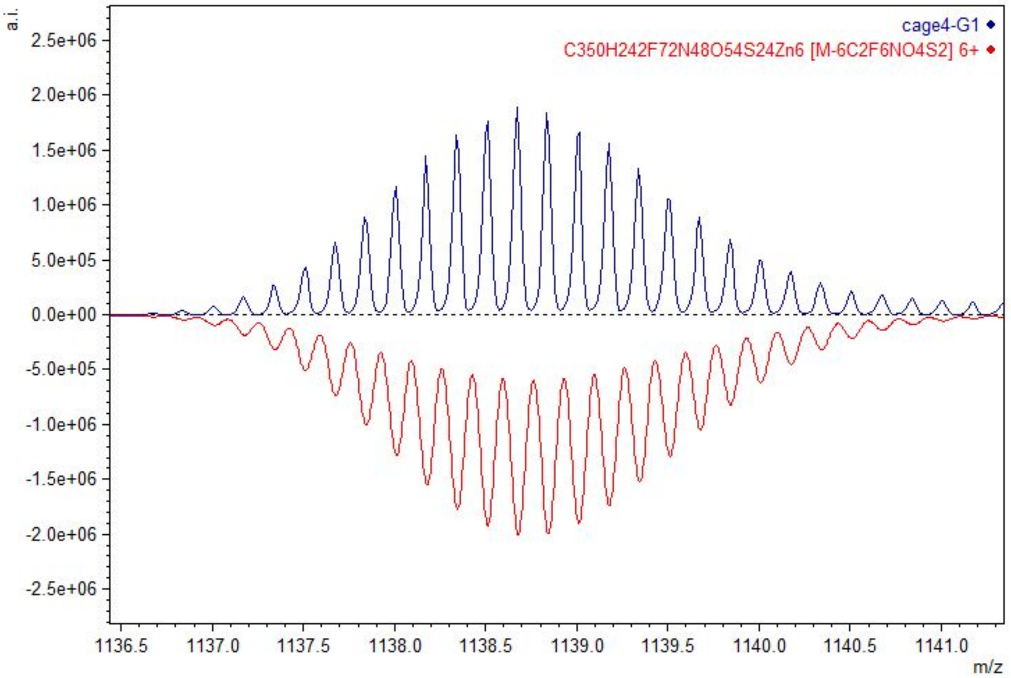

Figure S78: High-resolution ESI-mass spectrometric analysis of host-guest complex 2·**G1**⊂cage **4** showing the observed (blue) and theoretical (red) isotope patterns for the +7 and +6 peaks.

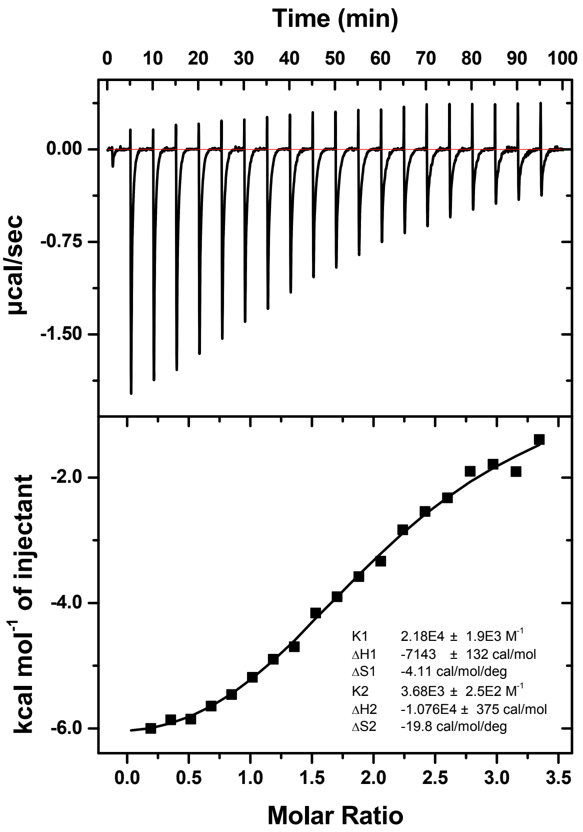

Figure S79: ITC experiments for cage **4** and **G1**. Titration plots (heat flow versus time and heat/mol versus guest/host ratio) were obtained by titrating a solution of the cage (0.15 mM) with **G1** (4 mM) in acetonitrile. The line represents the best fit using a two sequential binding sites model, in keeping with the two internal cavities of **4**. The binding constants were calculated to be $k_1 = (2.18 \pm 0.19) \times 10^4\ M^{-1}$ and $k_2 = (3.68 \pm 0.25) \times 10^3\ M^{-1}$.

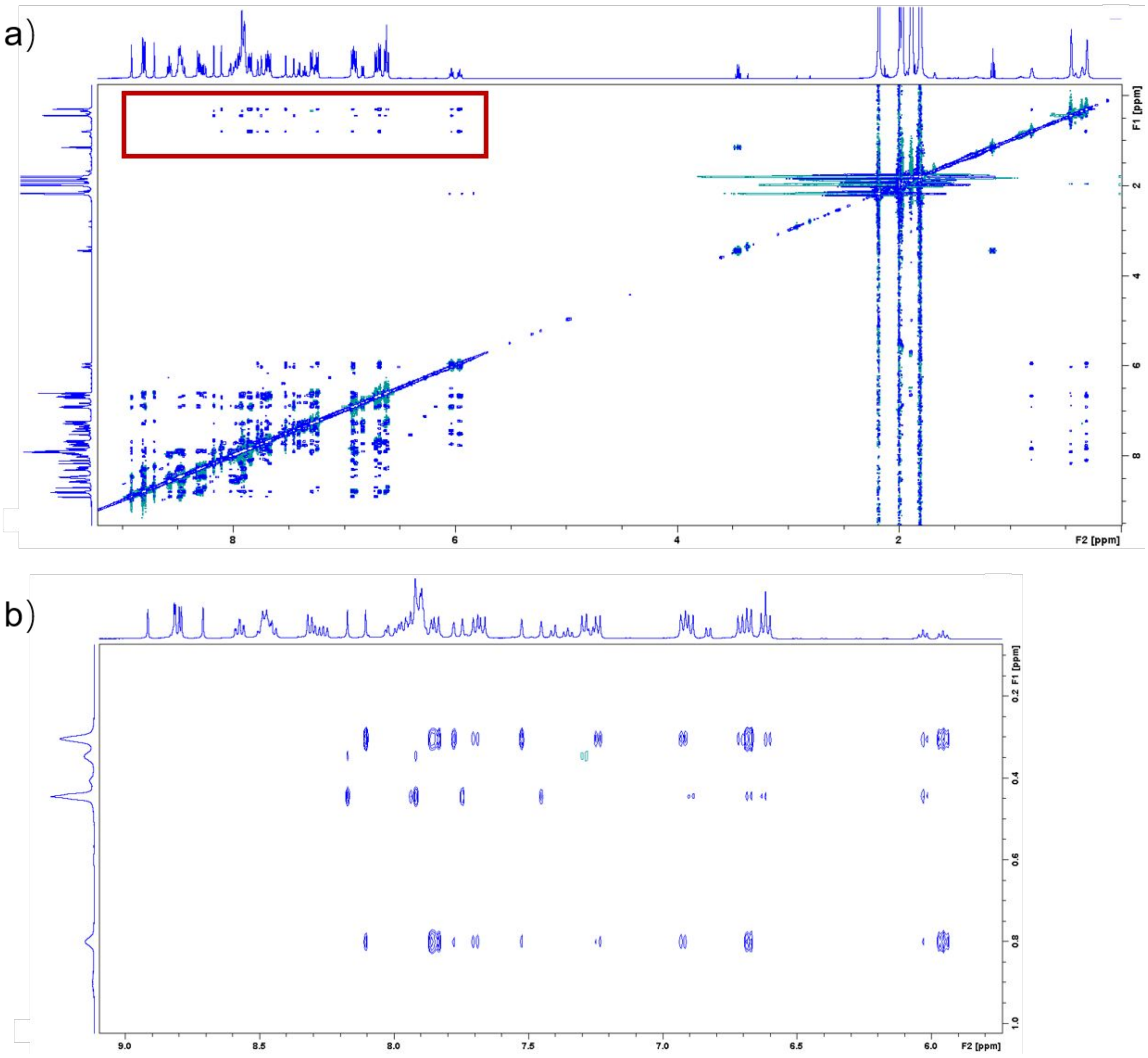

Figure S80: **a)** ^{1}H-^{1}H NOESY NMR spectrum of 2·**G1**⊂**4** (400 MHz, CD_3CN, 298 K); **b)** Partial ^{1}H-^{1}H NOESY NMR spectrum of 2·**G1**⊂**4.**

6.3.2 Host-guest interactions of cage 4 with norbornane (G2)

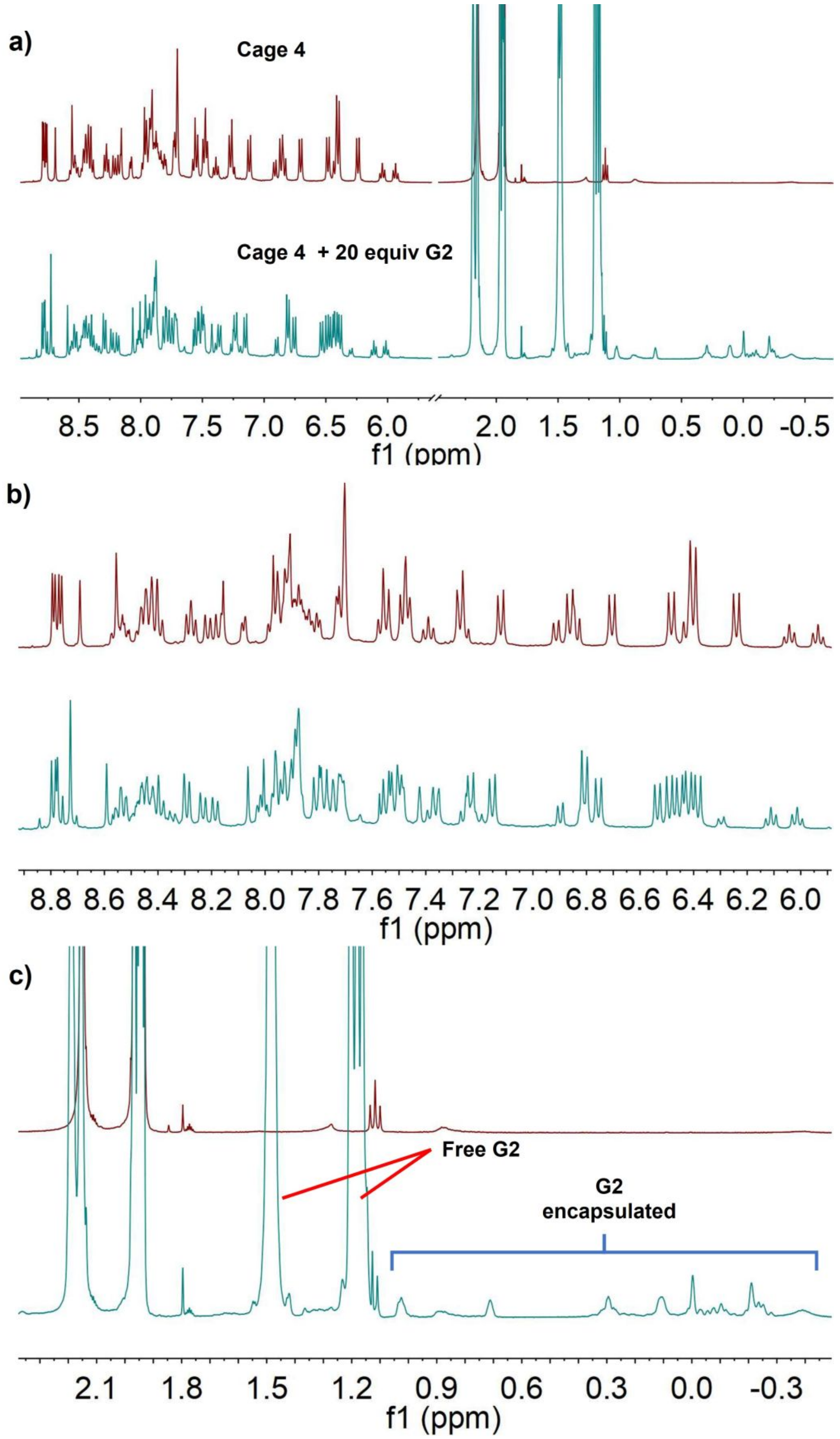

Figure S81: **a)** ^{1}H NMR (400 MHz, CD_3CN, 298 K) spectrum of cage **4** (1 mM) and cage **4** with 20 equiv **G2**. **b)** The aromatic region of the ^{1}H NMR spectrum shown in **a)**; no empty cage **4** was observed, confirming host-guest binding between **G2** and **4**; **c)** Zoom in of the ^{1}H NMR spectrum shown in **a)**. The observation of signals corresponding to both free **G2** and encapsulated **G2** indicated slow-exchange host-guest binding on the NMR timescale.

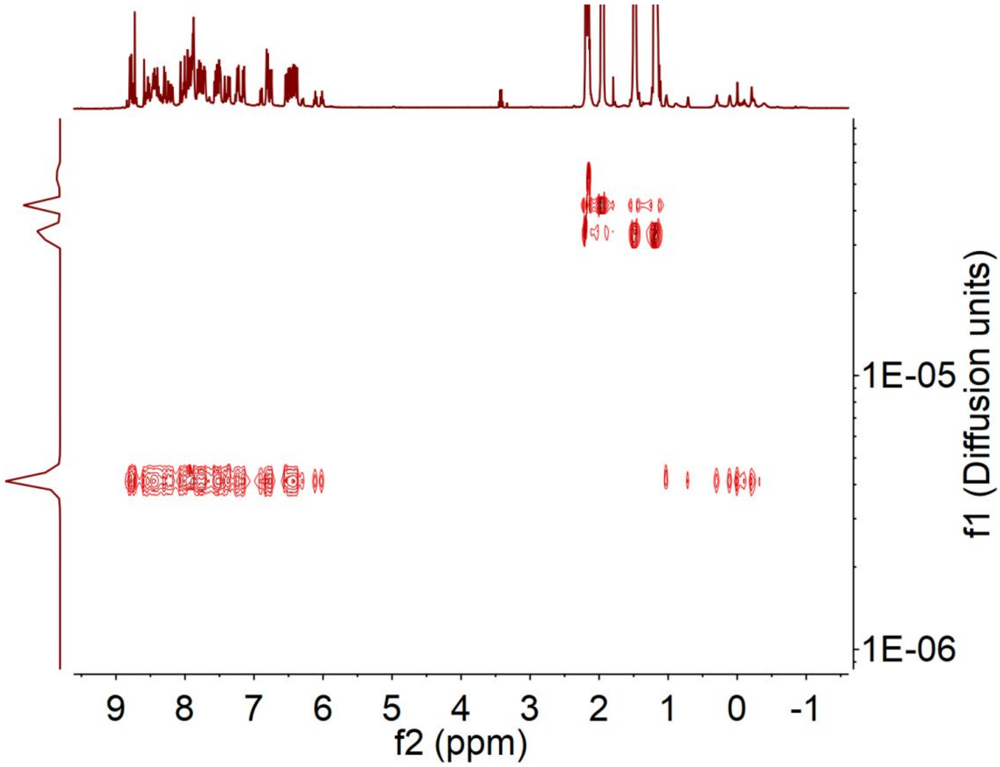

Figure S82: ^{1}H DOSY NMR spectrum of 2·**G2**⊂**4** (400 MHz, CD_3CN, 298 K). The proton signals belong to encapsulated guest could be observed.

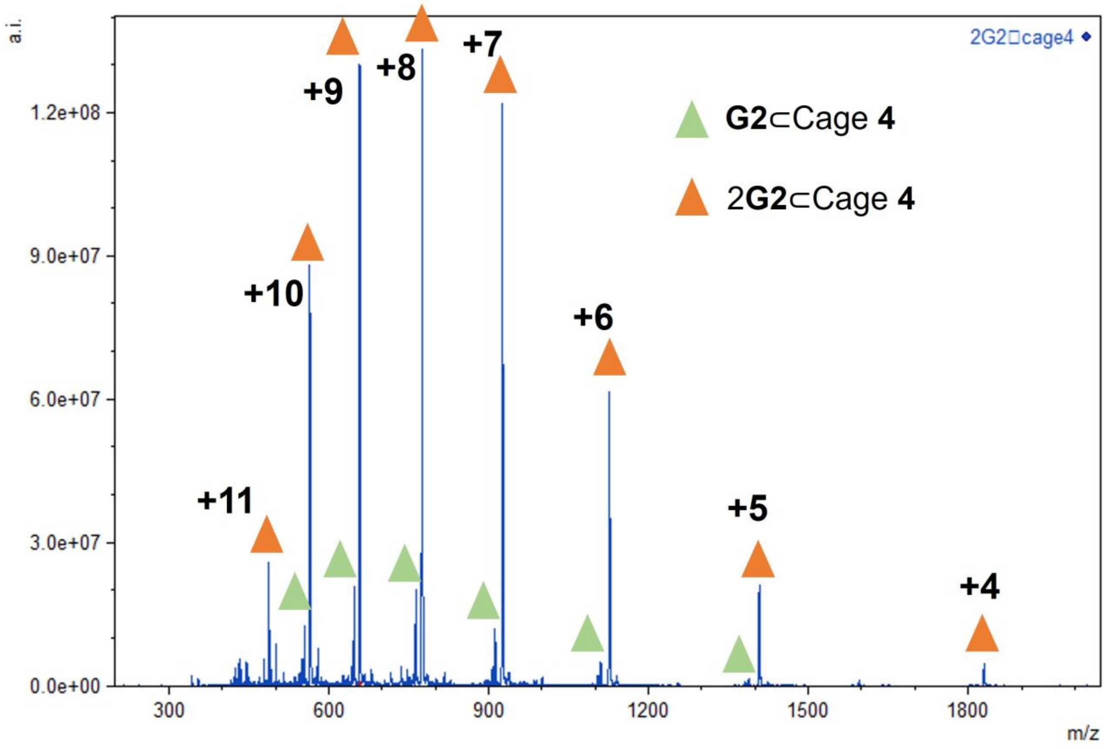

Figure S83: Low-resolution ESI-MS spectrum of 2·**G2**⊂cage **4** in MeCN.

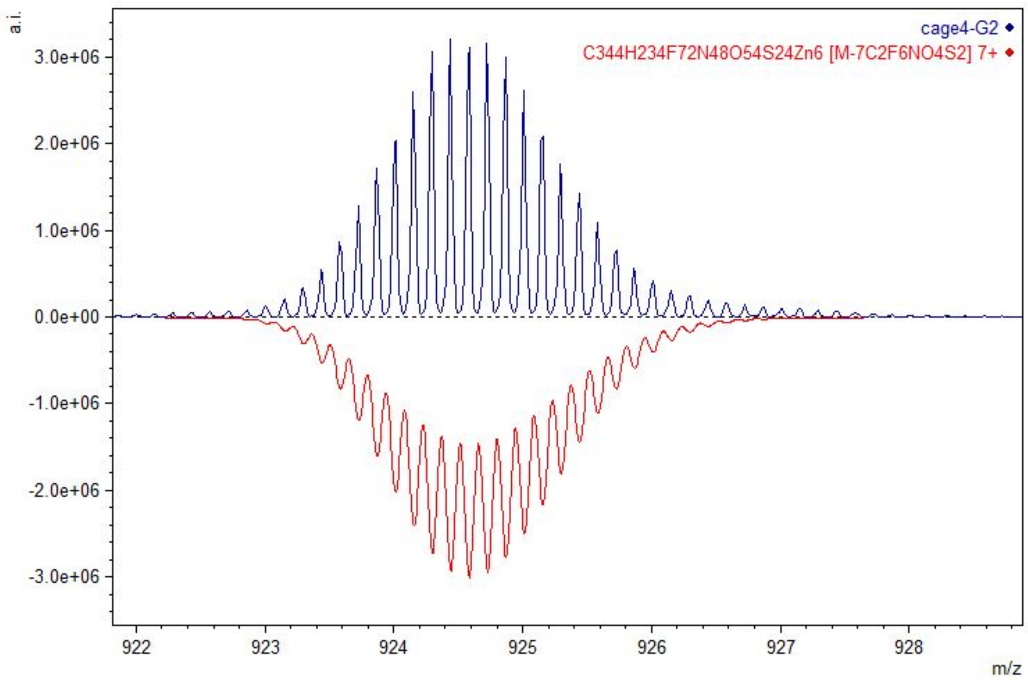

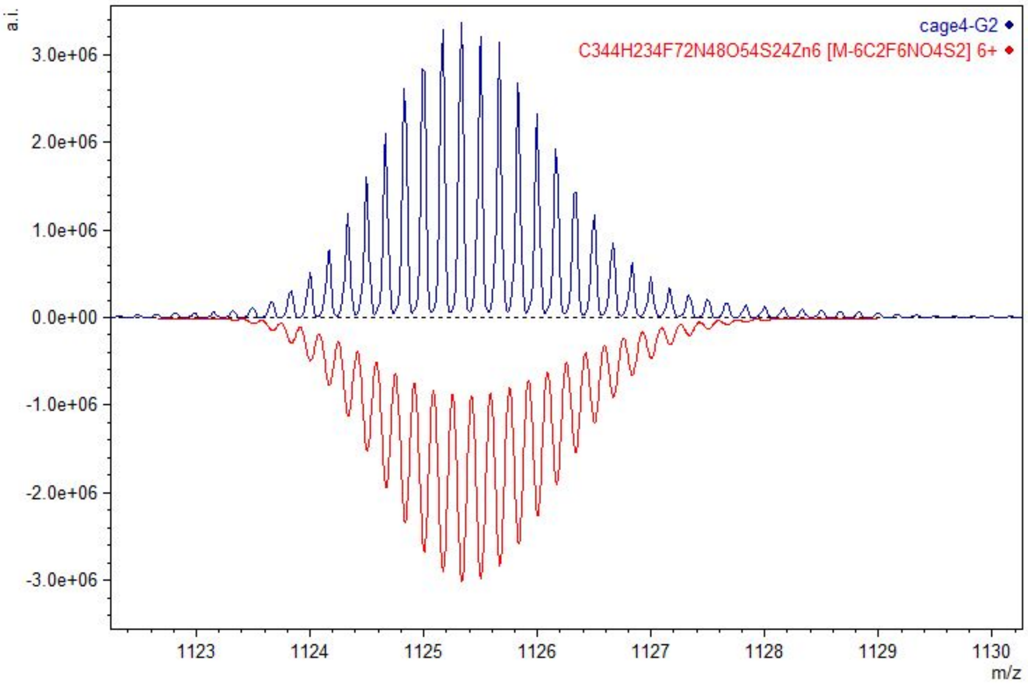

Figure S84: High-resolution ESI-mass spectrometric analysis of host-guest complex 2·**G2**⊂cage **4** showing the observed (blue) and theoretical (red) isotope patterns for the +7 and +6 peaks.

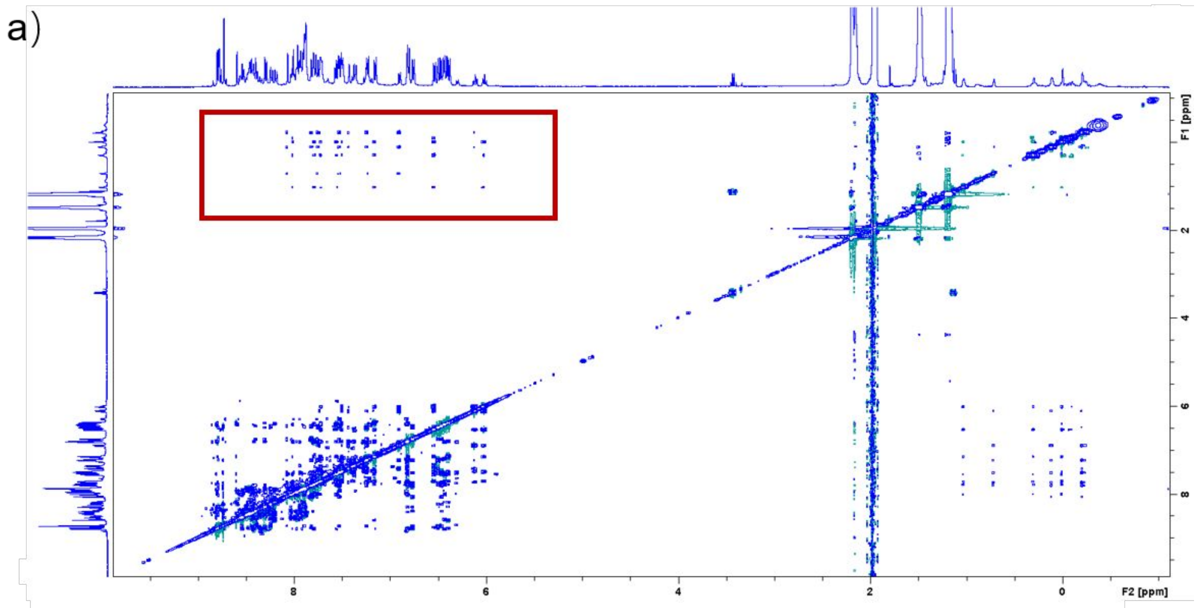

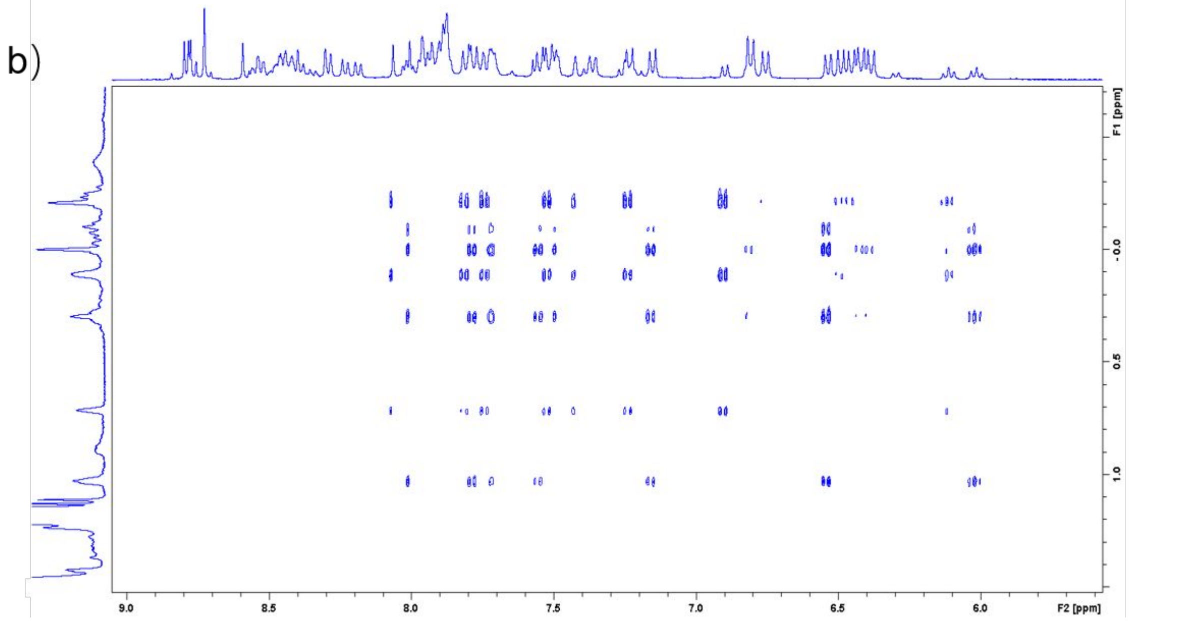

Figure S85: **a)** ^1H-^1H NOESY NMR spectrum of 2·**G2⊂4** (400 MHz, CD_3CN, 298 K); **b)** Partial ^1H-^1H NOESY NMR spectrum of 2·**G2⊂4.**

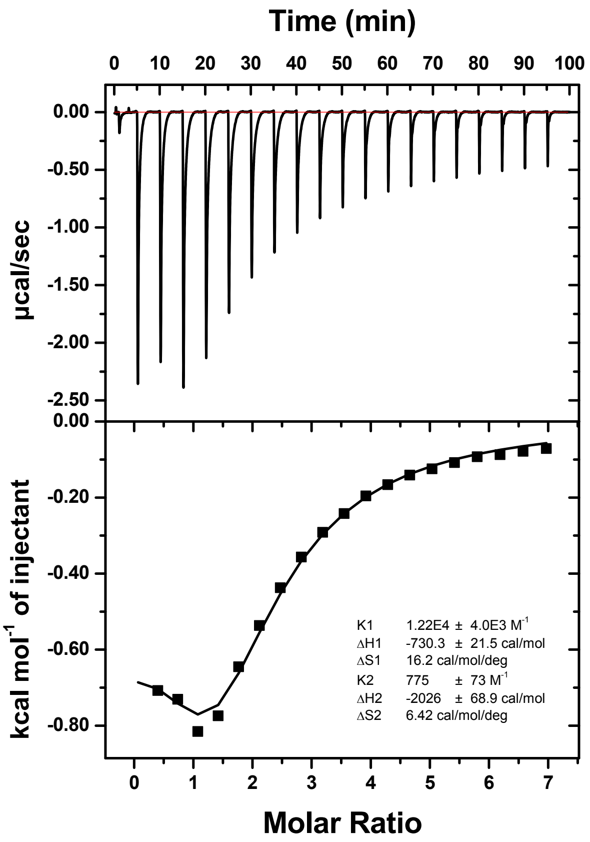

Figure S86: ITC experiments for cage **4** and **G2**. Titration plots (heat flow versus time and heat/mol versus guest/host ratio) were obtained by titrating a solution of the cage (1 mM) with **G2** (40 mM) in acetonitrile. The line represents the best fit using a two sequential binding sites model, in keeping with the two internal cavities of **4**. The binding constants were calculated to be $k_1 = (1.22 \pm 0.40) \times 10^4 M^{-1}$ and $k_2 = (7.75 \pm 0.73) \times 10^2 M^{-1}$.

6.3.3 Host-guest interactions of cage 4 with (1*S*)-(-)-camphor (G3)

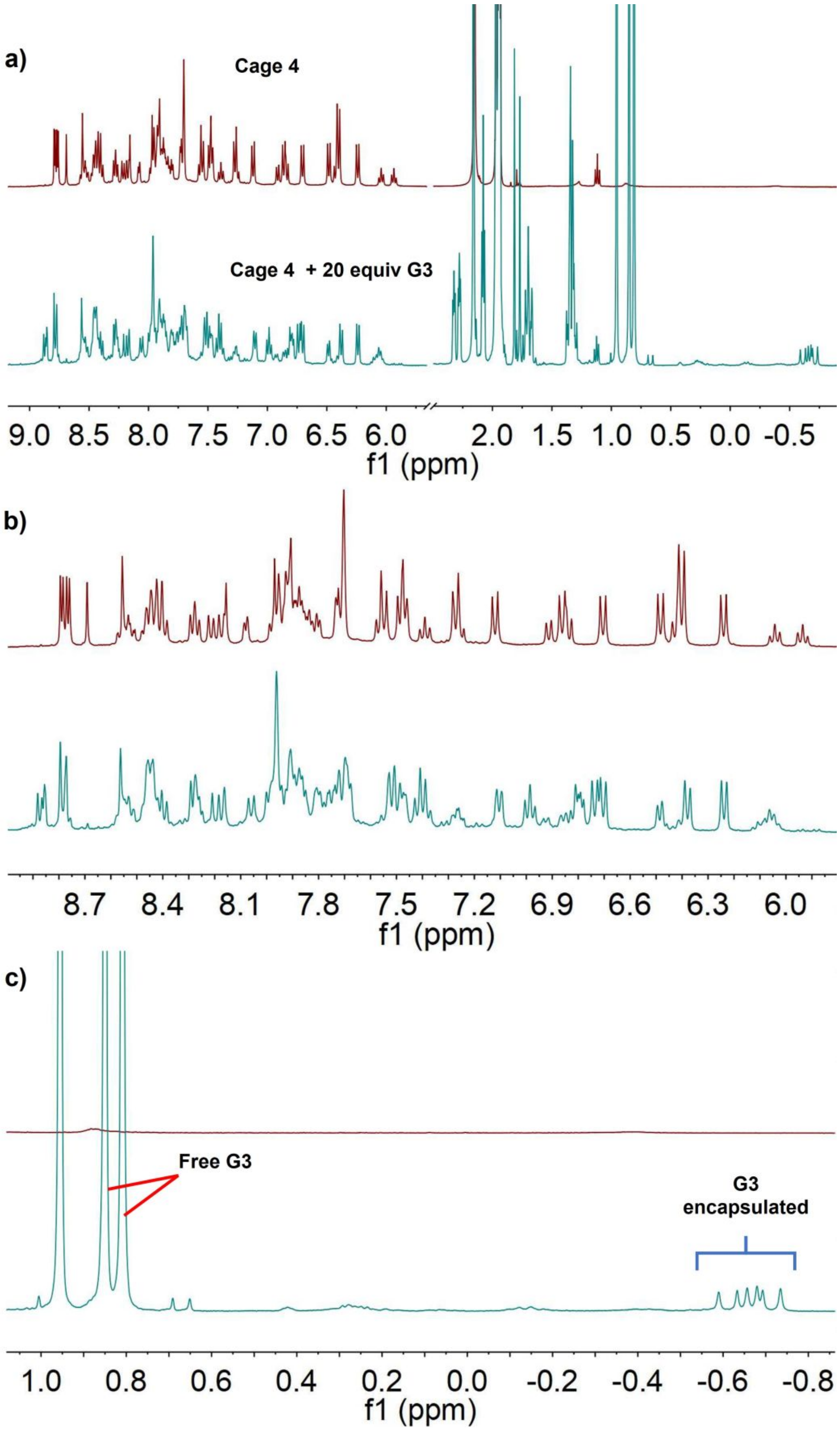

Figure S87: **a)** ^{1}H NMR (400 MHz, CD_3CN, 298 K) spectrum of cage **4** (1 mM) and cage **4** with 20 equiv **G3**; **b)** The aromatic region of the ^{1}H NMR spectrum shown in **a)**; no empty cage **4** was observed indicating host-guest binding between **G3** and **4**; **c)** Zoom in of the ^{1}H NMR spectrum shown in **a)**. The observation of signals corresponding to both free **G3** and encapsulated **G3** indicated slow-exchange host-guest binding on the NMR timescale

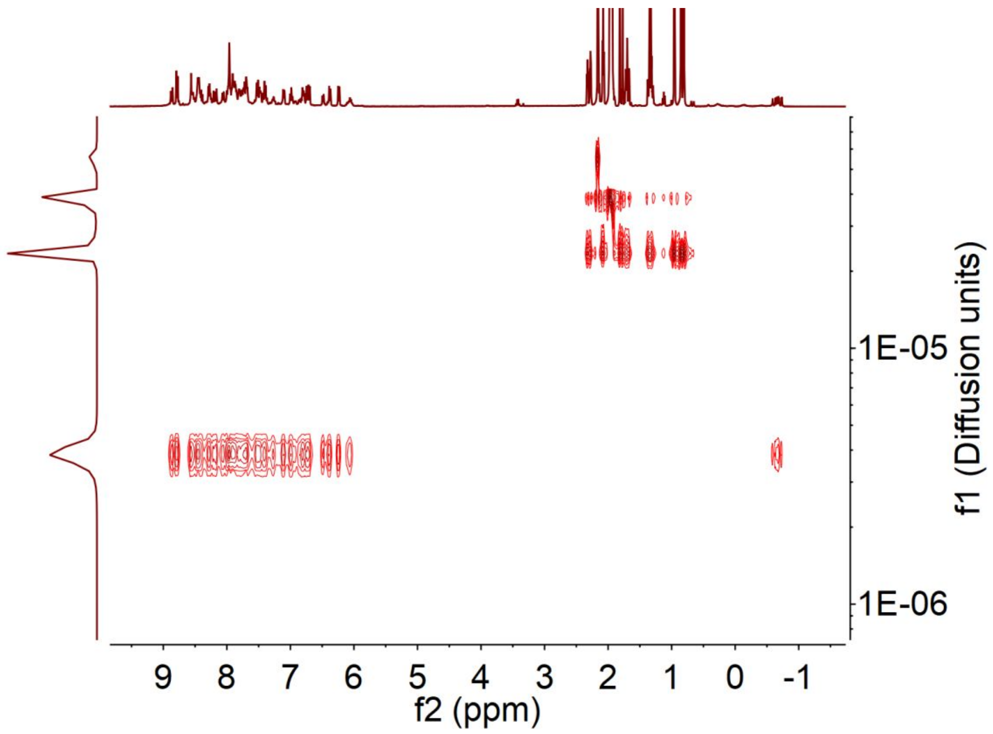

Figure S88: ^{1}H DOSY NMR spectrum of 2·**G3**⊂**4** (400 MHz, CD_3CN, 298 K). The proton signals belong to encapsulated guest could be observed.

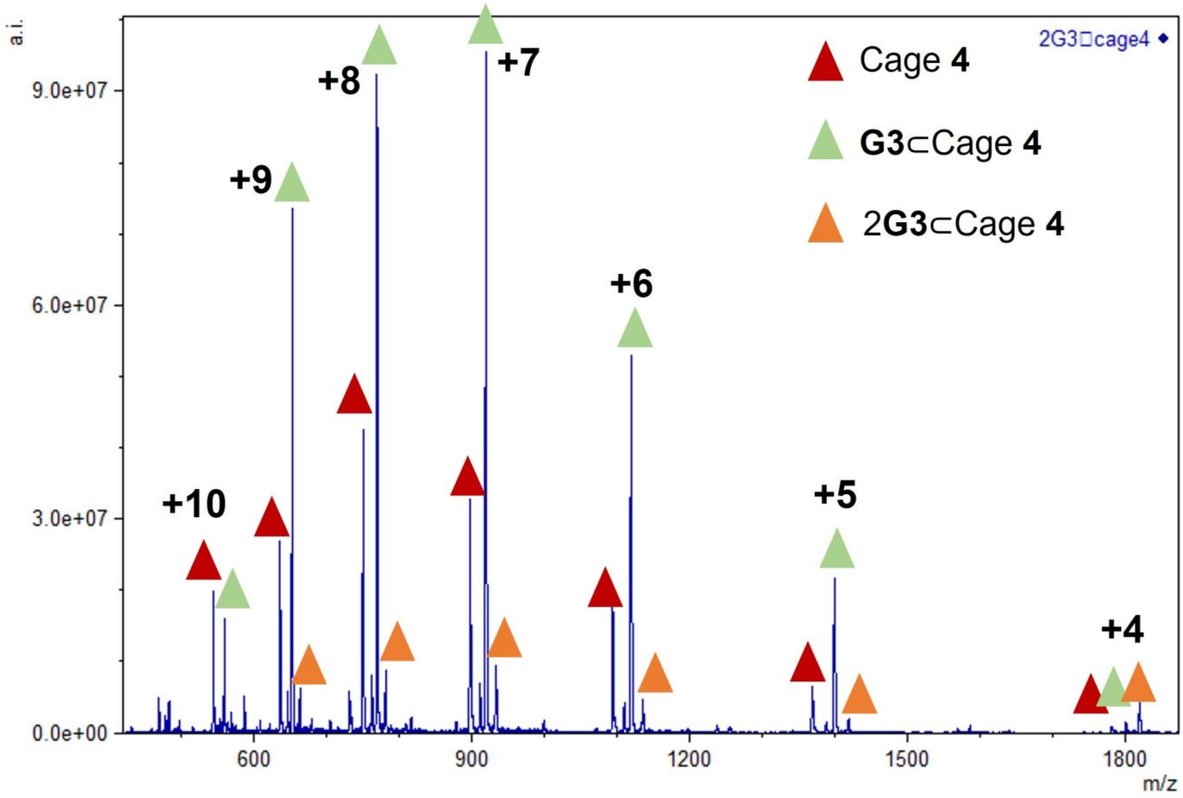

Figure S89: Low-resolution ESI-MS spectrum of 2·**G3**⊂cage **4** in MeCN.

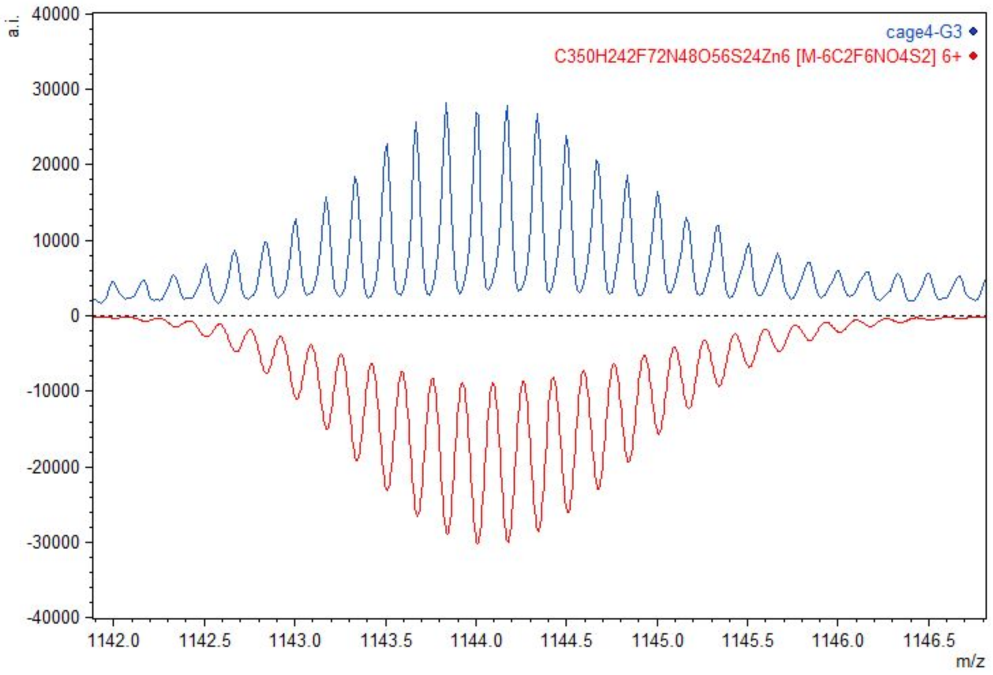

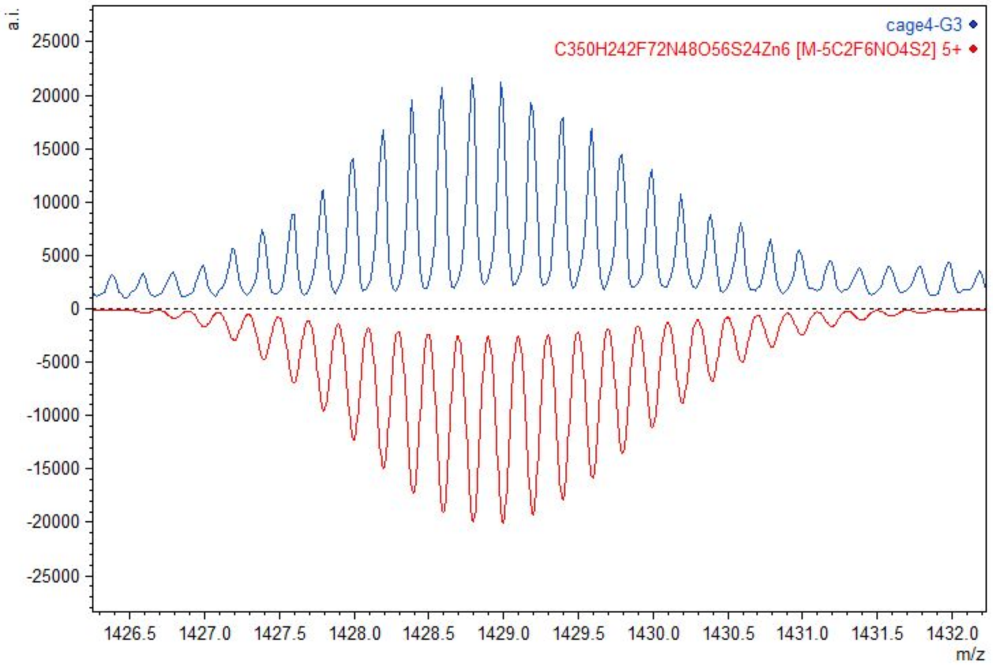

Figure S90: High-resolution ESI-mass spectrometric analysis of host-guest complex 2·**G3**⊂cage **4** showing the observed (blue) and theoretical (red) isotope patterns for the +6 and +5 peaks.

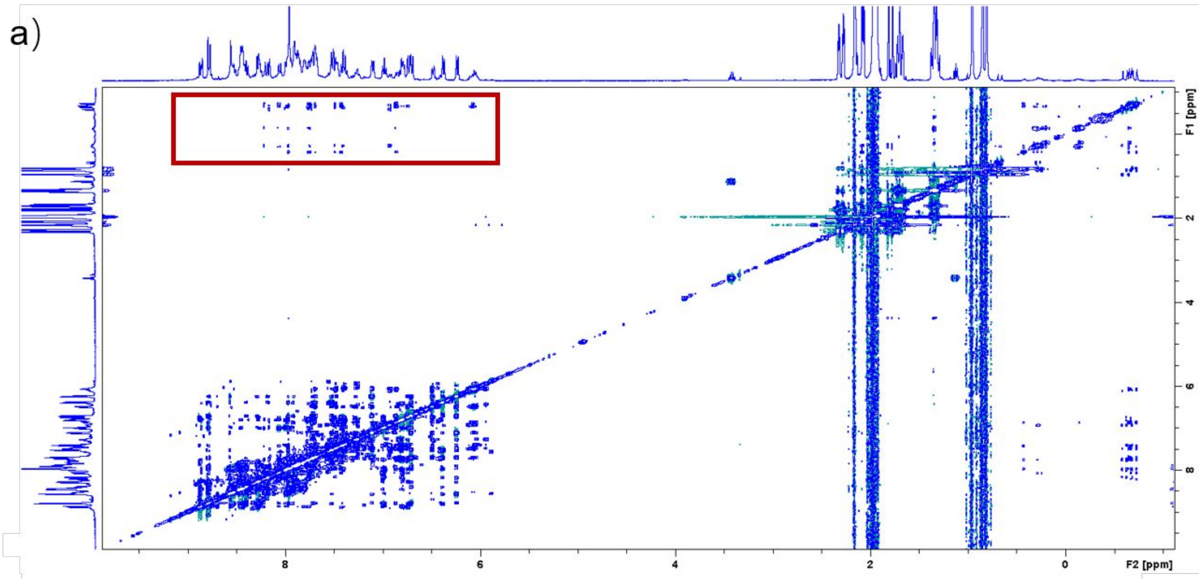

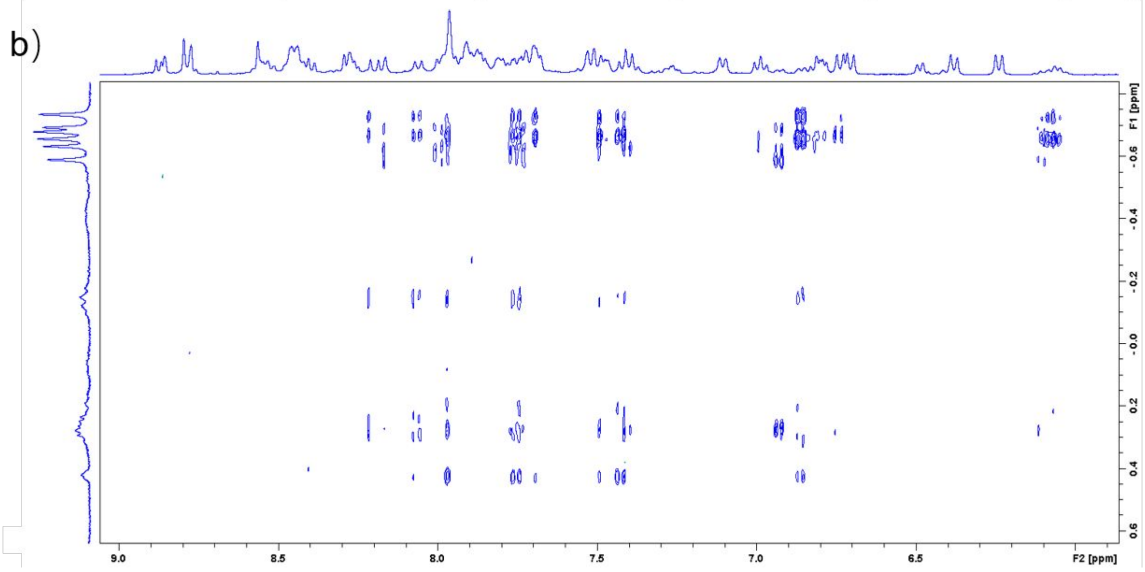

Figure S91: **a)** ^{1}H-^{1}H NOESY NMR spectrum of 2·**G3**⊂**4** (400 MHz, CD_3CN, 298 K); **b)** Partial ^{1}H-^{1}H NOESY NMR spectrum of 2·**G3**⊂**4.**

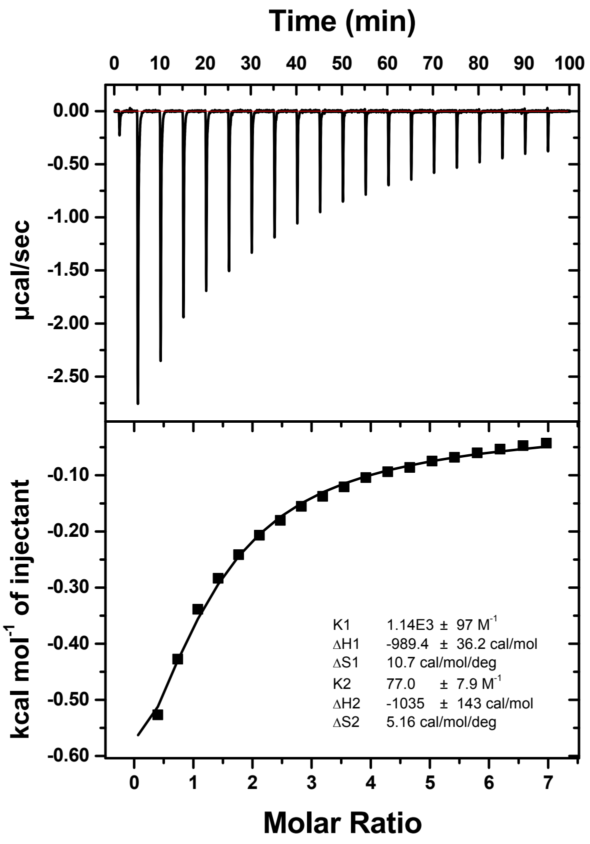

Figure S92: ITC experiments for cage **4** and **G3**. Titration plots (heat flow versus time and heat/mol versus guest/host ratio) were obtained by titrating a solution of the cage (1 mM) with **G3** (40 mM) in acetonitrile. The line represents the best fit using a two sequential binding sites model, in keeping with the two internal cavities of **4**. The binding constants were calculated to be $k_1 = (1.14 \pm 0.10) \times 10^3$ M^{-1} and $k_2 = (7.70 \pm 0.79) \times 10^1$ M^{-1}.

6.3.4 Host-guest interactions of cage 4 with verbenone (G4)

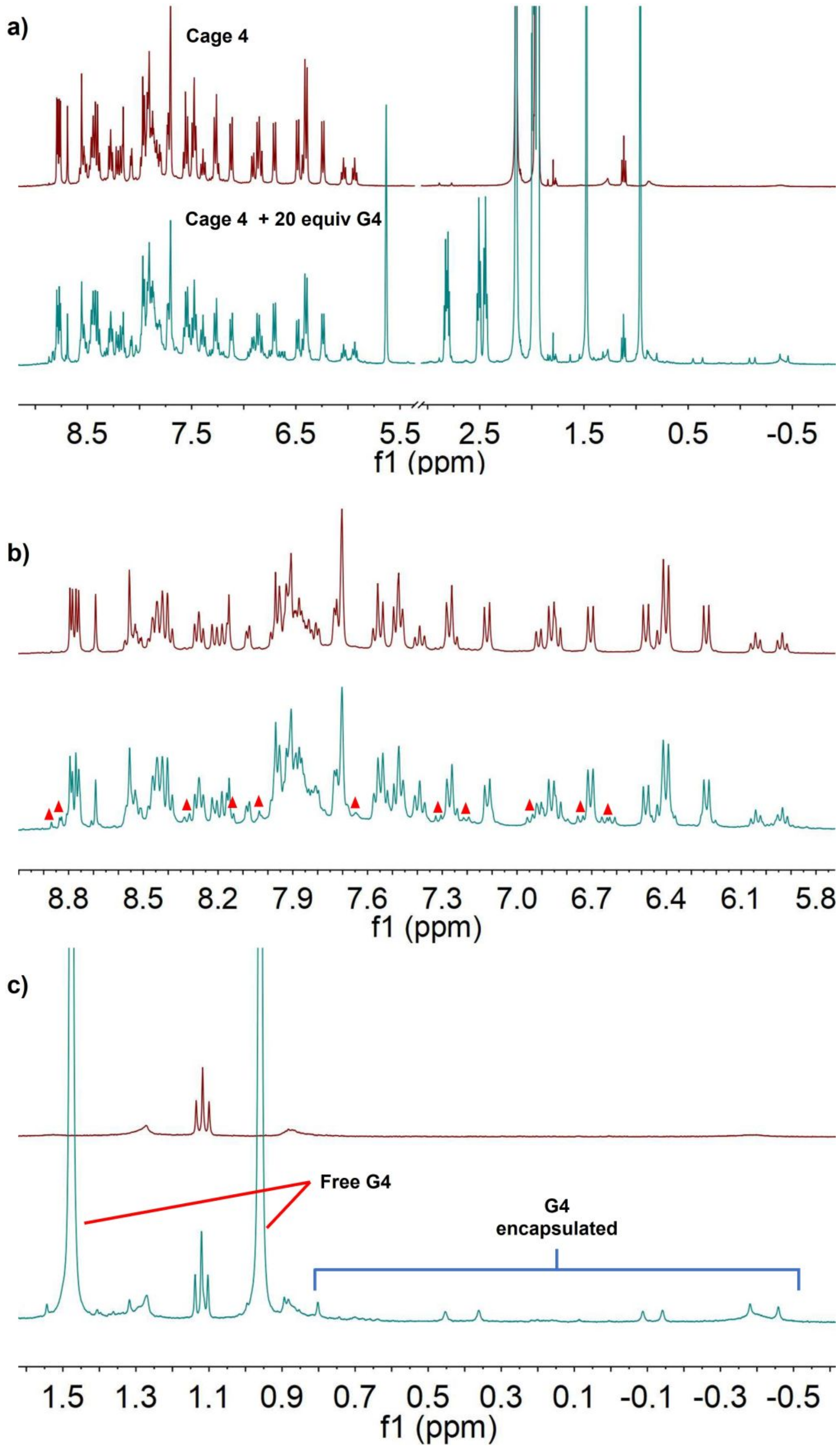

Figure S93: **a)** ^{1}H NMR (400 MHz, CD_3CN, 298 K) spectra of cage **4** (1 mM) and cage **4** with 20 equiv **G4**; **b)** The aromatic region of the ^{1}H NMR spectrum shown in **a)**, a new set of low-intensity peaks was observed, suggesting weak host-guest binding between **G4** and **4**, consistent with the data shown in Figures S95 and S98; **c)** Zoom in of the ^{1}H NMR spectrum shown in **a)**. The observation of signals corresponding to both free **G4** and encapsulated **G4** indicated slow-exchange host-guest binding on the NMR timescale.

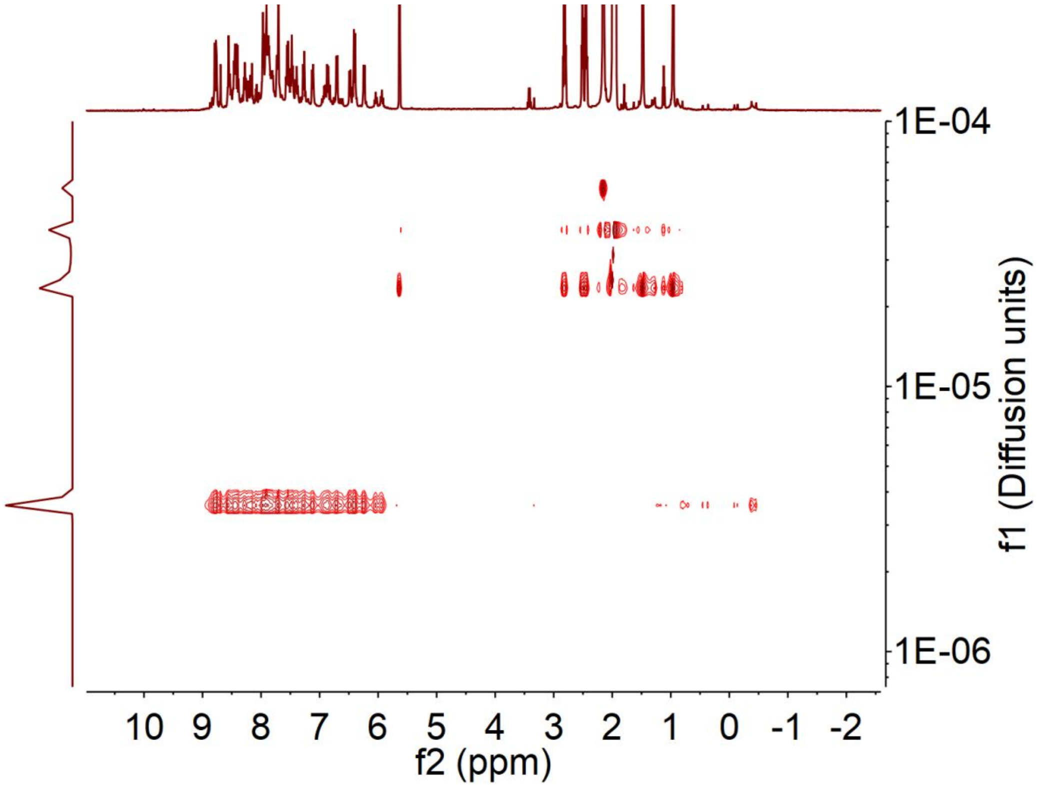

Figure S94: ^{1}H DOSY NMR spectrum of **G4⊂4** (400 MHz, CD_3CN, 298 K). The proton signals belong to encapsulated guest could be observed.

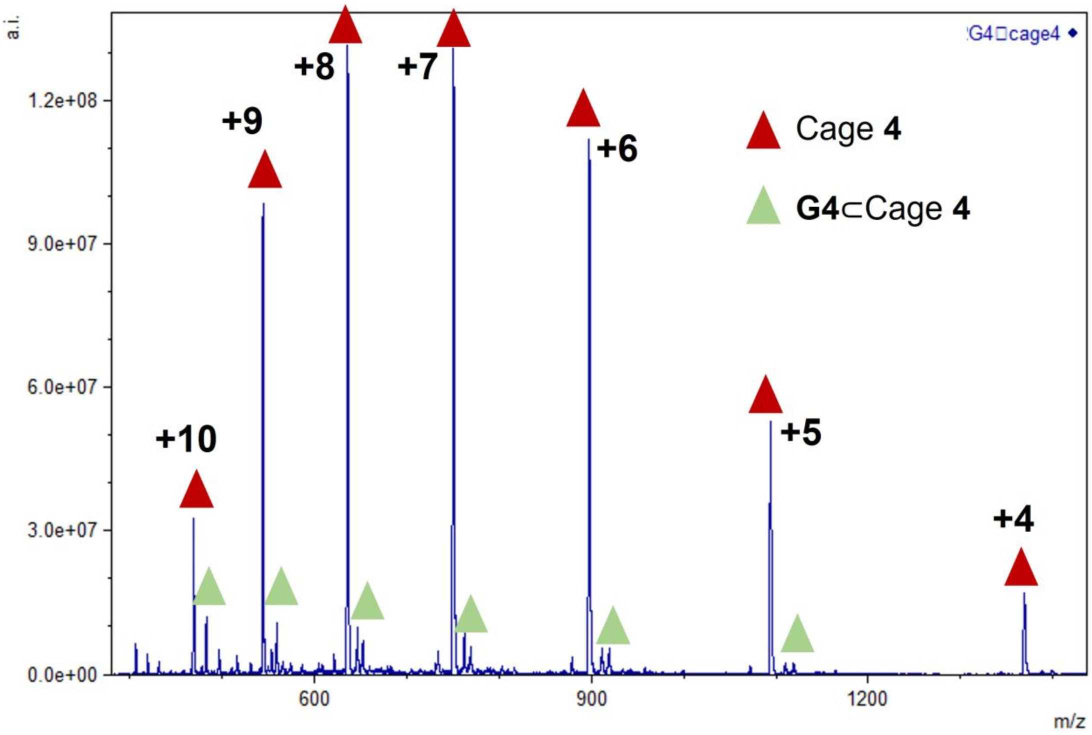

Figure S95: Low-resolution ESI-MS spectrum of **G4**⊂cage **4** in MeCN. The low intensity of the peaks corresponding to **G4**⊂cage **4** are inferred to result from weak binding between **G4** and cage **4**.

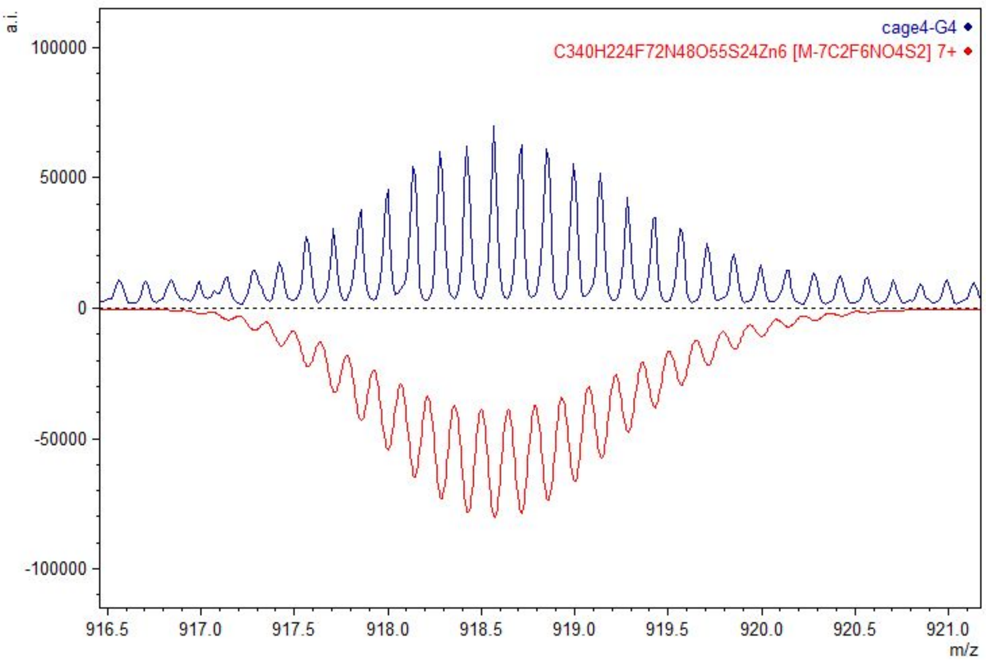

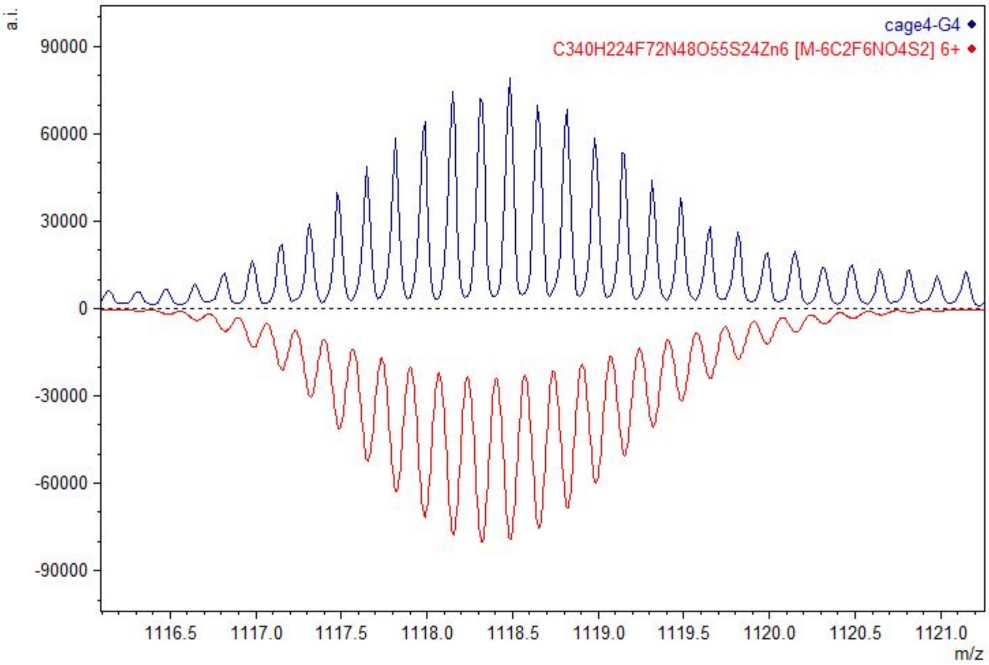

Figure S96: High-resolution ESI-mass spectrometric analysis of host-guest complex **G4**⊂cage **4** showing the observed (blue) and theoretical (red) isotope patterns for the +7 and +6 peaks.

.

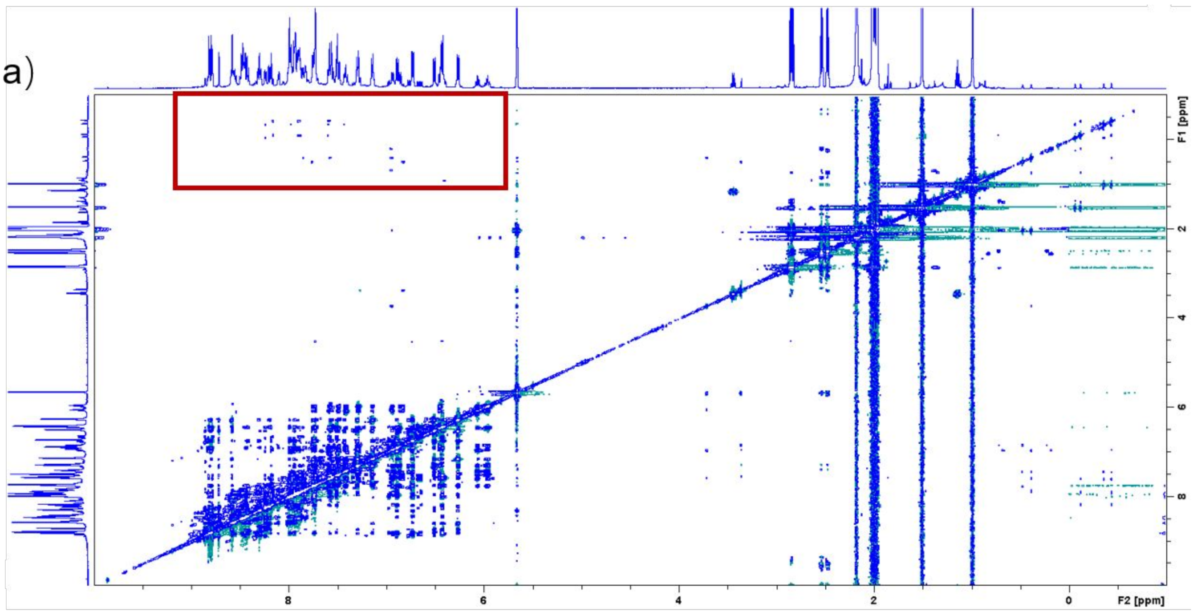

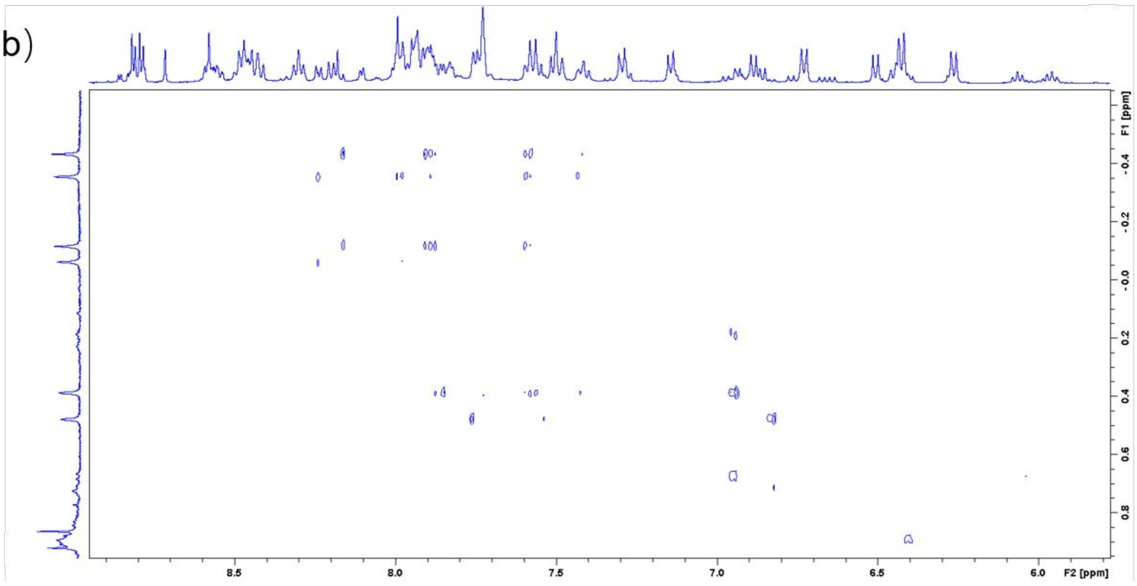

Figure S97: **a)** ^1H-^1H NOESY NMR spectrum of **G4⊂4** (400 MHz, CD_3CN, 298 K); **b)** Partial ^1H-^1H NOESY NMR spectrum of **G4⊂4.**

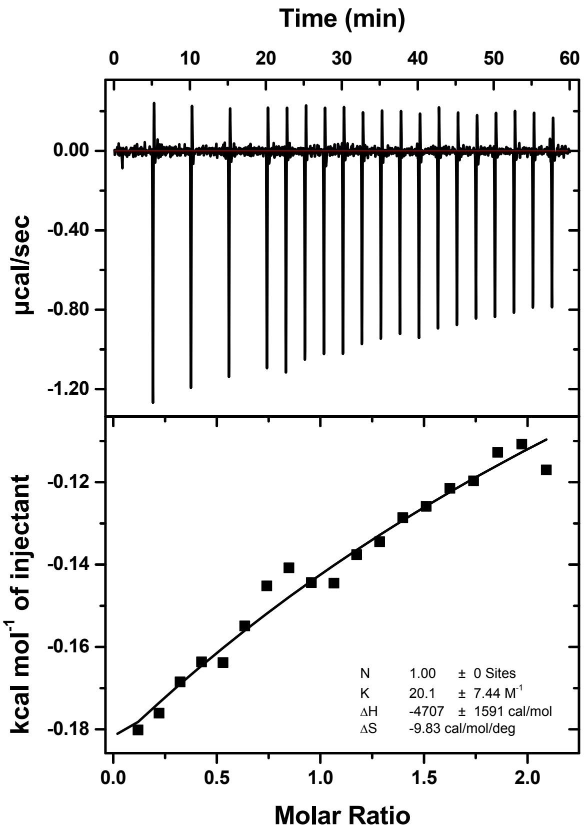

Figure S98: ITC experiments for cage **4** and **G4**. Titration plots (heat flow versus time and heat/mol versus guest/host ratio) were obtained by titrating a solution of the cage (2 mM) with **G4** (20 mM) in acetonitrile. The line represents the best fit resulting from fitting using a one site binding model, showing poor fitting with a two sequential binding sites model. The binding constant was calculated to be (2.01 ± 0.74) × 10^1 M^{-1}. Single guest binding was also supported by ESI-MS analysis, as shown in Figures S95 and S96. We infer that a putative second binding event between cage **4** and **G4** was too weak for ITC measurement, given the weakness of initial guest binding.

6.3.5 Host-guest interactions of cage 4 with (-)-beta pinene (G5)

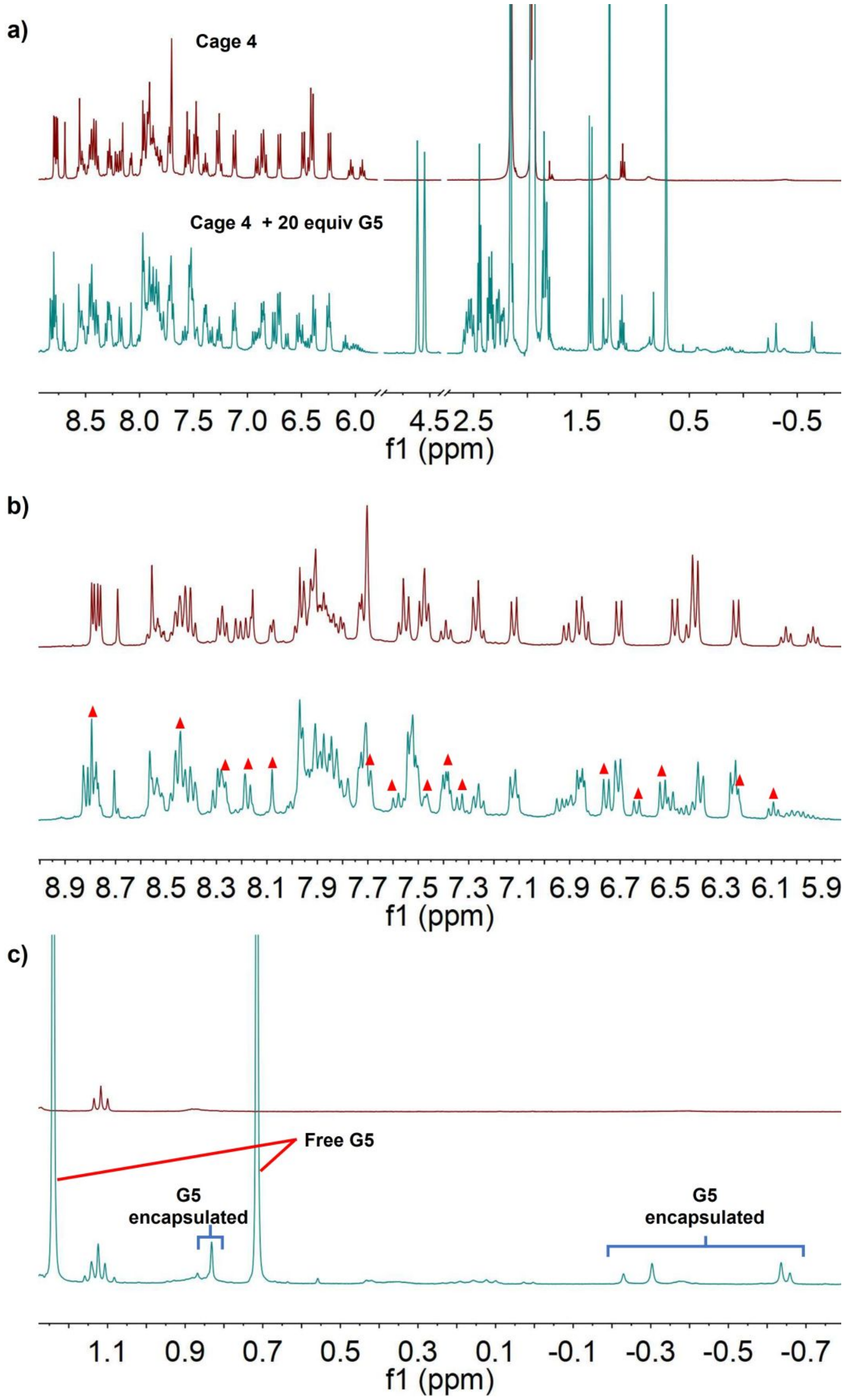

Figure S99: **a)** ^{1}H NMR (400 MHz, CD_3CN, 298 K) spectrum of cage **4** (1 mM) and cage **4** with 20 equiv **G5**; **b)** Aromatic ^{1}H NMR region of **a)**, a new set of small peaks was observed, suggesting a host-guest binding between **G5** and **4**; **c)** Zoom in of the ^{1}H NMR spectrum shown in **a)**. The observation of signals corresponding to both free **G5** and encapsulated **G5** indicated slow-exchange host-guest binding on the NMR timescale.

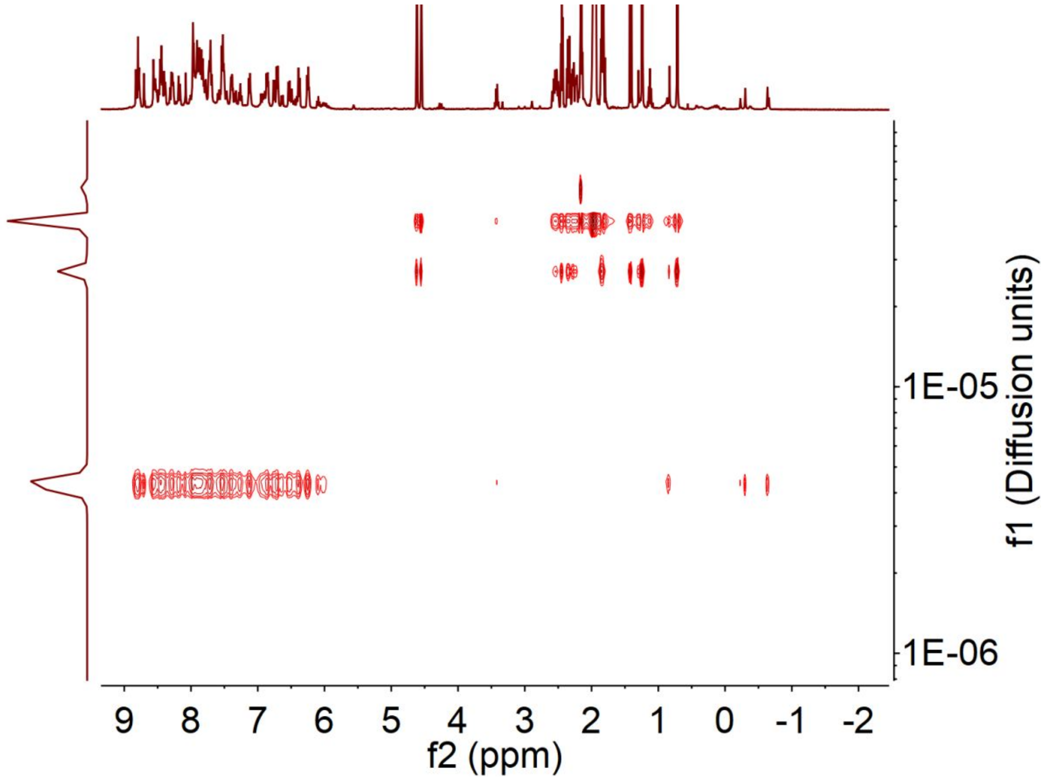

Figure S100: ^{1}H DOSY NMR spectrum of **G5⊂4** (400 MHz, CD_3CN, 298 K). The proton signals belong to encapsulated guest could be observed.

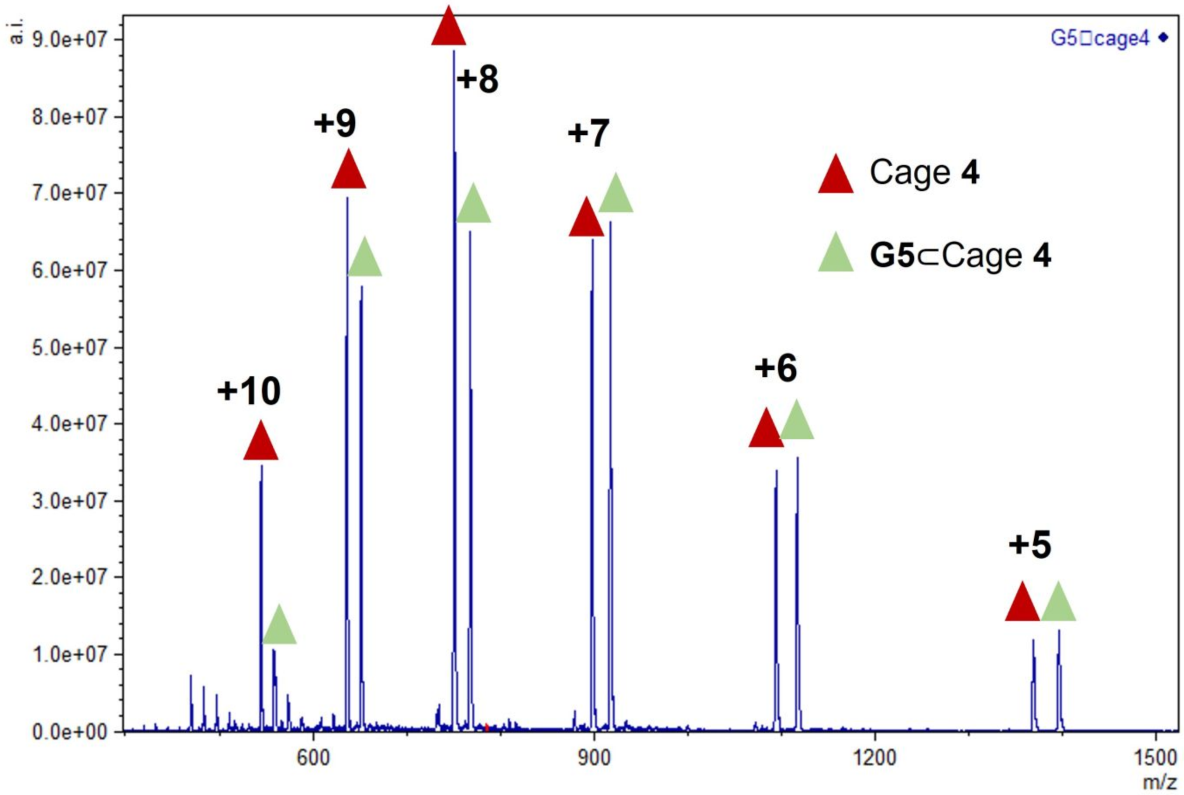

Figure S101: Low-resolution ESI-MS spectrum of **G5**⊂cage **4** in MeCN.

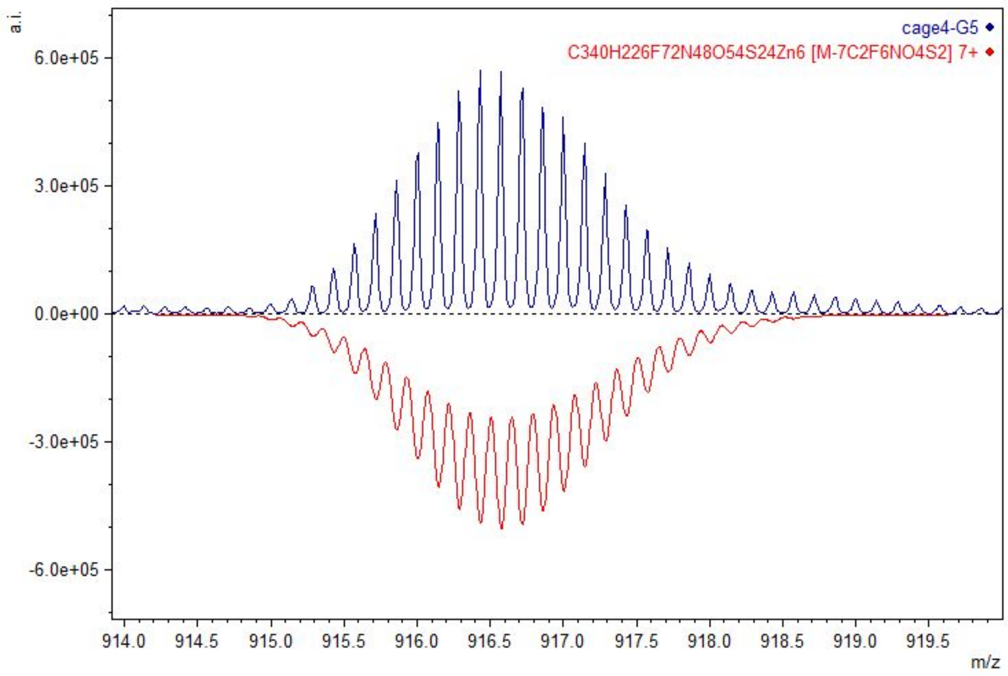

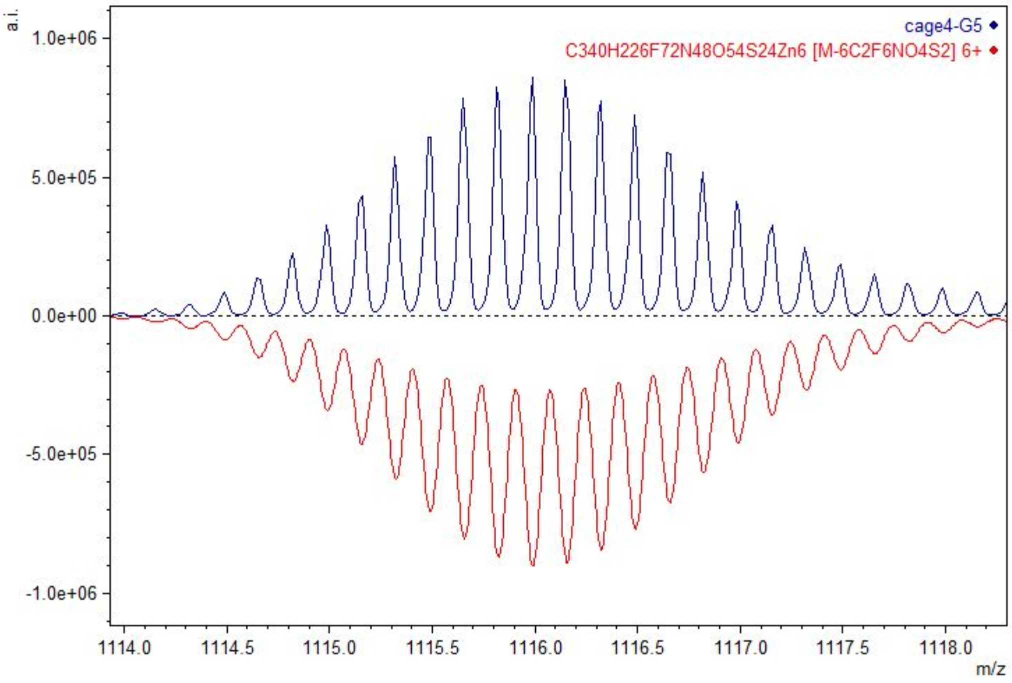

Figure S102: High-resolution ESI-mass spectrometry analysis of host-guest complex **G5**⊂cage **4** showing the observed (blue) and theoretical (red) isotope patterns for the +7 and +6 peaks

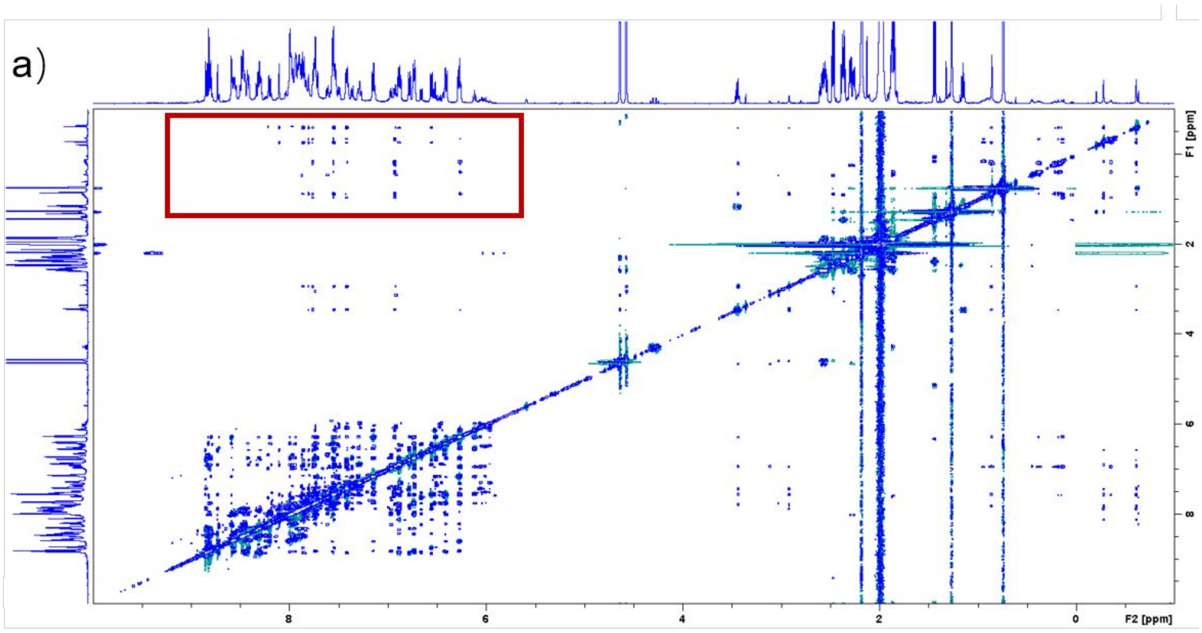

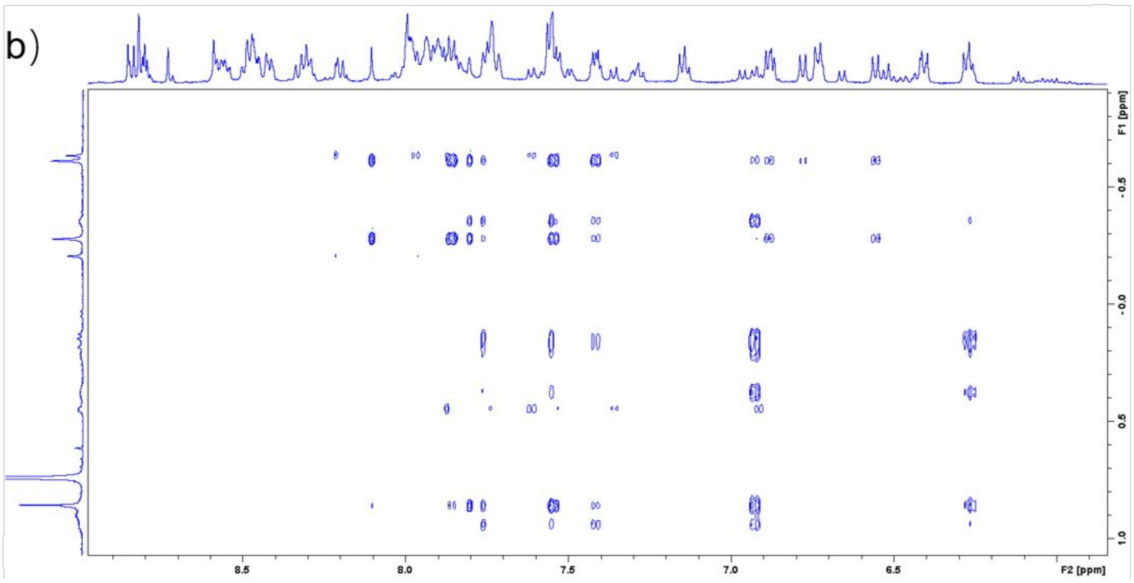

Figure S103: **a)** ¹H-¹H NOESY NMR spectrum of 2·**G5⊂4** (400 MHz, CD_3CN, 298 K); **b)** Partial ¹H-¹H NOESY NMR spectrum of 2·**G5⊂4.**

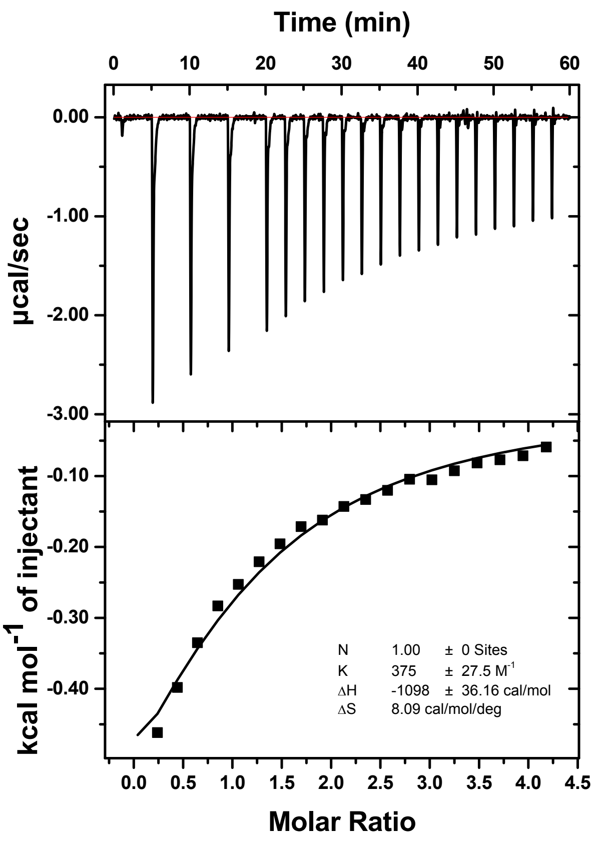

Figure S104: ITC experiments for cage **4** and **G5**. Titration plots (heat flow versus time and heat/mol versus guest/host ratio) were obtained by titrating a solution of the cage (2 mM) with the **G5** (20 mM) in acetonitrile. The line represents the best fit resulting from fitting using a one site binding model. The binding constant was calculated to be (3.75 ± 0.23) × 10^2 M^{-1}. Single guest binding was also supported by ESI-MS analysis, as shown in Figures S101 and S102. We infer that a putative second binding event between cage **4** and **G5** was too weak for ITC measurement, given the weakness of initial guest binding.

6.4 Host-guest interactions of cage 5

6.4.1 Host-guest interactions of cage 5 with hexadecahydropyrene (G6)

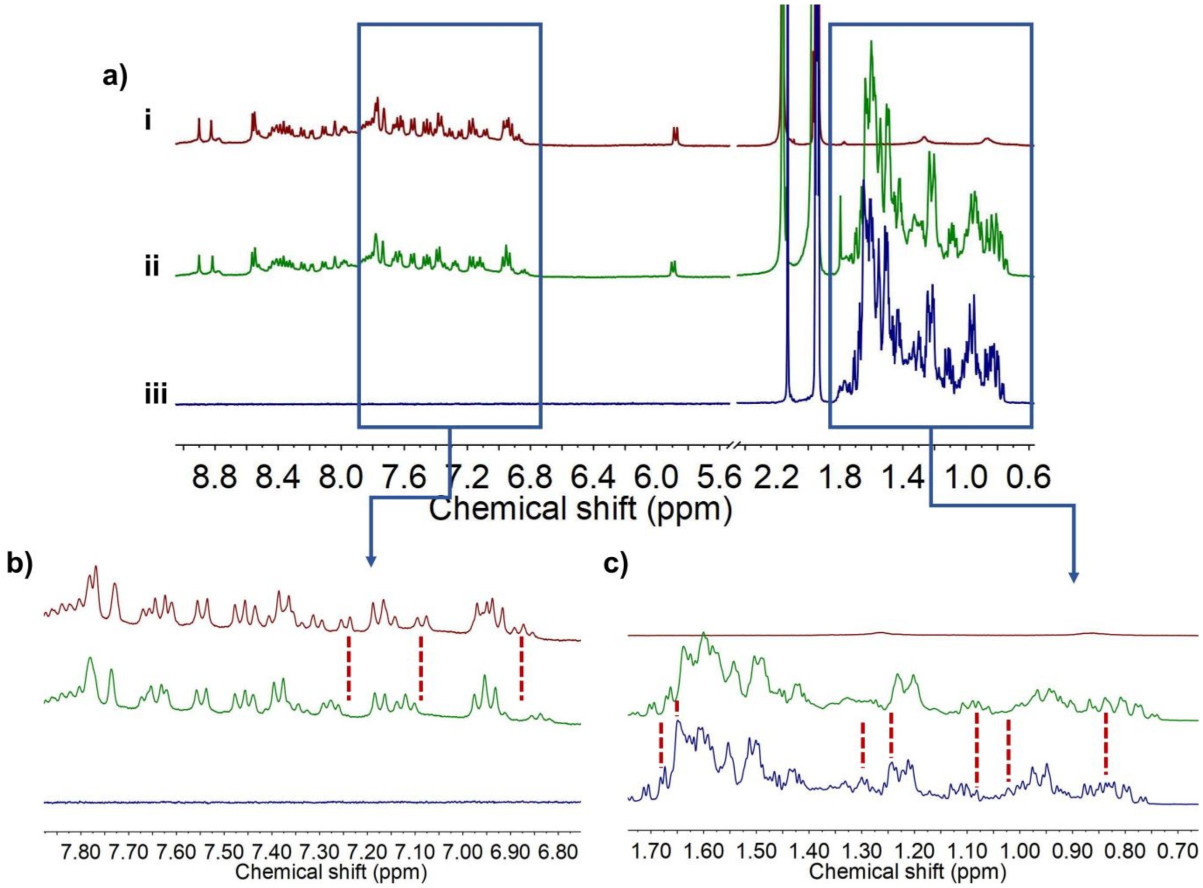

Figure S105: **a)** ^{1}H NMR (400 MHz, CD_3CN, 298 K) spectra of **i)** 1 mM cage **5**; **ii)** 1 mM cage **5** with 20 mM guest hexadecahydropyrene (**G6**); **iii)** 20 mM **G6**. **b)** Detailed ^{1}H NMR spectra of **a)** displaying the ^{1}H NMR shifts of cage **5** upon the addition **G6**. **c)** Detailed ^{1}H NMR spectra of **a)** displaying the ^{1}H NMR shifts of **G6** upon binding with cage **5**, indicating the formation of **G6⊂5**. The ^{1}H NMR shifts of both **G6** and **5** indicated the occurrence of fast exchange in the host-guest binding on the NMR timescale.

6.4.2 Cage 1 mixed with G6

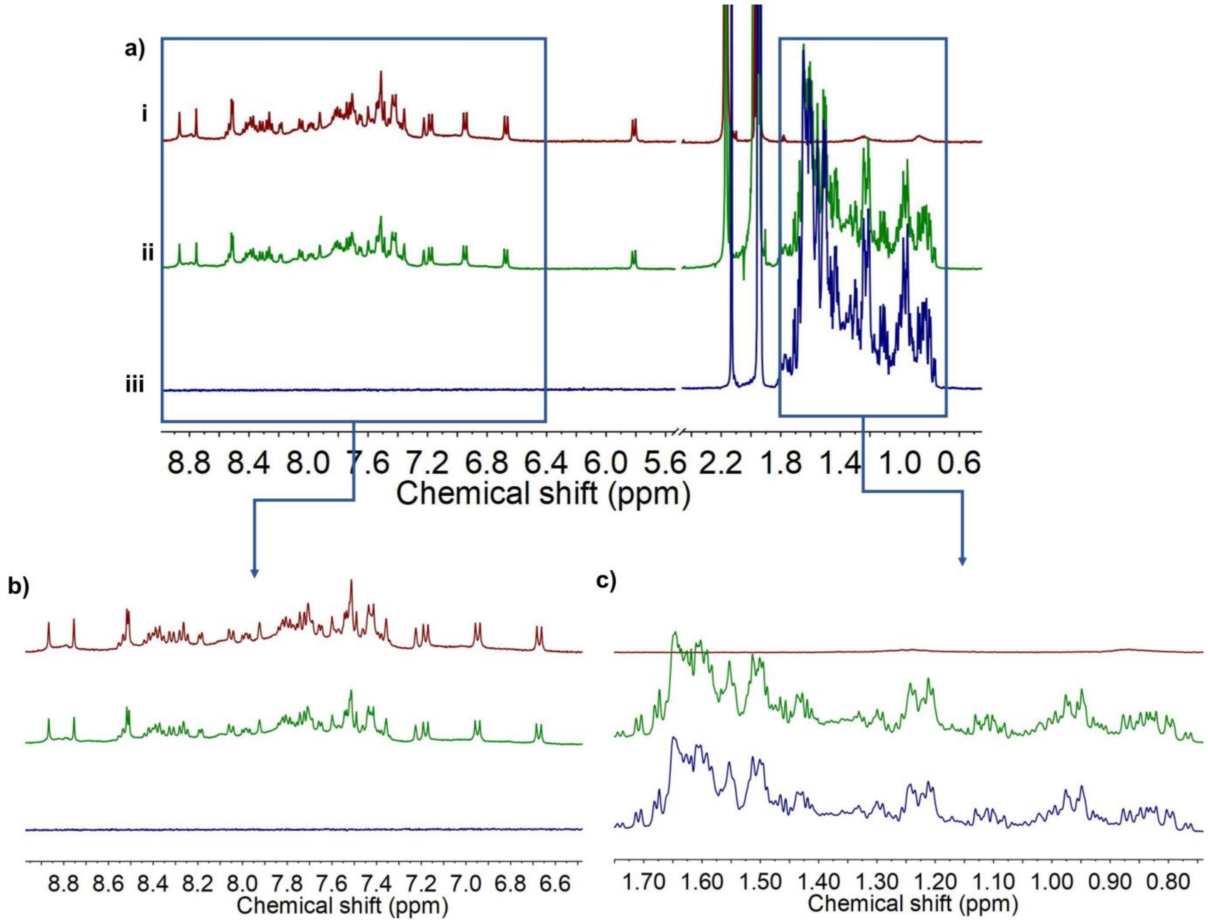

Figure S106: **a)** ^{1}H NMR (400 MHz, CD_3CN, 298 K) spectra of **i)** 1 mM cage **1**; **ii)** 1 mM cage **1** with 20 mM guest hexadecahydropyrene (**G6**); **iii)** 20 mM **G6**. **b)** Detailed ^{1}H NMR spectra of **a)**. No ^{1}H NMR shifts for cage **1** were observed in the presence of **G6**. **c)** Detailed ^{1}H NMR spectra of **a)**. No ^{1}H NMR shifts for **G6** were observed in the presence of cage **1**, indicating no host-guest binding between **G6** and **1**.

6.4.3 DOSY of G6⊂cage 5 and free G6

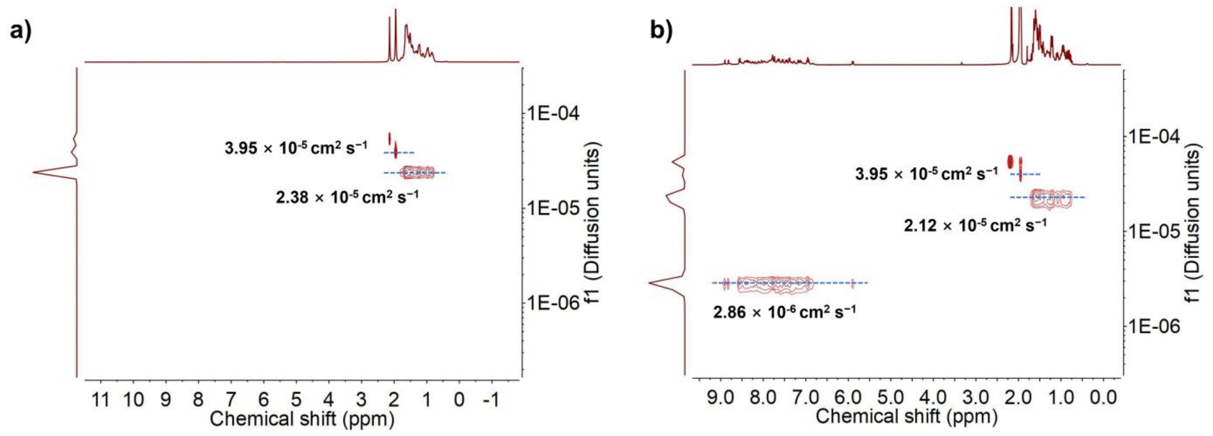

Figure S107: ^{1}H DOSY spectra (400 MHz, 298 K, CD_3CN) of **a)** 20 mM **G6** and **b)** 1 mM cage **5** and 20 mM **G6**. The diffusion coefficient for free **G6** was measured to be 2.38 × 10^{-5} cm^2 s^{-1}. The diffusion coefficient for **G6** in **b)** was measured to be 2.12 × 10^{-5} cm^2 s^{-1}, consistent with formation of a fast-exchange host-guest complex **G6⊂5**, as the value is slower than that for free **G6**. The diffusion coefficient for **5** in **b)** was measured to be 2.86 × 10^{-6} cm^2 s^{-1}, as noted in Figure S35. The diffusion coefficient of the solvent acetonitrile (3.95 × 10^{-5} cm^2 s^{-1}) in both **a)** and **b)** was used as the internal standard.

6.4.4 ESI-MS of G6⊂cage 5

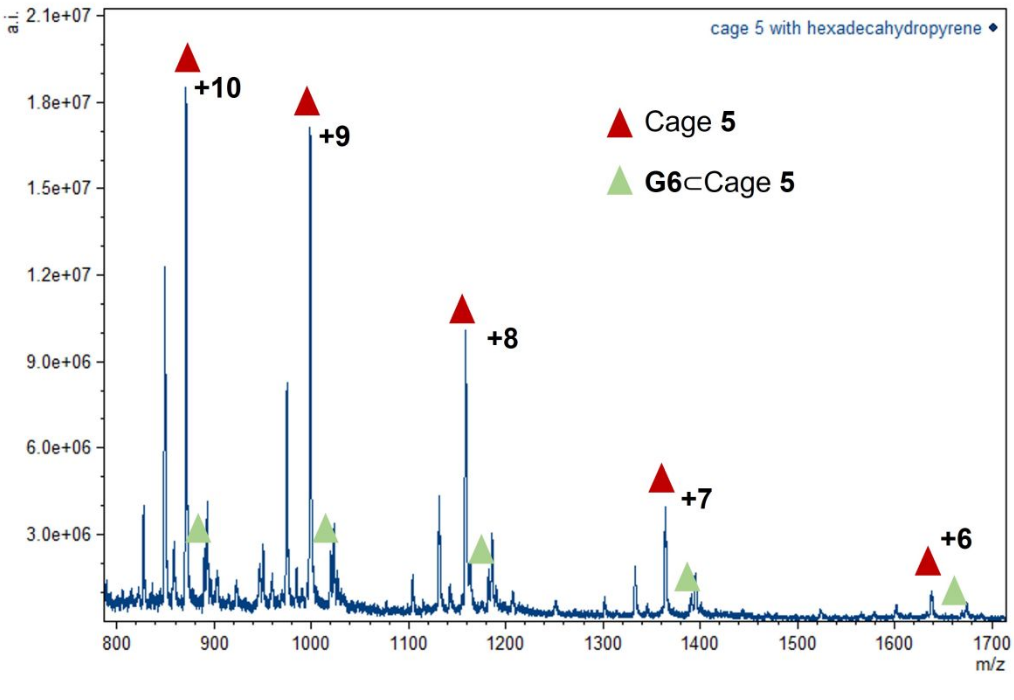

Figure S108: Low-resolution ESI-MS spectrum of cage **5** with **G6** in MeCN

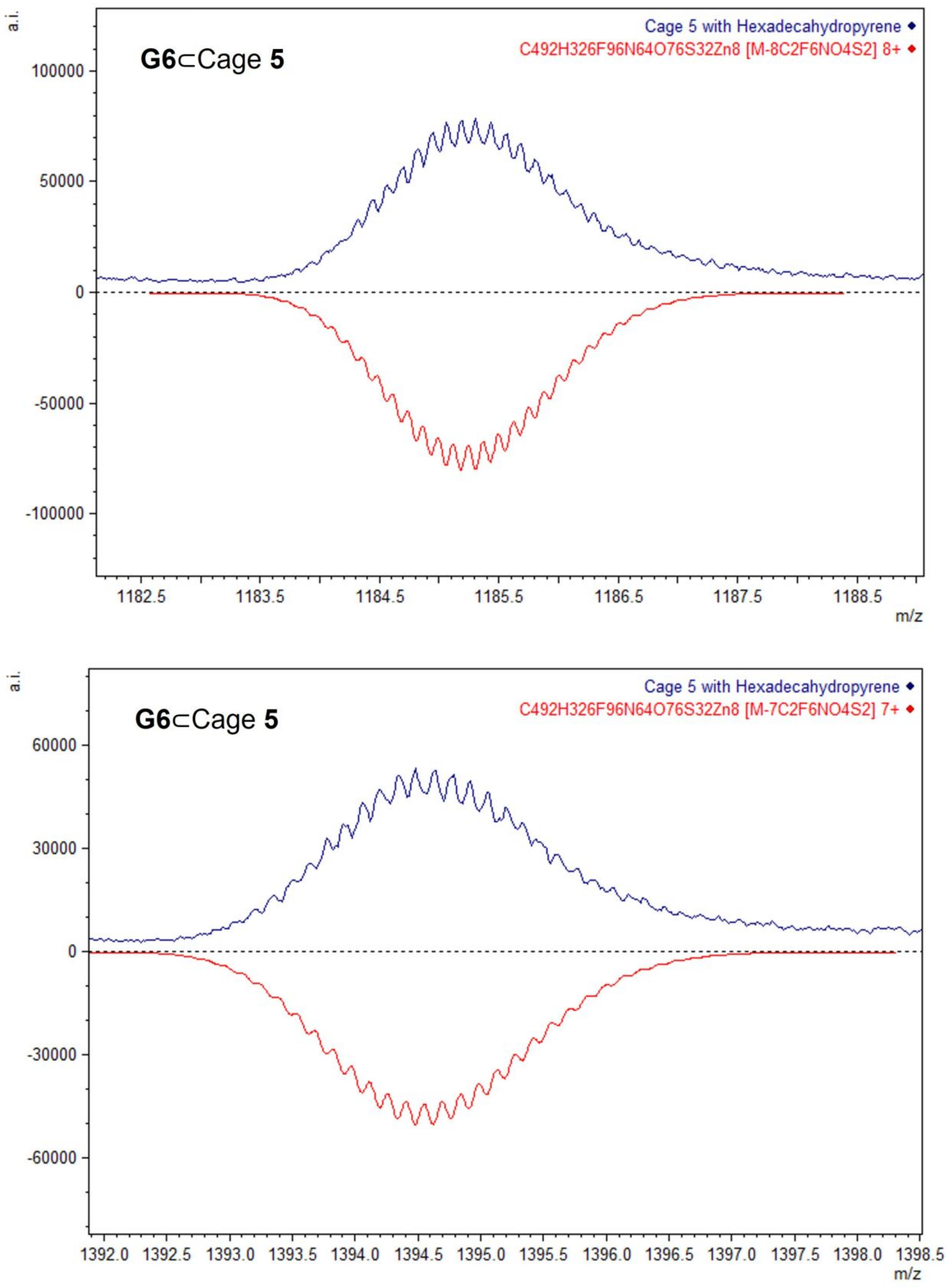

Figure S109: High-resolution ESI-mass spectrometric analysis of host-guest complex (1 equiv of **G6** encapsulated in **5**(NTf_2^-)$_{12}$) showing the observed (blue) and theoretical (red) isotope patterns for the +8 and +7 peaks.

6.4.5 ITC titration of guest G6 to cage 5

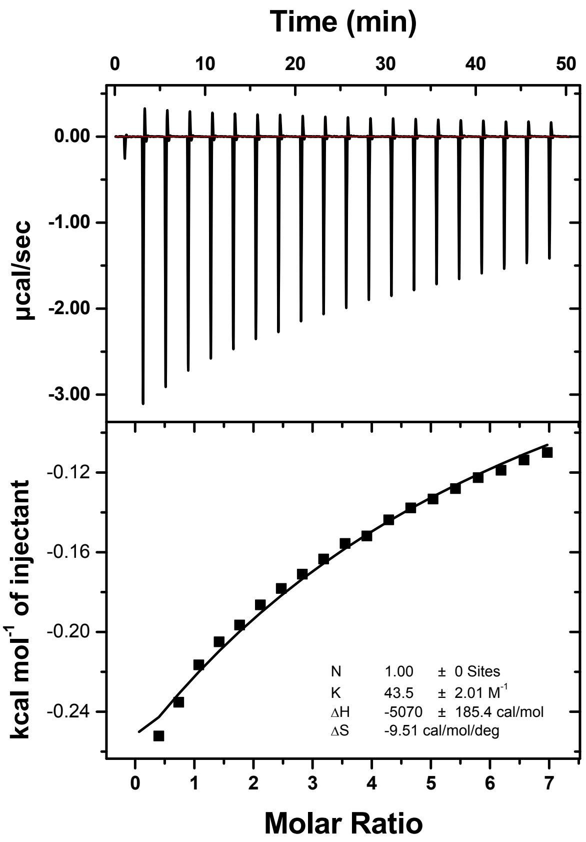

Figure S110: ITC experiments for cage **5** and **G6**. Titration plots (heat flow versus time and heat/mol versus guest/host ratio) were obtained by titrating a solution of the cage (1 mM) with the **G6** (40 mM) in acetonitrile. The line represents the best fit resulting from fitting using a one site binding model. The binding constant was calculated to be (4.35 ± 0.20) × 10^1 M^{-1}.

6.4.6 Uptake and release of G6

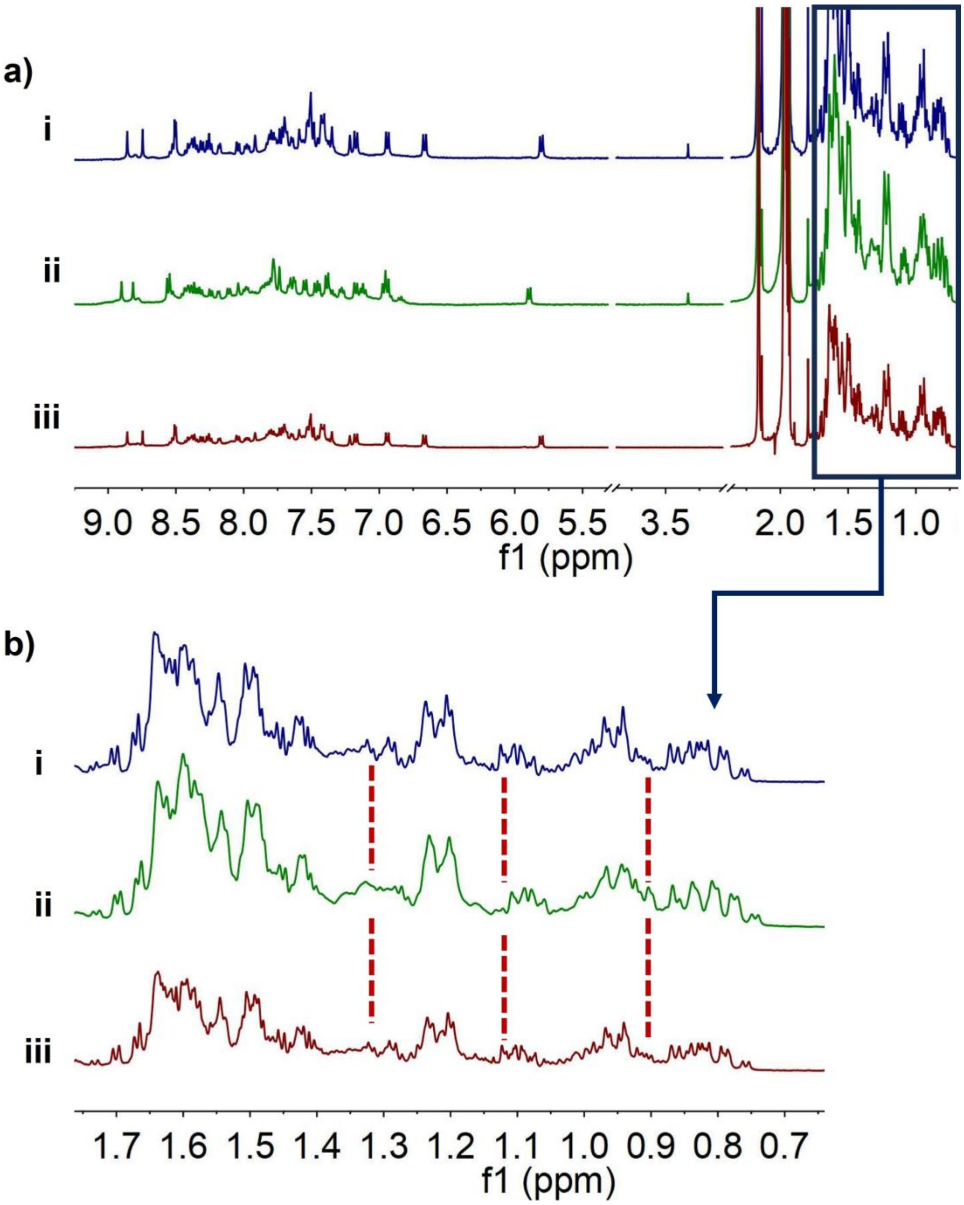

Figure S111: **a)** ^{1}H NMR (400 MHz, CD_3CN, 298 K) spectra of **i)** Thermally-reverted cage **1** in the presence of guest **G6**; **ii)** cage **5** with guest **G6**; **iii)** cage **1** with guest **G6**. **b)** Partial ^{1}H NMR (400 MHz, CD_3CN, 298 K) spectra of **a)**. After heating, the hetero-Diels-Alder reaction reversed to produce parent cage **1**, which released **G6**, closing the loop of reversible host-guest binding.

7 DFT calculations

7.1 General procedure

Geometries of cages **1**-**5** were first optimized using the semi-empirical method PM6 with Grimme-D3 type dispersion correction. To achieve higher accuracy, the geometries were further optimized using density functional theory (DFT). Density functional B3LYP[4] was employed. Los Alamos Effective Core Potential (LANL2DZ)[5] was used for the metal ion, Zn^{II}, while a Pople type basis set, 6-31G(d)[6, 7] was adopted for all other atoms. All quantum mechanical calculations were carried out using the Gaussian16 program suite[8]. Geometry optimizations and the subsequent vibrational analyses were performed in the gas phase.

7.2 Structural optimization of cage 1-5

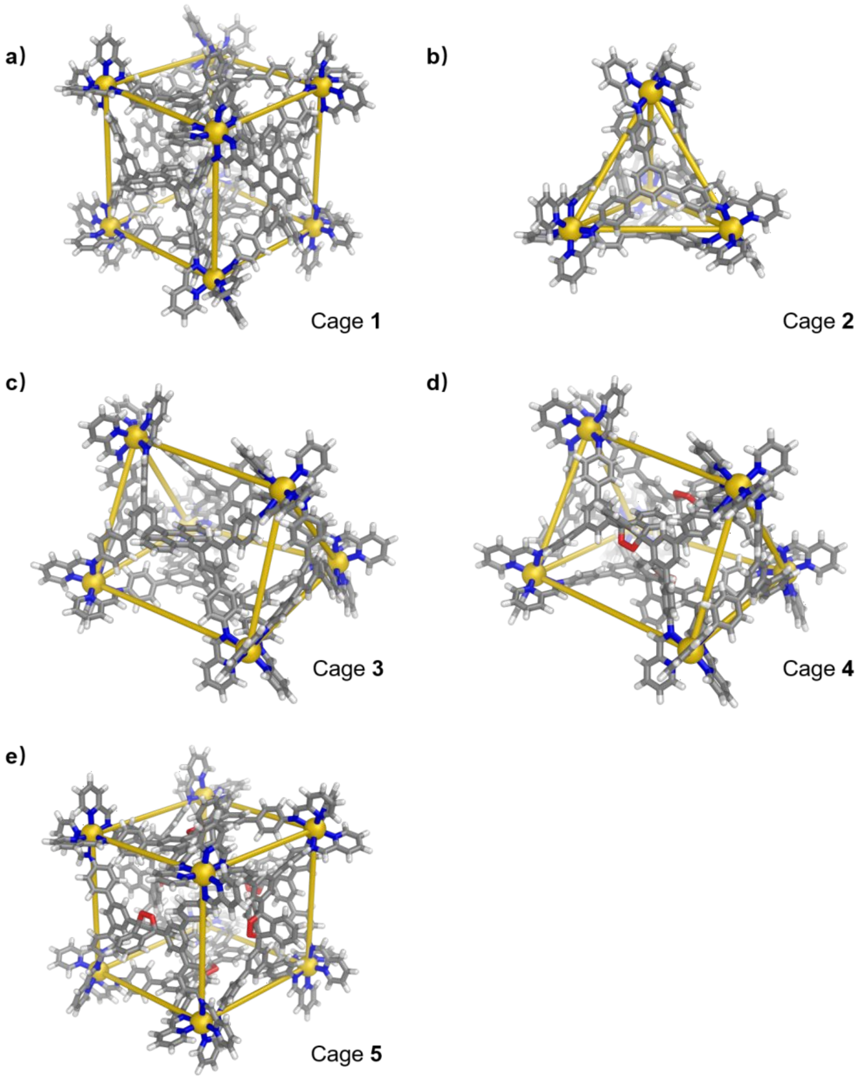

Figure S112. DFT model structure of **a)** cage **1**; **b)** cage **2**; **c)** cage **3**; **d)** cage **4**; **e)** cage **5**.

Table S1. Cartesian coordinates (in Å) for the B3LYP 6-31G(d) model of **1**.

Zn	4.99422	-4.01931	12.52136
Zn	4.97626	-8.83933	-9.74641
Zn	14.22627	-0.00226	-0.01043
Zn	4.98472	12.85632	-2.78687
N	-6.10550	-13.35084	4.66566
N	-3.85843	-11.70206	4.45004
N	3.73997	-4.81244	14.20550
N	3.45516	-5.42120	11.49167
N	15.54410	-1.05513	1.50607
N	13.19056	-2.04799	0.37200
N	6.48455	-8.23794	-11.29821
N	6.53957	-7.55464	-8.58457
C	-7.15991	-14.16421	4.81073
H	-7.55493	-14.61041	3.90381
C	-7.73584	-14.45265	6.05412
H	-8.58488	-15.12590	6.11447
C	-7.19240	-13.86948	7.19631
H	-7.60758	-14.07745	8.17775
C	-6.09473	-13.01548	7.05290
H	-5.63913	-12.54691	7.91988
C	-5.57887	-12.77985	5.77572
C	-4.41042	-11.91343	5.59697
H	-3.99748	-11.46690	6.50469
C	-2.73534	-10.81715	4.42262
C	-1.57289	-11.17839	3.72708
H	-1.53731	-12.12793	3.20449
C	-0.43819	-10.36863	3.77985
H	0.47119	-10.70143	3.28782
C	-0.42900	-9.16623	4.50952
C	-1.62103	-8.79062	5.15472
H	-1.66432	-7.84589	5.68854
C	-2.75556	-9.59642	5.11677
H	-3.66812	-9.26791	5.60551
C	3.85195	-4.54943	15.51399
H	4.58443	-3.80056	15.79603
C	3.08502	-5.19636	16.49138
H	3.22002	-4.94578	17.53863
C	2.16388	-6.16142	16.09169
H	1.55968	-6.69004	16.82273
C	2.03599	-6.43791	14.72685
H	1.33072	-7.18510	14.37577
C	2.83623	-5.74424	13.81484
C	2.72385	-6.00988	12.37826
H	1.98127	-6.75459	12.08161
C	3.17682	-5.72328	10.12285
C	1.85466	-5.75713	9.64951
H	1.03906	-5.45896	10.30144
C	1.58397	-6.13986	8.34049
H	0.55409	-6.13623	7.99740
C	2.61069	-6.51802	7.45623
C	3.93355	-6.43089	7.92913
H	4.75432	-6.72927	7.28387
C	4.21787	-6.02510	9.23333
H	5.24436	-6.00963	9.58347
C	16.65936	-0.60793	2.09835
H	16.92472	0.42719	1.91362
C	17.47257	-1.41295	2.90617
H	18.36984	-0.99799	3.35403
C	17.11357	-2.74341	3.10668
H	17.72670	-3.39980	3.71677
C	15.94610	-3.21708	2.49984
H	15.63058	-4.24775	2.63102
C	15.18868	-2.34610	1.71250
C	13.95420	-2.81284	1.07402

H	13.68754	-3.85943	1.24240	H	0.52032	-8.06415	6.75679
C	12.02051	-2.67529	-0.17830	C	2.28494	-7.02320	6.09289
C	10.74816	-2.21911	0.19110	C	3.10578	-6.76500	4.98162
H	10.65334	-1.35703	0.84494	H	3.99002	-6.14450	5.09633
C	9.61266	-2.91777	-0.22052	C	2.79827	-7.27339	3.70968
H	8.63470	-2.57072	0.10108	C	1.64454	-8.05805	3.55513
C	9.70497	-4.06259	-1.03394	H	1.39570	-8.44250	2.56981
C	10.98607	-4.46439	-1.44784	C	0.79727	-8.32909	4.64358
H	11.09826	-5.34629	-2.07160	C	3.68812	-6.95910	2.53608
C	12.13083	-3.79113	-1.02015	C	3.43888	-5.81394	1.74606
H	13.11155	-4.14140	-1.32968	C	2.32857	-4.94516	2.00423
C	6.48604	-8.52991	-12.60566	H	1.66366	-5.18514	2.82679
H	5.63964	-9.09874	-12.97540	C	2.09410	-3.83270	1.23972
C	7.51692	-8.14843	-13.47308	H	1.24252	-3.19386	1.45878
H	7.47072	-8.42220	-14.52216	C	2.96218	-3.50892	0.16024
C	8.59301	-7.42837	-12.95995	H	2.77004	-2.62416	-0.44116
H	9.41424	-7.12323	-13.60128	C	4.03872	-4.30780	-0.12213
C	8.59553	-7.11151	-11.59846	H	4.69866	-4.05597	-0.94549
H	9.41775	-6.55401	-11.16025	C	4.32298	-5.48421	0.64544
C	7.52791	-7.53086	-10.79936	C	5.43508	-6.30717	0.35954
C	7.51150	-7.22289	-9.36746	C	5.67251	-7.46526	1.13565
H	8.37640	-6.67596	-8.98479	C	6.77833	-8.33809	0.87206
C	6.68248	-7.26418	-7.19411	H	7.44444	-8.10162	0.04991
C	7.91986	-7.39960	-6.53908	C	7.00529	-9.45646	1.63013
H	8.77099	-7.82603	-7.06194	H	7.84915	-10.10331	1.40905
C	8.05647	-7.03021	-5.20468	C	6.13859	-9.77882	2.71125
H	9.01793	-7.16575	-4.71881	H	6.33000	-10.66810	3.30455
C	6.97140	-6.51882	-4.46923	C	5.06917	-8.97369	3.00258
C	5.72584	-6.44284	-5.12063	H	4.41322	-9.22713	3.82817
H	4.86586	-6.05045	-4.58545	C	4.78827	-7.79479	2.23752
C	5.57612	-6.81665	-6.45679	C	6.39674	-5.93103	-0.73658
H	4.61283	-6.70889	-6.94562	C	7.53097	-5.16483	-0.42936
C	1.13454	-7.80474	5.90021	H	7.68642	-4.84632	0.59745

C	8.49246	-4.84918	-1.40422
C	8.29453	-5.31482	-2.71297
H	9.01408	-5.05342	-3.48378
C	7.15705	-6.06365	-3.06114
C	6.21540	-6.36197	-2.05951
H	5.34873	-6.97050	-2.30254
N	-6.09828	10.71342	9.23719
N	-3.85185	9.70401	7.91454
N	3.72924	14.71074	-2.94029
N	3.44775	12.66478	-1.05572
N	15.54434	1.83758	0.14160
N	13.19228	1.35170	1.57088
N	6.50452	-5.66224	12.77482
N	6.55579	-3.65359	10.82609
C	-7.15242	11.24490	9.87021
H	-7.54693	10.68163	10.70969
C	-7.72888	12.46614	9.49983
H	-8.57767	12.85429	10.05360
C	-7.18626	13.16488	8.42402
H	-7.60182	14.11904	8.11460
C	-6.08894	12.61451	7.75488
H	-5.63404	13.13190	6.91575
C	-5.57254	11.39033	8.18792
C	-4.40463	10.80302	7.52536
H	-3.99274	11.36648	6.68470
C	-2.72961	9.23848	7.16026
C	-1.56627	8.81648	7.81915
H	-1.52933	8.83800	8.90273
C	-0.43253	8.45769	7.08982
H	0.47753	8.19763	7.62271
C	-0.42522	8.48906	5.68369
C	-1.61812	8.86028	5.03754
H	-1.66288	8.85058	3.95252

C	-2.75169	9.22997	5.75601
H	-3.66500	9.48909	5.22851
C	3.83993	15.71264	-3.82223
H	4.57169	15.58292	-4.61251
C	3.07251	16.88217	-3.74975
H	3.20642	17.66402	-4.49037
C	2.15224	17.01791	-2.71327
H	1.54769	17.91501	-2.62029
C	2.02572	15.97396	-1.79146
H	1.32119	16.04303	-0.96816
C	2.82642	14.83773	-1.93708
C	2.71565	13.72635	-0.98866
H	1.97363	13.84124	-0.19480
C	3.17105	11.63017	-0.10948
C	1.84943	11.23653	0.15824
H	1.03283	11.65167	-0.42480
C	1.58055	10.29399	1.14444
H	0.55105	9.99450	1.31418
C	2.60860	9.71761	1.91254
C	3.93087	10.08409	1.59874
H	4.75267	9.67463	2.17844
C	4.21338	11.01094	0.59496
H	5.23948	11.30667	0.40479
C	16.65911	2.12670	-0.54273
H	16.92387	1.44894	-1.34683
C	17.47259	3.22882	-0.25030
H	18.36948	3.40898	-0.83423
C	17.11437	4.06797	0.80172
H	17.72774	4.92453	1.06451
C	15.94739	3.77949	1.51625
H	15.63252	4.40862	2.34335
C	15.18968	2.66207	1.15647
C	13.95586	2.34252	1.88105

H	13.68975	3.01184	2.70325
C	12.02325	1.18862	2.39070
C	12.13537	1.01792	3.77786
H	13.11670	0.92571	4.23480
C	10.99160	0.98388	4.57617
H	11.10515	0.88503	5.65170
C	9.70978	1.14066	4.02276
C	9.61568	1.27224	2.62468
H	8.63709	1.37647	2.16448
C	10.75018	1.27967	1.81243
H	10.65398	1.41433	0.73897
C	6.50798	-6.64849	13.68145
H	5.66228	-6.68516	14.35979
C	7.53993	-7.58937	13.78377
H	7.49531	-8.36103	14.54548
C	8.61500	-7.50397	12.90249
H	9.43700	-8.21112	12.95806
C	8.61548	-6.48338	11.94726
H	9.43686	-6.38184	11.24448
C	7.54690	-5.58274	11.91193
C	7.52854	-4.49664	10.92930
H	8.39273	-4.43793	10.26345
C	6.69676	-2.59450	9.87924
C	5.58920	-2.18054	9.12432
H	4.62645	-2.65854	9.27643
C	5.73706	-1.21019	8.13234
H	4.87621	-0.94359	7.52586
C	6.98188	-0.60714	7.87104
C	8.06814	-0.98748	8.68044
H	9.02910	-0.49823	8.55379
C	7.93339	-1.95850	9.66769
H	8.78537	-2.19747	10.29751
C	1.13549	9.01305	3.80704
H	0.52084	9.88450	3.60438
C	2.28478	8.78939	3.03213
C	3.10631	7.69806	3.36294
H	3.98971	7.48729	2.76692
C	2.80058	6.85071	4.43970
C	1.64788	7.10887	5.19815
H	1.40039	6.44766	6.02408
C	0.79994	8.18684	4.88991
C	3.69142	5.67767	4.75324
C	4.79291	5.83782	5.62438
C	5.07421	7.09018	6.26194
H	4.41752	7.93148	6.06944
C	6.14490	7.24127	7.10311
H	6.33655	8.19996	7.57601
C	7.01259	6.14435	7.36346
H	7.85745	6.27696	8.03280
C	6.78529	4.92835	6.77473
H	7.45215	4.09851	6.98023
C	5.67816	4.71936	5.88889
C	5.44034	3.46784	5.27485
C	4.32695	3.30312	4.42102
C	4.04244	2.04989	3.78669
H	4.70319	1.21136	3.97936
C	2.96468	1.89419	2.95537
H	2.77241	0.93079	2.49029
C	2.09556	2.99038	2.69716
H	1.24304	2.86003	2.03571
C	2.33024	4.20897	3.27768
H	1.66458	5.04091	3.07503
C	3.44182	4.42059	4.15735
C	6.40281	2.33101	5.49607
C	7.53608	2.21440	4.67753
H	7.69022	2.94434	3.88799

C	8.49829	1.21279	4.89055	C	-1.58189	2.36267	-11.54092
C	8.30211	0.31228	5.94856	H	-1.54567	3.29000	-12.10194
H	9.02227	-0.48555	6.10688	C	3.82956	-11.16399	-11.70144
C	7.16559	0.38471	6.77249	H	4.56281	-11.78323	-11.19571
C	6.22315	1.40080	6.53106	C	3.06058	-11.68597	-12.74940
H	5.35726	1.49430	7.18065	H	3.19469	-12.71802	-13.05702
N	-6.11687	2.63971	-13.88764	C	2.13854	-10.85654	-13.38303
N	-3.86855	2.00074	-12.35413	H	1.53270	-11.22455	-14.20549
N	3.71873	-9.89959	-11.27383	C	2.01191	-9.53656	-12.93891
N	3.43648	-7.24546	-10.44178	H	1.30602	-8.85836	-13.40870
N	15.54226	-0.79042	-1.68132	C	2.81421	-9.09459	-11.88330
N	13.19004	0.69016	-1.97259	C	2.70346	-7.71786	-11.39410
N	6.49595	13.89778	-1.49225	H	1.96046	-7.08783	-11.88910
N	6.54758	11.20566	-2.25724	C	3.15994	-5.90882	-10.01827
C	-7.17217	2.92128	-14.66316	C	1.83842	-5.48077	-9.80939
H	-7.56705	3.92977	-14.59501	H	1.02208	-6.19368	-9.87632
C	-7.74919	1.98923	-15.53450	C	1.56935	-4.15554	-9.48586
H	-8.59892	2.27398	-16.14657	H	0.53995	-3.85919	-9.31005
C	-7.20588	0.70848	-15.60191	C	2.59712	-3.20169	-9.37233
H	-7.62182	-0.03706	-16.27274	C	3.91937	-3.65605	-9.53467
C	-6.10740	0.40510	-14.79193	H	4.74090	-2.94885	-9.47110
H	-5.65200	-0.58007	-14.82060	C	4.20211	-4.98866	-9.83574
C	-5.59052	1.39283	-13.94929	H	5.22815	-5.30063	-9.99838
C	-4.42161	1.11365	-13.11047	C	16.65692	-1.52794	-1.59080
H	-4.00920	0.10411	-13.17828	H	16.92246	-1.88587	-0.60217
C	-2.74524	1.58097	-11.57496	C	17.46938	-1.82553	-2.69230
C	-2.76624	0.36946	-10.86482	H	18.36622	-2.42161	-2.55736
H	-3.67944	-0.21700	-10.82391	C	17.11027	-1.33336	-3.94452
C	-1.63168	-0.06758	-10.18687	H	17.72288	-1.53382	-4.81831
H	-1.67558	-1.00218	-9.63553	C	15.94342	-0.56994	-4.05065
C	-0.43882	0.67765	-10.19053	H	15.62785	-0.16772	-5.00859
C	-0.44716	1.91078	-10.86697	C	15.18671	-0.32317	-2.90230
H	0.46283	2.50232	-10.90979	C	13.95283	0.46410	-2.98651

H	13.68590	0.84192	-3.97687	H	0.50747	-1.82078	-10.36006
C	12.02044	1.48132	-2.23970	C	2.27308	-1.76808	-9.12798
C	12.13123	2.76810	-2.78551	C	3.09546	-0.93564	-8.34949
H	13.11216	3.21038	-2.93540	H	3.97966	-1.34617	-7.87020
C	10.98665	3.47604	-3.15352	C	2.78973	0.42051	-8.15392
H	11.09913	4.45699	-3.60574	C	1.63616	0.94792	-8.75536
C	9.70525	2.91785	-3.01076	H	1.38864	1.99378	-8.59560
C	9.61251	1.64122	-2.42564	C	0.78733	0.14166	-9.53348
H	8.63430	1.19016	-2.28447	C	3.68145	1.27889	-7.29609
C	10.74787	0.93449	-2.02767	C	4.78040	1.95570	-7.87235
H	10.65269	-0.06260	-1.60761	C	5.05854	1.88341	-9.27642
C	6.49925	15.17606	-1.09146	H	4.40140	1.29545	-9.90775
H	5.65282	15.78138	-1.39780	C	6.12692	2.53852	-9.82974
C	7.53190	15.73567	-0.32914	H	6.31629	2.46989	-10.89693
H	7.48708	16.78113	-0.04164	C	6.99531	3.31304	-9.01125
C	8.60787	14.93032	0.03614	H	7.83840	3.82803	-9.46222
H	9.43041	15.33246	0.61970	C	6.77104	3.40952	-7.66317
C	8.60852	13.59278	-0.37011	H	7.43852	4.00271	-7.04821
H	9.43059	12.93381	-0.10773	C	5.66641	2.74453	-7.03731
C	7.53918	13.11127	-1.13098	C	5.43193	2.83649	-5.64575
C	7.52090	11.71717	-1.58003	C	4.32110	2.17690	-5.07413
H	8.38576	11.11165	-1.29897	C	4.03974	2.25243	-3.67090
C	6.68881	9.85612	-2.70097	H	4.70092	2.83919	-3.04208
C	7.92526	9.35550	-3.14714	C	2.96435	1.60804	-3.11845
H	8.77680	10.02083	-3.25603	H	2.77444	1.68570	-2.05107
C	8.06040	8.01502	-3.49434	C	2.09452	0.83574	-3.93737
H	9.02117	7.66115	-3.85564	H	1.24380	0.32639	-3.49226
C	6.97470	7.12330	-3.41771	C	2.32612	0.73096	-5.28363
C	5.73001	7.65050	-3.02487	H	1.65979	0.13921	-5.90181
H	4.86959	6.99153	-2.95149	C	3.43520	1.38930	-5.90872
C	5.58175	8.99475	-2.68066	C	6.39536	3.59704	-4.77336
H	4.61915	9.36504	-2.34185	C	7.52966	2.94702	-4.26471
C	1.12289	-1.20922	-9.70771	H	7.68392	1.89833	-4.50220

C	8.49273	3.63283	-3.50536	H	-0.47138	10.70196	-3.28813
C	8.29633	4.99929	-3.25435	C	0.42877	9.16674	-4.50982
H	9.01719	5.53571	-2.64374	C	1.62080	8.79108	-5.15499
C	7.15881	5.67606	-3.72746	H	1.66406	7.84634	-5.68882
C	6.21557	4.95842	-4.48523	C	2.75536	9.59682	-5.11701
H	5.34882	5.47381	-4.88970	H	3.66792	9.26827	-5.60572
Zn	-4.99416	4.01923	-12.52146	C	-3.85195	4.54923	-15.51413
Zn	-4.97622	8.83921	9.74637	H	-4.58435	3.80026	-15.79612
Zn	-14.22613	0.00224	0.01041	C	-3.08509	5.19616	-16.49158
Zn	-4.98466	-12.85636	2.78686	H	-3.22005	4.94549	-17.53881
N	6.10560	13.35091	-4.66562	C	-2.16405	6.16136	-16.09197
N	3.85833	11.70238	-4.45018	H	-1.55990	6.68998	-16.82305
N	-3.74001	4.81236	-14.20567	C	-2.03619	6.43797	-14.72714
N	-3.45533	5.42143	-11.49190	H	-1.33100	7.18527	-14.37613
N	-15.54402	1.05497	-1.50614	C	-2.83636	5.74430	-13.81508
N	-13.19054	2.04801	-0.37212	C	-2.72404	6.01008	-12.37853
N	-6.48458	8.23780	11.29810	H	-1.98150	6.75485	-12.08193
N	-6.53966	7.55479	8.58437	C	-3.17701	5.72362	-10.12309
C	7.16007	14.16421	-4.81060	C	-1.85485	5.75751	-9.64974
H	7.55514	14.61028	-3.90363	H	-1.03924	5.45931	-10.30165
C	7.73605	14.45272	-6.05396	C	-1.58419	6.14032	-8.34074
H	8.58514	15.12590	-6.11423	H	-0.55431	6.13671	-7.99764
C	7.19255	13.86971	-7.19621	C	-2.61092	6.51849	-7.45651
H	7.60777	14.07773	-8.17763	C	-3.93377	6.43132	-7.92942
C	6.09481	13.01580	-7.05289	H	-4.75456	6.72972	-7.28418
H	5.63918	12.54735	-7.91992	C	-4.21808	6.02547	-9.23360
C	5.57892	12.78009	-5.77574	H	-5.24456	6.00995	-9.58375
C	4.41038	11.91376	-5.59708	C	-16.65925	0.60767	-2.09841
H	3.99744	11.46733	-6.50485	H	-16.92455	-0.42745	-1.91364
C	2.73518	10.81755	-4.42285	C	-17.47251	1.41261	-2.90627
C	1.57272	11.17884	-3.72734	H	-18.36974	0.99757	-3.35411
H	1.53718	12.12836	-3.20473	C	-17.11359	2.74309	-3.10683
C	0.43799	10.36913	-3.78014	H	-17.72675	3.39942	-3.71695

C	-15.94615	3.21685	-2.50000
H	-15.63069	4.24753	-2.63123
C	-15.18867	2.34595	-1.71263
C	-13.95422	2.81279	-1.07417
H	-13.68762	3.85939	-1.24259
C	-12.02052	2.67541	0.17814
C	-10.74814	2.21930	-0.19127
H	-10.65328	1.35721	-0.84509
C	-9.61268	2.91804	0.22032
H	-8.63471	2.57105	-0.10129
C	-9.70505	4.06287	1.03372
C	-10.98616	4.46460	1.44763
H	-11.09840	5.34651	2.07138
C	-12.13089	3.79126	1.01997
H	-13.11163	4.14147	1.32950
C	-6.48607	8.52966	12.60557
H	-5.63963	9.09840	12.97536
C	-7.51698	8.14817	13.47295
H	-7.47077	8.42185	14.52205
C	-8.59310	7.42822	12.95975
H	-9.41435	7.12307	13.60105
C	-8.59563	7.11147	11.59823
H	-9.41787	6.55405	11.15996
C	-7.52798	7.53083	10.79917
C	-7.51158	7.22298	9.36725
H	-8.37649	6.67609	8.98454
C	-6.68258	7.26441	7.19390
C	-7.91998	7.39983	6.53890
H	-8.77111	7.82619	7.06180
C	-8.05660	7.03050	5.20449
H	-9.01808	7.16602	4.71865
C	-6.97153	6.51918	4.46899
C	-5.72595	6.44322	5.12035
H	-4.86596	6.05089	4.58514
C	-5.57622	6.81697	6.45653
H	-4.61291	6.70922	6.94534
C	-1.13479	7.80527	-5.90051
H	-0.52057	8.06465	-6.75709
C	-2.28519	7.02372	-6.09319
C	-3.10603	6.76553	-4.98191
H	-3.99027	6.14502	-5.09662
C	-2.79853	7.27394	-3.70997
C	-1.64480	8.05860	-3.55544
H	-1.39595	8.44305	-2.57011
C	-0.79752	8.32963	-4.64388
C	-3.68836	6.95964	-2.53637
C	-3.43909	5.81447	-1.74635
C	-2.32876	4.94572	-2.00455
H	-1.66386	5.18572	-2.82711
C	-2.09426	3.83326	-1.24004
H	-1.24267	3.19443	-1.45912
C	-2.96232	3.50945	-0.16055
H	-2.77015	2.62470	0.44084
C	-4.03888	4.30831	0.12183
H	-4.69880	4.05647	0.94520
C	-4.32317	5.48472	-0.64573
C	-5.43529	6.30765	-0.35981
C	-5.67275	7.46575	-1.13592
C	-6.77858	8.33855	-0.87231
H	-7.44468	8.10206	-0.05015
C	-7.00557	9.45691	-1.63037
H	-7.84944	10.10374	-1.40928
C	-6.13889	9.77930	-2.71150
H	-6.33033	10.66858	-3.30479
C	-5.06946	8.97419	-3.00284
H	-4.41353	9.22765	-3.82844

C	-4.78852	7.79530	-2.23779	H	-0.47737	-8.19765	-7.62263
C	-6.39692	5.93148	0.73631	C	0.42541	-8.48911	-5.68363
C	-7.53112	5.16523	0.42911	C	1.61832	-8.86035	-5.03750
H	-7.68657	4.84671	-0.59770	H	1.66310	-8.85066	-3.95248
C	-8.49258	4.84953	1.40399	C	2.75186	-9.23006	-5.75600
C	-8.29465	5.31516	2.71273	H	3.66519	-9.48920	-5.22852
H	-9.01417	5.05372	3.48356	C	-3.83988	-15.71265	3.82230
C	-7.15720	6.06404	3.06089	H	-4.57169	-15.58294	4.61255
C	-6.21558	6.36241	2.05925	C	-3.07245	-16.88217	3.74986
H	-5.34893	6.97098	2.30227	H	-3.20639	-17.66402	4.49048
N	6.09834	-10.71355	-9.23727	C	-2.15213	-17.01791	2.71343
N	3.85197	-9.70412	-7.91456	H	-1.54757	-17.91500	2.62047
N	-3.72917	-14.71075	2.94036	C	-2.02558	-15.97396	1.79161
N	-3.44757	-12.66478	1.05583	H	-1.32101	-16.04303	0.96835
N	-15.54410	-1.83767	-0.14153	C	-2.82630	-14.83774	1.93720
N	-13.19207	-1.35174	-1.57084	C	-2.71548	-13.72635	0.98878
N	-6.50451	5.66211	-12.77490	H	-1.97344	-13.84125	0.19493
N	-6.55570	3.65346	-10.82615	C	-3.17085	-11.63017	0.10959
C	7.15245	-11.24503	-9.87032	C	-1.84923	-11.23655	-0.15814
H	7.54695	-10.68176	-10.70981	H	-1.03263	-11.65171	0.42490
C	7.72892	-12.46628	-9.49996	C	-1.58034	-10.29401	-1.14434
H	8.57769	-12.85442	-10.05375	H	-0.55084	-9.99454	-1.31409
C	7.18633	-13.16501	-8.42413	C	-2.60838	-9.71762	-1.91244
H	7.60189	-14.11917	-8.11473	C	-3.93066	-10.08409	-1.59863
C	6.08904	-12.61464	-7.75496	H	-4.75245	-9.67460	-2.17833
H	5.63415	-13.13203	-6.91582	C	-4.21317	-11.01092	-0.59485
C	5.57262	-11.39045	-8.18798	H	-5.23927	-11.30665	-0.40467
C	4.40474	-10.80313	-7.52539	C	-16.65885	-2.12682	0.54281
H	3.99287	-11.36659	-6.68471	H	-16.92365	-1.44904	1.34688
C	2.72975	-9.23856	-7.16025	C	-17.47228	-3.22900	0.25043
C	1.56641	-8.81654	-7.81911	H	-18.36916	-3.40918	0.83437
H	1.52945	-8.83806	-8.90269	C	-17.11402	-4.06817	-0.80156
C	0.43270	-8.45773	-7.08976	H	-17.72734	-4.92478	-1.06430

C	-15.94706	-3.77967	-1.51610	H	-9.02893	0.49812	-8.55374
H	-15.63216	-4.40882	-2.34317	C	-7.93325	1.95838	-9.66770
C	-15.18941	-2.66219	-1.15637	H	-8.78525	2.19733	-10.29749
C	-13.95560	-2.34261	-1.88097	C	-1.13528	-9.01308	-3.80695
H	-13.68946	-3.01196	-2.70314	H	-0.52063	-9.88453	-3.60429
C	-12.02304	-1.18864	-2.39066	C	-2.28455	-8.78940	-3.03203
C	-12.13516	-1.01800	-3.77783	C	-3.10607	-7.69806	-3.36283
H	-13.11649	-0.92584	-4.23477	H	-3.98947	-7.48729	-2.76681
C	-10.99139	-0.98395	-4.57613	C	-2.80036	-6.85072	-4.43959
H	-11.10494	-0.88515	-5.65167	C	-1.64766	-7.10889	-5.19806
C	-9.70956	-1.14068	-4.02271	H	-1.40018	-6.44770	-6.02400
C	-9.61547	-1.27220	-2.62463	C	-0.79973	-8.18687	-4.88982
H	-8.63688	-1.37639	-2.16442	C	-3.69119	-5.67769	-4.75313
C	-10.74997	-1.27963	-1.81238	C	-4.79268	-5.83784	-5.62427
H	-10.65377	-1.41425	-0.73892	C	-5.07399	-7.09021	-6.26181
C	-6.50801	6.64835	-13.68153	H	-4.41730	-7.93151	-6.06930
H	-5.66233	6.68504	-14.35989	C	-6.14468	-7.24131	-7.10297
C	-7.54000	7.58920	-13.78384	H	-6.33634	-8.20001	-7.57586
H	-7.49541	8.36086	-14.54555	C	-7.01237	-6.14439	-7.36333
C	-8.61504	7.50379	-12.90253	H	-7.85724	-6.27701	-8.03267
H	-9.43705	8.21092	-12.95808	C	-6.78508	-4.92839	-6.77462
C	-8.61546	6.48320	-11.94729	H	-7.45194	-4.09855	-6.98013
H	-9.43683	6.38165	-11.24449	C	-5.67794	-4.71938	-5.88879
C	-7.54687	5.58259	-11.91198	C	-5.44011	-3.46786	-5.27477
C	-7.52846	4.49650	-10.92934	C	-4.32672	-3.30313	-4.42095
H	-8.39263	4.43778	-10.26347	C	-4.04220	-2.04989	-3.78664
C	-6.69664	2.59438	-9.87929	H	-4.70296	-1.21136	-3.97931
C	-5.58906	2.18043	-9.12439	C	-2.96445	-1.89418	-2.95532
H	-4.62631	2.65843	-9.27654	H	-2.77218	-0.93078	-2.49025
C	-5.73689	1.21009	-8.13239	C	-2.09533	-2.99037	-2.69710
H	-4.87602	0.94351	-7.52592	H	-1.24280	-2.86001	-2.03565
C	-6.98170	0.60705	-7.87105	C	-2.33000	-4.20896	-3.27760
C	-8.06799	0.98737	-8.68043	H	-1.66435	-5.04090	-3.07494

C	-3.44159	-4.42059	-4.15726	H	1.67544	1.00203	9.63576
C	-6.40258	-2.33103	-5.49600	C	0.43873	-0.67784	10.19074
C	-7.53585	-2.21441	-4.67746	C	0.44712	-1.91099	10.86713
H	-7.68999	-2.94434	-3.88790	H	-0.46285	-2.50256	10.90996
C	-8.49807	-1.21281	-4.89050	C	1.58188	-2.36287	11.54105
C	-8.30191	-0.31233	-5.94853	H	1.54569	-3.29023	12.10204
H	-9.02208	0.48549	-6.10687	C	-3.82958	11.16389	11.70142
C	-7.16540	-0.38477	-6.77247	H	-4.56275	11.78315	11.19559
C	-6.22294	-1.40085	-6.53102	C	-3.06066	11.68587	12.74942
H	-5.35705	-1.49436	-7.18062	H	-3.19474	12.71794	13.05698
N	6.11693	-2.63987	13.88762	C	-2.13871	10.85642	13.38315
N	3.86854	-2.00090	12.35422	H	-1.53292	11.22444	14.20565
N	-3.71877	9.89946	11.27387	C	-2.01211	9.53642	12.93909
N	-3.43661	7.24528	10.44198	H	-1.30628	8.85821	13.40897
N	-15.54218	0.79041	1.68125	C	-2.81434	9.09444	11.88344
N	-13.19002	-0.69025	1.97262	C	-2.70362	7.71770	11.39431
N	-6.49592	-13.89790	1.49234	H	-1.96066	7.08766	11.88937
N	-6.54767	-11.20584	2.25746	C	-3.16007	5.90862	10.01851
C	7.17224	-2.92145	14.66310	C	-1.83855	5.48056	9.80964
H	7.56715	-3.92993	14.59487	H	-1.02221	6.19347	9.87655
C	7.74925	-1.98943	15.53448	C	-1.56948	4.15532	9.48613
H	8.59901	-2.27420	16.14651	H	-0.54008	3.85898	9.31033
C	7.20590	-0.70871	15.60198	C	-2.59726	3.20148	9.37261
H	7.62184	0.03681	16.27285	C	-3.91951	3.65584	9.53494
C	6.10740	-0.40531	14.79204	H	-4.74104	2.94865	9.47138
H	5.65196	0.57984	14.82078	C	-4.20224	4.98846	9.83600
C	5.59054	-1.39301	13.94936	H	-5.22828	5.30043	9.99862
C	4.42160	-1.11383	13.11058	C	-16.65682	1.52795	1.59068
H	4.00915	-0.10430	13.17844	H	-16.92232	1.88588	0.60204
C	2.74520	-1.58114	11.57509	C	-17.46933	1.82554	2.69215
C	2.76616	-0.36961	10.86498	H	-18.36615	2.42164	2.55718
H	3.67935	0.21687	10.82406	C	-17.11028	1.33337	3.94439
C	1.63157	0.06742	10.18707	H	-17.72291	1.53384	4.81815

C	-15.94344	0.56994	4.05056	H	-4.86970	-6.99172	2.95184
H	-15.62792	0.16771	5.00852	C	-5.58186	-8.99494	2.68096
C	-15.18669	0.32316	2.90224	H	-4.61924	-9.36523	2.34218
C	-13.95283	-0.46415	2.98651	C	-1.12301	1.20902	9.70797
H	-13.68595	-0.84197	3.97688	H	-0.50759	1.82057	10.36031
C	-12.02045	-1.48144	2.23978	C	-2.27322	1.76787	9.12826
C	-12.13129	-2.76821	2.78560	C	-3.09560	0.93543	8.34978
H	-13.11223	-3.21047	2.93545	H	-3.97981	1.34595	7.87050
C	-10.98674	-3.47618	3.15366	C	-2.78986	-0.42072	8.15420
H	-11.09926	-4.45712	3.60588	C	-1.63628	-0.94813	8.75561
C	-9.70532	-2.91802	3.01094	H	-1.38875	-1.99397	8.59584
C	-9.61253	-1.64139	2.42581	C	-0.78744	-0.14186	9.53372
H	-8.63430	-1.19036	2.28467	C	-3.68158	-1.27910	7.29637
C	-10.74785	-0.93464	2.02779	C	-4.78054	-1.95590	7.87263
H	-10.65264	0.06244	1.60772	C	-5.05868	-1.88360	9.27670
C	-6.49918	-15.17618	1.09154	H	-4.40155	-1.29564	9.90802
H	-5.65271	-15.78146	1.39782	C	-6.12707	-2.53870	9.83002
C	-7.53185	-15.73583	0.32927	H	-6.31644	-2.47006	10.89720
H	-7.48700	-16.78128	0.04176	C	-6.99547	-3.31321	9.01152
C	-8.60787	-14.93052	-0.03594	H	-7.83856	-3.82819	9.46250
H	-9.43043	-15.33269	-0.61947	C	-6.77119	-3.40971	7.66345
C	-8.60857	-13.59298	0.37032	H	-7.43867	-4.00289	7.04849
H	-9.43069	-12.93406	0.10799	C	-5.66655	-2.74473	7.03759
C	-7.53922	-13.11144	1.13114	C	-5.43207	-2.83670	5.64603
C	-7.52098	-11.71735	1.58022	C	-4.32123	-2.17712	5.07441
H	-8.38585	-11.11185	1.29919	C	-4.03986	-2.25266	3.67119
C	-6.68891	-9.85631	2.70120	H	-4.70104	-2.83942	3.04237
C	-7.92538	-9.35570	3.14734	C	-2.96446	-1.60829	3.11874
H	-8.77693	-10.02103	3.25619	H	-2.77455	-1.68595	2.05136
C	-8.06053	-8.01522	3.49456	C	-2.09463	-0.83598	3.93765
H	-9.02131	-7.66136	3.85583	H	-1.24390	-0.32664	3.49254
C	-6.97483	-7.12350	3.41798	C	-2.32624	-0.73119	5.28391
C	-5.73013	-7.65070	3.02517	H	-1.65991	-0.13945	5.90209

C	-3.43532	-1.38952	5.90901	C	-8.29645	-4.99949	3.25459
C	-6.39549	-3.59725	4.77364	H	-9.01729	-5.53590	2.64396
C	-7.52978	-2.94722	4.26496	C	-7.15894	-5.67627	3.72773
H	-7.68403	-1.89852	4.50244	C	-6.21572	-4.95863	4.48552
C	-8.49284	-3.63302	3.50559	H	-5.34897	-5.47403	4.89001

Table S2. Cartesian coordinates (in Å) for the B3LYP 6-31G(d) model of **2**.

Zn	4.09565	3.41065	7.48702	C	-11.27965	3.64277	-3.97019
N	3.93233	5.57258	8.08008	H	-12.23602	3.19446	-4.22130
C	4.87556	6.35160	8.62506	C	-10.47928	3.09552	-2.96208
H	5.84897	5.89692	8.77893	H	-10.80070	2.21528	-2.41361
C	4.64693	7.68457	8.98917	C	-9.25925	3.70380	-2.66026
H	5.44739	8.26941	9.43046	N	-7.29841	3.72950	-1.24740
C	3.38458	8.23177	8.77538	C	-8.39489	3.15924	-1.60517
H	3.17015	9.26003	9.04994	H	-8.73096	2.23846	-1.12086
C	2.39593	7.42744	8.19922	C	-6.50893	3.05928	-0.25345
H	1.39859	7.81660	8.01810	C	-6.30001	3.63243	1.00778
C	2.70515	6.10702	7.86685	H	-6.77464	4.57655	1.25832
N	1.91278	3.99079	7.00939	C	-5.53340	2.96076	1.96213
C	1.69093	5.23223	7.26439	H	-5.42664	3.39607	2.95166
H	0.71944	5.68372	7.04601	C	-4.93267	1.71941	1.67911
C	0.86325	3.26176	6.35559	C	-5.15295	1.16692	0.40725
C	0.21163	2.20234	7.00049	H	-4.68008	0.22589	0.14266
H	0.46971	1.95182	8.02522	C	-5.93564	1.81575	-0.54416
C	-0.81070	1.51018	6.34828	H	-6.06505	1.38211	-1.53011
H	-1.33975	0.72689	6.88365	C	-4.05816	1.01475	2.65848
C	-1.19140	1.83313	5.03196	C	-4.09193	-0.38464	2.77014
C	-0.52147	2.89589	4.40496	H	-4.80421	-0.94705	2.17382
H	-0.76705	3.15321	3.37900	N	-2.65250	-9.58007	3.60862
C	0.48277	3.60930	5.05405	C	-2.48740	-10.90828	3.65469
H	1.00610	4.40615	4.53630	H	-2.70701	-11.45477	2.74307
C	-2.23471	1.06785	4.29279	C	-2.05773	-11.58386	4.80387
C	-3.12924	1.72535	3.43292	H	-1.94413	-12.66288	4.78603
H	-3.09020	2.80771	3.35407	C	-1.78578	-10.84693	5.95350
N	-8.81760	4.80797	-3.31049	H	-1.45567	-11.33777	6.86388
C	-9.58614	5.32109	-4.27980	C	-1.94779	-9.45829	5.91158
H	-9.20318	6.20178	-4.78544	H	-1.74765	-8.84869	6.78752
C	-10.82321	4.77350	-4.64234	C	-2.38227	-8.86219	4.72606
H	-11.40662	5.23257	-5.43380	N	-3.03380	-6.82660	3.59762

C	-2.56749	-7.40747	4.64670	H	-0.81570	6.31975	-3.01835
H	-2.29725	-6.82841	5.53383	C	-0.98361	5.27925	-1.13054
C	-3.10015	-5.39291	3.62113	C	-1.87889	4.95418	-0.09894
C	-4.33238	-4.72759	3.57833	H	-1.53229	4.36673	0.74598
H	-5.25580	-5.29912	3.57075	C	-3.21750	5.33427	-0.14825
C	-4.37440	-3.33224	3.60790	H	-3.89555	5.04379	0.64723
H	-5.34045	-2.83583	3.62741	C	0.43321	4.82273	-1.06151
C	-3.19572	-2.56365	3.65169	C	1.12647	4.82428	0.15969
C	-1.97290	-3.25263	3.69005	H	0.63719	5.21561	1.04665
H	-1.04240	-2.69289	3.69299	N	6.32251	3.34897	7.78590
C	-1.91928	-4.64398	3.68573	C	6.99612	3.14594	8.92544
H	-0.96066	-5.15183	3.68469	H	6.39996	2.96240	9.81360
C	-3.21311	-1.07396	3.61875	C	8.39467	3.16561	8.99837
C	-2.29613	-0.33085	4.37957	H	8.88888	2.99789	9.94975
H	-1.60814	-0.85236	5.03831	C	9.12413	3.40410	7.83660
Zn	-6.84443	5.57635	-2.55641	H	10.20927	3.43141	7.85676
N	-7.79390	7.05312	-1.15287	C	8.42860	3.60903	6.64053
C	-9.08357	7.40579	-1.07611	H	8.96102	3.79977	5.71366
H	-9.76409	6.90146	-1.75469	C	7.03279	3.57545	6.65398
C	-9.55786	8.37182	-0.17974	N	4.98377	3.83909	5.39960
H	-10.61378	8.62133	-0.16255	C	6.26937	3.79085	5.41799
C	-8.65321	8.99608	0.67500	H	6.84770	3.92011	4.49921
H	-8.98458	9.75305	1.37918	C	4.35498	3.98445	4.11741
C	-7.30560	8.62772	0.60705	C	3.62074	5.13491	3.80091
H	-6.56901	9.09248	1.25542	H	3.55862	5.95064	4.51515
C	-6.91425	7.65671	-0.31683	C	3.02174	5.25811	2.54564
N	-5.09358	6.41066	-1.30380	H	2.50143	6.17944	2.29973
C	-5.50729	7.24817	-0.41902	C	3.11263	4.23233	1.58571
H	-4.81147	7.69912	0.29360	C	3.85024	3.08749	1.92725
C	-3.70770	6.04015	-1.25322	H	3.91591	2.26291	1.22377
C	-2.83410	6.37266	-2.29682	C	4.47235	2.96366	3.16676
H	-3.19433	6.95651	-3.13870	H	5.01042	2.05564	3.41755
C	-1.48842	6.00715	-2.22475	C	2.42864	4.31339	0.26429

C	3.04447	3.80782	-0.89200
H	4.05129	3.40663	-0.82422
N	5.63019	-0.15587	-8.95012
C	6.05891	-0.76851	-10.06116
H	6.70837	-1.62659	-9.92038
C	5.70651	-0.34610	-11.34919
H	6.08587	-0.87828	-12.21543
C	4.87073	0.75883	-11.48807
H	4.58094	1.11717	-12.47107
C	4.41183	1.39961	-10.33258
H	3.76009	2.26552	-10.39883
C	4.81209	0.91627	-9.08527
N	4.75521	1.19802	-6.68548
C	4.35353	1.56664	-7.85091
H	3.65189	2.39740	-7.96439
C	4.17800	1.86806	-5.55482
C	4.96337	2.66317	-4.70989
H	6.01558	2.81393	-4.93249
C	4.38041	3.31051	-3.61871
H	4.99441	3.96479	-3.00636
C	3.01203	3.16205	-3.32303
C	2.24628	2.35713	-4.18160
H	1.19426	2.19463	-3.96718
C	2.81137	1.72638	-5.28688
H	2.20434	1.08501	-5.91702
C	2.38157	3.79066	-2.12808
C	1.07870	4.31005	-2.19648
H	0.55314	4.30007	-3.14671
Zn	-3.38269	-8.33548	1.88523
N	-3.28477	-10.04221	0.42599
C	-4.20370	-10.99080	0.20398
H	-5.12230	-10.91798	0.77740
C	-4.01658	-12.03441	-0.71118
H	-4.79416	-12.77865	-0.84895
C	-2.82427	-12.09301	-1.42785
H	-2.64289	-12.89054	-2.14180
C	-1.86248	-11.10156	-1.20799
H	-0.91930	-11.11318	-1.74578
C	-2.12653	-10.09608	-0.27587
N	-1.31147	-8.13877	0.88439
C	-1.13823	-9.04123	-0.01616
H	-0.22913	-9.05911	-0.62325
C	-0.30350	-7.12234	0.99108
C	0.48450	-7.00665	2.14370
H	0.36553	-7.71953	2.95428
C	1.46351	-6.01429	2.22401
H	2.09985	-5.97374	3.10350
C	1.66495	-5.09610	1.17602
C	0.86043	-5.22929	0.03304
H	0.96622	-4.51989	-0.78225
C	-0.10231	-6.23006	-0.06857
H	-0.73185	-6.29231	-0.94987
C	2.65895	-3.98950	1.26507
C	3.40265	-3.60357	0.13817
H	3.28750	-4.15798	-0.78845
N	8.04507	0.52693	-6.84214
C	8.65920	1.07809	-7.89693
H	8.16405	0.96845	-8.85648
C	9.87653	1.76362	-7.79806
H	10.33173	2.18813	-8.68694
C	10.47993	1.88338	-6.54897
H	11.42607	2.40364	-6.43573
C	9.84103	1.31779	-5.44069
H	10.28015	1.38824	-4.45015
C	8.62953	0.64865	-5.62534
N	6.85245	-0.64656	-4.62237

C	7.93250	0.03911	-4.48539	C	3.76067	-1.33550	3.76792
H	8.38101	0.18619	-3.49921	C	2.59635	-1.05113	4.49925
C	6.23029	-1.12958	-3.42229	H	1.63986	-1.43073	4.15264
C	6.13870	-2.50282	-3.16013	C	2.62926	-0.25855	5.64357
H	6.58093	-3.21599	-3.84950	H	1.71074	-0.02858	6.17303
C	5.53432	-2.95077	-1.98380	C	3.68143	-2.15591	2.52649
H	5.51995	-4.01634	-1.77308	C	2.81689	-3.25998	2.45268
C	4.98162	-2.04972	-1.05399	H	2.24569	-3.54580	3.33091
C	5.08198	-0.67884	-1.34037	N	-5.55054	-8.68441	2.36620
H	4.63979	0.04407	-0.66140	C	-6.07081	-9.54566	3.25001
C	5.70398	-0.21929	-2.49825	H	-5.36361	-10.13277	3.82715
H	5.74103	0.84384	-2.71103	C	-7.44944	-9.70384	3.43975
C	4.27527	-2.50597	0.17625	H	-7.81635	-10.41964	4.16813
C	4.40878	-1.79817	1.38168	C	-8.32452	-8.93199	2.68005
H	5.07867	-0.94453	1.42389	H	-9.39926	-9.03170	2.79702
N	3.65310	2.66112	9.55949	C	-7.78935	-8.02419	1.76037
C	3.57771	3.36918	10.69374	H	-8.43843	-7.40567	1.14793
H	3.72144	4.44128	10.60507	C	-6.40237	-7.92914	1.63065
C	3.33020	2.78494	11.94235	N	-4.53648	-6.92279	0.46992
H	3.28274	3.40675	12.83039	C	-5.80537	-6.99042	0.67190
C	3.15099	1.40605	12.01457	H	-6.49525	-6.34652	0.11982
H	2.96234	0.91860	12.96615	C	-4.07422	-5.91490	-0.44157
C	3.22004	0.65998	10.83349	C	-3.45459	-6.26416	-1.64859
H	3.08761	-0.41761	10.84825	H	-3.35796	-7.31012	-1.92436
C	3.47301	1.31955	9.62910	C	-3.01790	-5.26762	-2.52369
N	3.85891	1.12616	7.25103	H	-2.58796	-5.55781	-3.47815
C	3.55688	0.56790	8.37021	C	-3.15952	-3.90249	-2.21016
H	3.35817	-0.50583	8.42461	C	-3.77983	-3.57714	-0.99329
C	3.83839	0.29470	6.08113	H	-3.87837	-2.53520	-0.70387
C	5.01302	0.02798	5.36558	C	-4.24121	-4.56191	-0.12375
H	5.96283	0.41726	5.72017	H	-4.68933	-4.28448	0.82444
C	4.97132	-0.78817	4.23342	C	-2.64406	-2.82252	-3.09811
H	5.90006	-1.02646	3.72283	C	-3.36061	-1.62559	-3.25872

H	-4.32535	-1.51584	-2.77243
N	-6.63218	7.07543	-4.21789
C	-7.15065	8.30827	-4.28726
H	-7.72365	8.64102	-3.42767
C	-6.98116	9.14541	-5.39728
H	-7.42667	10.13486	-5.40131
C	-6.23893	8.68204	-6.48036
H	-6.09124	9.30171	-7.35949
C	-5.68706	7.39860	-6.41276
H	-5.10358	7.00035	-7.23732
C	-5.90496	6.62659	-5.26991
N	-5.58111	4.50273	-4.16279
C	-5.34471	5.27299	-5.16590
H	-4.71506	4.93658	-5.99397
C	-4.91683	3.23033	-4.14683
C	-5.64441	2.03620	-4.23319
H	-6.72284	2.06754	-4.35740
C	-4.97761	0.80945	-4.22170
H	-5.55411	-0.10325	-4.34288
C	-3.57734	0.73685	-4.09555
C	-2.87026	1.94660	-4.00657
H	-1.79225	1.92889	-3.87723
C	-3.52203	3.17652	-4.04161
H	-2.95535	4.09601	-3.94014
C	-2.84979	-0.56117	-4.01614
C	-1.59913	-0.71916	-4.63485
H	-1.19085	0.09823	-5.22173
N	7.14594	-2.58345	-7.35351
C	8.41073	-2.76710	-7.75365
H	9.03625	-1.88147	-7.80173
C	8.92627	-4.02245	-8.10001
H	9.95886	-4.11304	-8.42103
C	8.09206	-5.13471	-8.02486
H	8.45634	-6.12235	-8.29046
C	6.77190	-4.95295	-7.60003
H	6.08972	-5.79477	-7.52986
C	6.33559	-3.66706	-7.27493
N	4.49259	-2.26152	-6.58974
C	4.95500	-3.43801	-6.82953
H	4.32175	-4.32211	-6.71686
C	3.14846	-2.17808	-6.09278
C	2.13767	-1.56768	-6.84682
H	2.35773	-1.18864	-7.84045
C	0.83561	-1.50256	-6.34680
H	0.05489	-1.07307	-6.96814
C	0.51122	-2.01533	-5.07648
C	1.54158	-2.61870	-4.33777
H	1.33538	-2.99752	-3.34122
C	2.83845	-2.71119	-4.83613
H	3.62326	-3.15465	-4.23257
C	-0.85618	-1.90077	-4.49544
C	-1.39785	-2.94601	-3.73001
H	-0.83165	-3.86508	-3.61175
Zn	6.13117	-0.65118	-6.81565

Table S3. Cartesian coordinates (in Å) for the B3LYP 6-31G(d) model of trigonal prism cage **3** (all Δ handedness).

C	-6.52123	0.02725	7.43856	C	-12.88602	-10.61887	0.07029
C	-6.91306	-0.06989	6.09637	C	-11.81526	-10.00475	0.72658
C	-6.38117	-1.05800	5.27208	C	-1.42581	-4.29598	3.79301
C	-5.44659	-1.99003	5.75474	C	-0.75177	-5.43614	4.29005
C	-5.08966	-1.91106	7.11350	C	0.69218	-5.53540	4.17442
C	-5.62186	-0.92986	7.94631	C	1.42928	-4.48571	3.58196
C	-4.86667	-3.02665	4.85422	C	0.75877	-3.33466	3.10646
C	-5.69864	-3.78681	4.02219	C	-0.68498	-3.24273	3.20228
C	-5.18347	-4.77013	3.15989	C	1.46964	-2.23691	2.51802
C	-3.78631	-4.93817	3.10690	C	0.82124	-1.12108	2.05783
C	-2.92154	-4.17109	3.90539	C	-0.59676	-1.03951	2.12965
C	-3.48030	-3.24023	4.79603	C	-1.31891	-2.06947	2.67365
C	-6.11561	-5.60160	2.34484	C	-1.45070	-6.50590	4.93846
C	-5.76571	-6.13246	1.08867	C	-0.79078	-7.60411	5.42378
C	-6.66540	-6.88645	0.33421	C	0.62224	-7.70729	5.29698
C	-7.95919	-7.15008	0.81083	C	1.33572	-6.70480	4.69507
C	-8.32630	-6.60839	2.05714	C	2.92390	-4.58607	3.42155
C	-7.41941	-5.86941	2.80620	C	3.46536	-5.26999	2.32198
N	-7.03201	1.10330	8.22517	C	4.85098	-5.29859	2.09255
N	-8.87978	-7.90815	0.02414	C	5.69499	-4.63856	2.99546
C	-6.28048	1.59498	9.15241	C	5.19421	-3.98374	4.13383
C	-9.76143	-8.61826	0.64692	C	3.79953	-3.96511	4.32651
C	-6.70653	2.67819	10.04129	C	6.13383	-3.34022	5.09604
C	-10.86212	-9.31082	-0.02512	C	5.42132	-5.98902	0.90060
C	-5.83255	3.16971	11.01593	C	7.44197	-3.83564	5.26123
C	-6.25835	4.20149	11.85588	C	8.35223	-3.23202	6.12006
C	-7.54331	4.71104	11.68569	C	7.98396	-2.11058	6.88701
C	-8.35602	4.16494	10.68582	C	6.68572	-1.60135	6.72820
N	-7.95833	3.16998	9.88159	C	5.78275	-2.20755	5.85486
N	-10.92977	-9.22808	-1.37677	C	6.36496	-5.35051	0.07773
C	-11.96098	-9.81630	-1.99795	C	6.89677	-5.98247	-1.04342
C	-12.96387	-10.51602	-1.31656	C	6.49623	-7.28042	-1.38932

C	5.58466	-7.94719	-0.54826	H	-11.98573	-9.73198	-3.07911
C	5.05255	-7.30551	0.56743	H	-13.77862	-10.97042	-1.87081
N	7.01486	1.68969	8.10176	H	-13.64003	-11.16248	0.63142
N	8.90320	-5.99600	-5.15877	H	-11.72268	-10.05914	1.80705
C	6.26302	1.52855	9.13855	H	2.54996	-2.29827	2.44857
C	9.79544	-6.13013	-6.08349	H	1.38370	-0.29132	1.63824
C	6.69209	0.80628	10.33840	H	-1.10140	-0.15333	1.75409
C	10.88908	-7.10053	-6.01394	H	-2.39972	-1.99993	2.71572
N	7.94841	0.30048	10.35638	H	-2.52603	-6.43135	5.05569
C	8.34765	-0.36789	11.44680	H	-1.34339	-8.39740	5.91826
C	7.53196	-0.55893	12.56769	H	1.13344	-8.57998	5.69228
C	6.24229	-0.03329	12.55377	H	2.41484	-6.78218	4.61876
C	5.81515	0.65787	11.41747	H	2.79396	-5.75146	1.61659
C	11.85414	-7.14182	-7.02483	H	6.76375	-4.64537	2.80631
C	12.91570	-8.04512	-6.91746	H	3.38119	-3.47813	5.20240
C	12.97245	-8.87554	-5.80071	H	7.75637	-4.72120	4.71898
C	11.95883	-8.78305	-4.83959	H	9.36147	-3.62804	6.17417
N	10.93635	-7.92260	-4.93663	H	6.38073	-0.73238	7.29951
H	-7.60299	0.65582	5.68516	H	4.79342	-1.77344	5.74733
H	-6.67576	-1.08786	4.22705	H	6.66873	-4.33218	0.30250
H	-4.41215	-2.64893	7.53299	H	7.59530	-5.45045	-1.67646
H	-5.36322	-0.93820	9.00101	H	5.31696	-8.98269	-0.73650
H	-6.76884	-3.61004	4.05763	H	4.36669	-7.84835	1.21113
H	-3.35737	-5.69030	2.45135	H	9.35957	-0.75792	11.43164
H	-2.81940	-2.64569	5.42020	H	7.91171	-1.10167	13.42711
H	-4.77994	-5.93707	0.67753	H	5.58394	-0.15493	13.40833
H	-6.36076	-7.28281	-0.62755	H	4.81779	1.08391	11.36846
H	-9.33261	-6.74371	2.44089	H	11.77752	-6.47548	-7.87868
H	-7.73305	-5.50602	3.77907	H	13.67862	-8.09587	-7.68836
H	-4.83895	2.74428	11.11799	H	13.77908	-9.58938	-5.66934
H	-5.60271	4.59475	12.62657	H	11.96752	-9.42690	-3.96664
H	-7.92190	5.51052	12.31411	C	-7.95807	4.27758	5.78590
H	-9.36432	4.53511	10.53540	C	-6.66414	3.73336	5.79667

C	-5.76429	4.00927	4.76647
C	-6.11416	4.83088	3.67797
C	-7.41810	5.36399	3.67855
C	-8.32515	5.08522	4.69302
C	-5.18180	5.12068	2.55048
C	-5.69664	5.37562	1.26757
C	-4.86442	5.71589	0.19335
C	-3.47810	5.77210	0.40765
C	-2.91967	5.46654	1.65933
C	-3.78467	5.15886	2.72277
C	-5.44409	5.97782	-1.15466
C	-6.38022	5.09519	-1.72011
C	-6.91204	5.31562	-2.98789
C	-6.51854	6.42858	-3.74350
C	-5.61754	7.34552	-3.16888
C	-5.08550	7.11432	-1.90278
N	-8.87883	3.97621	6.83581
N	-7.02965	6.57229	-5.06848
C	-9.75997	4.87127	7.13889
C	-6.27810	7.12879	-5.95832
C	-10.86089	4.63663	8.07470
C	-6.70465	7.35734	-7.34063
C	-11.81348	5.63523	8.29919
C	-12.88459	5.37490	9.15904
C	-12.96334	4.12269	9.76382
C	-11.96092	3.18205	9.49918
N	-10.92929	3.42501	8.67943
N	-7.95698	6.97425	-7.68606
C	-8.35522	7.17388	-8.94956
C	-7.54258	7.76628	-9.92277
C	-6.25707	8.16725	-9.56723
C	-5.83069	7.95508	-8.25401
C	-1.42399	5.43181	1.82399
C	-0.68302	4.39332	1.20788
C	0.76069	4.35644	1.33567
C	1.43102	5.34425	2.09432
C	0.69378	6.38251	2.70643
C	-0.75017	6.43276	2.56248
C	1.33715	7.41863	3.45825
C	0.62350	8.44140	4.02481
C	-0.78952	8.49930	3.87201
C	-1.44929	7.52940	3.16413
C	-1.31683	3.34848	0.45660
C	-0.59456	2.36200	-0.16263
C	0.82342	2.34067	-0.05580
C	1.47169	3.29760	0.67996
C	2.92563	5.25567	2.26157
C	3.80124	5.72833	1.27107
C	5.19591	5.57076	1.38359
C	5.69666	4.91277	2.52012
C	4.85267	4.46131	3.54339
C	3.46706	4.64585	3.40393
C	5.42317	3.77444	4.73730
C	6.13557	6.08185	0.34504
C	6.36730	2.74302	4.59556
C	6.89954	2.08822	5.70329
C	6.49888	2.43720	7.00039
C	5.58679	3.49850	7.15751
C	5.05432	4.14379	6.04407
C	7.44365	6.47277	0.69149
C	8.35398	6.91448	-0.26072
C	7.98577	7.01745	-1.61545
C	6.68763	6.62501	-1.97706
C	5.78459	6.17204	-1.01536
N	8.90494	-1.47068	7.77154
N	8.90653	7.46355	-2.61206

C	9.79730	-2.20493	8.34925	H	2.41627	7.39145	3.56347
C	9.79877	8.33123	-2.26529	H	1.13457	9.22051	4.58247
C	10.89182	-1.66001	9.15405	H	-1.34227	9.32431	4.31135
C	10.89268	8.75644	-3.14003	H	-2.52464	7.59338	3.04096
N	10.94015	-0.31595	9.32664	H	-2.39763	3.35012	0.37519
C	11.96360	0.19790	10.02218	H	-1.09912	1.59330	-0.74188
C	12.97709	-0.58868	10.58246	H	1.38598	1.56212	-0.56403
C	12.91910	-1.97107	10.42255	H	2.55199	3.26821	0.76806
C	11.85661	-2.51527	9.69498	H	3.38292	6.24296	0.41112
C	11.85753	9.65251	-2.66987	H	6.76542	4.75237	2.62062
C	12.91941	10.01128	-3.50539	H	2.79563	4.27608	4.17376
C	12.97675	9.45941	-4.78292	H	6.67119	2.42881	3.60123
C	11.96327	8.58088	-5.18389	H	7.59872	1.27456	5.55891
N	10.94043	8.23469	-4.39067	H	5.31899	3.85303	8.14843
H	-6.35961	3.09952	6.62153	H	4.36815	4.97239	6.19250
H	-4.77837	3.55590	4.80347	H	7.75793	6.44627	1.72958
H	-7.73170	6.02425	2.87693	H	9.36314	7.15955	0.05524
H	-9.33153	5.48500	4.61802	H	6.38302	6.68488	-3.01538
H	-6.76681	5.31807	1.09651	H	4.79534	5.86154	-1.33750
H	-2.81695	6.01518	-0.41918	H	11.97323	1.27587	10.14269
H	-3.35588	4.96745	3.70202	H	13.78453	-0.11829	11.13404
H	-6.67626	4.20573	-1.17148	H	13.68181	-2.61368	10.85178
H	-7.60345	4.59783	-3.41034	H	11.77906	-3.58792	9.54544
H	-5.35750	8.26257	-3.68921	H	11.78053	10.05868	-1.66586
H	-4.40680	7.84559	-1.47378	H	13.68217	10.70428	-3.16353
H	-11.72023	6.59788	7.80567	H	13.78371	9.70257	-5.46643
H	-13.63821	6.13317	9.34877	H	11.97236	8.14681	-6.17795
H	-13.77844	3.87061	10.43431	C	7.98292	-4.90947	-5.27092
H	-11.98641	2.20377	9.96709	C	6.68512	-5.02537	-4.74916
H	-9.36391	6.85936	-9.19445	C	5.78266	-3.96552	-4.83750
H	-7.92162	7.91129	-10.92910	C	6.13387	-2.74282	-5.44049
H	-5.60144	8.63763	-10.29344	C	7.44154	-2.63931	-5.95353
H	-4.83666	8.25528	-7.93710	C	8.35132	-3.68531	-5.86018

C	5.19485	-1.58734	-5.51675
C	5.69629	-0.27435	-5.51516
C	4.85273	0.83798	-5.63523
C	3.46699	0.62560	-5.72485
C	2.92495	-0.66837	-5.68193
C	3.80010	-1.76292	-5.59654
C	5.42367	2.21519	-5.63718
C	6.36796	2.60788	-4.67311
C	6.90025	3.89459	-4.65964
C	6.49953	4.84360	-5.61021
C	5.58744	4.44926	-6.60795
C	5.05487	3.16241	-6.61026
N	7.01165	-7.86073	-2.58753
N	7.01539	6.17120	-5.51329
C	6.25915	-8.67769	-3.24518
C	6.26324	7.14968	-5.89148
C	6.68741	-9.35601	-4.47071
C	6.69218	8.54994	-5.86583
C	5.80971	-10.21595	-5.13822
C	6.23592	-10.85471	-6.30512
C	7.52541	-10.60445	-6.76807
C	8.34190	-9.72957	-6.04268
N	7.94362	-9.11928	-4.91839
N	7.94868	8.81847	-5.43731
C	8.34772	10.09705	-5.40364
C	7.53171	11.16324	-5.79814
C	6.24189	10.88828	-6.24588
C	5.81491	9.55859	-6.27627
C	1.43031	-0.85711	-5.67481
C	0.69283	-0.84470	-6.87987
C	-0.75107	-0.99481	-6.85136
C	-1.42468	-1.13546	-5.61530
C	-0.68347	-1.15105	-4.40802
C	0.76021	-1.02167	-4.44010
C	-1.31696	-1.28068	-3.12751
C	-0.59443	-1.32474	-1.96376
C	0.82351	-1.22120	-1.99887
C	1.47149	-1.06121	-3.19545
C	1.33598	-0.70998	-8.15303
C	0.62217	-0.72948	-9.32197
C	-0.79079	-0.89126	-9.29569
C	-1.45034	-1.02094	-8.10184
C	-2.92037	-1.29535	-5.56307
C	-3.78519	-0.22043	-5.82852
C	-5.18237	-0.35037	-5.70970
C	-5.69751	-1.58879	-5.28920
C	-4.86549	-2.68930	-5.04641
C	-3.47911	-2.53203	-5.20198
C	-5.44532	-3.98757	-4.59918
C	-6.11450	0.77108	-6.02268
C	-6.38139	-4.03581	-3.55203
C	-6.91324	-5.24389	-3.10888
C	-6.51985	-6.45482	-3.69485
C	-5.61881	-6.41582	-4.77622
C	-5.08676	-5.20381	-5.20917
C	-7.41829	0.50530	-6.48521
C	-8.32516	1.52342	-6.75104
C	-7.95807	2.87365	-6.59746
C	-6.66427	3.15475	-6.13094
C	-5.76461	2.12446	-5.85482
N	-7.03098	-7.67409	-3.15676
N	-8.87870	3.93383	-6.86106
C	-6.27926	-8.72284	-3.19351
C	-9.76028	3.74919	-7.78741
C	-6.70577	-10.03434	-2.70056
C	-10.86097	4.67739	-8.05141

N	-7.95827	-10.14227	-2.19659
C	-8.35650	-11.33650	-1.73820
C	-7.54364	-12.47538	-1.76456
C	-6.25792	-12.36761	-2.28899
C	-5.83158	-11.12406	-2.76142
C	-11.81423	4.37305	-9.02799
C	-12.88505	5.24840	-9.23176
C	-12.96281	6.39823	-8.44957
C	-11.95975	6.63878	-7.50315
N	-10.92845	5.80683	-7.30429
H	6.38017	-5.95407	-4.28116
H	4.79368	-4.08842	-4.40666
H	7.75590	-1.72748	-6.45039
H	9.36023	-3.53498	-6.23139
H	6.76518	-0.10769	-5.42699
H	2.79595	1.47751	-5.78905
H	3.38136	-2.76480	-5.61233
H	6.67179	1.90369	-3.90396
H	7.59934	4.17633	-3.88268
H	5.31968	5.13038	-7.41028
H	4.36861	2.87686	-7.40206
H	4.81246	-10.38600	-4.74424
H	5.57700	-11.53373	-6.83733
H	7.90441	-11.07749	-7.66806
H	9.35364	-9.52177	-6.37367
H	9.35974	10.27912	-5.05866
H	7.91134	12.17894	-5.75781
H	5.58331	11.68914	-6.56742
H	4.81742	9.30304	-6.62028
H	-2.39773	-1.35216	-3.08803
H	-1.09875	-1.44304	-1.00842
H	1.38625	-1.27273	-1.07065
H	2.55176	-0.96994	-3.21420

H	2.41507	-0.60499	-8.18213
H	1.13308	-0.63482	-10.27549
H	-1.34367	-0.92249	-10.22980
H	-2.52562	-1.16008	-8.09571
H	-3.35625	0.72327	-6.15238
H	-6.76776	-1.70804	-5.15442
H	-2.81819	-3.36978	-4.99899
H	-6.67738	-3.11591	-3.05610
H	-7.60451	-5.25072	-2.27591
H	-5.35882	-7.32503	-5.31016
H	-4.40811	-5.19804	-6.05702
H	-7.73199	-0.51897	-6.65655
H	-9.33147	1.25867	-7.06004
H	-6.35956	4.18586	-5.99376
H	-4.77884	2.38298	-5.48015
H	-9.36536	-11.39169	-1.34387
H	-7.92269	-13.41955	-1.38738
H	-5.60211	-13.23158	-2.33318
H	-4.83741	-10.99944	-3.17944
H	-11.72172	3.46436	-9.61507
H	-13.63918	5.03400	-9.98292
H	-13.77762	7.10534	-8.56601
H	-11.98443	7.53312	-6.88982
Zn	9.17896	-7.79557	-3.59227
Zn	9.18232	0.78567	8.54611
Zn	9.18298	7.00739	-4.95399
Zn	-9.18310	6.15749	-5.98827
Zn	-9.18484	2.10852	8.32554
Zn	-9.18448	-8.26363	-2.33848
C	11.09435	1.32920	0.41342
C	11.09009	0.30862	1.37680
C	11.09371	-1.02551	0.94448
C	11.08981	-1.34955	-0.42107

C	11.09391	-0.30808	-1.36030	C	10.97084	-10.18113	-0.34750
C	11.09044	1.03654	-0.95815	C	10.77532	-11.53601	-0.62649
C	11.00484	2.11666	-1.97604	C	10.08532	-11.88380	-1.78568
C	11.00400	0.65014	2.82111	C	9.61711	-10.86459	-2.62256
C	11.00349	-2.77111	-0.84742	N	9.80742	-9.56330	-2.36719
C	11.76015	-3.77563	-0.21483	C	10.48152	3.53677	8.60519
C	11.60151	-5.11718	-0.54120	C	10.97318	4.78934	8.98681
C	10.65430	-5.51162	-1.50733	C	10.78021	5.22489	10.30014
C	9.96379	-4.50794	-2.20138	C	10.09303	4.39416	11.18245
C	10.12909	-3.16521	-1.87283	C	9.62481	3.15944	10.71914
N	10.34403	-6.86981	-1.80834	N	9.81262	2.73027	9.46405
C	11.76093	2.07030	-3.16260	H	11.08607	2.36592	0.73678
C	11.60293	3.02392	-4.16114	H	11.08494	-1.82389	1.68064
C	10.65700	4.05893	-4.01918	H	11.08526	-0.54642	-2.41980
C	9.96712	4.15887	-2.80264	H	12.50451	-3.50017	0.52627
C	10.13168	3.20270	-1.80421	H	12.24332	-5.84188	-0.05179
N	10.34723	4.99906	-5.04471	H	9.26787	-4.78198	-2.98439
C	11.76093	1.70005	3.37470	H	9.54020	-2.41871	-2.39747
C	11.60251	2.08813	4.69974	H	12.50438	1.28994	-3.29493
C	10.65521	1.44889	5.52447	H	12.24428	2.96177	-5.03374
C	9.96435	0.34617	5.00232	H	9.27239	4.97493	-2.64812
C	10.12949	-0.04067	3.67517	H	9.54325	3.28444	-0.89517
N	10.34548	1.86748	6.85126	H	12.50536	2.20397	2.76554
C	10.71009	4.75592	-6.26088	H	12.24470	2.87396	5.08274
C	10.70769	-7.80150	-0.99006	H	9.26821	-0.19470	5.63110
C	10.48179	5.68432	-7.36706	H	9.54033	-0.86809	3.29102
C	10.97318	5.38945	-8.64294	H	11.21982	3.83036	-6.52910
C	10.77856	6.30904	-9.67650	H	11.21845	-7.57106	-0.05495
C	10.09001	7.48755	-9.39761	H	11.51128	4.46303	-8.81890
C	9.62223	7.70284	-8.09637	H	11.16162	6.11043	-10.67279
N	9.81163	6.83050	-7.09741	H	9.91917	8.23551	-10.16490
C	10.70850	3.04251	7.24806	H	9.08559	8.61237	-7.84828
C	10.47884	-9.22367	-1.24054	H	11.21764	3.73734	6.57991

H	11.51000	-9.87027	0.54213	C	-10.12561	1.26483	3.45641
H	11.15877	-12.29949	0.04348	N	-10.35545	0.52714	7.08687
H	9.91367	-12.92227	-2.04925	C	-10.72870	-6.57955	-4.31215
H	9.07940	-11.10454	-3.53365	C	-10.72468	7.02456	-3.54122
H	11.51022	5.40488	8.27165	C	-10.50743	-7.82546	-5.04445
H	11.16351	6.18716	10.62557	C	-11.00887	-7.97438	-6.34167
H	9.92355	4.68485	12.21402	C	-10.82067	-9.18713	-7.00914
H	9.08915	2.48982	11.38359	C	-10.12845	-10.20500	-6.35683
C	-11.08147	-1.11627	0.83392	C	-9.65073	-9.97266	-5.06216
C	-11.07748	0.16698	1.40234	N	-9.83371	-8.81595	-4.41146
C	-11.08086	1.28083	0.55015	C	-10.72715	-0.44385	7.85432
C	-11.07718	1.13146	-0.84539	C	-10.50421	8.28174	-4.25426
C	-11.08120	-0.16348	-1.38393	C	-11.00474	9.47969	-3.73392
C	-11.07782	-1.29736	-0.55680	C	-10.81809	10.66396	-4.45114
C	-10.99591	-2.65515	-1.15671	C	-10.12835	10.60774	-5.66021
C	-10.99529	0.32638	2.87814	C	-9.65131	9.37031	-6.10692
C	-10.99457	2.32986	-1.72129	N	-9.83270	8.22865	-5.42977
C	-11.75219	3.48653	-1.45686	C	-10.50682	-0.45446	9.29960
C	-11.59919	4.64260	-2.21271	C	-11.00758	-1.50373	10.07719
C	-10.65692	4.69420	-3.25950	C	-10.82101	-1.47431	11.46141
C	-9.96508	3.51787	-3.58141	C	-10.13105	-0.39910	12.01694
C	-10.12464	2.36128	-2.82294	C	-9.65371	0.60609	11.16834
N	-10.35322	5.87453	-3.99882	N	-9.83511	0.59017	9.84106
C	-11.75349	-3.00399	-2.29082	H	-11.07381	-1.98606	1.48417
C	-11.60099	-4.23658	-2.91425	H	-11.07284	2.27886	0.97827
C	-10.65934	-5.16950	-2.43545	H	-11.07348	-0.29170	-2.46231
C	-9.96753	-4.86054	-1.25564	H	-12.49326	3.47268	-0.66326
C	-10.12656	-3.62541	-0.63306	H	-12.24185	5.48748	-1.98975
N	-10.35582	-6.39999	-3.08795	H	-9.27313	3.51629	-4.41432
C	-11.75319	-0.48091	3.74745	H	-9.53497	1.48378	-3.07110
C	-11.60074	-0.40420	5.12663	H	-12.49400	-2.30929	-2.67580
C	-10.65874	0.47667	5.69496	H	-12.24338	-4.46527	-3.75780
C	-9.96658	1.34348	4.83733	H	-9.27589	-5.58144	-0.83795

H	-9.53687	-3.40198	0.25104	H	-11.54653	-2.32086	9.60753
H	-12.49404	-1.16137	3.33846	H	-11.21120	-2.26968	12.08904
H	-12.24358	-1.01974	5.74663	H	-9.96617	-0.32757	13.08701
H	-9.27466	2.06547	5.25268	H	-9.11565	1.45703	11.57226
H	-9.53566	1.91845	2.82077	H	-5.26219	1.22977	9.30350
H	-11.24272	-5.79795	-4.87180	H	-9.73382	-8.72387	1.73337
H	-11.23707	7.11835	-2.58362	H	9.78095	-5.50055	-6.97550
H	-11.54946	-7.15969	-6.81367	H	-9.73172	5.86472	6.68660
H	-11.21130	-9.33308	-8.01159	H	-5.25943	7.44145	-5.71813
H	-9.96213	-11.16687	-6.83062	H	9.78407	8.78854	-1.27387
H	-9.11100	-10.74736	-4.52806	H	5.23718	-8.88640	-2.92076
H	-11.23950	-1.32016	7.45695	H	5.24113	6.97363	-6.23432
H	-11.54360	9.48127	-2.79138	H	-5.26043	-8.67097	-3.58395
H	-11.20809	11.60512	-4.07579	H	-9.73267	2.86084	-8.42174
H	-9.96347	11.49885	-6.25696	H	9.78229	-3.29220	8.24975
H	-9.11360	9.29476	-7.04602	H	5.24104	1.91379	9.15779

Table S4. Cartesian coordinates (in Å) for the B3LYP 6-31G(d) model of trigonal prism cage **4** (all Δ handedness).

C	6.59562	-3.52794	-6.19859	C	12.74982	-8.68826	6.08591
C	7.13132	-4.53946	-5.39028	C	12.48391	-9.37418	4.90328
C	6.55602	-4.82670	-4.15202	C	11.37860	-8.98652	4.13857
C	5.44719	-4.10446	-3.67251	C	1.25166	-4.68494	-1.00225
C	4.93920	-3.07746	-4.48675	C	0.65794	-5.94854	-1.61754
C	5.49722	-2.79174	-5.72974	C	-0.73718	-6.01847	-1.47581
C	4.81191	-4.40694	-2.35858	C	-1.30871	-4.79803	-0.76619
C	5.57318	-4.68537	-1.21371	C	-0.84050	-3.52176	-1.46584
C	4.96382	-4.98171	0.01561	C	0.56014	-3.44954	-1.57926
C	3.55880	-4.97205	0.10358	C	-1.62891	-2.43905	-1.85259
C	2.77676	-4.66828	-1.01946	C	-1.02628	-1.29317	-2.38281
C	3.41404	-4.41336	-2.24191	C	0.36121	-1.21195	-2.48152
C	5.84067	-5.31871	1.18133	C	1.15562	-2.28261	-2.05727
C	6.85426	-4.43458	1.59341	C	1.35979	-7.01428	-2.17632
C	7.76034	-4.77626	2.59642	C	0.66420	-8.14772	-2.61055
C	7.70423	-6.04660	3.18513	C	-0.71913	-8.22423	-2.45528
C	6.67323	-6.92507	2.82023	C	-1.42295	-7.16368	-1.87461
C	5.74499	-6.55446	1.84249	C	-2.80862	-4.85625	-0.49873
N	7.14727	-3.26195	-7.49209	C	-3.34545	-4.87410	0.79312
N	8.74059	-6.43530	4.10102	C	-4.73914	-4.91040	0.99567
C	6.32160	-3.23900	-8.48083	C	-5.58427	-4.90715	-0.12388
C	9.44616	-7.46244	3.77704	C	-5.07043	-4.90142	-1.42924
C	6.72701	-2.90491	-9.85013	C	-3.67958	-4.88778	-1.60114
C	10.57838	-7.93302	4.58648	C	-6.00084	-4.90196	-2.60013
C	5.81320	-3.00339	-10.90235	C	-5.32134	-4.96153	2.36752
C	6.23267	-2.69092	-12.19835	C	-5.97361	-5.92726	-3.55933
C	7.55023	-2.28328	-12.39181	C	-6.90206	-5.96547	-4.60280
C	8.40109	-2.20711	-11.28278	C	-7.86271	-4.95178	-4.72893
N	8.00838	-2.51105	-10.03851	C	-7.86006	-3.88673	-3.81844
N	10.82728	-7.27836	5.74734	C	-6.95589	-3.88205	-2.75738
C	11.89283	-7.64928	6.46993	C	-6.41814	-5.79624	2.65734

C	-6.99234	-5.83299	3.92655	H	7.92360	-2.02959	-13.37864
C	-6.46676	-5.04742	4.96077	H	9.43354	-1.89561	-11.40183
C	-5.37200	-4.21145	4.68946	H	12.06609	-7.10317	7.39182
C	-4.81305	-4.17187	3.41298	H	13.59908	-8.94807	6.70956
N	-7.03133	-2.86852	-7.57554	H	13.12158	-10.19099	4.57905
N	-8.87591	-2.47354	7.20316	H	11.14364	-9.49224	3.20688
C	-6.21558	-3.62625	-8.22328	H	-2.70686	-2.47831	-1.73704
C	-9.65575	-1.96230	8.09117	H	-1.63329	-0.45022	-2.69979
C	-6.65255	-4.80600	-8.97755	H	0.82028	-0.31147	-2.87894
C	-10.75933	-2.71617	8.70022	H	2.23554	-2.19249	-2.09300
N	-7.96445	-5.13498	-8.91424	H	2.43952	-6.97554	-2.27143
C	-8.38573	-6.19839	-9.61215	H	1.20714	-8.97673	-3.05451
C	-7.53436	-6.97874	-10.40292	H	-1.25198	-9.11552	-2.77299
C	-6.18513	-6.64007	-10.46910	H	-2.49458	-7.24496	-1.72821
C	-5.73678	-5.53514	-9.74034	H	-2.67640	-4.89507	1.64589
C	-11.64652	-2.08904	9.57794	H	-6.66124	-4.91524	0.01847
C	-12.71732	-2.82173	10.10099	H	-3.26990	-4.87767	-2.60691
C	-12.86144	-4.15392	9.72142	H	-5.24754	-6.73009	-3.46841
C	-11.92269	-4.71064	8.84338	H	-6.88243	-6.78611	-5.31480
N	-10.89015	-4.01811	8.34469	H	-8.59431	-3.09412	-3.91557
H	7.95770	-5.13949	-5.75876	H	-6.98562	-3.06572	-2.04099
H	6.95036	-5.65241	-3.56658	H	-6.80577	-6.45438	1.88525
H	4.10497	-2.47727	-4.13628	H	-7.81177	-6.51398	4.13446
H	5.10725	-1.96942	-6.32223	H	-4.98510	-3.56105	5.46854
H	6.65778	-4.68439	-1.27823	H	-3.98257	-3.49806	3.22300
H	3.07467	-5.19516	1.04810	H	-9.44236	-6.43447	-9.54281
H	2.81192	-4.24586	-3.12884	H	-7.93157	-7.82630	-10.95195
H	6.93720	-3.46177	1.11676	H	-5.49692	-7.21927	-11.07687
H	8.54528	-4.08436	2.88336	H	-4.69386	-5.23503	-9.76962
H	6.60403	-7.90421	3.28650	H	-11.50466	-1.04620	9.84517
H	4.97166	-7.26066	1.55378	H	-13.42121	-2.35961	10.78649
H	4.79527	-3.32788	-10.70964	H	-13.67877	-4.76145	10.09639
H	5.54663	-2.76693	-13.03624	H	-12.00152	-5.74919	8.53786

C	7.70379	0.26267	-6.82734	C	5.81072	10.94295	2.85079
C	7.76072	0.13775	-5.43286	C	1.25235	3.20986	-3.55495
C	6.85495	0.83567	-4.63520	C	0.56033	3.09282	-2.19671
C	5.84099	1.63431	-5.19463	C	-0.84029	3.03084	-2.31645
C	5.74435	1.67887	-6.59537	C	-1.30793	3.06256	-3.77175
C	6.67223	1.01727	-7.40545	C	-0.73583	4.28687	-4.47423
C	4.96441	2.47565	-4.31995	C	0.65927	4.37427	-4.34244
C	5.57386	3.39211	-3.44868	C	-1.42108	5.20472	-5.26718
C	4.81260	4.24476	-2.63546	C	-0.71672	6.23734	-5.89560
C	3.41473	4.14732	-2.69973	C	0.66660	6.33316	-5.75137
C	2.77740	3.21602	-3.53153	C	1.36166	5.39053	-4.98623
C	3.55939	2.39496	-4.35577	C	1.15534	2.92426	-0.94677
C	5.44766	5.23149	-1.71649	C	0.36050	2.75720	0.19240
C	6.55755	6.00679	-2.10136	C	-1.02694	2.71228	0.07221
C	7.13245	6.93574	-1.23342	C	-1.62913	2.82527	-1.18556
C	6.59533	7.13121	0.04593	C	-2.80780	2.86035	-3.95632
C	5.49605	6.35795	0.44838	C	-3.67861	3.83132	-3.43308
C	4.93837	5.42422	-0.42062	C	-5.06944	3.68942	-3.53082
N	8.74001	-0.33588	-7.62218	C	-5.58347	2.56153	-4.18781
N	7.14644	8.11889	0.92279	C	-4.73845	1.59323	-4.74995
C	9.44513	0.45851	-8.34989	C	-3.34477	1.75035	-4.61733
C	6.32031	8.96351	1.43663	C	-5.32072	0.43056	-5.47976
C	10.57746	-0.00676	-9.16225	C	-5.99980	4.70410	-2.94624
C	6.72510	9.98284	2.41031	C	-6.41716	0.59692	-6.34797
C	11.37732	0.90818	-9.85070	C	-6.99126	-0.48390	-7.01440
C	12.48277	0.44015	-10.56883	C	-6.46596	-1.77241	-6.85076
C	12.74916	-0.92691	-10.56615	C	-5.37158	-1.95545	-5.99065
C	11.89252	-1.77927	-9.85833	C	-4.81272	-0.86972	-5.31815
N	10.82688	-1.33931	-9.17573	C	-5.97631	6.04604	-3.35935
N	8.00655	9.95009	2.84544	C	-6.90473	6.96886	-2.87079
C	8.39873	10.87626	3.73042	C	-7.86150	6.57281	-1.92517
C	7.54726	11.87438	4.21872	C	-7.85476	5.25344	-1.45338
C	6.22964	11.90968	3.76903	C	-6.95084	4.33193	-1.97978

N	-8.87647	-5.00074	-5.74343
N	-8.87527	7.47548	-1.45991
C	-9.65658	-6.02517	-5.74482
C	-9.65629	7.98859	-2.34582
C	-10.75974	-6.17555	-6.70269
C	-10.75883	8.89373	-1.99640
N	-10.88987	-5.21685	-7.65273
C	-11.92202	-5.30244	-8.50231
C	-12.86109	-6.34090	-8.45935
C	-12.71770	-7.33551	-7.49510
C	-11.64728	-7.24897	-6.59859
C	-11.64727	9.34008	-2.97735
C	-12.71692	10.16047	-2.60337
C	-12.85864	10.49915	-1.25994
C	-11.91880	10.01719	-0.33987
N	-10.88740	9.23792	-0.69118
H	8.54596	-0.45668	-4.97760
H	6.93841	0.76242	-3.55441
H	4.97052	2.28153	-7.06238
H	6.60241	1.10227	-8.48659
H	6.65847	3.44721	-3.41534
H	2.81266	4.83211	-2.11166
H	3.07518	1.68847	-5.02114
H	6.95303	5.91167	-3.10863
H	7.95979	7.55405	-1.56800
H	5.10505	6.46073	1.45628
H	4.10339	4.82134	-0.07662
H	11.14199	1.96783	-9.82281
H	13.12017	1.12958	-11.11411
H	13.59853	-1.33680	-11.10306
H	12.06608	-2.85065	-9.84634
H	9.43127	10.82442	4.05954
H	7.92022	12.60265	4.93155
H	5.54315	12.67301	4.12193
H	4.79278	10.93757	2.47347
H	-2.49271	5.11892	-5.41096
H	-1.24915	6.95805	-6.50909
H	1.20993	7.13176	-6.24753
H	2.44138	5.45324	-4.90473
H	2.23522	2.91019	-0.85044
H	0.81916	2.65191	1.17120
H	-1.63427	2.56573	0.96057
H	-2.70706	2.74492	-1.27762
H	-3.26877	4.69760	-2.92212
H	-6.66046	2.44227	-4.26572
H	-2.67585	1.02201	-5.06161
H	-6.80456	1.59467	-6.53223
H	-7.81036	-0.32329	-7.70845
H	-4.98491	-2.95539	-5.81663
H	-3.98251	-1.04212	-4.63928
H	-5.25313	6.36746	-4.10344
H	-6.88778	7.99467	-3.22882
H	-8.58564	4.94265	-0.71449
H	-6.97760	3.30450	-1.62743
H	-12.00021	-4.51884	-9.24931
H	-13.67809	-6.36184	-9.17334
H	-13.42185	-8.16001	-7.43775
H	-11.50598	-8.00169	-5.82884
H	-11.50723	9.04913	-4.01407
H	-13.42172	10.52292	-3.34553
H	-13.67497	11.12854	-0.92054
H	-11.99584	10.27289	0.71219
C	-7.86179	-1.61970	6.65367
C	-7.85999	-1.36237	5.27632
C	-6.95563	-0.44592	4.74193
C	-5.99962	0.19891	5.54647

C	-5.97171	-0.12032	6.91372	C	1.25288	1.47348	4.55751
C	-6.90029	-1.00487	7.46842	C	0.56094	0.35646	3.77594
C	-5.06912	1.21263	4.96069	C	-0.83970	0.49075	3.78259
C	-5.58295	2.34591	4.31278	C	1.15605	-0.64080	3.00385
C	-4.73775	3.31693	3.75563	C	0.36129	-1.54344	2.28901
C	-3.34411	3.12329	3.82555	C	-1.02621	-1.41743	2.31082
C	-2.80732	1.99559	4.45599	C	-1.62849	-0.38546	3.03860
C	-3.67828	1.05679	5.03469	C	-1.42049	1.95706	7.14166
C	-5.31954	4.53031	3.11320	C	-0.71610	1.98417	8.35014
C	-6.41638	5.19925	3.69036	C	0.66721	1.81119	8.36084
C	-6.98993	6.31673	3.08683	C	1.36222	1.62061	7.16179
C	-6.46380	6.81884	1.88932	C	2.77791	1.45014	4.55115
C	-5.36907	6.16516	1.30172	C	3.55998	2.57457	4.25283
C	-4.81066	5.04010	1.90651	C	4.96501	2.50316	4.30511
N	-7.03226	-5.12700	6.27236	C	5.57435	1.29028	4.66298
N	-7.02852	7.99457	1.30185	C	4.81296	0.15952	4.99399
C	-6.21668	-5.30978	7.25246	C	3.41512	0.26389	4.94123
C	-6.21231	8.93371	0.96869	C	5.44772	-1.12993	5.38874
C	-6.65370	-5.37321	8.65125	C	5.84154	3.68137	4.01417
C	-6.64864	10.17698	0.32405	C	6.55732	-1.18481	6.25294
C	-5.73817	-5.67008	9.66407	C	7.13190	-2.40120	6.62306
C	-6.18662	-5.74867	10.98532	C	6.59473	-3.60660	6.15197
C	-7.53568	-5.52105	11.24554	C	5.49554	-3.56795	5.28100
C	-8.38681	-5.22561	10.17437	C	4.93821	-2.34826	4.90723
N	-7.96544	-5.15303	8.90451	C	5.74235	4.87351	4.75064
N	-7.96069	10.28785	0.00847	C	6.66978	5.90633	4.58284
C	-8.38124	11.42409	-0.56379	C	7.70360	5.78199	3.64291
C	-7.52902	12.49813	-0.84526	C	7.76350	4.63510	2.84014
C	-6.17968	12.38493	-0.51976	C	6.85800	3.59511	3.04552
C	-5.73206	11.20115	0.07293	N	7.14603	-4.85996	6.56804
C	-1.30743	1.73461	4.53865	N	8.73913	6.77047	3.52192
C	-0.73528	1.72989	5.95014	C	6.32022	-5.72796	7.04183
C	0.65981	1.57199	5.95976	C	9.44343	7.00462	4.57402

C	6.72561	-7.08088	7.43703
C	10.57530	7.94136	4.57733
N	8.00727	-7.44058	7.19119
C	8.40006	-8.67002	7.55034
C	7.54897	-9.59268	8.17006
C	6.23110	-9.22170	8.42530
C	5.81156	-7.94319	8.04765
C	11.37424	8.08150	5.71439
C	12.47950	8.93769	5.66808
C	12.74670	9.61764	4.48230
C	11.89090	9.42953	3.38977
N	10.82534	8.61832	3.42959
H	-8.59509	-1.84162	4.63860
H	-6.98609	-0.23262	3.67702
H	-5.24497	0.35886	7.56350
H	-6.88013	-1.21198	8.53494
H	-6.65992	2.47319	4.24842
H	-2.67500	3.87222	3.41730
H	-3.26856	0.18074	5.52881
H	-6.80445	4.86028	4.64640
H	-7.80926	6.83784	3.57221
H	-4.98166	6.51406	0.34892
H	-3.98016	4.53816	1.41851
H	-4.69536	-5.84616	9.41881
H	-5.49860	-5.98603	11.79079
H	-7.93295	-5.57259	12.25404
H	-9.44333	-5.04676	10.34409
H	-9.43799	11.48298	-0.80227
H	-7.92568	13.39756	-1.30490
H	-5.49080	13.20012	-0.71833
H	-4.68906	11.07555	0.34675
H	2.23594	-0.71679	2.94318
H	0.82008	-2.33781	1.70759

H	-1.63351	-2.11332	1.73949
H	-2.70647	-0.26592	3.01550
H	-2.49208	2.12472	7.13935
H	-1.24849	2.15464	9.28114
H	1.21058	1.84099	9.30053
H	2.44194	1.51868	7.17527
H	3.07585	3.50417	3.97400
H	6.65895	1.23391	4.69448
H	2.81294	-0.58796	5.23955
H	6.95285	-0.26519	6.67474
H	7.95899	-2.42112	7.32608
H	5.10435	-4.49190	4.86561
H	4.10338	-2.34427	4.21296
H	4.96676	4.97747	5.50415
H	6.59788	6.80116	5.19526
H	8.55076	4.53694	2.10004
H	6.94370	2.69470	2.44358
H	9.43282	-8.92842	7.34106
H	7.92242	-10.57403	8.44406
H	5.54488	-9.90955	8.90950
H	4.79340	-7.61435	8.23156
H	11.13840	7.52835	6.61850
H	13.11620	9.06626	6.53814
H	13.59604	10.28757	4.39569
H	12.06519	9.95374	2.45545
Zn	-9.20896	-4.77213	7.06381
Zn	-9.20819	-3.73095	-7.66500
Zn	-9.20530	8.50479	0.60062
Zn	9.25932	8.34705	1.86140
Zn	9.26045	-2.56205	-8.15799
Zn	9.25976	-5.78612	6.29671
C	-10.96914	0.97161	-0.99764
C	-10.96416	-0.38399	-1.36054

C	-10.96975	-1.34728	-0.34186	C	-10.90386	-1.24979	-7.76969
C	-10.96421	-0.98379	1.01355	C	-10.72534	-7.18481	5.93989
C	-10.96935	0.38005	1.33845	C	-11.34419	-8.42209	5.75182
C	-10.96369	1.37217	0.34597	C	-11.16001	-9.42446	6.71010
C	-10.87675	2.81272	0.70905	C	-10.35632	-9.14945	7.81321
C	-10.87766	-0.79012	-2.78957	C	-9.77066	-7.88162	7.92485
C	-10.87773	-2.01848	2.07960	N	-9.94796	-6.91311	7.01759
C	-11.43273	-1.82131	3.35868	C	-10.72347	-1.55153	-9.19369
C	-11.27294	-2.76231	4.37430	C	-11.34187	-0.77033	-10.17170
C	-10.58741	-3.95693	4.11929	C	-11.15666	-1.09919	-11.51875
C	-10.05805	-4.18594	2.84368	C	-10.35236	-2.19176	-11.83145
C	-10.18877	-3.22104	1.84980	C	-9.76717	-2.92202	-10.78879
N	-10.41097	-4.92819	5.15428	N	-9.94556	-2.62049	-9.49658
C	-11.43111	3.82209	-0.10140	H	-10.96376	1.72836	-1.77475
C	-11.27082	5.17207	0.20568	H	-10.96490	-2.39864	-0.60875
C	-10.58549	5.54837	1.36795	H	-10.96419	0.67458	2.38240
C	-10.05682	4.55794	2.20430	H	-12.01893	-0.93048	3.56171
C	-10.18800	3.21480	1.86560	H	-11.71829	-2.59240	5.34947
N	-10.40803	6.93027	1.69131	H	-9.50070	-5.09543	2.64699
C	-11.43347	-1.99609	-3.25829	H	-9.72038	-3.40007	0.88736
C	-11.27356	-2.40557	-4.58089	H	-12.01719	3.55275	-0.97454
C	-10.58704	-1.58817	-5.48789	H	-11.71568	5.93170	-0.42930
C	-10.05675	-0.36931	-5.04848	H	-9.49968	4.84212	3.09049
C	-10.18773	0.00942	-3.71605	H	-9.72021	2.47066	2.50213
N	-10.41036	-1.99928	-6.84643	H	-12.02037	-2.61670	-2.58836
C	-10.90126	7.35561	2.80194	H	-11.71957	-3.33474	-4.92127
C	-10.90488	-6.10229	4.96662	H	-9.49838	0.25499	-5.73764
C	-10.71980	8.73962	3.25250	H	-9.71878	0.93217	-3.38989
C	-11.33784	9.19660	4.41802	H	-11.49097	6.69543	3.44309
C	-11.15133	10.52740	4.80681	H	-11.49399	-6.32679	4.07375
C	-10.34614	11.34373	4.01707	H	-11.96100	8.52808	5.00441
C	-9.76149	10.80540	2.86328	H	-11.62668	10.91336	5.70342
N	-9.94117	9.53576	2.47823	H	-10.17003	12.38221	4.27801

H	-9.13125	11.41911	2.22814
H	-11.49305	-0.36425	-7.51803
H	-11.96624	-8.59500	4.87875
H	-11.63604	-10.39350	6.59525
H	-10.18214	-9.89483	8.58237
H	-9.14167	-7.63905	8.77501
H	-11.96436	0.07209	-9.88547
H	-11.63235	-0.51547	-12.30094
H	-10.17733	-2.48522	-12.86139
H	-9.13762	-3.77930	-11.00323
C	10.99255	-1.38795	-0.07891
C	10.98775	-0.63446	-1.26282
C	10.99342	0.76370	-1.16223
C	10.98852	1.41223	0.08228
C	10.99354	0.62602	1.24284
C	10.98787	-0.77604	1.18229
C	10.91028	-1.59191	2.42459
C	10.91007	-1.30244	-2.59046
C	10.91117	2.89601	0.16751
C	11.43723	3.61049	1.26124
C	11.29414	4.99299	1.36554
C	10.65872	5.70841	0.34297
C	10.15651	5.01974	-0.76714
C	10.26605	3.63485	-0.83822
N	10.51291	7.12911	0.43082
C	11.43440	-2.89719	2.49563
C	11.29111	-3.67897	3.64056
C	10.65775	-3.15053	4.77231
C	10.15734	-1.84406	4.73175
C	10.26683	-1.08986	3.56806
N	10.51213	-3.93730	5.95847
C	11.43709	-0.71295	-3.75587
C	11.29435	-1.31396	-5.00528
C	10.65828	-2.55689	-5.11372
C	10.15463	-3.17327	-3.96253
C	10.26387	-2.54227	-2.72765
N	10.51332	-3.19151	-6.38799
C	11.07632	-3.50534	7.03240
C	11.07762	7.84285	-0.48015
C	10.94423	-4.20274	8.31530
C	11.63719	-3.75663	9.44228
C	11.49730	-4.45727	10.64494
C	10.66223	-5.57087	10.67100
C	10.00199	-5.95004	9.49483
N	10.13588	-5.29149	8.33683
C	11.07876	-4.33698	-6.55018
C	10.94552	9.30256	-0.51835
C	11.63892	10.05504	-1.46821
C	11.49895	11.44689	-1.46359
C	10.66327	12.02670	-0.51301
C	10.00254	11.19811	0.40348
N	10.13659	9.86602	0.41302
C	10.94762	-5.10009	-7.79524
C	11.64168	-6.29860	-7.97164
C	11.50254	-6.99061	-9.17937
C	10.66704	-6.45771	-10.15716
C	10.00566	-5.24998	-9.89817
N	10.13890	-4.57560	-8.74926
H	10.98955	-2.47057	-0.14017
H	10.99117	1.35795	-2.06926
H	10.99136	1.11448	2.21095
H	11.99075	3.08646	2.03400
H	11.71814	5.52234	2.21311
H	9.63904	5.56371	-1.54969
H	9.81794	3.12280	-1.68339
H	11.98656	-3.30500	1.65483

H	11.71347	-4.67838	3.67437	H	10.52521	-6.95787	-11.10968
H	9.64111	-1.43781	5.59462	H	9.35046	-4.81182	-10.64357
H	9.81993	-0.10135	3.54797	H	5.26790	-3.48828	-8.33194
H	11.99123	0.21792	-3.68819	H	9.25540	-8.00344	2.84602
H	11.71926	-0.84506	-5.88730	H	-9.54919	-0.91799	8.39746
H	9.63645	-4.12257	-4.04253	H	9.25401	1.53524	-8.35285
H	9.81485	-3.01767	-1.86182	H	5.26665	8.95862	1.14618
H	11.69320	-2.60323	7.01664	H	-9.55147	7.73094	-3.40335
H	11.69490	7.37782	-1.25302	H	-5.14856	-5.45314	7.07002
H	12.28131	-2.88480	9.37824	H	-5.14407	8.84648	1.18311
H	12.03054	-4.13936	11.53567	H	5.26642	-5.47459	7.18286
H	10.51990	-6.14628	11.57996	H	9.25198	6.46934	5.50821
H	9.34722	-6.81498	9.48702	O	-0.63631	-4.75878	0.55330
H	11.69594	-4.77332	-5.76068	O	0.83238	-4.70427	0.41875
H	12.28348	9.56333	-2.19061	O	0.83348	2.71414	3.86462
H	12.03254	12.05899	-2.18430	O	-0.63521	2.85785	3.84492
H	10.52080	13.10159	-0.46983	O	0.83299	1.98862	-4.28156
H	9.34714	11.62409	1.15573	O	-0.63565	1.89985	-4.39680
H	12.28609	-6.67803	-7.18432	H	-5.14738	-3.39686	-8.25569
H	12.03664	-7.92058	-9.34886	H	-9.55060	-6.81248	-4.99338

Table S5. Cartesian coordinates (in Å) for the B3LYP 6-31G(d) model of cage **5**.

Zn	14.36888	0.00571	0.00019	C	-6.89200	-13.70631	-5.89086
Zn	4.84231	0.07382	-13.08554	C	-7.40302	-14.76340	-5.12769
N	15.68982	-1.81211	0.29962	C	-6.81293	-15.04962	-3.89888
N	13.32934	-1.70810	-1.19887	C	-5.73508	-14.26587	-3.47590
C	16.80719	-1.91971	1.03091	C	-5.28416	-13.23003	-4.29812
C	17.62958	-3.05347	1.01055	C	-4.13727	-12.40941	-3.89736
C	17.27828	-4.12392	0.19211	C	-2.53338	-10.74799	-4.10948
C	16.10844	-4.02502	-0.56774	C	-1.36910	-10.56504	-4.86905
C	15.34168	-2.85976	-0.48589	C	-0.24531	-9.95866	-4.30391
C	14.10163	-2.73860	-1.25772	C	-0.25342	-9.50290	-2.97400
C	12.14341	-1.77761	-2.00762	C	-1.45440	-9.62609	-2.25188
C	10.88584	-1.71232	-1.39335	C	-2.57615	-10.23870	-2.80201
C	9.73312	-1.94662	-2.14257	C	3.14745	-11.00995	3.52602
C	9.79009	-2.22157	-3.52208	C	4.22021	-10.62311	2.71042
C	11.05486	-2.20777	-4.13385	C	3.98215	-10.01188	1.47978
C	12.21792	-1.99964	-3.39050	C	2.67556	-9.73462	1.03649
N	6.41320	1.48437	-13.83214	C	1.61539	-10.06960	1.89788
N	6.44754	0.14952	-11.36574	C	1.84030	-10.70186	3.11542
C	6.42865	2.15375	-14.99275	C	2.99138	-7.64458	-2.14970
C	7.49469	2.96952	-15.39071	C	1.85206	-8.05605	-2.85207
C	8.59272	3.09850	-14.54371	C	0.97118	-8.99862	-2.28611
C	8.58100	2.41099	-13.32668	C	1.25985	-9.52095	-1.01805
C	7.47790	1.61539	-13.00348	C	2.40011	-9.13486	-0.29806
C	7.44802	0.87533	-11.73951	C	3.25737	-8.19399	-0.88449
C	6.58962	-0.58156	-10.14638	N	3.58895	-13.10984	7.15264
C	5.50727	-0.68920	-9.26005	N	3.38279	-11.72498	4.73931
C	5.67042	-1.31177	-8.02046	C	3.66815	-13.80207	8.29627
C	6.90664	-1.85846	-7.62915	C	2.89974	-14.94515	8.55208
C	7.96102	-1.80685	-8.55954	C	2.01211	-15.38974	7.57539
C	7.81015	-1.18635	-9.79550	C	1.91782	-14.66903	6.38058
N	-5.85657	-12.95469	-5.49455	C	2.71766	-13.53647	6.20558
N	-3.63908	-11.48270	-4.64402	C	2.64234	-12.75797	4.96673

C	8.55721	-2.57852	-4.28332
C	8.29751	-2.08153	-5.56967
C	7.13327	-2.43746	-6.27159
Zn	4.83553	11.30023	6.60515
N	15.68799	0.65716	-1.72389
N	13.32690	1.90051	-0.88299
C	16.80553	0.07869	-2.18369
C	17.62653	0.66394	-3.15610
C	17.27364	1.90760	-3.67367
C	16.10364	2.51510	-3.20710
C	15.33832	1.86090	-2.23821
C	14.09810	2.46760	-1.74639
C	12.14053	2.63456	-0.53805
C	12.21431	3.94287	-0.03797
C	11.05083	4.68984	0.15425
C	9.78632	4.16633	-0.16363
C	9.73011	2.83442	-0.61613
C	10.88327	2.06931	-0.78870
N	6.40678	11.24481	8.19961
N	6.44180	9.77311	5.81227
C	6.42211	11.91695	9.35862
C	7.48872	11.85628	10.26357
C	8.58755	11.05918	9.95229
C	8.57603	10.34724	8.74937
C	7.47228	10.46256	7.89922
C	7.44273	9.73625	6.62730
C	6.58422	9.08154	4.57009
C	5.50239	8.36625	4.03470
C	5.66569	7.60370	2.87596
C	6.90154	7.53930	2.20609
C	7.95545	8.32035	2.71521
C	7.80442	9.08085	3.87028
N	-5.86669	11.23041	-8.47040
N	-3.64857	9.75910	-7.62028
C	-6.90267	11.94854	-8.92332
C	-7.41380	11.81527	-10.22023
C	-6.82320	10.89424	-11.08221
C	-5.74482	10.13690	-10.61475
C	-5.29390	10.33186	-9.30669
C	-4.14675	9.57512	-8.79606
C	-2.54252	8.92948	-7.25094
C	-1.37886	9.49651	-6.71213
C	-0.25453	8.70469	-6.46944
C	-0.26152	7.32514	-6.74008
C	-1.46200	6.76057	-7.20822
C	-2.58423	7.54251	-7.46384
C	3.14155	2.45283	-11.29642
C	1.83451	2.65243	-10.82319
C	1.60950	3.39024	-9.66659
C	2.66945	3.97012	-8.94658
C	3.97602	3.72659	-9.40931
C	4.21420	2.96699	-10.55432
C	2.98507	5.68503	-5.54374
C	1.84503	6.49800	-5.54856
C	0.96371	6.47820	-6.64748
C	1.25273	5.64128	-7.73383
C	2.39371	4.82574	-7.75979
C	3.25136	4.86405	-6.65210
N	3.58440	0.36541	-14.93022
N	3.37691	1.76043	-12.52277
C	3.66418	-0.27753	-16.10223
C	2.89447	0.07211	-17.21930
C	2.00478	1.13838	-17.11440
C	1.90992	1.81136	-15.89212
C	2.71119	1.39714	-14.82467
C	2.63547	2.07960	-13.53046

C	8.55294	5.00322	-0.09147
C	8.29303	5.86827	0.98245
C	7.12829	6.65331	1.02575
C	6.21088	6.57794	-0.04204
C	6.46026	5.73983	-1.13413
C	7.62393	4.95609	-1.14051
Zn	4.84592	-11.36623	6.48262
N	15.68727	1.17437	1.42594
N	13.32693	-0.17673	2.08300
C	16.80425	1.86253	1.15447
C	17.62525	2.41249	2.14727
C	17.27300	2.23843	3.48321
C	16.10362	1.52975	3.77643
C	15.33819	1.01746	2.72567
C	14.09846	0.28747	3.00554
C	12.14104	-0.84304	2.54667
C	10.88349	-0.34333	2.18354
C	9.73077	-0.87571	2.76042
C	9.78779	-1.93390	3.68718
C	11.05258	-2.47095	3.98046
C	12.21563	-1.93067	3.42909
N	6.41812	-12.71813	5.63663
N	6.45041	-9.91470	5.55600
C	6.43473	-14.05791	5.63901
C	7.50139	-14.81023	5.13251
C	8.59882	-14.14101	4.59635
C	8.58593	-12.74329	4.58138
C	7.48229	-12.06581	5.10797
C	7.45140	-10.60115	5.11520
C	6.59167	-8.49305	5.57838
C	7.81175	-7.88578	5.92657
C	7.96155	-6.50506	5.84573
C	6.90656	-5.67437	5.42509
C	5.67092	-6.28762	5.14705
C	5.50883	-7.67255	5.22786
N	-5.86429	1.71446	13.96371
N	-3.64649	1.71713	12.26419
C	-6.89971	1.74592	14.81283
C	-7.41176	2.93483	15.34697
C	-6.82272	4.14271	14.98068
C	-5.74481	4.11829	14.09046
C	-5.29286	2.88881	13.60422
C	-4.14577	2.82667	12.69329
C	-2.54060	1.81348	11.36100
C	-1.37604	1.06469	11.58255
C	-0.25213	1.25147	10.77514
C	-0.26034	2.17545	9.71558
C	-1.46158	2.86192	9.46100
C	-2.58345	2.69127	10.26631
C	3.13699	8.55988	7.77145
C	1.83029	8.04924	7.70884
C	1.60642	6.67852	7.77002
C	2.66721	5.76590	7.91158
C	3.97341	6.28942	7.93073
C	4.21043	7.66100	7.84485
C	2.98542	1.96170	7.69539
C	1.84605	1.55862	8.40250
C	0.96433	2.51961	8.93525
C	1.25233	3.87908	8.75346
C	2.39266	4.31010	8.05953
C	3.25072	3.33223	7.53845
N	3.57609	12.75092	7.77913
N	3.37125	9.96829	7.78466
C	3.65443	14.08748	7.80755
C	2.88394	14.87973	8.66848
C	1.99499	14.25528	9.53987

C	1.90161	12.86014	9.51234
C	2.70361	12.14317	8.62024
C	2.62932	10.68101	8.56481
C	8.55501	-2.41546	4.37653
C	8.29613	-3.77818	4.58896
C	7.13206	-4.20898	5.24787
C	6.21418	-3.24716	5.71682
C	6.46245	-1.88218	5.53667
C	7.62554	-1.48401	4.86053
Zn	-14.36910	-0.00587	-0.00052
Zn	-4.84231	-0.07336	13.08586
N	-15.68921	1.81244	-0.30015
N	-13.32862	1.70772	1.19820
C	-16.80654	1.92044	-1.03145
C	-17.62842	3.05457	-1.01123
C	-17.27665	4.12497	-0.19292
C	-16.10685	4.02565	0.56693
C	-15.34059	2.86005	0.48521
C	-14.10057	2.73848	1.25704
C	-12.14268	1.77686	2.00694
C	-10.88513	1.71117	1.39267
C	-9.73234	1.94518	2.14187
C	-9.78922	2.22021	3.52136
C	-11.05399	2.20677	4.13315
C	-12.21712	1.99895	3.38981
N	-6.41301	-1.48412	13.83246
N	-6.44695	-0.15033	11.36547
C	-6.42859	-2.15312	14.99328
C	-7.49456	-2.96897	15.39128
C	-8.59238	-3.09844	14.54409
C	-8.58052	-2.41133	13.32682
C	-7.47749	-1.61564	13.00359
C	-7.44747	-0.87604	11.73936
C	-6.58896	0.58045	10.14591
C	-7.80943	1.18529	9.79490
C	-7.96021	1.80558	8.55883
C	-6.90581	1.85693	7.62846
C	-5.66965	1.31017	8.01987
C	-5.50659	0.68780	9.25958
N	5.85615	12.95498	5.49450
N	3.63934	11.48209	4.64377
C	6.89133	13.70692	5.89083
C	7.40229	14.76392	5.12749
C	6.81240	15.04970	3.89849
C	5.73480	14.26560	3.47548
C	5.28395	13.22986	4.29787
C	4.13736	12.40885	3.89705
C	2.53387	10.74707	4.10918
C	1.36959	10.56384	4.86868
C	0.24596	9.95724	4.30346
C	0.25424	9.50153	2.97353
C	1.45524	9.62497	2.25149
C	2.57684	10.23780	2.80171
C	-3.14672	11.00887	-3.52635
C	-1.83955	10.70072	-3.11588
C	-1.61455	10.06829	-1.89844
C	-2.67466	9.73319	-1.03702
C	-3.98129	10.01048	-1.48020
C	-4.21943	10.62189	-2.71074
C	-2.99031	7.64288	2.14900
C	-1.85104	8.05441	2.85143
C	-0.97024	8.99710	2.28554
C	-1.25892	9.51947	1.01750
C	-2.39914	9.13332	0.29746
C	-3.25633	8.19234	0.88382
N	-3.58876	13.10989	-7.15224

N	-3.38214	11.72418	-4.73945
C	-3.66808	13.80242	-8.29568
C	-2.89944	14.94537	-8.55140
C	-2.01145	15.38950	-7.57482
C	-1.91704	14.66845	-6.38022
C	-2.71710	13.53604	-6.20531
C	-2.64164	12.75717	-4.96670
C	-8.55626	2.57696	4.28255
C	-8.29661	2.07998	5.56892
C	-7.13233	2.43582	6.27082
C	-6.21513	3.32314	5.67175
C	-6.46424	3.84931	4.39960
C	-7.62746	3.46218	3.71731
Zn	-4.83583	-11.29979	-6.60492
N	-15.68861	-0.65660	1.72369
N	-13.32738	-1.90022	0.88348
C	-16.80618	-0.07795	2.18318
C	-17.62734	-0.66291	3.15564
C	-17.27455	-1.90644	3.67360
C	-16.10449	-2.51411	3.20738
C	-15.33902	-1.86020	2.23841
C	-14.09874	-2.46706	1.74691
C	-12.14103	-2.63442	0.53880
C	-10.88375	-2.06926	0.78952
C	-9.73062	-2.83443	0.61701
C	-9.78687	-4.16633	0.16449
C	-11.05141	-4.68978	-0.15341
C	-12.21485	-3.94275	0.03875
N	-6.40708	-11.24405	-8.19938
N	-6.44210	-9.77253	-5.81187
C	-6.42243	-11.91604	-9.35849
C	-7.48905	-11.85523	-10.26343
C	-8.58785	-11.05814	-9.95204
C	-8.57631	-10.34635	-8.74903
C	-7.47256	-10.46179	-7.89891
C	-7.44301	-9.73562	-6.62690
C	-6.58457	-9.08108	-4.56962
C	-7.80478	-9.08053	-3.86983
C	-7.95588	-8.32015	-2.71469
C	-6.90201	-7.53908	-2.20548
C	-5.66615	-7.60334	-2.87535
C	-5.50279	-8.36578	-4.03415
N	5.86674	-11.23001	8.47061
N	3.64849	-9.75874	7.62059
C	6.90278	-11.94806	8.92350
C	7.41402	-11.81468	10.22036
C	6.82347	-10.89359	11.08231
C	5.74503	-10.13633	10.61488
C	5.29400	-10.33140	9.30687
C	4.14680	-9.57470	8.79631
C	2.54241	-8.92914	7.25131
C	1.37870	-9.49620	6.71265
C	0.25435	-8.70440	6.46998
C	0.26136	-7.32483	6.74052
C	1.46188	-6.76023	7.20853
C	2.58413	-7.54215	7.46411
C	-3.14154	-2.45234	11.29675
C	-4.21422	-2.96654	10.55472
C	-3.97609	-3.72618	9.40974
C	-2.66953	-3.96972	8.94696
C	-1.60956	-3.38980	9.66690
C	-1.83453	-2.65195	10.82348
C	-2.98531	-5.68483	5.54425
C	-1.84526	-6.49779	5.54906
C	-0.96388	-6.47790	6.64792
C	-1.25284	-5.64092	7.73424

C	-2.39384	-4.82540	7.76021
C	-3.25155	-4.86379	6.65257
N	-3.58432	-0.36480	14.93044
N	-3.37685	-1.75990	12.52308
C	-3.66411	0.27820	16.10243
C	-2.89436	-0.07134	17.21950
C	-2.00462	-1.13757	17.11463
C	-1.90974	-1.81061	15.89238
C	-2.71106	-1.39648	14.82493
C	-2.63536	-2.07900	13.53075
C	-8.55351	-5.00325	0.09229
C	-8.29359	-5.86823	-0.98168
C	-7.12882	-6.65322	-1.02506
C	-6.21138	-6.57787	0.04272
C	-6.46076	-5.73982	1.13485
C	-7.62447	-4.95612	1.14130
Zn	-4.84605	11.36651	-6.48237
N	-15.68754	-1.17467	-1.42626
N	-13.32739	0.17684	-2.08334
C	-16.80456	-1.86279	-1.15486
C	-17.62558	-2.41266	-2.14769
C	-17.27331	-2.23855	-3.48362
C	-16.10387	-1.52993	-3.77679
C	-15.33845	-1.01771	-2.72599
C	-14.09876	-0.28764	-3.00587
C	-12.14156	0.84326	-2.54698
C	-12.21620	1.93077	-3.42954
C	-11.05316	2.47117	-3.98082
C	-9.78833	1.93434	-3.68733
C	-9.73127	0.87626	-2.76044
C	-10.88397	0.34378	-2.18364
N	-6.41812	12.71860	-5.63645
N	-6.45065	9.91521	-5.55568
C	-6.43459	14.05838	-5.63889
C	-7.50117	14.81083	-5.13240
C	-8.59867	14.14174	-4.59620
C	-8.58592	12.74402	-4.58117
C	-7.48236	12.06641	-5.10775
C	-7.45160	10.60175	-5.11492
C	-6.59200	8.49357	-5.57807
C	-5.50922	7.67302	-5.22753
C	-5.67136	6.28809	-5.14678
C	-6.90701	5.67490	-5.42494
C	-7.96195	6.50565	-5.84559
C	-7.81208	7.88638	-5.92636
N	5.86407	-1.71411	-13.96341
N	3.64619	-1.71661	-12.26393
C	6.89951	-1.74567	-14.81250
C	7.41141	-2.93462	-15.34670
C	6.82220	-4.14244	-14.98049
C	5.74426	-4.11793	-14.09030
C	5.29246	-2.88840	-13.60400
C	4.14537	-2.82618	-12.69310
C	2.54027	-1.81294	-11.36079
C	1.37576	-1.06407	-11.58234
C	0.25179	-1.25085	-10.77501
C	0.25991	-2.17490	-9.71551
C	1.46110	-2.86145	-9.46091
C	2.58302	-2.69081	-10.26616
C	-3.13760	-8.55931	-7.77157
C	-4.21104	-7.66041	-7.84492
C	-3.97400	-6.28883	-7.93081
C	-2.66779	-5.76534	-7.91170
C	-1.60701	-6.67797	-7.77021
C	-1.83090	-8.04868	-7.70903
C	-2.98592	-1.96113	-7.69544

C	-1.84652	-1.55807	-8.40251	H	7.34018	7.75392	-7.00851
C	-0.96481	-2.51906	-8.93527	H	8.87567	7.77846	-5.06115
C	-1.25286	-3.87852	-8.75353	H	8.15251	6.83186	-2.89319
C	-2.39322	-4.30954	-8.05964	C	3.39355	3.10419	-4.03964
C	-3.25126	-3.33166	-7.53855	C	3.57010	1.95087	-3.26824
N	-3.57651	-12.75038	-7.77914	C	4.43271	1.96594	-2.17406
N	-3.37187	-9.96772	-7.78477	C	5.12631	3.13393	-1.84067
C	-3.65478	-14.08695	-7.80755	H	2.69389	3.09480	-4.86787
C	-2.88436	-14.87915	-8.66859	H	3.02098	1.04724	-3.51813
C	-1.99558	-14.25465	-9.54011	H	4.55857	1.07173	-1.56881
C	-1.90227	-12.85950	-9.51259	H	5.76601	3.15199	-0.96490
C	-2.70418	-12.14258	-8.62038	C	7.48871	-0.13991	7.74713
C	-2.62998	-10.68041	-8.56497	C	7.89367	0.64823	8.82944
C	-8.55554	2.41595	-4.37660	C	7.03024	1.60288	9.36485
C	-8.29666	3.77868	-4.58899	C	5.75307	1.77924	8.82247
C	-7.13256	4.20951	-5.24781	C	5.35902	1.01654	7.72451
C	-6.21465	3.24771	-5.71675	C	6.22799	0.05473	7.18552
C	-6.46291	1.88273	-5.53665	C	5.57117	-0.77285	6.08351
C	-7.62603	1.48452	-4.86057	C	4.98220	0.14583	5.01495
C	6.21616	-3.32490	-5.67255	C	4.11241	1.10848	5.55445
C	6.46533	-3.85109	-4.40041	C	3.98077	0.98915	7.07247
C	7.62851	-3.46387	-3.71811	H	8.15475	-0.90350	7.36121
C	5.75240	6.75384	-5.94830	H	8.87748	0.50096	9.26498
C	7.02925	7.31255	-6.06624	H	7.34104	2.19839	10.21816
C	7.89215	7.32694	-4.97136	H	5.07353	2.49670	9.26941
C	7.48696	6.78357	-3.74779	C	5.12686	0.03211	3.63372
C	6.22662	6.19897	-3.63609	C	4.43264	0.90459	2.78916
C	5.35818	6.18416	-4.73894	C	3.56959	1.85914	3.32351
C	3.98005	5.63252	-4.38968	C	3.39323	1.95019	4.70807
C	4.11209	4.25839	-3.73378	H	5.76688	-0.73495	3.21122
C	4.98143	4.27307	-2.63000	H	4.55830	0.82774	1.71209
C	5.56956	5.65815	-2.36861	H	3.02000	2.52721	2.66610
H	5.07320	6.78173	-6.79340	H	2.69321	2.67170	5.11422

C	7.49341	-6.63595	-3.99716
C	7.89930	-7.96704	-3.85572
C	7.03671	-8.90851	-3.29614
C	5.75945	-8.52773	-2.87183
C	5.36448	-7.19581	-2.98347
C	6.23265	-6.24763	-3.54732
C	5.57498	-4.87983	-3.71269
C	4.98634	-4.41420	-2.38250
C	4.11733	-5.36327	-1.81824
C	3.98600	-6.61823	-2.68066
H	8.15877	-5.91959	-4.46592
H	8.88317	-8.27001	-4.20139
H	7.34824	-9.94503	-3.20700
H	5.08052	-9.27389	-2.47360
C	5.13059	-3.16116	-1.79027
C	4.43679	-2.86652	-0.61202
C	3.57457	-3.80712	-0.05219
C	3.39858	-5.05171	-0.66571
H	5.76998	-2.41135	-2.24355
H	4.56218	-1.89532	-0.13998
H	3.02530	-3.57224	0.85538
H	2.69918	-5.76471	-0.24376
C	-7.48916	0.14056	-7.74717
C	-7.89413	-0.64752	-8.82952
C	-7.03070	-1.60217	-9.36496
C	-5.75354	-1.77858	-8.82257
C	-5.35950	-1.01592	-7.72458
C	-6.22845	-0.05411	-7.18556
C	-5.57163	0.77341	-6.08351
C	-4.98270	-0.14532	-5.01498
C	-4.11292	-1.10797	-5.55449
C	-3.98126	-0.98858	-7.07250
H	-8.15519	0.90415	-7.36123
H	-8.87792	-0.50022	-9.26507
H	-7.34150	-2.19764	-10.21830
H	-5.07400	-2.49602	-9.26953
C	-5.12737	-0.03165	-3.63374
C	-4.43317	-0.90417	-2.78920
C	-3.57014	-1.85872	-3.32358
C	-3.39377	-1.94972	-4.70813
H	-5.76737	0.73542	-3.21122
H	-4.55884	-0.82735	-1.71214
H	-3.02057	-2.52682	-2.66619
H	-2.69375	-2.67122	-5.11430
C	-5.75844	8.52591	2.87092
C	-7.03575	8.90665	3.29514
C	-7.89834	7.96516	3.85470
C	-7.49239	6.63409	3.99619
C	-6.23158	6.24581	3.54645
C	-5.36343	7.19401	2.98262
C	-3.98490	6.61648	2.67992
C	-4.11612	5.36149	1.81752
C	-4.98513	4.41240	2.38175
C	-5.57388	4.87804	3.71189
H	-5.07952	9.27209	2.47270
H	-7.34731	9.94315	3.20594
H	-8.88223	8.26809	4.20028
H	-8.15775	5.91772	4.46493
C	-3.39727	5.04992	0.66505
C	-3.57318	3.80530	0.05155
C	-4.43539	2.86469	0.61135
C	-5.12928	3.15933	1.78954
H	-2.69787	5.76293	0.24313
H	-3.02383	3.57042	-0.85596
H	-4.56071	1.89347	0.13934
H	-5.76866	2.40950	2.24281

C	-3.39393	-3.10406	4.04010	H	9.44119	3.71555	-14.82337
C	-3.57055	-1.95077	3.26868	H	9.41938	2.48378	-12.64060
C	-4.43324	-1.96587	2.17456	H	8.33241	0.97734	-11.10604
C	-5.12683	-3.13389	1.84124	H	4.55561	-0.23450	-9.51699
C	-4.98187	-4.27301	2.63059	H	4.83248	-1.33591	-7.32944
C	-4.11246	-4.25828	3.73431	H	8.90997	-2.27747	-8.32099
C	-3.98036	-5.63239	4.39024	H	8.63659	-1.19764	-10.50012
C	-5.35845	-6.18405	4.73960	H	-7.32519	-13.46303	-6.85579
C	-6.22696	-6.19891	3.63680	H	-8.23922	-15.34593	-5.50079
C	-5.57000	-5.65810	2.36927	H	-7.17786	-15.86702	-3.28445
H	-2.69421	-3.09463	4.86828	H	-5.24509	-14.46148	-2.52701
H	-3.02143	-1.04711	3.51852	H	-3.69203	-12.64156	-2.92688
H	-4.55915	-1.07168	1.56930	H	-1.31803	-10.96903	-5.87440
H	-5.76658	-3.15198	0.96551	H	0.66981	-9.89429	-4.88577
C	-5.75259	-6.75370	5.94900	H	-1.50996	-9.23859	-1.23891
C	-7.02942	-7.31243	6.06704	H	-3.49338	-10.30861	-2.22479
C	-7.89239	-7.32687	4.97221	H	5.23525	-10.86082	3.00994
C	-7.48728	-6.78352	3.74860	H	4.82738	-9.78604	0.83654
H	-5.07334	-6.78155	6.79406	H	0.59633	-9.82051	1.61924
H	-7.34029	-7.75378	7.00934	H	1.00086	-10.93084	3.76472
H	-8.87590	-7.77840	5.06208	H	1.64262	-7.63096	-3.82764
H	-8.15288	-6.83185	2.89404	H	0.61229	-10.29113	-0.61197
H	17.06663	-1.06690	1.64832	H	4.13910	-7.86601	-0.34263
H	18.52755	-3.08099	1.61946	H	4.37439	-13.43741	9.03471
H	17.89830	-5.01390	0.14205	H	3.00771	-15.46964	9.49602
H	15.79790	-4.83774	-1.21742	H	1.40788	-16.27751	7.73567
H	13.83516	-3.59628	-1.88073	H	1.23912	-14.98484	5.59407
H	10.81689	-1.54174	-0.32300	H	1.92196	-13.10176	4.22051
H	8.76911	-1.92799	-1.64207	H	8.98931	-1.37333	-6.01671
H	11.14257	-2.41660	-5.19603	H	17.06637	-0.88209	-1.75406
H	13.18562	-2.02785	-3.88353	H	18.52471	0.15119	-3.48511
H	5.56407	2.02758	-15.63549	H	17.89258	2.39650	-4.41992
H	7.45777	3.47991	-16.34768	H	15.79186	3.48380	-3.58584

H	13.83050	3.43580	-2.17737	H	1.91391	2.89660	-13.45408
H	13.18179	4.38441	0.18417	H	8.98504	5.90154	1.81910
H	11.13798	5.71394	0.50515	H	5.32357	7.20159	-0.03873
H	8.76632	2.39117	-0.85026	H	7.83277	4.32806	-2.00099
H	10.81483	1.05734	-1.17690	H	17.06457	1.97098	0.10747
H	5.55694	12.53600	9.57027	H	18.52296	2.95443	1.86746
H	9.43647	10.99482	10.62612	H	17.89197	2.64058	4.27956
H	9.41505	9.71735	8.46979	H	15.79238	1.37302	4.80483
H	8.32782	9.13758	6.39921	H	13.83150	0.17604	4.05962
H	4.55101	8.36048	4.55744	H	10.81460	0.49908	1.50157
H	4.82812	7.01635	2.51027	H	8.76676	-0.45134	2.49441
H	8.90410	8.34982	2.18786	H	11.14032	-3.28720	4.69149
H	8.63050	9.69762	4.21204	H	13.18338	-2.34376	3.69952
H	-7.33624	12.66253	-8.23039	H	5.57055	-14.55163	6.07026
H	-8.25045	12.42897	-10.53831	H	7.46539	-15.89417	5.17045
H	-7.18816	10.77018	-12.09722	H	9.44772	-14.69153	4.20246
H	-5.25446	9.41297	-11.25834	H	9.42384	-12.18539	4.17454
H	-3.70123	8.85066	-9.48208	H	8.33555	-10.10339	4.70981
H	-1.32865	10.56916	-6.55907	H	8.63868	-8.48961	6.28878
H	0.66014	9.17702	-6.12242	H	8.91015	-6.06239	6.13403
H	-1.51676	5.68955	-7.37927	H	4.83258	-5.67784	4.82213
H	-3.50102	7.07694	-7.81329	H	4.55762	-8.12316	4.96229
H	0.99530	2.20342	-11.34550	H	-7.33186	0.78813	15.08453
H	0.59057	3.50552	-9.31068	H	-8.24790	2.90210	16.03805
H	4.82106	4.17171	-8.89263	H	-7.18847	5.08311	15.38156
H	5.22913	2.82792	-10.91082	H	-5.25557	5.03835	13.78571
H	1.63538	7.13028	-4.69266	H	-3.70131	3.78362	12.40928
H	0.60491	5.67403	-8.60370	H	-1.32491	0.39595	12.43502
H	4.13361	4.23151	-6.63920	H	0.66316	0.71574	11.01052
H	4.37196	-1.09795	-16.15692	H	-1.51730	3.54541	8.61893
H	3.00299	-0.48198	-18.14610	H	-3.50085	3.22574	10.03804
H	1.39943	1.44310	-17.96270	H	0.99044	8.72538	7.58168
H	1.22965	2.64895	-15.77110	H	0.58773	6.31183	7.69283

H	4.81907	5.62007	8.05729	H	7.17728	15.86701	3.28393
H	5.22509	8.04020	7.90154	H	5.24498	14.46085	2.52643
H	1.63727	0.50110	8.52228	H	3.69219	12.64073	2.92648
H	0.60422	4.61559	9.21706	H	1.31836	10.96782	5.87403
H	4.13251	3.63788	6.98377	H	-0.66919	9.89269	4.88525
H	4.36168	14.54538	7.12406	H	1.51094	9.23752	1.23851
H	2.99131	15.95952	8.65142	H	3.49408	10.30794	2.22453
H	1.38910	14.83730	10.22766	H	-1.00015	10.92982	-3.76519
H	1.22195	12.33619	10.17756	H	-0.59547	9.81920	-1.61988
H	1.90834	10.20598	9.23453	H	-4.82648	9.78456	-0.83694
H	8.98848	-4.51891	4.19917	H	-5.23449	10.85966	-3.01015
H	5.32734	-3.56240	6.25570	H	-1.64160	7.62931	3.82699
H	7.83353	-0.42465	4.74665	H	-0.61144	10.28976	0.61148
H	-17.06640	1.06769	-1.64874	H	-4.13804	7.86435	0.34194
H	-18.52639	3.08241	-1.62013	H	-4.37461	13.43815	-9.03402
H	-17.89629	5.01523	-0.14297	H	-3.00752	15.47013	-9.49518
H	-15.79595	4.83832	1.21651	H	-1.40704	16.27715	-7.73502
H	-13.83378	3.59608	1.88001	H	-1.23805	14.98389	-5.59380
H	-10.81623	1.54055	0.32232	H	-1.92109	13.10068	-4.22051
H	-8.76834	1.92629	1.64135	H	-8.98850	1.37190	6.01600
H	-11.14163	2.41567	5.19532	H	-5.32824	3.63281	6.21379
H	-13.18480	2.02745	3.88285	H	-7.83618	3.89298	2.74299
H	-5.56419	-2.02655	15.63618	H	-17.06692	0.88272	1.75326
H	-7.45776	-3.47902	16.34843	H	-18.52554	-0.15005	3.48439
H	-9.44079	-3.71555	14.82376	H	-17.89360	-2.39512	4.41991
H	-9.41873	-2.48453	12.64058	H	-15.79278	-3.48270	3.58645
H	-8.33177	-0.97830	11.10581	H	-13.83122	-3.43515	2.17817
H	-8.63587	1.19680	10.49951	H	-10.81526	-1.05728	1.17769
H	-8.90911	2.27627	8.32018	H	-8.76682	-2.39122	0.85116
H	-4.83170	1.33412	7.32887	H	-11.13860	-5.71388	-0.50432
H	-4.55498	0.23307	9.51662	H	-13.18234	-4.38422	-0.18347
H	7.32436	13.46400	6.85593	H	-5.55728	-12.53508	-9.57022
H	8.23828	15.34673	5.50062	H	-7.45197	-12.42995	-11.18320

H	-9.43677	-10.99368	-10.62586
H	-9.41531	-9.71647	-8.46938
H	-8.32811	-9.13697	-6.39878
H	-8.63082	-9.69732	-4.21166
H	-8.90453	-8.34971	-2.18735
H	-4.82863	-7.01598	-2.50959
H	-4.55141	-8.35990	-4.55689
H	7.33632	-12.66209	8.23059
H	8.25072	-12.42833	10.53841
H	7.18851	-10.76944	12.09728
H	5.25471	-9.41236	11.25845
H	3.70134	-8.85021	9.48234
H	1.32848	-10.56886	6.55967
H	-0.66035	-9.17676	6.12307
H	1.51666	-5.68919	7.37949
H	3.50095	-7.07655	7.81345
H	-5.22914	-2.82746	10.91126
H	-4.82114	-4.17134	8.89312
H	-0.59064	-3.50509	9.31095
H	-0.99530	-2.20291	11.34573
H	-1.63565	-7.13012	4.69318
H	-0.60499	-5.67361	8.60408
H	-4.13381	-4.23126	6.63968
H	-4.37194	1.09859	16.15707
H	-3.00290	0.48280	18.14627
H	-1.39924	-1.44221	17.96294
H	-1.22944	-2.64817	15.77140
H	-1.91376	-2.89598	13.45437
H	-8.98560	-5.90147	-1.81834
H	-5.32404	-7.20147	0.03934
H	-7.83328	-4.32812	2.00180
H	-17.06487	-1.97135	-0.10786
H	-18.52331	-2.95458	-1.86792
H	-17.89230	-2.64060	-4.27999
H	-15.79263	-1.37315	-4.80517
H	-13.83171	-0.17636	-4.05994
H	-13.18398	2.34369	-3.70014
H	-11.14093	3.28731	-4.69197
H	-8.76722	0.45205	-2.49429
H	-10.81504	-0.49854	-1.50156
H	-5.57037	14.55200	-6.07017
H	-7.46506	15.89477	-5.17039
H	-9.44751	14.69237	-4.20233
H	-9.42388	12.18622	-4.17430
H	-8.33580	10.10407	-4.70955
H	-4.55800	8.12358	-4.96189
H	-4.83306	5.67826	-4.82184
H	-8.91054	6.06304	-6.13397
H	-8.63897	8.49025	-6.28860
H	7.33181	-0.78793	-15.08413
H	8.24758	-2.90198	-16.03774
H	7.18783	-5.08287	-15.38141
H	5.25489	-5.03793	-13.78562
H	3.70078	-3.78308	-12.40915
H	1.32470	-0.39527	-12.43477
H	-0.66345	-0.71506	-11.01039
H	1.51674	-3.54501	-8.61890
H	3.50038	-3.22535	-10.03790
H	-5.22571	-8.03959	-7.90157
H	-4.81965	-5.61947	-8.05733
H	-0.58831	-6.31128	-7.69306
H	-0.99105	-8.72484	-7.58192
H	-1.63770	-0.50055	-8.52224
H	-0.60475	-4.61504	-9.21712
H	-4.13307	-3.63731	-6.98389
H	-4.36190	-14.54489	-7.12396

H	-2.99167	-15.95894	-8.65152	O	3.47599	6.50548	-3.30810
H	-1.38974	-14.83663	-10.22798	O	4.39017	6.51945	-2.14526
H	-1.22274	-12.33551	-10.17790	O	-4.39489	5.11576	4.56988
H	-1.90908	-10.20535	-9.23476	O	-3.48092	6.11620	3.97671
H	-8.98904	4.51939	-4.19921	O	-4.39060	-6.51938	2.14587
H	-5.32778	3.56297	-6.25558	O	-3.47635	-6.50537	3.30865
H	-7.83401	0.42516	-4.74671	O	3.48196	-6.11789	-3.97741
H	5.32928	-3.63460	-6.21458	O	4.39594	-5.11748	-4.57062
H	7.83725	-3.89465	-2.74378	O	3.47753	-0.38438	7.28749
O	-4.39282	1.39833	-6.71801	O	4.39238	-1.39777	6.71805
O	-3.47799	0.38494	-7.28747	H	7.45162	12.43111	11.18328

7.3 Calculated energy of cage 1-5

Table S5 Calculated energy of cage **1**-**5** after DFT structural optimization.

	Energy (Hartree a.u.)
Cage **1**	-20247.0344910
Cage **2**	-8048.25180331
Cage **3**	-14147.7891744
Cage **4**	-14598.7336504
Cage **5**	-21148.8859770

7.4 Energy calculations of structural transformation between cages

Reaction 1

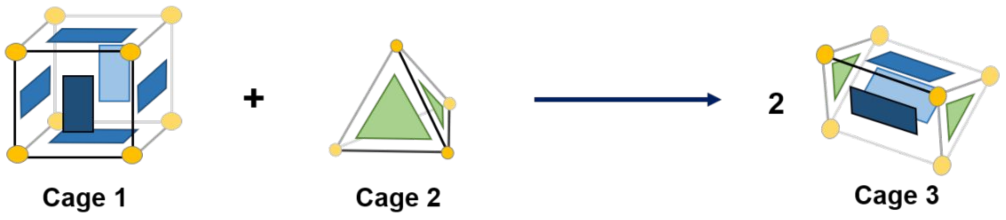

$$E_1 = 2E(\text{cage } 3) - [E(\text{cage } 1) + E(cage\ 2)] = -0.29205\ a.u. = -766.64\ kJ/mol$$

Reaction 2

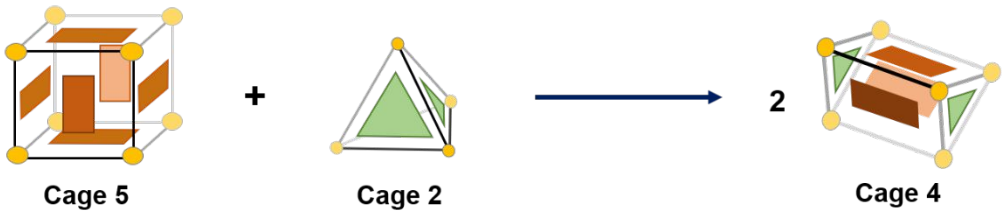

$$E_2 = 2E(\text{cage } 4) - [E(\text{cage } 5) + E(cage\ 2)] = -0.32952\ a.u. = -864.99\ kJ/mol$$

Figure S113: Calculated energy of Reactions 1 and 2.

By comparing the calculated energies of Reactions 1 and 2, we concluded that Reaction 2 was more energetically favorable.

Reaction 3

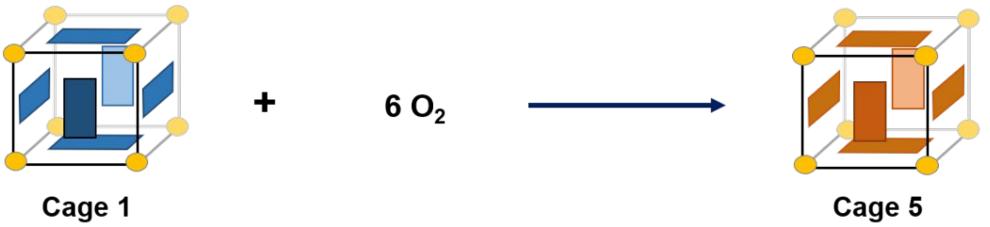

$$E_3 = E(\text{cage } 5) - [E(\text{cage } 1) + 6E(O_2)] = 0.068755\ a.u. = 180.48\ kJ/mol$$

Reaction 4

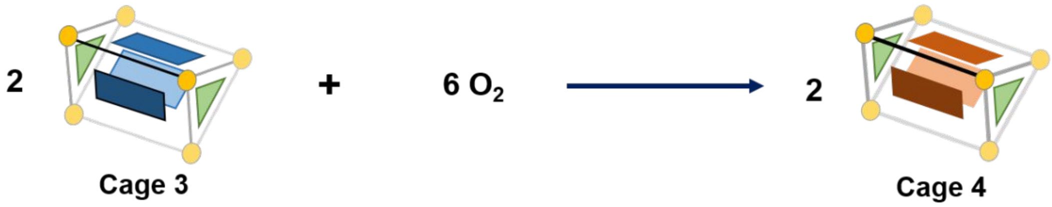

$$E_4 = 2E(\text{cage } 4) - [2E(\text{cage } 3) + 6E(O_2)] = 0.031289\ a.u. = 82.134\ kJ/mol$$

Figure S114: Calculated energy of Reaction 3 and Reaction 4. Energy of O_2 was calculated via same method (B3LYP 6-31G(d)) as cage **1**-**5** with energy number of -150.320040192 a.u..

By comparing the calculated energies of Reactions 3 and 4, we concluded that cage **4** was an energetically favorable structure driving the Reaction 2 into an integrative self-sorting system (only cage **4** left).

7.5 Internal cavity volume of cage 1, 3 and 4

In order to determine the available void spaces within the cages, MoloVol calculations[9] based on the modelled structures obtained in this study were performed. A virtual probe with a radius of 1.4 Å was employed to cage **4**, and because the more porous structure of cage **3** and cage **5** a virtual probe with a radius of 2.0 Å was employed to cage **3**, and a radius of 1.66 Å was employed to cage **5.**

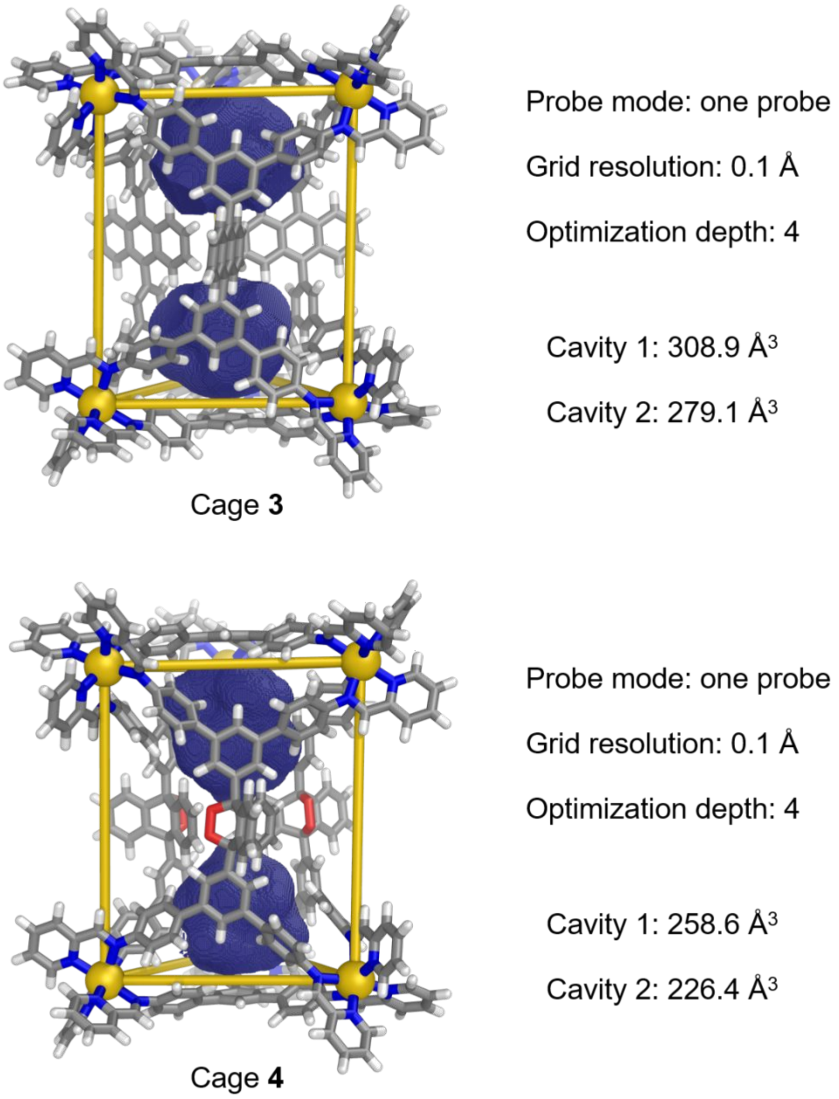

Figure S115: Molovol[9] calculated void spaces (shown in deep blue mesh) within the structures of cage **3** and cage **4**.

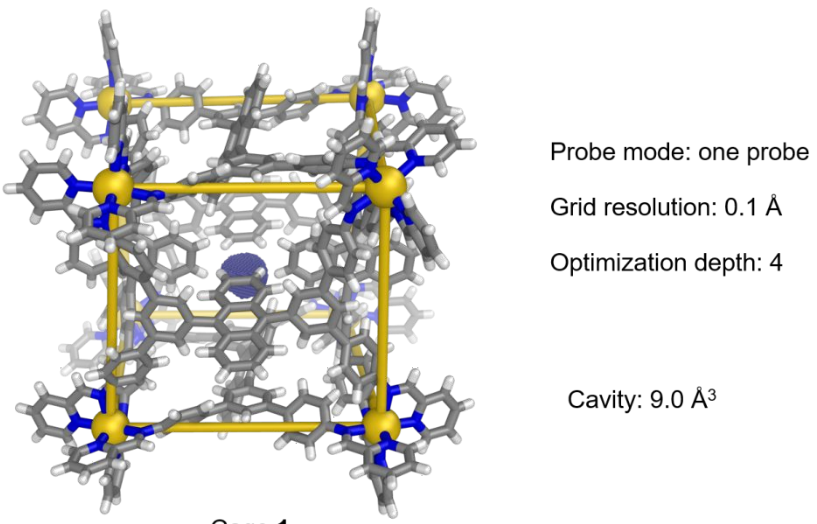

Cage **1**

Figure S116: Molovol[9] calculated void spaces (shown in deep blue mesh) within the crystal structure of cage **1**.

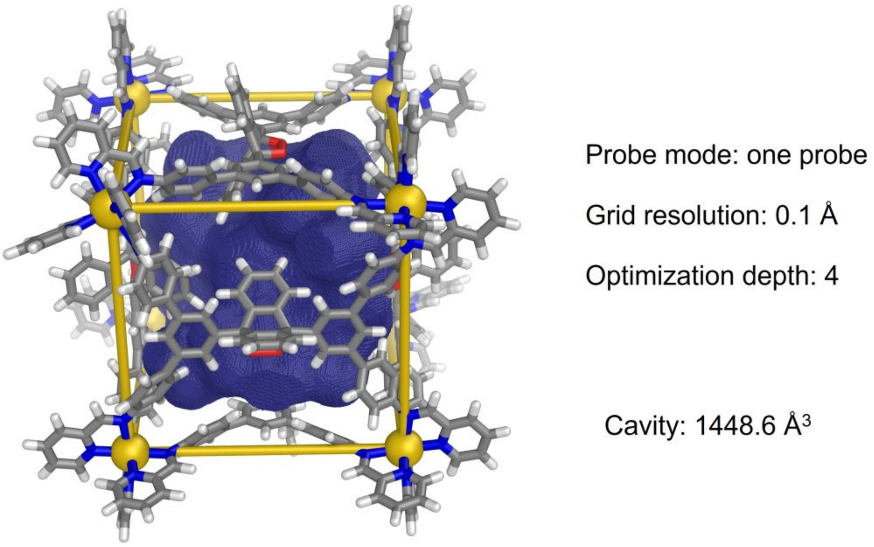

Cage **5**

Figure S117: Molovol[9] calculated void spaces (shown in deep blue mesh) within the structure of cage **5**.

8 X-ray crystallography

Data were collected at Beamline I19 of Diamond Light Source employing silicon double crystal monochromated synchrotron radiation (0.6889 Å) with ω and ψ scans at 100(2) K.[10] Data integration and reduction were undertaken with Xia2.[11-13] Subsequent computations were carried out using the WinGX-32 graphical user interface.[14] Multi-scan empirical absorption corrections were applied to the data using the AIMLESS[15] tool in the CCP4 suite.[16] The structures were solved by direct methods using SHELXT[17] then refined and extended with SHELXL.[18] In general, non-hydrogen atoms with occupancies greater than 0.5 were refined anisotropically. Carbon-bound hydrogen atoms were included in idealised positions and refined using a riding model. Disorder was modelled using standard crystallographic methods including constraints, restraints and rigid bodies where necessary. Crystallographic data along with specific details pertaining to the refinement follow. Crystallographic data have been deposited with the CCDC (2255439 for cage **1**).

[Zn8L6]·16NTf4·16.75MeCN·10.5Et2O·0.75H2O [+ solvent]

Formula $C_{551.50}H_{456.75}F_{96}N_{80.75}O_{75.25}S_{32}Zn_8$, *M* 12792.08, Triclinic, space group P-1 (#2), *a* 26.1066(4), *b* 32.5730(7), *c* 36.9750(8) Å, *a* 88.500(2), *b* 73.586(2), *g* 87.592(2)°, *V* 30131.0(11) Å^3, D_c 1.410 g cm^{-3}, *Z* 2, crystal size 0.050 by 0.040 by 0.040 mm, colour yellow, habit block, temperature 100(2) Kelvin, *l*(Synchrotron) 0.6889 Å, *m*(Synchrotron) 0.483 mm^{-1}, T(Analytical)$_{min,max}$ 0.9901981473973039, 1.0, 2q_{max} 40.21, *hkl* range -26 26, -32 32, -36 36, *N* 152705, N_{ind} 61235(R_{merge} 0.1074), N_{obs} 18905(I > 2s(I)), N_{var} 7807, residuals* *R*1(*F*) 0.1214, *wR*2(F^2) 0.3316, GoF(all) 0.882, D$r_{min,max}$ -0.612, 1.142 e$^-$ Å^{-3}.

$^*R1 = S||F_o| - |F_c||/S|F_o|$ for $F_o > 2s(F_o)$; $wR2 = (Sw(F_o^2 - F_c^2)^2/S(wF_c^2)^2)^{1/2}$ all reflections

$w=1/[s^2(F_o^2)+(0.1675P)^2]$ where $P=(F_o^2+2F_c^2)/3$

Specific refinement details:

The crystals of [Zn8L6]·16NTf2·16.75MeCN·10.5Et2O·0.75H2O [+ solvent] were grown by diffusion of diethyl ether into an acetonitrile solution of the complex. The crystals employed immediately lost solvent after removal from the mother liquor and rapid handling prior to flash cooling in liquid nitrogen was required to collect data. Despite these measures and the use of synchrotron radiation few reflections at greater than 1.0 Å resolution were observed and the data were trimmed accordingly. Furthermore, there was a significant drop-off in diffraction intensity after around 1.5 Å resolution resulting in a low ratio of observed/unique reflections. Nevertheless, the

quality of the data is far more than sufficient to establish the connectivity of the structure. The asymmetric unit was found to two crystallographically independent halves of a Zn_8L_6 assembly and associated counterions and solvent molecules. Due to the limited resolution bond lengths and angles within pairs of chemically identical organic ligands were restrained to be similar to each other and thermal parameter restraints (SIMU, RIGU) were applied to all atoms except for zinc.

The anions within the structure show evidence of substantial disorder. Four of the located triflimide anions were modelled as disordered over two locations and one lattice site was modelled as a disordered mixture of triflimide and diethyl ether. Substantial bond length and thermal parameter restraints were applied to facilitate stable refinement of the disordered anions and most low occupancy anions were modelled with isotropic thermal parameters. The occupancies of all located anions were allowed to freely refine which resulted in a discrepancy of ca. 2.1 anions per Zn_8L_6 assembly. Some solvent molecules were also modelled with partial occupancy. Similarity restraints were applied to the diethyl ether molecules and bond length restraints were applied to some acetonitrile molecules. The hydrogen atoms of some water and acetonitrile molecules could not be located in the electron density map and were therefore not included in the model.

Further reflecting the solvent loss and poor diffraction properties there is a significant amount of void volume in the lattice containing smeared electron density from disordered solvent and the remaining anions. Consequently, the SQUEEZE[19] function of PLATON[20] was employed to remove the contribution of the electron density associated with these remaining anions and further highly disordered solvent, which gave a potential solvent accessible void of 1521 $Å^3$ per unit cell (a total of approximately 484 electrons). Diffuse solvent molecules could not be assigned to acetonitrile or diethyl ether and were therefore not included in the formula. Consequently, the molecular weight and density given above are underestimated.

CheckCIF gives 1 A and 3 B level alerts, all resulting from the limited resolution of the data and the water molecule for which hydrogen atoms could not be modelled.

9 References

(1) Davies, J. A.; Tarzia, A.; Ronson, T. K.; Auras, F.; Jelfs, K. E.; Nitschke, J. R. *Angew. Chem. Int. Ed.* **2023**, *62*, e202217987.

(2) Castilla, A. M.; Ronson, T. K.; Nitschke, J. R. *J. Am. Chem. Soc.* **2016**, *138*, 2342-2351.

(3) Jiménez, A.; Bilbeisi, R. A.; Ronson, T. K.; Zarra, S.; Woodhead, C.; Nitschke, J. R. *Angew. Chem. Int. Ed.* **2014**, *53*, 4556.

(4) Stephens, P. J.; Devlin, F. J.; Chabalowski, C. F.; Frisch, M. J. *J. Phys. Chem.* **1994**, *98*, 11623.

(5) Hay, P. J.; Wadt, W. R. *J. Chem. Phys.* **1985**, *82*, 270.

(6) Ditchfield, R.; Hehre, W. J.; Pople, J. A. *J. Chem. Phys.* **1971**, *54*, 724.

(7) Petersson, G. A.; Al‐Laham, M. A. *J. Chem. Phys.* **1991**, *94*, 6081.

(8) Frisch, M. J.; Trucks, G. W.; Schlegel, H. B.; Scuseria, G. E.; Robb, M. A.; Cheeseman, J. R.; Scalmani, G.; Barone, V.; Petersson, G. A.; Nakatsuji, H.; Li, X.; Caricato, M.; Marenich, A. V.; Bloino, J.; Janesko, B. G.; Gomperts, R.; Mennucci, B.; Hratchian, H. P.; Ortiz, J. V.; Izmaylov, A. F.; Sonnenberg, J. L.; Williams; Ding, F.; Lipparini, F.; Egidi, F.; Goings, J.; Peng, B.; Petrone, A.; Henderson, T.; Ranasinghe, D.; Zakrzewski, V. G.; Gao, J.; Rega, N.; Zheng, G.; Liang, W.; Hada, M.; Ehara, M.; Toyota, K.; Fukuda, R.; Hasegawa, J.; Ishida, M.; Nakajima, T.; Honda, Y.; Kitao, O.; Nakai, H.; Vreven, T.; Throssell, K.; Montgomery Jr., J. A.; Peralta, J. E.; Ogliaro, F.; Bearpark, M. J.; Heyd, J. J.; Brothers, E. N.; Kudin, K. N.; Staroverov, V. N.; Keith, T. A.; Kobayashi, R.; Normand, J.; Raghavachari, K.; Rendell, A. P.; Burant, J. C.; Iyengar, S. S.; Tomasi, J.; Cossi, M.; Millam, J. M.; Klene, M.; Adamo, C.; Cammi, R.; Ochterski, J. W.; Martin, R. L.; Morokuma, K.; Farkas, O.; Foresman, J. B.; Fox, D. J. Gaussian 16 Rev. B.01, Wallingford, CT, 2016.

(9) Maglic, J. B., Lavendomme, R. *J. Appl. Cryst.*, **2022**, *55*, 1033.

(10) Allan, D.; Nowell, H.; Barnett, S.; Warren, M.; Wilcox, A.; Christensen, J.; Saunders, L.; Peach, A.; Hooper, M.; Zaja, L.; Patel, S.; Cahill, L.; Marshall, R.; Trimnell, S.; Foster, A.; Bates, T.; Lay, S.; Williams, M.; Hathaway, P.; Winter, G.; Gerstel, M.; Wooley, R. *Crystals* **2017**, *7*, 336.

(11) Evans, P. *Acta Cryst.* **2006**, *D62*, 72.

(12) Winter, G. *J. Appl. Crystallogr.* **2010**, *43*, 186.

(13) Winter, G.; Waterman, D. G.; Parkhurst, J. M.; Brewster, A. S.; Gildea, R. J.; Gerstel, M.; Fuentes-Montero, L.; Vollmar, M.; Michels-Clark, T.; Young, I. D.; Sauter, N. K.; Evans, G. *Acta Cryst.* **2018**, *D74*, 85.

(14) Farrugia, L. *J. Appl. Crystallogr.* **2012**, *45*, 849.

(15) Evans, P. R.; Murshudov, G. N. *Acta Cryst.* **2013**, *D69*, 1204.

(16) Winn, M. D.; Ballard, C. C.; Cowtan, K. D.; Dodson, E. J.; Emsley, P.; Evans, P. R.; Keegan, R. M.; Krissinel, E. B.; Leslie, A. G. W.; McCoy, A.; McNicholas, S. J.; Murshudov, G. N.; Pannu, N. S.; Potterton, E. A.; Powell, H. R.; Read, R. J.; Vagin, A.; Wilson, K. S. *Acta Cryst.* **2011**, *D67*, 235.

(17) Sheldrick, G. *Acta. Cryst.* **2015**, *A71*, 3.

(18) Sheldrick, G. M. *Acta. Cryst.* **2015**, *C71*, 3.

(19) van der Sluis, P.; Spek, A. L. *Acta Cryst.* **1990**, *A46*, 194.

(20) Spek, A. L. PLATON: A Multipurpose Crystallographic Tool; Utrecht University: Utrecht, The Netherlands, 2008.
